# *Bacteroides salyersiae* Is a Candidate Probiotic Species with Potential Anti-Colitis Properties in the Human Colon: First Evidence from an In Vivo Mouse Model

**DOI:** 10.3390/nu16172918

**Published:** 2024-09-01

**Authors:** Wei Dai, Youjing Lv, Min Quan, Mingfeng Ma, Qingsen Shang, Guangli Yu

**Affiliations:** 1 Key Laboratory of Marine Drugs of Ministry of Education, Shandong Provincial Key Laboratory of Glycoscience and Glycotechnology, School of Medicine and Pharmacy, Ocean University of China, Qingdao 266003, China; daiwei3266@stu.ouc.edu.cn (W.D.); lvyoujing1988@163.com (Y.L.); q15265991920@163.com (M.Q.); mmf621121@163.com (M.M.); 2 Laboratory for Marine Drugs and Bioproducts, Laoshan Laboratory, Qingdao 266237, China; 3 Qingdao Marine Biomedical Research Institute, Qingdao 266071, China

**Keywords:** *Bacteroides salyersiae*, ulcerative colitis, dextran sulfate sodium, gut microbiota, probiotic, bacterial metabolites, equol, *Bifidobacterium pseudolongum*, anti-colitis properties, inflammatory bowel disease

## Abstract

Previous studies have indicated a critical role of intestinal bacteria in the pathogenesis of ulcerative colitis (UC). *B. salyersiae* is a commensal species from the human gut microbiota. However, what effect it has on UC development has not been investigated. In the present study, we explored this issue and demonstrated for the first time that oral administration of *B. salyersiae* CSP6, a bacterium previously isolated from the fecal sample of a healthy individual, protected against dextran sulfate sodium (DSS)-induced colitis in C57BL/6J mice. In particular, *B. salyersiae* CSP6 improved mucosal damage and attenuated gut dysbiosis in the colon of DSS-fed mice. Specifically, *B. salyersiae* CSP6 decreased the population of pathogenic *Escherichia-Shigella* spp. and increased the abundance of probiotic *Dubosiella* spp. and *Bifidobacterium pseudolongum.* Additionally, by reshaping the colonic microbiota, *B. salyersiae* CSP6 remarkably increased the fecal concentrations of equol, 8-deoxylactucin, and tiglic acid, three beneficial metabolites that have been well documented to exert strong anti-inflammatory effects. Altogether, our study provides novel evidence that *B. salyersiae* is a candidate probiotic species with potential anti-colitis properties in the human colon, which has applications for the development of next-generation probiotics.

## 1. Introduction

UC is a lifelong and chronic inflammatory disease that mainly occurs in the mucosa of the human colon [1,2,3]. UC has a profound impact on the daily life of an affected individual [1,2]. Patients with UC commonly present with rectal bleeding, fecal incontinence, increased stool frequency, and abdominal pain [1]. UC has gradually become a global challenge in health care in the past two decades. In 2023, it was estimated that there were 5.0 million cases of UC all around the world and the disease incidence is increasing at an alarming rate [1,3].

The precise etiology of UC is very complex as genetic predispositions, environmental exposures, dysregulated immune responses, and intestinal barrier defects are strongly implicated in this disease [1,2,3]. Recently, specific microbes from the human gut have been shown to play a significant role in the development of UC [4,5,6]. For example, pathogenic *E. coli*, *Bacteroides vulgatus*, and *Candida albicans* have been demonstrated to promote intestinal inflammation by producing harmful toxins and disrupting the tight junctions in epithelial cells [7,8,9]. In contrast, probiotic bacteria including *B. pseudolongum*, *Lactobacillus salivarius*, *Lactobacillus reuteri*, *Bacteroides thetaiotaomicron*, *Faecalibacterium prausnitzii*, *Bacteroides ovatus*, *Bacteroides xylanisolvens*, *Bacteroides uniformis*, *Bifidobacterium breve*, and *Parabacteroides distasonis* have been discovered to improve intestinal inflammation and attenuate mucosal damage in the colon [10,11,12,13,14,15,16,17,18,19]. Given that gut microbial dysbiosis is a well-established feature of UC in the clinic [1,4,20], these studies highlight an urgent need for further understanding of the roles of different bacteria in disease prevention and management.

*B. salyersiae* is a commensal species in the human gut [21,22]. Our recent study suggests that *B. salyersiae* plays a fundamental role in the degradation of different polysaccharides in our daily diet [23]. Nonetheless, what effect *B. salyersiae* has on UC development has not been investigated. We hypothesized that *B. salyersiae* is protective against the onset of UC in the human colon. We tested this possibility using a DSS-induced disease model in mice and found that *B. salyersiae* is a candidate probiotic species with potential anti-colitis properties.

## 2. Materials and Methods

### 2.1. Chemicals and Reagents

The well-established VI medium was used to culture *B. salyersiae* CSP6 as previously described [23]. Tryptone, yeast extract, and Tween 80 were obtained from Sigma-Aldrich (St. Louis, MO, USA). Hemin chloride, chondroitin sulfate, agar, and L-cysteine hydrochloride were acquired from Sangon Biotech (Shanghai, China).

All other analytical-grade chemicals, including NaCl, KCl, KH_2_PO_4_, MgSO_4_·7H_2_O, CaCl_2_·2H_2_O, MnCl_2_·4H_2_O, FeSO_4_·7H_2_O, CoCl_2_·H_2_O, ZnSO_4_·7H_2_O, CuSO_4_·5H_2_O, and NiCl_2_·6H_2_O, were purchased from Sinopharm Chemical (Shanghai, China). The DSS used for the mouse UC model was obtained from MP Biomedicals (Solon, OH, USA).

### 2.2. Animal Experiment

A total of 30 male, 7-week-old, specific-pathogen-free (SPF) C57BL/6J mice were obtained from Beijing Vital River Laboratory Animal Technology (Beijing, China). The mice were randomly divided into three groups upon arrival to the animal experiment center. The three groups consisted of the normal control group (NC, *n* = 10), the UC model group (MD, *n* = 10), and the *B. salyersiae* CSP6 treatment group (BS, *n* = 10). 

The animals in each group were housed in one cage. Because different samples were needed for the staining analysis, gut microbiome analysis, and fecal metabolome analysis, we included 10 mice in each group. Confounders including the order of treatments and animal cage location were controlled for the experiment. Randomization was used to allocate experimental units to the control and treatment groups. The randomization sequence was generated using computer-generated random numbers. Investigators directly involved in the animal experiment were blinded to the group allocation of the mice.

All experimental mice from both the BS group and the MD group were given DSS in their drinking water at a concentration of 2.2% (*w*/*v*). Mice from the NC group received no DSS during the whole experiment and thus served as a normal control. *B. salyersiae* CSP6 was isolated from the fecal sample of a healthy Chinese individual [23]. The human experiments were approved and supported by the Ethical Committee of the Ocean University of China, School of Medicine and Pharmacy (Permission No. OUC-2021–1011-01). 

The detailed isolation procedure, the ethical conditions for the studies, and the genomic information of this bacterium was published in our recent paper [23]. VI medium that contained chondroitin sulfate at a concentration of 8.0 g/L was used to culture *B. salyersiae* CSP6. The bacterial cells of *B. salyersiae* CSP6 were obtained in the exponential phase by centrifugation (8000× *g* for 15 min). Mice in the BS group were given *B. salyersiae* CSP6 orally at a dosage of about 1.68 × 10^9^ colony-forming units (CFUs)/day.

After 10 days of treatment, all the experimental mice were humanely sacrificed under deep anesthesia. The colon was harvested for hematoxylin and eosin (H&E) staining analysis. Three mice from the NC group and three mice from the MD group were excluded from the H&E staining analysis. This was because, at that time, we were also interested in studying the changes in the immune cells in the colons of DSS-fed mice. The whole colon samples of these six mice were used for the isolation and analyses of lamina propria lymphocytes. However, unfortunately, we did not succeed in this experiment.

The fecal content in the cecum and colon was collected and pooled for the analysis of intestinal microbiota and its metabolites. The symptom score of the mice was calculated based on different UC symptoms on the last day of the animal experiment. The histopathological score of the colon was determined based on the H&E staining results using the method previously described [17,18].

### 2.3. 16S rRNA Gene Amplicon High-Throughput Sequencing and Bioinformatics Analysis

About 70 mg of the fresh fecal samples from six different mice in each treatment group was collected for the analysis of the gut microbiota. The other mice were excluded from the analysis because we were not able to obtain enough fecal content for the analysis. The metagenomic DNA of the intestinal bacteria was extracted using a SPINeasy DNA kit for feces from MP Biomedicals (Solon, OH, USA). 

The obtained DNA was checked for quality, and the V3 to V4 hypervariable regions of the 16S rRNA gene were amplified using the 338F and 806R primers. The obtained amplicons were sequenced on an Illumina PE300 platform (San Diego, CA, USA) from Majorbio Bio-Pharm Biotechnology (Shanghai, China). Bioinformatic analyses of the sequencing data, including Wilcoxon rank-sum test analysis, Venn diagram analysis, principal components analysis (PCA), and heatmap analysis, were all conducted using the online tools from Majorbio Cloud Platform (www.majorbio.com, accessed on 10 July 2024). 

### 2.4. Metabolome Analysis

Briefly, about 60 mg of the fresh fecal samples from six different mice in each treatment group was collected for the metabolome analysis. The other mice were excluded from this analysis because we were not able to obtain enough fecal content for the analysis. After that, equal volumes of all samples were mixed first to obtain a pooled sample for quality control. The mass spectrometric data were collected under both the negative and positive mode. The raw data of mass spectrometry were preprocessed by Progenesis QI software (Version 2.0) (Waters Corporation, Milford, CT, USA). 

The fecal metabolites were identified with the help of the Human Metabolome Database (HMDB) (http://www.hmdb.ca/, accessed on 16 November 2023), Metlin (https://metlin.scripps.edu/, accessed on 16 November 2023), and Majorbio Database. Data analysis was conducted using the online tools from the Majorbio cloud platform (https://cloud.majorbio.com, accessed on 16 November 2023). PCA, partial least squares discriminant analysis (PLS-DA), and orthogonal partial least squares discriminant analysis (OPLS-DA) were performed by the R package ropls (Version 1.6.2) (http://www.bioconductor.org/, accessed on 16 November 2023). The significantly different metabolites were selected based on the variable importance in the projection (VIP) and the *p*-value of Student’s *t*-test, within the range of VIP > 1 and *p* < 0.05.

### 2.5. Statistical Analyses

All the data were expressed as the mean ± standard error of mean (SEM). The statistical analyses were performed using Student’s *t*-test and ANOVA with post hoc Tukey’s tests (GraphPad Prism Version 8.0.2; San Diego, CA, USA). The *p* values were adjusted for multiple-hypothesis testing. The false discovery rate (FDR) was applied in multiple-hypothesis testing to correct the *p* values for multiple comparisons. The Benjamini and Hochberg method was used for the *p* value correction. A predetermined FDR cutoff (FDR < 0.05) was used for the analyses. 

## 3. Results

### 3.1. Oral Intake of B. salyersiae CSP6 Protected against DSS-Induced Colitis in C57BL/6J Mice

DSS-induced UC is usually characterized by diarrhea, colonic inflammation, and ulceration, which is very similar to human disease [24,25,26]. This model has been widely used throughout the world [25,26]. In addition, it has served as a valuable tool for investigating disease pathogenesis and evaluating new treatment options [24]. Therefore, in the present study, we applied this model and explored the potential therapeutic effect of *B. salyersiae* CSP6 on UC in mice. 

**Figure 1:**
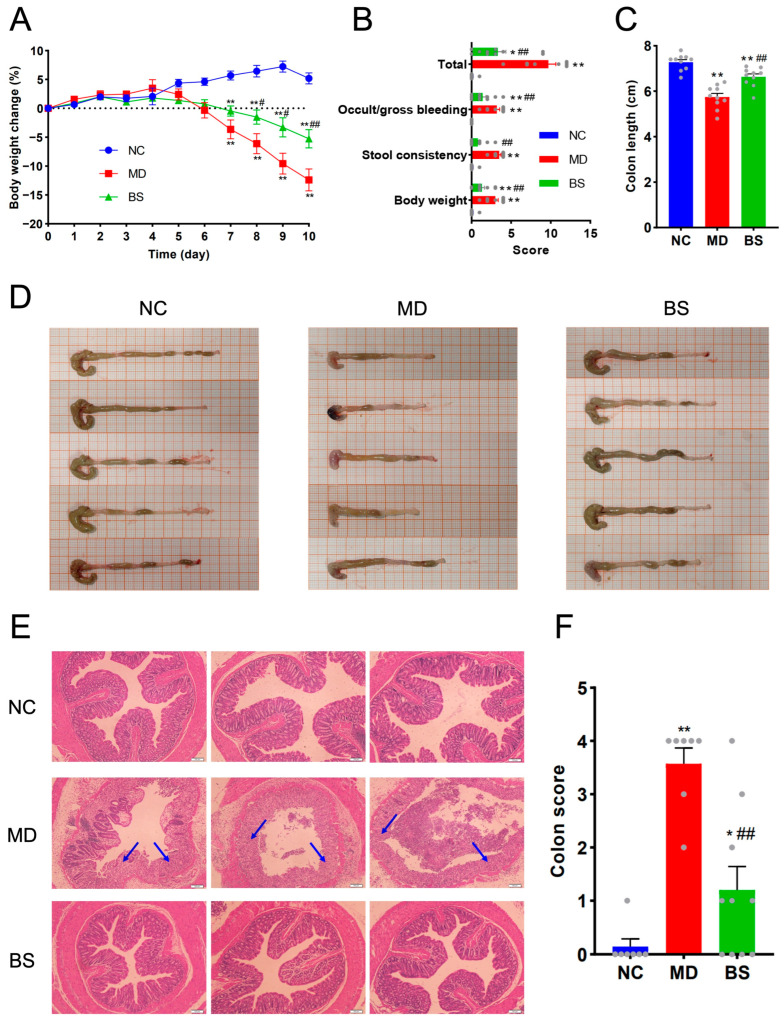
*B. salyersiae* CSP6 protected against DSS-induced colitis in C57BL/6J mice. Body weight change (**A**). NC group, *n* = 10; MD group, *n* = 10; BS group, *n* = 10. Symptom score (**B**). NC group, *n* = 10; MD group, *n* = 10; BS group, *n* = 10. Colon length (**C**). NC group, *n* = 10; MD group, *n* = 10; BS group, *n* = 10. Representative images of the colon (**D**). Representative H&E staining images of the colon sections (**E**). Arrows indicate the lesion sites of the colon. The scale bar indicates 100 μm. Histopathological score (**F**). NC group, *n* = 7; MD group, *n* = 7; BS group, *n* = 10. * *p* < 0.05 versus NC group; ** *p* < 0.01 versus NC group; # *p* < 0.05 versus MD group; ## *p* < 0.01 versus MD group. Each gray dot in the panels (**B**,**C**,**F**) represents the corresponding data of one mouse in the experiment.

Interestingly and expectedly, oral intake of *B. salyersiae* CSP6 successfully protected against the development of chemically-induced UC in rodents (Figure 1). Specifically, *B. salyersiae* CSP6 administration for ten days significantly slowed the body weight loss in diseased mice (Figure 1A). In addition, *B. salyersiae* CSP6 treatment improved stool consistency and reduced the occurrence of bleeding complications in the intestine of DSS-fed mice (Figure 1B). Additionally, the shortening of the colon from rectum to cecum in UC mice was also found to be remarkably improved in response to *B. salyersiae* CSP6 treatment (Figure 1C,D). Moreover, as evidenced by H&E staining, the supplementation of *B. salyersiae* CSP6 beneficially alleviated the intestinal mucosal damage in UC mice (Figure 1E,F). Taken together, our results indicated that *B. salyersiae* CSP6 administration was protective against UC development in mice.

### 3.2. B. salyersiae CSP6 Changed the Structure of the Intestinal Microbiota and Attenuated Gut Dysbiosis in DSS-Fed Mice

**Figure 2:**
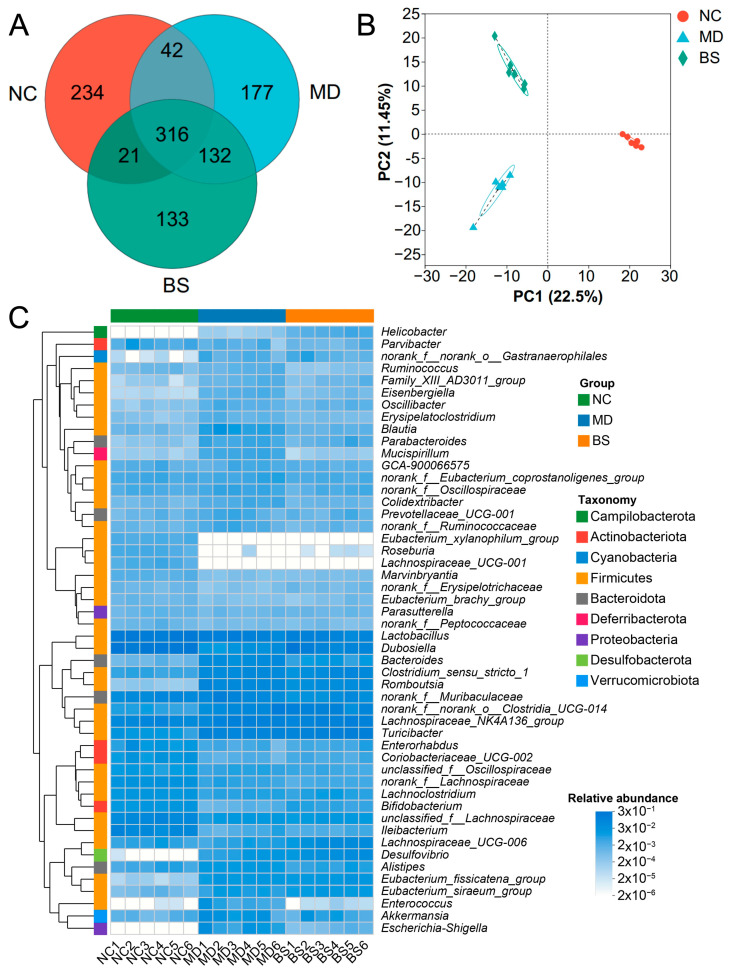
*B. salyersiae* CSP6 modulated intestinal microbiota in diseased mice. A Venn diagram showing the distribution of shared operational taxonomic units (OTUs) of the intestinal microbiota among different groups (**A**). The numbers are the amount of different OTUs in each group. PCA of the gut microbiota (**B**). A heatmap showing the compositional differences in the colonic microbiota at the genus level (**C**). NC group, *n* = 6; MD group, *n* = 6; BS group, *n* = 6.

Previous studies have illustrated that the gut microbiota contributes significantly to the development and treatment of UC [4,5,6]. In this regard, to understand how *B. salyersiae* CSP6 improved UC in DSS-fed mice, we next sought to explore the impact of *B. salyersiae* CSP6 on the gut microbiota. Intriguingly, as indicated by the PCA and Venn diagram analysis, we found that *B. salyersiae* CSP6 administration significantly changed the structure of the gut microbiota in UC mice (Figure 2A,B). 

**Figure 3:**
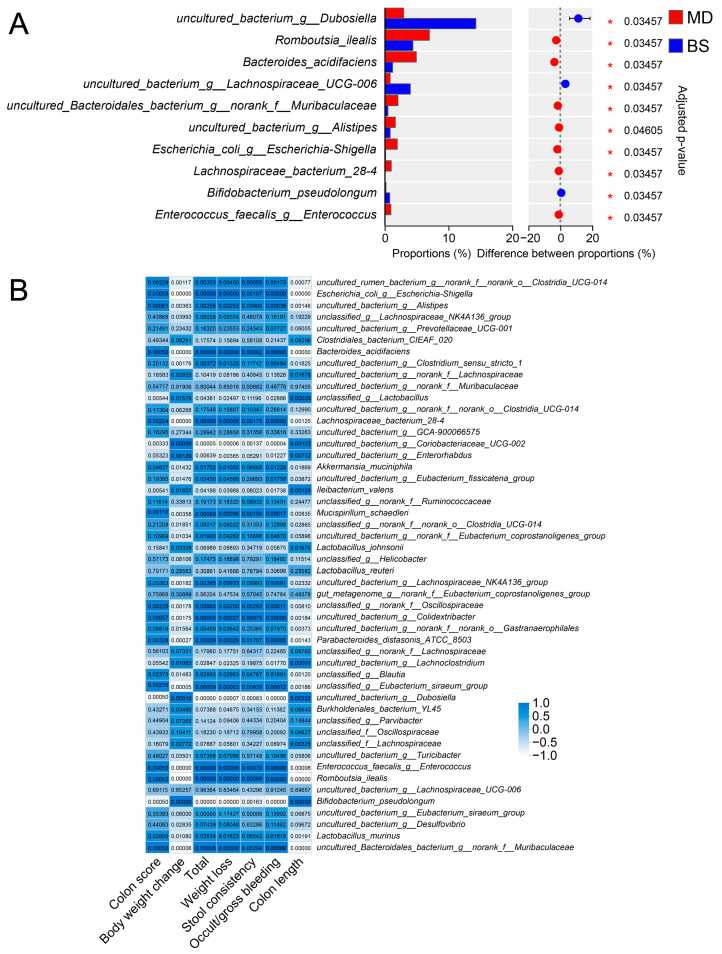
*B. salyersiae* CSP6 attenuated gut dysbiosis in DSS-fed mice. Wilcoxon rank-sum test analysis (**A**). The numbers represent adjusted *p* values for the bacteria. Heatmap of the correlation between different gut bacteria and disease parameters (**B**). The numbers represent adjusted *p* values for the bacteria. NC group, *n* = 6; MD group, *n* = 6; BS group, *n* = 6. * *p* < 0.05.

Further analysis suggested that *B. salyersiae* CSP6 modulated the gut microbiota at different taxonomic levels (Figure 2C and Figure S1). Specifically, *B. salyersiae* CSP6 decreased the populations of pathogenic *Escherichia-Shigella* spp. and increased the abundances of probiotic *Dubosiella* spp. and *B. pseudolongum* (Figure 3A). Additionally, the abundance of *Escherichia-Shigella* spp. was positively correlated with UC severity, while that of *Dubosiella* spp. and *B. pseudolongum* were negatively associated with UC severity (Figure 3B). Altogether, these results suggested that *B. salyersiae* CSP6 treatment attenuated gut dysbiosis in DSS-fed mice.

### 3.3. B. salyersiae CSP6 Modulated the Composition of Intestinal Metabolites in UC Mice and Increased the Fecal Concentrations of Anti-Inflammatory Equol, 8-Deoxylactucin, and Tiglic Acid

**Figure 4:**
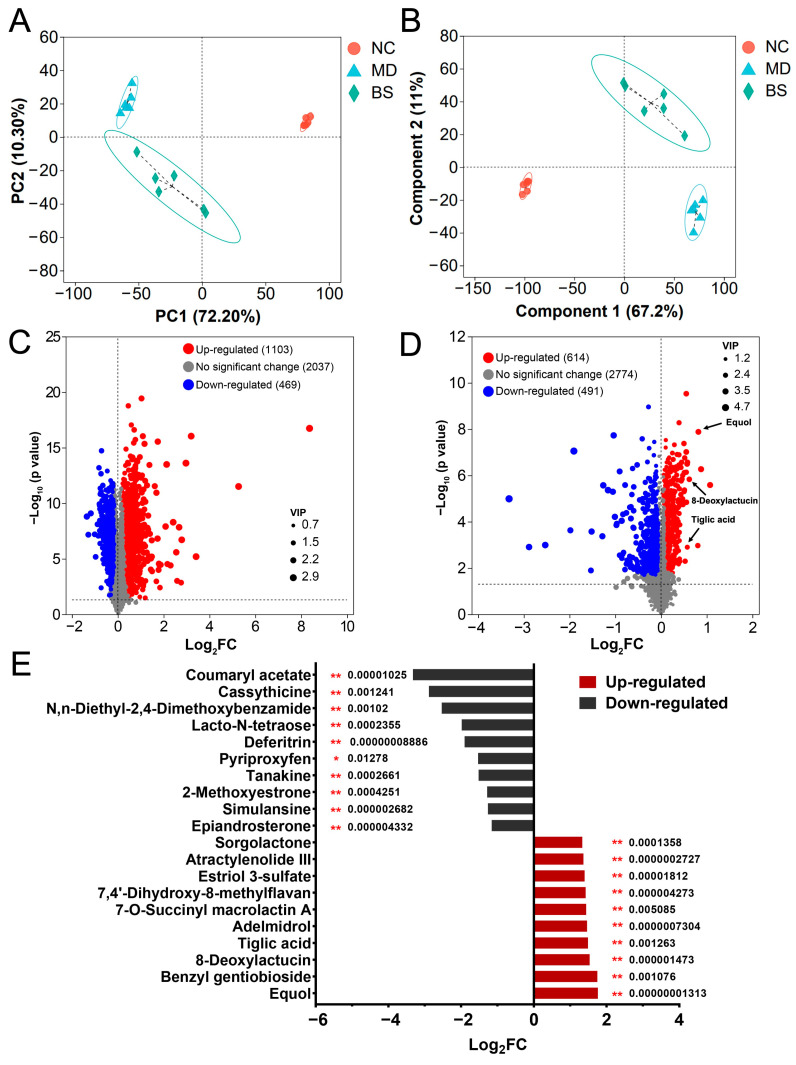
*B. salyersiae* CSP6 modulated the composition of intestinal metabolites in diseased mice by increasing the concentrations of anti-inflammatory equol, 8-deoxylactucin, and tiglic acid. PCA of the metabolites (**A**). PLS-DA of the metabolites (**B**). Volcano plot of the metabolites in the MD group vs. NC group (**C**). Volcano plot of the metabolites in the BS group vs. MD group (**D**). Butterfly plot of the representative metabolites in the BS group vs. MD group (**E**). The numbers represent adjusted *p* values for the metabolites. NC group, *n* = 6; MD group, *n* = 6; BS group, *n* = 6. The Benjamini and Hochberg method was used for the *p* value correction. A predetermined FDR cutoff (FDR < 0.05) was used for the analyses. * *p* < 0.05; ** *p* < 0.01.

Given the pivotal role of different bacterial metabolites in mediating the anti-colitis effect of probiotic bacteria [5,27,28], we next analyzed the fecal metabolome of the mice in different groups (Figure 4 and Figure S2). PCA and PLS-DA both indicated that intake of *B. salyersiae* CSP6 induced a considerable shift in the metabolite profile in DSS-fed mice (Figure 4A,B). Specifically, 1103 metabolites (FDR < 0.05) were identified to be up-regulated while 469 metabolites (FDR < 0.05) were identified to be down-regulated in the MD group, as compared to those in the NC group (Figure 4C and Table S1). Similarly, 614 metabolites (FDR < 0.05) were identified to be up-regulated, while 491 metabolites (FDR < 0.05) were identified to be down-regulated in the BS group, as compared to those in the MD group (Figure 4D and Table S2). 

Moreover, it is of interest to note that the supplementation of *B. salyersiae* CSP6 significantly increased the intestinal concentrations of equol, 8-deoxylactucin, and tiglic acid (Figure 4D,E). Intriguingly, these three metabolites have been well documented to exert strong anti-inflammatory effects [29,30,31,32,33]. Additionally, the abundances of these three metabolites were all negatively associated with disease symptoms (Figure S3). Taken together, our results indicated that *B. salyersiae* CSP6 modulated the composition of intestinal metabolites in UC mice and increased the fecal concentrations of anti-inflammatory equol, 8-deoxylactucin, and tiglic acid.

## 4. Discussion

In the last two decades, mounting evidence has demonstrated the involvement of specific gut microbes in the progression of UC [4,5,6,20]. *Bacteroides* spp. are well-known obligate anaerobes that dominate the human gut microbiota [21,34]. Recent studies have found that the intestinal populations of *Bacteroides* spp. are significantly lower in UC patients [35,36]. Given that certain bacteria from *Bacteroides* spp. have been proposed as next-generation probiotics [37,38], these results indicate that *Bacteroides* spp. might be protective against the development of UC in humans. Indeed, *B. thetaiotaomicron*, *B. uniformis*, *B. xylanisolvens*, and *B. ovatus* have all been shown to alleviate UC in preclinical mouse models [15,16,17,18]. 

*B. salyersiae* is a commensal bacterium in the human colon [21,22,34]. Preceding studies have shown that *B. salyersiae* plays a fundamental role in the degradation of dietary polysaccharides in the intestine [23,39,40,41]. Specifically, *B. salyersiae* could degrade and ferment arabinogalactan, α-mannan, inulin, chondroitin sulfate, and hyaluronic acid in our daily diet [23,39,40,41]. Nonetheless, what effect *B. salyersiae* has on UC development has not been elucidated. 

We hypothesized that *B. salyersiae* is protective against the onset of UC in the human colon. We tested this possibility using a DSS-induced disease model in mice and found that *B. salyersiae* is a candidate probiotic species with potential anti-colitis properties. Our results suggest that *B. salyersiae* might be used as a next-generation probiotic candidate for the prevention and potential treatment of UC.

*Dubosiella newyorkensis* is a probiotic SCFA-producing bacterium in the gut [42,43]. Recent studies have indicated that oral intake of *D. newyorkensis* could ameliorate DSS-induced colitis by rebalancing the Treg/Th17 responses and improving the integrity of the mucosal barrier in mice [42]. Similarly, *B. pseudolongum* supplementation was also found to be able to attenuate colitis by increasing the intestinal proportion of Foxp3+T cells and modulating the Pparγ/STAT3 pathway in DSS-fed mice [11]. In the present research, the anti-colitis effect of *B. salyersiae* was found to be associated with decreased populations of pathogenic *Escherichia-Shigella* spp. and increased abundances of probiotic *Dubosiella* spp. and *B. pseudolongum* in diseased mice. These results suggested that, to some extent, the attenuation of gut dysbiosis might have contributed to the therapeutic effect of *B. salyersiae* on UC in DSS-fed mice. 

Equol, 8-deoxylactucin, and tiglic acid are three bacterial metabolites with robust anti-inflammatory properties [29,30,31,32,33]. Specifically, previous studies have shown that dietary intake of equol, a functional metabolite from *Bifidobacterium* spp., alleviates DSS-induced colitis in mice and increases the ratio of regulated T cells in the colon [44]. In the present study, it was of interest to discover that the intestinal abundances of these metabolites were significantly increased in response to *B. salyersiae* treatment. Our results suggest that these metabolites might be able to mediate the anti-colitis effect of *B. salyersiae*. However, more studies are needed to test this hypothesis.

Our study has some limitations. First, although we observed that the oral intake of *B. salyersiae* significantly changed the gut microbiota profiles of DSS-fed mice, currently, we do not know whether this was a cause or a consequence of the attenuated inflammatory response in the colon. More detailed studies are therefore needed to fully characterize the mechanism underlying the modulatory effect of *B. salyersiae* on gut microbiota. 

Second, for a microorganism to be considered a probiotic bacterium, it must meet some requirements, including resistance to different adverse conditions in the gut, tolerance to the acidic pH and bile salts, and safety (absence of virulence and acquired resistance genes), among others. However, in the present study, we did not explored these issues. Our current results indicate that *B. salyersiae* is a candidate probiotic species with potential anti-colitis properties. These questions could be the topic of future studies. 

Third, in our study we found that there was a predominance of *Bacteroides* spp., especially *Bacteroides acidifaciens*, in the MD group as compared to the BS group. This is a surprising and unexpected result because, theoretically, there would be a predominance of *Bacteroides* spp. in the BS group since we directly gave live *B. salyersiae* to these mice. Part of the reason might be that *B. salyersiae* was not able to colonize the gut of UC mice at the dosage given in the present study. However, future studies are needed to verify this possibility.

Fourth, only one *B. salyersiae* strain was included in our study and, therefore, we do not know whether other *B. salyersiae* strains would also have the same anti-colitis effects. It is possible that the anti-colitis effect of *B. salyersiae* is confined to this specific genus, but more systematic investigations are needed to further explore this issue. 

Fifth, due to the experimental design, we did not check the effect of *B. salyersiae* on the composition of the gut microbiota and the profile of the bacterial metabolites in healthy mice. It is possible that *B. salyersiae* could modulate the gut microbiota and its metabolites independent of UC. Additionally, in the present study, although we clearly show that *B. salyersiae* CSP6 was protective against DSS-induced colitis in mice, the changes in the inflammatory cytokines and oxidative stress markers in the colon were not investigated. These questions could be the subject of follow-up work in the future.

## 5. Conclusions

In conclusion, oral administration of *B. salyersiae* CSP6 could protect against DSS-induced colitis in mice. Specifically, *B. salyersiae* CSP6 improves body weight loss, colon contraction, intestinal bleeding, and mucosal damage in diseased mice. Moreover, *B. salyersiae* CSP6 attenuates gut dysbiosis in DSS-fed mice by decreasing the population of pathogenic *Escherichia-Shigella* spp. and increasing the abundance of probiotic *Dubosiella* spp. and *B. pseudolongum*. Additionally, by reshaping the colonic microbiota, *B. salyersiae* CSP6 increases the fecal concentrations of anti-inflammatory equol, 8-deoxylactucin, and tiglic acid. Altogether, *B. salyersiae* is a candidate probiotic species with potential anti-colitis properties in the human colon. 

## Data Availability

The data are available on request from the corresponding authors.

## Supplementary Materials

The following supporting information can be downloaded at https://www.mdpi.com/article/10.3390/nu16172918/s1, Figure S1: Wilcoxon rank-sum test analysis of the gut microbiota at the species level. The numbers represent adjusted *p* values for the bacteria; Figure S2: Butterfly plot of the representative metabolites in the MD group vs. the NC group. The numbers represent adjusted *p* values for the metabolites; Figure S3: Heatmap of the correlation between different metabolites and disease parameters. * *p* < 0.05; ** *p* < 0.01; *** *p* < 0.001; Table S1: Summary of the up-regulated and down-regulated metabolites in the MD group vs. the NC group; Table S2: Summary of the up-regulated and down-regulated metabolites in the BS group vs. the MD group.
