# Supplementary material for: Bacteroides salyersiae Is a Candidate Probiotic Species with Potential Anti-Colitis Properties in the Human Colon: First Evidence from an In Vivo Mouse Model
Source: Nutrients. 2024 Sep 1;16(17):2918. doi: 10.3390/nu16172918 (PMC11397318; doi:10.3390/nu16172918)
Supplement: Supplementary file 1 [file nutrients-16-02918-s001.zip › nutrients-3145420-supplementary.pdf]

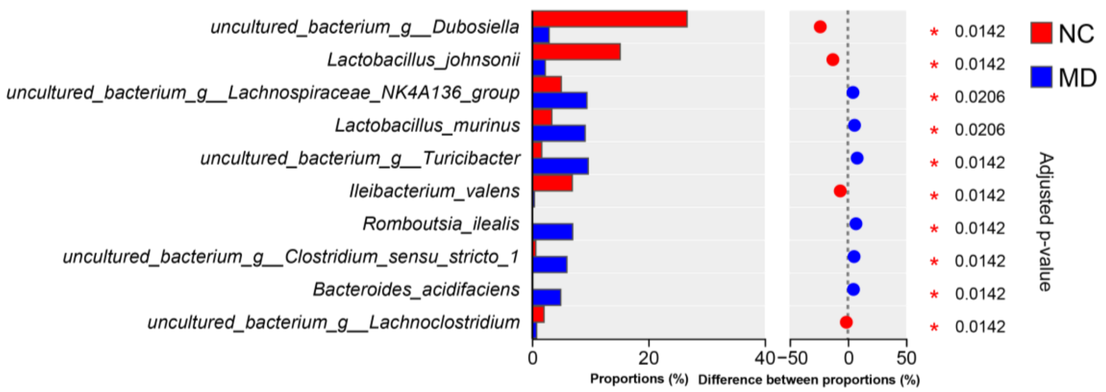


Figure S1. Wilcoxon rank-sum test analysis of the gut microbiota at the species level. The numbers represent adjusted *p* values for the bacteria.

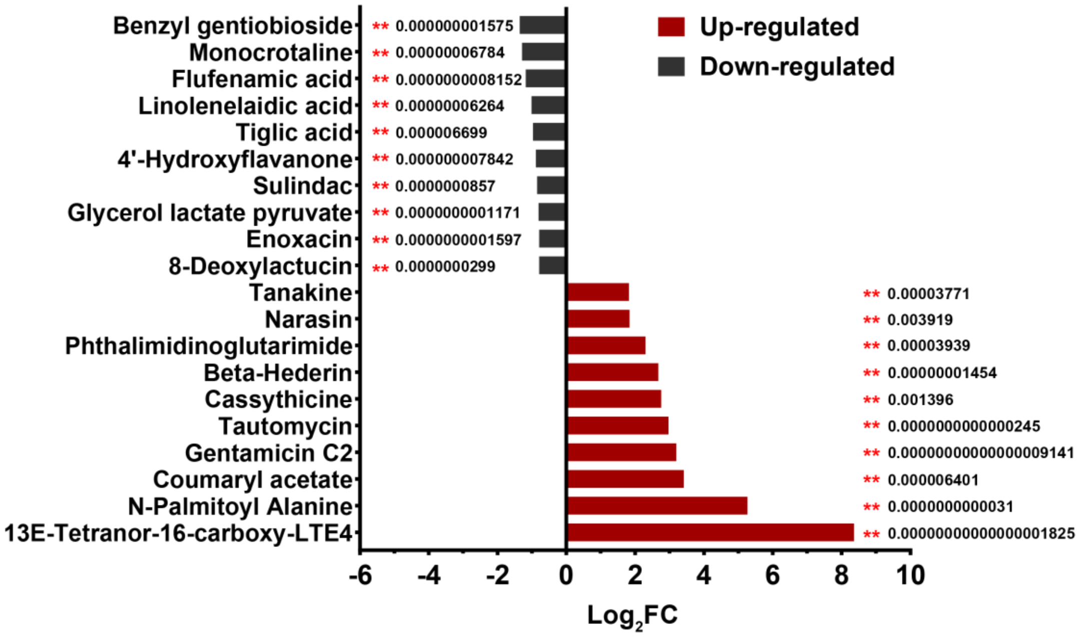


Figure S2. Butterfly plot of the representative metabolites in MD group vs NC group. The numbers represent adjusted *p* values for the metabolites.

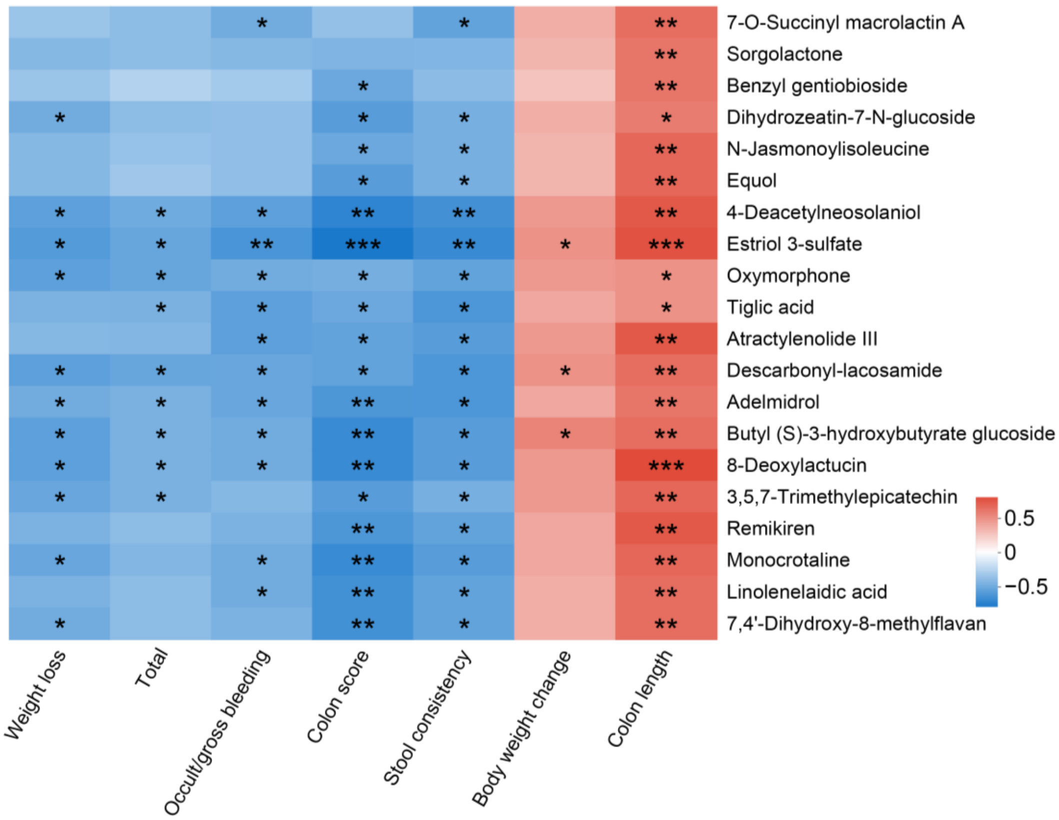


Figure S3. Heatmap of the correlation between different metabolites and disease parameters. * $p < 0.05$; ** $p < 0.01$; *** $p < 0.001$.

## Table S1 Summary of the up-regulated and down-regulated metabolites in MD group vs NC group

| Metabolite | Adjusted P value | FDR | Log$_2$FC(MD/NC) | m/z | Class |
|---|---|---|---|---|---|
| 13E-Tetranor-16-carboxy-LTE4 | 1.83E-17 | 4.17E-14 | 8.3636 | 394.132 | Carboxylic acids and derivatives |
| Tautomycin | 2.45E-14 | 3.16E-12 | 2.9707 | 811.4466 | Fatty Acyls |
| N-Palmitoyl Alanine | 3.10E-12 | 1.60E-10 | 5.2675 | 416.2497 | |
| Coumaryl acetate | 6.40E-06 | 1.66E-05 | 3.4132 | 237.0761 | Benzene and substituted derivatives |
| Gentamicin C2 | 9.14E-17 | 6.36E-14 | 3.1993 | 484.2763 | Organooxygen compounds |
| Ser Leu Ser Gly Leu | 3.22E-14 | 3.86E-12 | 2.1295 | 474.2557 | |
| Cis-and trans-L-Mercapto-p-menthan-3-one | 1.96E-07 | 8.21E-07 | 2.7875 | 417.2166 | |
| Val Leu Thr Gly Ser | 5.12E-09 | 4.73E-08 | 2.4145 | 476.2712 | |
| Beta-Hederin | 1.45E-08 | 1.09E-07 | 2.6733 | 379.2318 | Prenol lipids |
| Benzeneacetonitrile alpha-(3-((2-(3,4-dimethoxyphenyl)ethyl)amino)propyl)-4-hydroxy-3-methoxy-alpha-(1-methylethyl)- | 1.25E-08 | 9.64E-08 | 2.0827 | 444.2815 | Benzene and substituted derivatives |
| PE(22:6(4Z,7Z,11E,13Z,15E,19Z)-2OH(10S,17)/16:1(9Z)) | 2.60E-06 | 7.86E-06 | 2.5484 | 408.7447 | |
| PS(22:6(5Z,8E,10Z,13Z,15E,19Z)-2OH(7S, 17S)/20:4(8Z,11Z,14Z,17Z)) | 1.53E-07 | 7.35E-07 | 1.7315 | 444.7504 | |
| Ethyl glucoside | 2.85E-16 | 1.67E-13 | 1.742 | 461.1875 | Organooxygen compounds |
| N-[[3-(b-D-Glucopyranosyloxy)-2,3-dihydro-2-oxo-1H-indol-3-yl]acetyl]aspartic acid | 1.32E-05 | 3.14E-05 | 1.2021 | 483.1244 | Carboxylic acids and derivatives |
| Geneticin | 1.13E-11 | 3.96E-10 | 1.6831 | 535.2358 | Organooxygen compounds |
| Benzyl gentiobioside | 1.58E-09 | 1.64E-08 | -1.3533 | 477.1601 | Organooxygen compounds |
| Implitapide | 6.43E-06 | 1.67E-05 | 1.4816 | 530.2821 | Indoles and derivatives |
| Phthalimidinoglutarimide | 3.94E-05 | 8.33E-05 | 2.2985 | 289.0826 | Isoindoles and derivatives |
| Soyasaponin IV | 7.19E-07 | 2.70E-06 | 1.1858 | 767.4575 | Prenol lipids |
| N-[1'-(6-Cyano-1,2,3,4-tetrahydronaphthalen-2-yl)-4-hydroxyspiro[3,4-dihydrochromene-2,4'-piperidine]-6-yl]methanesulfonamide | 4.49E-16 | 2.20E-13 | 1.1637 | 488.1583 | Tetralins |
| Morphine 3-Sulfate | 2.75E-05 | 6.03E-05 | 1.7909 | 364.0854 | Morphinans |
| PKG Inhibitor | 5.70E-08 | 3.26E-07 | 1.3977 | 472.3016 | Carboxylic acids and derivatives |
| (2S,4S,6S)-2-[2-(4-Hydroxy-3-meyhoxyphenyl)ethyl]tetrahydro-6-(4,5-dihydroxy-3-methoxyphenyl)-2H-pyran-4-ol | 3.47E-05 | 7.48E-05 | 1.8669 | 803.3318 | Diarylheptanoids |
| Tanakine | 3.77E-05 | 8.02E-05 | 1.8183 | 264.1236 | Indoles and derivatives |
| Betamethasone 17 Valerate | 2.32E-08 | 1.41E-07 | 1.3198 | 457.2409 | Steroids and steroid derivatives |
| N-Oleoyl Aspartic acid | 5.10E-08 | 2.66E-07 | 1.3512 | 396.2743 | |
| 16-(Acetyloxy)-3,14-dihydroxycard-20(22)-enolide | 2.98E-05 | 6.51E-05 | 2.1623 | 477.2202 | Steroids and steroid derivatives |
| Deferitrin | 9.36E-06 | 2.38E-05 | 1.4459 | 254.0481 | Carboxylic acids and derivatives |
| Glucosyl passiflorate | 7.57E-12 | 2.48E-10 | 1.0506 | 731.3844 | Prenol lipids |
| N-((Hexahydro-1-azepinyl)carbonyl)-leucyl(1-methyl)-tryptophyl-tryptophan | 1.30E-09 | 1.40E-08 | 1.3244 | 600.3233 | Carboxylic acids and derivatives |
| 1-Nonadecanoyl-glycero-3-phosphoserine | 9.21E-17 | 6.36E-14 | 1.0911 | 574.2907 | Glycerophospholipids |
| Epothilone D | 2.19E-07 | 9.91E-07 | 1.4791 | 536.2384 | Macrolides and analogues |
| Sisomicin sulfate | 2.90E-12 | 1.53E-10 | 1.6107 | 430.2658 | |
| CDP-DG(i-22:0/20:5(5Z,8Z,11Z,14Z,16E)-OH(18R)) | 0.0001 | 0.000194 | 1.7768 | 550.8064 | |
| Verdamicin | 2.45E-12 | 1.37E-10 | 1.2726 | 444.2812 | Organooxygen compounds |
| Flufenamic acid | 8.15E-10 | 9.81E-09 | -1.1805 | 607.1268 | Benzene and substituted derivatives |
| Soyasaponin A1 | 2.72E-08 | 1.61E-07 | 0.9031 | 633.2935 | Prenol lipids |
| Dibekacin | 5.91E-06 | 1.60E-05 | 1.3508 | 416.2497 | Organooxygen compounds |
| Fenofibric acid | 1.04E-09 | 1.18E-08 | 0.9443 | 353.033 | Benzene and substituted derivatives |

| | | | | | |
|---|---|---|---|---|---|
| Pro Leu Ile | 3.59E-14 | 6.25E-12 | 1.3768 | 342.2385 | |
| CDP-DG(18:3(10,12,15)-OH(9)/i-22:0) | 7.33E-11 | 1.64E-09 | 1.203 | 560.7797 | |
| Phaseic acid | 3.69E-20 | 2.17E-16 | 1.0328 | 317.0772 | Prenol lipids |
| Sulfoglycolithocholate(2-) | 9.53E-11 | 1.76E-09 | 1.0716 | 558.2765 | Steroids and steroid derivatives |
| Simulansine | 2.69E-06 | 7.77E-06 | 1.2626 | 699.3585 | Quinolines and derivatives |
| Ponasterone A | 4.43E-11 | 9.69E-10 | 1.1272 | 501.2667 | Steroids and steroid derivatives |
| L-Cystine | 5.11E-07 | 1.85E-06 | 1.1374 | 239.0159 | Carboxylic acids and derivatives |
| Alpha-Bungarotoxin | 8.52E-09 | 7.14E-08 | 1.1525 | 459.2398 | Brevetoxins and derivatives |
| Soyasaponin II | 6.46E-13 | 3.93E-11 | 0.8117 | 957.5041 | Prenol lipids |
| DG(TXB2/0:0/12:0) | 9.17E-05 | 0.00018 | 1.4362 | 336.2077 | |
| Strictosidine | 9.23E-10 | 1.08E-08 | 1.0976 | 575.2208 | Prenol lipids |
| N,n-Diethyl-2,4-Dimethoxybenzamide | 0.000967 | 0.001532 | 2.5856 | 270.17 | |
| (5E,8E,11E)-13-[(2S,3S)-3-Pentylthiiran-2-yl]trideca-5,8,11-trienoic acid | 0.000134 | 0.000252 | 1.4928 | 350.2241 | Fatty Acyls |
| Ile Leu Leu Gly Ala | 9.47E-13 | 7.00E-11 | 1.1923 | 486.3284 | |
| 3,4,5,6-Tetrahydrohippuric acid | 6.99E-15 | 1.32E-12 | 0.9604 | 548.2565 | Carboxylic acids and derivatives |
| PIP(18:2(9Z,11E)+=O(13)/18:1(15Z)) | 4.96E-13 | 4.44E-11 | 1.0267 | 478.246 | |
| Monocrotaline | 6.78E-08 | 3.77E-07 | -1.2877 | 325.1545 | Pyrrolizines |
| CL(8:0/8:0/i-14:0/18:2(9Z,11Z)) | 7.85E-08 | 4.24E-07 | 0.9987 | 585.3294 | Glycerophospholipids |
| (4Z,8Z,10Z,13Z,16Z,19Z)-7-Hydroxydocosa-4,8,10,13,16,19-hexaenoylcarnitine | 5.05E-13 | 4.48E-11 | 1.0191 | 532.2975 | Fatty Acyls |
| PE(18:2(9Z,11E)+=O(13)/14:1(9Z)) | 1.10E-11 | 3.89E-10 | 1.0454 | 350.7335 | |
| Degammae | 4.87E-10 | 7.08E-09 | 0.836 | 663.3507 | Carboxylic acids and derivatives |
| PG(i-14:0/22:6(5Z,8E,10Z,13Z,15E,19Z)-2OH(7S, 17S)) | 9.90E-10 | 1.23E-08 | 1.0084 | 411.2293 | |
| Tert-butyl (2-{7-[(2S)-3-(4-cyanophenoxy)-2-hydroxypropyl]-9-oxa-3,7-diazabicyclo[3.3.1]non-3-yl}ethyl)carbamate | 5.72E-12 | 2.02E-10 | 1.1226 | 491.2501 | Phenol ethers |
| Neomacrostemonoside D | 5.41E-09 | 4.38E-08 | 0.8189 | 552.2669 | Steroids and steroid derivatives |
| Gitoxin | 3.59E-10 | 5.59E-09 | 1.157 | 844.4407 | Steroids and steroid derivatives |
| 5-Hydroxyindoleacetaldehyde | 2.55E-06 | 7.41E-06 | 1.1033 | 174.055 | Indoles and derivatives |
| (E)-2-Methyl-2-buten-1-ol O-beta-D-Glucopyranoside | 8.21E-08 | 3.90E-07 | 1.0237 | 293.1232 | Fatty Acyls |
| DG(10:0/0:0/20:4(8Z,11Z,14Z,17Z)-2OH(5S,6R)) | 1.58E-08 | 1.16E-07 | 1.295 | 294.1977 | |
| Soyasapogenol B 3-O-b-D-glucuronide | 5.40E-05 | 0.000111 | 0.998 | 657.3968 | Prenol lipids |
| PA(14:1(9Z)/20:5(5Z,8Z,11Z,14Z,16E)-OH(18R)) | 5.41E-07 | 1.95E-06 | 0.9526 | 701.3736 | |
| CDP-DG(18:2(10E,12Z)+=O(9)/i-22:0) | 8.30E-08 | 4.43E-07 | 1.199 | 538.7977 | |
| Sulfolithocholylglycine | 2.99E-11 | 7.08E-10 | 0.8155 | 534.2554 | Steroids and steroid derivatives |
| Netilmicin | 3.12E-13 | 3.11E-11 | 1.0578 | 458.2968 | Organooxygen compounds |
| Sampatrilat | 9.08E-08 | 4.26E-07 | 0.9352 | 605.2234 | Peptidomimetics |
| Arvensoside D | 1.08E-08 | 7.53E-08 | 0.6557 | 941.5095 | Prenol lipids |
| Folinic acid | 1.25E-10 | 2.19E-09 | 0.8527 | 472.1573 | Pteridines and derivatives |
| CDP-DG(20:2(11Z,14Z)/6 keto-PGF1alpha) | 8.20E-09 | 6.06E-08 | 0.8821 | 558.7637 | |
| Leu Ala Gly Glu Phe | 1.79E-14 | 3.65E-12 | 0.8552 | 536.271 | |
| Leucylproline | 4.73E-08 | 2.51E-07 | 0.8459 | 227.1394 | Carboxylic acids and derivatives |
| Asn Thr Val | 2.01E-08 | 1.41E-07 | 1.0785 | 333.1767 | |
| Thalidomide | 7.36E-15 | 1.37E-12 | 0.6421 | 303.0617 | Isoindoles and derivatives |
| Linalool oxide D 3-[apiosyl-(1->6)-glucoside] | 1.72E-08 | 1.10E-07 | 0.9444 | 509.2238 | Organooxygen compounds |
| (R)-Menthone 8-thioacetate | 1.62E-08 | 1.05E-07 | 0.8996 | 249.0948 | Prenol lipids |

| | | | | | |
|---|---|---|---|---|---|
| 2-(2-Hydroxyethyl)phenyl hydrogen sulfate | 1.04E-08 | 7.33E-08 | 0.7351 | 217.0169 | Organic sulfuric acids and derivatives |
| PS(20:4(8Z,11Z,14Z,17Z)-2OH(5S,6R)/20:2(11Z,14Z)) | 2.70E-09 | 2.83E-08 | 0.9746 | 445.7581 | |
| Perulactone B | 5.09E-10 | 6.68E-09 | 0.9489 | 533.2715 | Steroids and steroid derivatives |
| 11-beta-Hydroxyandrosterone-3-glucuronide | 1.63E-07 | 7.01E-07 | 0.8855 | 527.2457 | Indoles and derivatives |
| Midecamycin acetate | 6.39E-10 | 8.88E-09 | 0.8589 | 460.7345 | Organooxygen compounds |
| Methotrexate | 2.99E-06 | 8.89E-06 | 1.1378 | 496.2036 | Triazines |
| Valyl-Gamma-glutamate | 2.27E-11 | 6.90E-10 | 0.7459 | 554.2934 | Carboxylic acids and derivatives |
| PS(20:5(5Z,8Z,11Z,14Z,16E)-OH(18R)/22:5(4Z,7Z,10Z,13Z,16Z)) | 7.41E-08 | 4.05E-07 | 0.8224 | 447.75 | |
| LysoPA(22:5(7Z,10Z,13Z,16Z,19Z)/0:0) | 3.71E-08 | 2.07E-07 | 0.944 | 529.2614 | Glycerophospholipids |
| Nummularine A | 5.84E-08 | 3.33E-07 | 1.1773 | 324.6892 | Carboxylic acids and derivatives |
| Cassythicine | 0.001396 | 0.002139 | 2.7598 | 348.1228 | Aporphines |
| PGP(i-12:0/22:6(5Z,8E,10Z,13Z,15E,19Z)-2OH(7S, 17S)) | 7.77E-07 | 2.87E-06 | 0.9064 | 426.2106 | |
| 2-Feruloyl-1-sinapoylgentiobiose | 3.13E-09 | 2.83E-08 | 0.7296 | 723.2184 | Cinnamic acids and derivatives |
| 2-Hydroxyclomipramine | 2.29E-11 | 5.73E-10 | 0.8589 | 367.0959 | Benzazepines |
| 6-Lactoyltetrahydropterin | 1.46E-14 | 2.17E-12 | 0.7963 | 716.2915 | Pteridines and derivatives |
| Alliospiroside D | 2.15E-08 | 1.49E-07 | 1.0614 | 389.2084 | Organooxygen compounds |
| Naringenin | 4.35E-07 | 1.62E-06 | 0.8137 | 271.0608 | Flavonoids |
| Bz-Ile-glu-gly-arg-pna | 6.11E-13 | 3.77E-11 | 0.811 | 732.2865 | Carboxylic acids and derivatives |
| Pisumionoside | 6.63E-06 | 1.71E-05 | 0.9245 | 449.2031 | Prenol lipids |
| Mpomeovt | 3.03E-09 | 3.09E-08 | 0.9054 | 538.2527 | Carboxylic acids and derivatives |
| 6S,9R-Dihydroxy-4,7E-megastigmadien-3-one 9-[apiosyl-(1->6)-glucoside] | 7.51E-09 | 5.66E-08 | 0.8037 | 517.2277 | Fatty Acyls |
| Isovitexin 2''-O-(6'''-feruloyl)glucoside | 2.01E-12 | 9.31E-11 | 0.7115 | 769.197 | Flavonoids |
| Aldosterone | 1.78E-08 | 1.13E-07 | 0.9093 | 395.1617 | Steroids and steroid derivatives |
| Linolenelaidic acid | 6.26E-08 | 3.53E-07 | -1.0101 | 323.1964 | Fatty Acyls |
| 4'-Hydroxyflavanone | 7.84E-09 | 6.70E-08 | -0.8876 | 241.0858 | Flavonoids |
| (1R,2S,5S,6R,9S,10S)-3,6,17,20-Tetrahydroxy-6-(2-hydroxyacetyl)-1,5-dimethylpentacyclo[11.8.0.02,10.05,9.016,21]henicosa-13,16,18,20-tetraen-15-one | 2.79E-09 | 2.58E-08 | 0.8227 | 479.1481 | Steroids and steroid derivatives |
| Palustrine | 1.83E-09 | 2.02E-08 | 0.942 | 354.2133 | Macrolactams |
| Corynanthine | 6.35E-09 | 4.94E-08 | 0.8316 | 389.167 | Steroids and steroid derivatives |
| Tamoxifen | 1.61E-05 | 3.81E-05 | 1.2951 | 394.2122 | Stilbenes |
| Cholestane-3,7,12,25-tetrol-3-glucuronide | 2.25E-07 | 9.21E-07 | 0.8928 | 649.3301 | Steroids and steroid derivatives |
| PI(5-iso PGF2VI/18:2(9Z,12Z)) | 8.72E-07 | 3.15E-06 | 1.0937 | 475.2403 | |
| Pro Trp | 3.03E-09 | 3.09E-08 | 0.9026 | 302.1499 | |
| Dihydrozeatin-7-N-glucoside | 3.24E-10 | 5.25E-09 | -0.7772 | 425.2126 | Organooxygen compounds |
| Cis-4-Hydroxy-D-proline | 9.53E-08 | 4.98E-07 | 1.0701 | 304.15 | Carboxylic acids and derivatives |
| Cilastatin | 4.27E-13 | 2.88E-11 | 0.8264 | 403.1532 | Carboxylic acids and derivatives |
| PA(22:2(13Z,16Z)/6 keto-PGF1alpha) | 1.52E-07 | 7.33E-07 | 1.0049 | 433.2659 | |
| Stercobilinogen | 2.89E-07 | 1.24E-06 | 0.9772 | 321.1697 | Tetrapyrroles and derivatives |
| Verbascose | 8.24E-09 | 6.07E-08 | 0.6825 | 809.2553 | Organooxygen compounds |
| Frangulanine | 2.70E-13 | 2.87E-11 | 0.913 | 545.3092 | Carboxylic acids and derivatives |
| PS(20:3(8Z,11Z,14Z)/18:4(6Z,9Z,12Z,15Z)) | 1.11E-08 | 8.78E-08 | 0.8711 | 425.7367 | Glycerophospholipids |
| 17-AAG | 5.95E-08 | 3.01E-07 | 0.8129 | 630.3089 | Macrolactams |
| Sesamolinol 4'-O-b-D-glucosyl (1->6)-O-b-D-glucoside | 1.13E-11 | 3.32E-10 | 0.712 | 677.2128 | Organooxygen compounds |

| | | | | | |
|---|---|---|---|---|---|
| Gln Val Phe | 3.21E-12 | 1.61E-10 | 0.9145 | 393.2129 | |
| Tiglic acid | 6.70E-06 | 1.73E-05 | -0.9689 | 299.1495 | Fatty Acyls |
| N-Eicosapentaenoyl Leucine | 2.31E-09 | 2.46E-08 | 1.0738 | 460.2762 | |
| Anhydroerythromycin | 5.89E-11 | 1.38E-09 | 0.7078 | 754.4093 | Organooxygen compounds |
| PC(18:1(6Z)/0:0) | 1.60E-08 | 1.04E-07 | 0.792 | 542.3184 | Glycerophospholipids |
| 2-Chloro-1-[(2S,3S,5S,10S,13S)-3-hydroxy-10,13-dimethyl-2-morpholino-2,3,4,5,6,7,8,9,11,12,14,15,16,17-tetradecahydro-1H-cyclopenta[a]phenanthren-17-yl]ethanone | 7.20E-10 | 8.87E-09 | 0.8006 | 474.2192 | Steroids and steroid derivatives |
| 4-Hydroxydodecanedioylcarnitine | 2.52E-11 | 7.27E-10 | 0.7947 | 389.2393 | Fatty Acyls |
| Stevioside | 5.89E-11 | 1.21E-09 | -0.6246 | 841.3193 | Prenol lipids |
| 4,4'-Methylenebis(2,6-dimethylphenol) | 1.45E-10 | 2.46E-09 | 0.7595 | 557.2927 | Benzene and substituted derivatives |
| Bisoprolol | 1.25E-09 | 1.49E-08 | 0.79 | 389.2393 | Benzene and substituted derivatives |
| PE(20:3/0:0) | 0.000205 | 0.000372 | 0.9477 | 502.2924 | |
| Caroverine | 9.91E-09 | 8.07E-08 | 1.0453 | 379.2197 | Diazanaphthalenes |
| Hordatine A | 1.11E-05 | 2.76E-05 | 0.9153 | 531.277 | 2 arylbenzofuran flavonoids |
| 7,8-Dihydrovomifoliol 9-[rhamnosyl-(1->6)-glucoside] | 1.89E-16 | 1.54E-13 | 0.7647 | 576.3061 | Fatty Acyls |
| Dihydroergocristine | 8.87E-09 | 6.44E-08 | 0.7981 | 632.2885 | Ergoline and derivatives |
| Didemnin C | 0.001075 | 0.001686 | 1.6811 | 508.2978 | Peptidomimetics |
| Lucuminic acid | 2.49E-17 | 4.17E-14 | 0.7117 | 445.1341 | Organooxygen compounds |
| Dynorphin A (6-8) | 1.34E-06 | 4.54E-06 | 0.8619 | 488.2714 | Carboxylic acids and derivatives |
| PA(14:0/22:6(4Z,7Z,10Z,13Z,16Z,19Z)) | 1.66E-07 | 7.89E-07 | 0.9084 | 369.213 | Glycerophospholipids |
| 1,3-Dithiane-2-propanamine, N-(2-(3,4-dimethoxyphenyl)ethyl)-N-methyl-2-(2-naphthalenyl)- | 7.28E-12 | 2.40E-10 | 0.7865 | 516.1795 | Naphthalenes |
| Apelin-12 (human, bovine, mouse, rat) | 2.92E-09 | 3.00E-08 | 0.7045 | 722.8876 | Carboxylic acids and derivatives |
| 5-Hydroxycapsanthin | 4.15E-13 | 2.82E-11 | 0.7421 | 585.3968 | Prenol lipids |
| 8-Deoxylactucin | 2.99E-08 | 1.72E-07 | -0.7876 | 281.0812 | Lactones |
| Sulindac | 8.57E-08 | 4.05E-07 | -0.8523 | 401.09 | Indenes and isoindenes |
| 6-Thioinosine-5'-monophosphate | 4.05E-07 | 1.53E-06 | 0.6069 | 363.0174 | Purine nucleotides |
| Microcystin RR | 2.34E-07 | 1.05E-06 | 0.8693 | 530.7762 | Peptidomimetics |
| Propranolol | 9.80E-06 | 2.47E-05 | 1.0883 | 323.1751 | Naphthalenes |
| Threonylproline | 1.77E-09 | 1.80E-08 | 0.8127 | 431.2132 | Carboxylic acids and derivatives |
| 5''-(4-Hydroxy-(E)-cinnamoyl) alpha-L-arabinofuranosyl-(1->3)-beta-D-xylopyranosyl-(1->4)-D-xylopyranoside | 4.62E-12 | 1.77E-10 | 0.6273 | 581.1474 | Organooxygen compounds |
| CDP-DG(18:0/20:5(5Z,8Z,11Z,14Z,16E)-OH(18R)) | 2.36E-13 | 2.59E-11 | 0.8241 | 533.7617 | |
| Janthitrem C | 2.02E-06 | 6.37E-06 | 0.9195 | 285.684 | Peptidomimetics |
| Avapritinib | 1.79E-08 | 1.29E-07 | 0.8213 | 531.2767 | Diazinanes |
| Tremetone | 3.11E-05 | 6.78E-05 | 1.1288 | 220.1331 | Benzene and substituted derivatives |
| (-)-Arctigenin | 4.81E-11 | 1.03E-09 | 0.709 | 803.3238 | Furanoid lignans |
| Bisphenol B | 5.07E-10 | 6.67E-09 | 0.7479 | 529.2613 | Benzene and substituted derivatives |
| Trans-Zeatin-O-glucoside riboside | 4.20E-14 | 4.74E-12 | 0.7668 | 494.1882 | Naphthalenes |
| 2-Anthraquinonesulfonic acid | 7.62E-05 | 0.000151 | 0.6612 | 333.0068 | Anthracenes |
| Nelfinavir | 2.28E-07 | 9.33E-07 | 0.7524 | 588.287 | Carboxylic acids and derivatives |
| PS(20:2(11Z,14Z)/20:3(8Z,11Z,14Z)-2OH(5,6)) | 9.47E-06 | 2.40E-05 | 0.9854 | 457.7559 | |
| Gibberellin A37 | 1.94E-11 | 6.08E-10 | 0.8453 | 379.211 | Prenol lipids |

| | | | | | |
|---|---|---|---|---|---|
| Sulindac sulfide | 2.15E-11 | 5.51E-10 | 0.7973 | 361.0705 | Indenes and isoindenes |
| Narasin | 0.003919 | 0.005532 | 1.8365 | 394.2549 | Prenol lipids |
| Enoxacin | 1.60E-10 | 3.02E-09 | -0.7926 | 343.1147 | Diazanaphthalenes |
| Dimethyl dimethoxy biphenyl | 8.08E-08 | 3.85E-07 | 0.7429 | 529.2615 | Benzene and substituted derivatives |
| Epothilone B | 3.02E-11 | 8.21E-10 | 0.8078 | 508.2761 | Macrolides and analogues |
| 1-[(3R,4R,5R)-3-Fluoro-3,4-dihydroxy-5-(1-hydroxyethyl)oxolan-2-yl]pyrimidine-2,4-dione | 1.78E-10 | 2.90E-09 | 0.7962 | 321.0724 | Organooxygen compounds |
| Moroxydine | 8.91E-13 | 5.10E-11 | 0.7654 | 387.2241 | Organonitrogen compounds |
| [(2R,5R)-5-(2-Amino-6-oxo-1H-purin-9-yl)-3,4-dihydroxyoxolan-2-yl]methyl [(2R,5R)-5-(4-amino-2-oxopyrimidin-1-yl)-3,4-dihydroxyoxolan-2-yl]methyl hydrogen phosphate | 3.72E-16 | 1.90E-13 | 0.6938 | 569.1135 | (3''->5'') dinucleotides |
| 3,5,7-Trimethylepicatechin | 1.14E-08 | 8.98E-08 | -0.7238 | 333.1331 | Flavonoids |
| Val Trp | 7.73E-07 | 2.65E-06 | 0.8388 | 302.1504 | |
| Isoline | 2.27E-06 | 6.69E-06 | 0.7554 | 849.3986 | |
| Lagerstroemine | 3.95E-08 | 2.42E-07 | 0.9497 | 479.2497 | Macrolides and analogues |
| Dukunolide E | 1.09E-07 | 4.97E-07 | 0.7568 | 519.1428 | Naphthopyrans |
| 3-Hydroxybenzyl alcohol glucoside | 2.08E-11 | 5.39E-10 | 0.6462 | 321.0722 | Organooxygen compounds |
| PA(PGF2alpha/19:0) | 4.43E-11 | 1.10E-09 | 0.779 | 406.2552 | |
| Nystatin | 4.21E-08 | 2.56E-07 | 0.8449 | 463.7635 | Organooxygen compounds |
| Toosendanin | 3.03E-11 | 7.14E-10 | 0.7 | 619.2353 | Prenol lipids |
| Glycerol lactate pyruvate | 1.17E-10 | 2.42E-09 | -0.8062 | 298.0895 | Glycerolipids |
| Cis-Ferulic acid 4-sulfate | 8.75E-10 | 1.04E-08 | 0.561 | 273.0069 | Cinnamic acids and derivatives |
| 3-hydroxy-3-(3-hydroxyphenyl)propanoic acid-O-sulphate | 3.88E-11 | 8.70E-10 | 0.6607 | 261.007 | Hydroxy acids and derivatives |
| Omadacycline | 1.24E-10 | 2.53E-09 | 0.7222 | 589.3191 | Tetracyclines |
| N-Nervonoyl Glutamine | 6.34E-10 | 8.83E-09 | -0.7295 | 536.442 | |
| Epiandrosterone | 4.93E-05 | 0.000102 | 0.8197 | 329.1858 | Steroids and steroid derivatives |
| PI(PGD2/18:1(9Z)) | 1.58E-05 | 3.75E-05 | 0.8576 | 489.2488 | |
| Biochanin A 7-(6-malonylglucoside) | 3.26E-10 | 5.26E-09 | 0.6243 | 565.155 | Isoflavonoids |
| O-Ureidohomoserine | 1.36E-09 | 1.45E-08 | 0.7274 | 530.2208 | Carboxylic acids and derivatives |
| Biocytin | 6.29E-08 | 3.54E-07 | 0.8906 | 405.2131 | Carboxylic acids and derivatives |
| Geosmin | 1.29E-09 | 1.39E-08 | -0.6918 | 181.1587 | Organooxygen compounds |
| Prunetin | 2.59E-07 | 1.04E-06 | 0.8466 | 283.0607 | Isoflavonoids |
| Asn Ile Ile | 1.52E-10 | 2.92E-09 | 0.7598 | 359.2287 | |
| Abeado | 9.76E-12 | 3.54E-10 | 0.743 | 363.2004 | 5''-deoxyribonucleosides |
| Cis-10-Hydroxylinalyl oxide 7-glucoside | 1.06E-08 | 7.41E-08 | 0.7545 | 393.1771 | Organooxygen compounds |
| Isofloxythepin | 8.70E-09 | 6.34E-08 | 0.725 | 399.1875 | Benzothiepins |
| Cyclosquamosin E | 2.72E-10 | 4.63E-09 | 0.7889 | 334.1607 | Carboxylic acids and derivatives |
| (3b,20R,22R)-3,20,27-Trihydroxy-1-oxowitha-5,24-dienolide 3-glucoside | 3.96E-13 | 3.79E-11 | 0.7349 | 657.3237 | Prenol lipids |
| PI(20:4(7E,9E,11Z,13E)-3OH(5S,6R,15S)/20:3(5Z,8Z,11Z)) | 4.64E-07 | 1.85E-06 | 0.7427 | 501.2561 | |
| N-Jasmonoylisoleucine | 2.22E-13 | 2.47E-11 | -0.7348 | 324.2168 | Carboxylic acids and derivatives |
| Idebenone | 2.07E-06 | 6.51E-06 | 0.8995 | 181.1027 | |
| Grepafloxacin | 7.69E-10 | 9.37E-09 | 0.6596 | 777.3413 | Quinolines and derivatives |
| PE(LTE4/20:2(11Z,14Z)) | 1.41E-11 | 4.69E-10 | 0.7424 | 464.2846 | |
| Pyridinoline | 0.00289 | 0.004181 | 1.1133 | 446.2242 | Carboxylic acids and derivatives |
| Apiin | 2.01E-10 | 3.18E-09 | 0.5176 | 563.1392 | Naphthalenes |

| | | | | | |
|---|---|---|---|---|---|
| 1-Octanesulfonic acid | 1.03E-08 | 7.27E-08 | 0.7219 | 447.2088 | Organic sulfonic acids and derivatives |
| Nivacortol | 2.10E-08 | 1.29E-07 | 0.698 | 467.1923 | Steroids and steroid derivatives |
| Angiotensin IV | 2.07E-07 | 9.48E-07 | 0.881 | 388.2135 | Carboxylic acids and derivatives |
| PG(i-14:0/20:3(8Z,11Z,14Z)-2OH(5,6)) | 4.14E-10 | 6.30E-09 | 0.7416 | 411.2291 | |
| PE(15:0/20:3(6,8,11)-OH(5)) | 1.42E-06 | 4.76E-06 | 0.6673 | 394.7423 | |
| Pro Leu Leu | 0.000153 | 0.000285 | 0.8703 | 342.2385 | |
| S-(11-hydroxy-9-deoxy-delta12-PGD2)-glutathione | 1.12E-09 | 1.36E-08 | 0.7641 | 323.6738 | Carboxylic acids and derivatives |
| Bedoradrine | 9.93E-14 | 9.52E-12 | 0.6448 | 449.203 | Tetralins |
| (R)-1-O-[b-D-Apiofuranosyl-(1->2)-b-D-glucopyranoside]-1,3-octanediol | 7.23E-09 | 6.27E-08 | 0.8007 | 459.2565 | Fatty Acyls |
| (S)-N-Methylcoclaurine | 0.000204 | 0.00037 | 0.8725 | 363.17 | Isoquinolines and derivatives |
| LysoPE(20:5(5Z,8Z,11Z,14Z,17Z)/0:0) | 6.45E-07 | 2.27E-06 | 0.7018 | 534.2406 | Glycerophospholipids |
| Histidinal | 3.56E-09 | 3.53E-08 | -0.7884 | 140.0819 | Organonitrogen compounds |
| N-Acetyl-DL-Phenylalanine | 4.51E-09 | 3.78E-08 | 0.7574 | 206.0814 | Carboxylic acids and derivatives |
| Cyclosquamosin G | 5.29E-12 | 2.32E-10 | 0.6641 | 797.4041 | Carboxylic acids and derivatives |
| Streptolydigin | 1.65E-06 | 5.07E-06 | 0.6972 | 645.3087 | Carboxylic acids and derivatives |
| Atractylenolide III | 4.36E-09 | 3.68E-08 | -0.5788 | 541.2824 | Prenol lipids |
| Tetradec-5-Ynoic Acid | 7.36E-08 | 3.58E-07 | -0.6074 | 223.1695 | |
| (6R)-Folinic acid | 2.23E-13 | 2.47E-11 | 0.7027 | 474.1729 | Pteridines and derivatives |
| Val Phe Tyr | 1.78E-05 | 4.14E-05 | 0.9153 | 428.2178 | |
| Betanin | 7.21E-08 | 3.52E-07 | 0.7258 | 571.1181 | Prenol lipids |
| Ixabepilone | 3.94E-11 | 1.01E-09 | 0.6567 | 520.2949 | Macrolides and analogues |
| Reserpine | 7.42E-11 | 1.44E-09 | 0.5981 | 589.2578 | Yohimbine alkaloids |
| Osmundalin | 7.21E-14 | 9.89E-12 | 0.772 | 354.115 | Organooxygen compounds |
| Momordin IIa | 2.53E-12 | 1.39E-10 | 0.7102 | 482.2481 | Prenol lipids |
| Indole-3-carboxilic acid-O-sulphate | 4.16E-07 | 1.56E-06 | 0.8065 | 239.9966 | Indoles and derivatives |
| Camphorsulfonic acid | 0.000168 | 0.00031 | 0.7217 | 232.0791 | Prenol lipids |
| Oxymorphone | 1.50E-11 | 4.90E-10 | -0.7668 | 319.1651 | Phenanthrenes and derivatives |
| Met-lys-bradykinin | 9.20E-10 | 1.17E-08 | 0.6893 | 671.3452 | Polypeptides |
| 2-Hydroxy-p-mentha-1,8-dien-6-one | 6.45E-14 | 9.25E-12 | -0.8239 | 208.1333 | Prenol lipids |
| Tributyrylglycerol | 2.88E-08 | 1.67E-07 | -0.5262 | 301.1651 | Fatty Acyls |
| Supinine | 2.47E-07 | 9.99E-07 | 0.6892 | 304.1507 | |
| Glycylprolylhydroxyproline | 5.31E-06 | 1.41E-05 | 0.6664 | 615.2616 | Carboxylic acids and derivatives |
| Hebevinoside I | 5.61E-07 | 2.01E-06 | 0.5912 | 829.4775 | Organooxygen compounds |
| CDP-DG(20:1(11Z)/18:1(12Z)-2OH(9,10)) | 1.60E-09 | 1.82E-08 | 0.65 | 555.7748 | |
| Ala Val Trp | 9.01E-07 | 3.24E-06 | 0.7878 | 375.2024 | |
| (2R,3R)-3-[[(2S)-1-[4-(Diaminomethylideneamino)butylamino]-4-methyl-1-oxopentan-2-yl]carbamoyl]oxirane-2-carboxylic acid | 2.14E-09 | 2.10E-08 | 0.7127 | 402.1984 | Carboxylic acids and derivatives |
| MG(PGJ2/0:0/0:0) | 1.29E-13 | 1.57E-11 | 0.7492 | 447.2104 | |
| Gly Ile His | 1.17E-11 | 4.05E-10 | 0.7201 | 326.182 | |
| (5E,7E)-Undeca-2,5,7-trienedioylcarnitine | 8.13E-09 | 6.02E-08 | 0.6776 | 374.1565 | Fatty Acyls |
| Bacoside A | 2.45E-08 | 1.48E-07 | 0.5362 | 767.457 | Prenol lipids |
| Pteroic acid | 9.80E-12 | 3.54E-10 | -0.7343 | 313.1042 | Pteridines and derivatives |
| Desfuroyl Ceftiofur | 2.48E-12 | 1.08E-10 | -0.6549 | 474.0242 | Lactams |

| | | | | | |
|---|---|---|---|---|---|
| Soyasaponin I | 3.01E-09 | 3.08E-08 | 0.5458 | 943.526 | Prenol lipids |
| Dihydroouabain | 7.67E-08 | 4.16E-07 | 0.6958 | 569.2927 | Steroids and steroid derivatives |
| LysoPE(0:0/24:6(6Z,9Z,12Z,15Z,18Z,21Z)) | 7.06E-06 | 1.81E-05 | 0.6388 | 588.2871 | Glycerophospholipids |
| DG(PGF1alpha/2:0/0:0) | 2.76E-08 | 1.82E-07 | 0.7659 | 473.3083 | |
| (5Z)-3-Hydroxytetradec-5-enedioylcarnitine | 7.38E-14 | 9.89E-12 | 0.7123 | 415.255 | Fatty Acyls |
| Cinncassiol E | 5.81E-13 | 3.63E-11 | 0.5827 | 795.3882 | Prenol lipids |
| Sulfamethoxazole | 9.09E-10 | 1.07E-08 | 0.6482 | 565.1184 | Benzene and substituted derivatives |
| Withaperuvin G | 6.95E-06 | 1.84E-05 | 0.7333 | 485.2555 | Steroids and steroid derivatives |
| MURABUTIDE | 1.46E-06 | 4.85E-06 | 0.6828 | 590.3031 | Carboxylic acids and derivatives |
| Aspartyl-Tryptophan | 1.64E-11 | 5.29E-10 | 0.7541 | 320.124 | Carboxylic acids and derivatives |
| PS(18:0/20:5(5Z,8Z,11Z,14Z,16E)-OH(18R)) | 7.00E-10 | 9.51E-09 | 0.7116 | 435.7499 | |
| 5-(4'-Hydroxyphenyl)-gamma-valerolactone 4'-sulfate | 0.000122 | 0.000232 | 0.9669 | 317.033 | Organic sulfuric acids and derivatives |
| PA(22:6(5Z,8E,10Z,13Z,15E,19Z)-2OH(7S, 17S)/22:5(4Z,7Z,10Z,13Z,16Z)) | 2.80E-13 | 2.91E-11 | 0.7281 | 425.239 | |
| Tebipenem | 7.69E-09 | 5.76E-08 | 0.705 | 404.0746 | Lactams |
| (+/-)-Tryptophan | 1.07E-07 | 5.51E-07 | 0.8334 | 409.1866 | Indoles and derivatives |
| His Val Ile | 1.12E-05 | 2.77E-05 | 0.7155 | 368.2292 | |
| PA(18:3(6Z,9Z,12Z)/PGJ2) | 5.81E-15 | 1.12E-12 | 0.5513 | 783.4004 | |
| Lacto-N-tetraose | 1.66E-09 | 1.70E-08 | -0.6246 | 688.2295 | Organooxygen compounds |
| Chrysosplenetin | 1.90E-15 | 5.06E-13 | -0.7122 | 395.0738 | Flavonoids |
| 6-Thioxanthylic acid | 3.58E-08 | 2.01E-07 | 0.7284 | 379.012 | Purine nucleosides |
| Cys-Arg-Glu-Lys-Ala | 5.72E-06 | 1.56E-05 | 0.7042 | 647.3243 | Carboxylic acids and derivatives |
| Asn Leu Glu Ala Ile | 2.84E-10 | 4.79E-09 | 0.6782 | 559.3084 | |
| Schaftoside | 8.11E-11 | 1.54E-09 | 0.4479 | 563.1391 | Flavonoids |
| 2,3-Dihydroflavon-3-ol | 4.53E-12 | 2.09E-10 | -0.7659 | 241.0859 | Flavonoids |
| Soyasaponin III | 1.71E-19 | 7.68E-16 | 0.4561 | 797.4679 | Prenol lipids |
| Ala Leu Leu Ala Ser | 7.35E-09 | 6.37E-08 | 0.6846 | 474.2921 | |
| Tyr Trp Thr | 5.75E-12 | 2.41E-10 | 0.695 | 469.208 | |
| 6\|A-Hydroxy-7\|A-(thiomethyl)spirolactone | 1.53E-08 | 1.01E-07 | 0.6506 | 449.2031 | Steroids and steroid derivatives |
| 5-Acetamidovalerate | 1.98E-09 | 2.16E-08 | 0.6489 | 360.2127 | Fatty Acyls |
| 4-Vinylphenol sulfate | 1.10E-05 | 2.68E-05 | 0.5803 | 245.0119 | Organic sulfuric acids and derivatives |
| Manoalide | 1.04E-07 | 5.40E-07 | -0.6588 | 461.2276 | Prenol lipids |
| 11-Dehydro-thromboxane B2 | 6.69E-10 | 9.17E-09 | 0.7168 | 369.2242 | Fatty Acyls |
| Isovitexin 2''-O-arabinoside | 1.07E-08 | 8.60E-08 | 0.514 | 565.1549 | Flavonoids |
| S-(2-Methylbutanoyl)-dihydrolipoamide | 3.33E-06 | 9.77E-06 | 0.684 | 274.1298 | Fatty Acyls |
| 6-O-Oleuropeoylsucrose | 3.27E-06 | 9.23E-06 | 0.6707 | 529.1926 | Saccharolipids |
| CDP-DG(PGF2alpha/i-21:0) | 5.92E-09 | 5.33E-08 | 0.6549 | 572.7958 | |
| Benazeprilat | 6.04E-07 | 2.32E-06 | 0.6847 | 397.1765 | Carboxylic acids and derivatives |
| (+/-)-Dulciol E | 3.41E-09 | 3.41E-08 | -0.6224 | 313.1069 | Benzopyrans |
| Adelmidrol | 9.32E-09 | 6.70E-08 | -0.689 | 295.1657 | Organonitrogen compounds |
| Beraprost | 1.74E-09 | 1.94E-08 | -0.6459 | 437.1763 | Fatty Acyls |
| (4beta,5beta,6beta,14beta,15alpha,20S,22R)-5,6-Epoxy-4,14,15-trihydroxy-1-oxowitha-2,24-dienolide | 0.000751 | 0.001216 | 1.0378 | 244.1369 | Steroids and steroid derivatives |

| | | | | | |
|---|---|---|---|---|---|
| Noralfentanil | 8.28E-11 | 1.82E-09 | 0.7682 | 294.2175 | Benzene and substituted derivatives |
| Glutamylarginine | 7.88E-07 | 2.90E-06 | 0.5997 | 304.1612 | Carboxylic acids and derivatives |
| Trichlormethine | 1.15E-05 | 2.77E-05 | 0.7373 | 248.0017 | Organonitrogen compounds |
| 7-O-Succinyl macrolactin A | 0.000967 | 0.001543 | 1.2123 | 537.226 | Macrolides and analogues |
| 4-Demethylsimmondsin 2'-(E)-ferulate | 2.90E-08 | 1.68E-07 | -0.5519 | 572.1545 | Cinnamic acids and derivatives |
| Norfloxacin | 2.28E-09 | 2.20E-08 | 0.7268 | 354.1049 | Carboxylic acids and derivatives |
| Phenylalanyl-prolyl-arginine-chloromethyl ketone | 1.37E-07 | 6.77E-07 | 0.6209 | 451.2187 | Carboxylic acids and derivatives |
| Imidaprilat | 0.00077 | 0.001249 | 0.7987 | 422.1562 | Carboxylic acids and derivatives |
| PI(20:3(8Z,11Z,14Z)-2OH(5,6)/20:0) | 8.74E-10 | 1.12E-08 | 0.6486 | 486.292 | |
| Ala Asp Phe Asp | 4.64E-12 | 1.77E-10 | 0.6468 | 465.1614 | |
| Asp Glu Leu Val Ile | 1.61E-05 | 3.80E-05 | 0.7876 | 588.3235 | |
| Picrocrocin | 2.15E-09 | 2.11E-08 | 0.6341 | 365.1353 | Organooxygen compounds |
| 3,6-Ditigloyloxytropan-7-ol | 4.98E-09 | 4.11E-08 | 0.6532 | 358.1612 | Tropane alkaloids |
| Gibberellin A24 | 2.73E-09 | 2.53E-08 | 0.6282 | 345.17 | Prenol lipids |
| (1S,2S,3S,4R)-3-(1-Acetamido-2-ethylbutyl)-4-(diaminomethylideneamino)-2-hydroxycyclopentane-1-carboxylic acid | 8.45E-09 | 6.18E-08 | 0.5039 | 373.2083 | Carboxylic acids and derivatives |
| Mibefradil | 3.52E-07 | 1.35E-06 | 0.6117 | 532.2359 | Tetralins |
| (R)-Higenamine | 0.001933 | 0.002883 | 1.1212 | 272.1281 | Isoquinolines and derivatives |
| (9S,10E,12Z)-9-hydroperoxy-10,12-octadecadienoate | 2.31E-12 | 1.32E-10 | 0.6271 | 356.1926 | Fatty Acyls |
| Aesculetin | 6.37E-05 | 0.000129 | 0.6723 | 357.0571 | Coumarins and derivatives |
| Gln Leu Ile | 4.61E-07 | 1.85E-06 | 0.7039 | 373.2443 | |
| 5',8-Dihydroxy-3',4',7-trimethoxyflavan | 2.62E-09 | 2.75E-08 | -0.5871 | 333.1331 | Flavonoids |
| Telocinobufagin | 2.46E-08 | 1.67E-07 | 0.7447 | 416.2501 | Steroids and steroid derivatives |
| CL(10:0/11:0/11:0/12:0) | 2.13E-07 | 9.70E-07 | 0.605 | 548.324 | Glycerophospholipids |
| 2'-O-Methyladenosine | 2.10E-06 | 6.58E-06 | 0.6618 | 281.1131 | Purine nucleosides |
| Valylproline | 2.44E-07 | 9.88E-07 | 0.6474 | 473.26 | Carboxylic acids and derivatives |
| Flavan-3-ol | 1.92E-10 | 3.53E-09 | -0.5829 | 475.1921 | Flavonoids |
| Glu Lys Leu | 8.05E-12 | 3.07E-10 | 0.6747 | 389.2393 | |
| Eliglustat | 2.00E-11 | 6.24E-10 | 0.6521 | 449.2391 | Benzodioxanes |
| Gentianine | 6.92E-05 | 0.000138 | 0.7432 | 174.055 | Pyranopyridines |
| Rhizopterin | 6.73E-07 | 2.35E-06 | -0.5713 | 339.0839 | |
| Phenylalanylisoleucine | 8.74E-05 | 0.000171 | 0.6694 | 601.319 | Carboxylic acids and derivatives |
| Dihydroferulic acid 4-O-sulfate | 6.72E-10 | 8.37E-09 | 0.4637 | 275.0226 | Organic sulfuric acids and derivatives |
| 3'-DEOXY-3'-FLUOROTHYMIDINE | 7.38E-05 | 0.000147 | 0.7477 | 243.0769 | Pyrimidine nucleosides |
| Araliasaponin II | 7.07E-06 | 1.81E-05 | 0.6534 | 471.2407 | Prenol lipids |
| Hippuric acid | 1.67E-08 | 1.08E-07 | 0.6729 | 224.0556 | Benzene and substituted derivatives |
| Eptifibatide | 6.92E-08 | 3.40E-07 | -0.5615 | 812.2928 | Carboxylic acids and derivatives |
| Gly Val Ala Asp Val | 4.18E-08 | 2.27E-07 | 0.634 | 458.2248 | |
| Ala Val Val Pro Leu | 2.84E-11 | 7.89E-10 | 0.6227 | 498.3281 | |
| Cholic acid glucuronide | 1.30E-06 | 4.43E-06 | 0.6161 | 598.3271 | Steroids and steroid derivatives |
| 3-Demethylsimmondsin 2'-(Z)-ferulate | 1.80E-07 | 7.64E-07 | -0.5251 | 572.1546 | Cinnamic acids and derivatives |
| 2,5-Dihydroxybenzenesulfonic Acid | 2.09E-09 | 2.06E-08 | 0.5674 | 188.9854 | Benzene and substituted derivatives |
| Brevetoxin B4a | 7.60E-07 | 2.81E-06 | 0.6256 | 622.8609 | Brevetoxins and derivatives |

| | | | | | |
|---|---|---|---|---|---|
| Glycylalanylprolylmethionylphenylalanylvalinamide | 2.32E-07 | 9.48E-07 | 0.528 | 600.2985 | Carboxylic acids and derivatives |
| Gly Phe Phe | 8.39E-11 | 1.84E-09 | 0.6668 | 370.176 | |
| Shoyuflavone B | 2.42E-06 | 7.09E-06 | -0.5771 | 437.0251 | Isoflavonoids |
| Dapt | 5.95E-08 | 3.02E-07 | 0.6154 | 431.1774 | Carboxylic acids and derivatives |
| Avenestergenin A2 | 3.51E-07 | 1.35E-06 | 0.5734 | 643.3368 | Prenol lipids |
| 3,4-Dihydroxyphenylacetaldehyde | 1.19E-09 | 1.31E-08 | 0.6496 | 363.108 | Benzene and substituted derivatives |
| Mozenavir | 7.71E-06 | 2.01E-05 | 0.7054 | 501.2666 | Benzene and substituted derivatives |
| Leu Glu Lys Glu | 3.05E-10 | 5.05E-09 | 0.6352 | 518.282 | |
| 16-feruloyloxypalmitate | 1.66E-05 | 3.91E-05 | 0.7073 | 489.3065 | Cinnamic acids and derivatives |
| PE(PGJ2/14:1(9Z)) | 3.43E-08 | 2.15E-07 | 0.625 | 370.7277 | |
| Arginyl-prolyl-proline | 3.48E-09 | 3.47E-08 | 0.6959 | 369.2243 | Carboxylic acids and derivatives |
| Ulimorelin | 0.000981 | 0.001552 | 0.8346 | 503.2821 | Carboxylic acids and derivatives |
| PE(22:4(7Z,10Z,13Z,16Z)/22:6(4Z,8Z,10Z,13Z,16Z,19Z)-OH(7)) | 6.08E-10 | 8.56E-09 | 0.6681 | 450.7628 | |
| Blasticidin S | 9.56E-08 | 4.46E-07 | 0.5987 | 403.1825 | Carboxylic acids and derivatives |
| PS(15:0/6 keto-PGF1alpha) | 4.24E-09 | 4.06E-08 | 0.6352 | 429.7374 | |
| AcOrn[Oic(2),(alphaMe)Phe(5),dbetaNal(7),Ile(8)]desArg(9)-bradykinin | 4.45E-11 | 1.11E-09 | 0.5917 | 619.824 | Carboxylic acids and derivatives |
| Glu-Ile-Leu-Asp-Val | 6.68E-07 | 2.34E-06 | 0.627 | 586.3079 | Peptidomimetics |
| N-Acetylmuramoyl-Ala | 4.21E-07 | 1.71E-06 | -0.7777 | 347.1451 | Organooxygen compounds |
| Norizalpinin | 6.67E-05 | 0.000134 | 0.7403 | 271.0599 | Flavonoids |
| PG(PGD2/20:3(5Z,8Z,11Z)) | 2.21E-10 | 3.90E-09 | 0.6575 | 435.2635 | |
| Trp Val Ile | 1.62E-09 | 1.83E-08 | 0.6438 | 417.2492 | |
| N-Lauroyl Tryptophan | 0.000276 | 0.000488 | 0.7706 | 431.2248 | Carboxylic acids and derivatives |
| 2-(4-Morpholinyl)-8-phenyl-4H-1-benzopyran-4-one | 6.23E-08 | 3.51E-07 | -0.6565 | 325.1542 | Benzopyrans |
| 2'',6''-O-Diacetyloninin | 2.02E-11 | 5.29E-10 | 0.4954 | 415.1826 | Pteridines and derivatives |
| Trilostane | 3.96E-06 | 1.13E-05 | 0.6819 | 393.2123 | Steroids and steroid derivatives |
| Nalmefene | 3.74E-08 | 2.08E-07 | 0.6139 | 374.1561 | Phenanthrenes and derivatives |
| PE(P-18:0/TXB2) | 3.75E-06 | 1.08E-05 | 0.5749 | 420.7682 | |
| Glutamylleucylarginine | 3.15E-08 | 2.02E-07 | 0.6711 | 430.2474 | Carboxylic acids and derivatives |
| Luteolin 4'-sulfate | 5.74E-07 | 2.05E-06 | 0.6851 | 364.9963 | Flavonoids |
| 2'-Fluorothymidine | 0.000569 | 0.000943 | 0.9245 | 225.0693 | Pyrimidine nucleosides |
| Gravacridonediol | 4.38E-10 | 6.54E-09 | 0.6968 | 342.1334 | Quinolines and derivatives |
| Biochanin A | 1.22E-06 | 3.91E-06 | 0.5843 | 283.0606 | Isoflavonoids |
| Zearalenone 4-sulfate | 2.84E-08 | 1.66E-07 | -0.5838 | 443.0996 | Macrolides and analogues |
| Butenoylcarnitine | 3.34E-09 | 2.98E-08 | 0.5694 | 686.3899 | Keto acids and derivatives |
| 5-Hydroxylysine | 2.99E-09 | 3.06E-08 | 0.6533 | 347.1923 | Carboxylic acids and derivatives |
| 1-[3,4-Dihydroxy-5-(hydroxymethyl)oxolan-2-yl]-5-hydroxyimidazole-4-carboxamide | 1.87E-10 | 3.45E-09 | -0.5391 | 259.0825 | Imidazole ribonucleosides and ribonucleotides |
| Acetyl-Ser-Asp-Lys-Pro | 1.11E-05 | 2.69E-05 | 0.6763 | 486.2193 | Carboxylic acids and derivatives |
| Topotecan | 7.32E-08 | 3.56E-07 | 0.5383 | 887.3259 | Camptothecins |
| Glycitein | 7.96E-12 | 2.57E-10 | 0.5214 | 283.0607 | Isoflavonoids |
| Cilengitide | 8.88E-11 | 1.93E-09 | 0.5677 | 553.2939 | Carboxylic acids and derivatives |
| Ala Val Leu Glu | 2.47E-10 | 4.26E-09 | 0.5985 | 431.2497 | |
| PA(8:0/20:5(5Z,8Z,10E,14Z,17Z)-OH(12)) | 9.43E-05 | 0.000183 | 0.5265 | 597.3241 | |
| 1-Aminocyclohexanecarboxylic acid | 1.03E-06 | 3.65E-06 | -0.6661 | 176.1282 | Prenol lipids |

| | | | | | |
|---|---|---|---|---|---|
| Phenylalanylvaline | 3.55E-11 | 9.34E-10 | -0.5395 | 297.1807 | Carboxylic acids and derivatives |
| 2-Quinolinecarboxylic acid | 3.27E-08 | 2.08E-07 | -0.6659 | 174.0551 | Quinolines and derivatives |
| Kaempferide | 9.10E-09 | 6.56E-08 | 0.5844 | 299.0554 | Flavonoids |
| Glycocholenate sulfate | 1.82E-08 | 1.15E-07 | 0.5477 | 542.2415 | Steroids and steroid derivatives |
| Macimorelin | 4.17E-13 | 3.95E-11 | 0.5938 | 538.2506 | Carboxylic acids and derivatives |
| Aminosalicylic Acid | 6.84E-10 | 9.35E-09 | 0.666 | 307.0923 | Benzene and substituted derivatives |
| 4,4'-DIMETHOXYDALBERGIONE | 1.53E-08 | 1.14E-07 | -0.5698 | 267.1014 | |
| Taurohyocholate | 3.17E-06 | 8.99E-06 | 0.5049 | 560.2923 | Steroids and steroid derivatives |
| Valylglutamine | 0.006005 | 0.008218 | 1.0856 | 246.1448 | Carboxylic acids and derivatives |
| 8-Ocimenyl acetate | 5.02E-09 | 4.65E-08 | -0.6723 | 236.1645 | Fatty Acyls |
| (3R, 6'Z)-3,4-Dihydro-8-hydroxy-3-(6-pentadecenyl)-1H-2-benzopyran-1-one | 1.57E-08 | 1.16E-07 | 0.5876 | 417.234 | Benzopyrans |
| (8R,9R,10S,13S,17R)-11,17-Dihydroxy-17-(2-hydroxyacetyl)-1,10,13-trimethyl-7,8,9,11,12,14,15,16-octahydro-6H-cyclopenta[a]phenanthren-3-one | 1.81E-06 | 5.82E-06 | 0.6224 | 388.219 | Steroids and steroid derivatives |
| Wogonin | 2.27E-10 | 3.98E-09 | 0.4413 | 285.0756 | |
| Isoleucyl-Threonine | 4.25E-09 | 4.06E-08 | 0.7088 | 215.1391 | Carboxylic acids and derivatives |
| Pectenotoxin 7 | 5.44E-08 | 3.15E-07 | 0.5897 | 456.2268 | Macrolides and analogues |
| Leu Gly Asn Val Thr | 8.23E-10 | 1.08E-08 | 0.6098 | 503.2822 | |
| N-Docosahexaenoyl Proline | 4.08E-09 | 3.96E-08 | 0.5566 | 470.2649 | |
| Pyrazofurin | 0.001132 | 0.001767 | 0.983 | 277.1117 | Nucleoside and nucleotide analogues |
| Dalfopristin | 2.19E-09 | 2.36E-08 | 0.5726 | 704.341 | Macrolide lactams |
| Ergocornine | 4.70E-06 | 1.32E-05 | 0.5509 | 562.3079 | Ergoline and derivatives |
| 5-Hydroxy-6-methoxyindole glucuronide | 2.75E-05 | 6.03E-05 | 0.6132 | 384.093 | Organooxygen compounds |
| Gardenin B | 2.14E-06 | 6.38E-06 | -0.5585 | 339.0873 | Flavonoids |
| PI(16:0/6 keto-PGF1alpha) | 8.11E-12 | 3.07E-10 | 0.5854 | 474.2552 | |
| Momordicoside E | 3.49E-07 | 1.34E-06 | 0.5223 | 731.3845 | Steroids and steroid derivatives |
| 14-Deoxy-11,12-Didehydroandrographolide | 5.85E-06 | 1.54E-05 | 0.5363 | 377.1962 | Prenol lipids |
| 3alpha-Hydroxyoreadone | 0.00054 | 0.0009 | 0.8217 | 270.1698 | Naphthofurans |
| Pro Glu Ala | 4.27E-11 | 1.07E-09 | 0.6217 | 316.15 | |
| Beta-D-Glucosamine | 1.71E-05 | 3.96E-05 | 0.5646 | 417.1732 | Organooxygen compounds |
| Muzanzagenin | 5.06E-11 | 1.22E-09 | 0.6078 | 487.2397 | Steroids and steroid derivatives |
| Suspensolide F | 7.42E-07 | 2.76E-06 | 0.6075 | 511.2402 | Prenol lipids |
| Gamma-Glutamyl-2-aminobutyrate | 2.02E-11 | 6.26E-10 | -0.6584 | 295.1156 | Carboxylic acids and derivatives |
| Limonoate a-ring-lactone | 2.18E-08 | 1.34E-07 | 0.4922 | 523.1743 | Prenol lipids |
| Prolylproline | 4.37E-11 | 1.09E-09 | 0.3971 | 213.1234 | Carboxylic acids and derivatives |
| 2-Methoxyestrone | 0.002111 | 0.003123 | 0.9648 | 345.1443 | Steroids and steroid derivatives |
| N-Eicosapentaenoyl Valine | 2.75E-07 | 1.19E-06 | 0.6612 | 446.2608 | |
| Val Ala Phe Asp | 1.98E-10 | 3.59E-09 | 0.5945 | 451.2184 | |
| 4-Hydroxyphenylacetic acid sulfate | 4.41E-05 | 9.23E-05 | 0.6833 | 230.9962 | Phenols |
| Eremopetasinorol | 4.46E-10 | 6.60E-09 | -0.6146 | 250.1802 | Organooxygen compounds |
| (Carbamoylamino) (2R)-2,5-diaminopentanoate | 5.91E-11 | 1.38E-09 | -0.6941 | 190.1075 | Carboxylic acids and derivatives |
| Trh-gly-lys | 2.73E-06 | 8.20E-06 | 0.591 | 590.3026 | Carboxylic acids and derivatives |
| GalNAc-beta1->4Gal-beta1->4Glc-beta1->1'Cer | 2.85E-06 | 8.52E-06 | 0.548 | 446.2337 | Sphingolipids |
| 6-Hydroxy-4-methoxy-3-(3-methyl-2-butenyl)-2-(2-phenylethenyl)benzoic acid | 8.96E-18 | 1.46E-14 | 0.5872 | 361.1424 | Stilbenes |
| 2-Chloro-N-icosa-5,8,11,14-tetraenylacetamide | 8.35E-11 | 1.57E-09 | 0.5771 | 400.219 | Carboxylic acids and derivatives |

| | | | | | |
|---|---|---|---|---|---|
| Diethylaminoethyl-Sephacel | 3.04E-08 | 1.75E-07 | 0.5325 | 415.219 | Organooxygen compounds |
| 16-Hydroxy-10-oxohexadecanoic acid | 2.37E-11 | 5.91E-10 | -0.5008 | 323.1605 | Fatty Acyls |
| Threonylhistidine | 6.70E-11 | 1.33E-09 | 0.6021 | 255.1093 | Carboxylic acids and derivatives |
| Leu Gln Val | 3.10E-10 | 5.11E-09 | 0.6144 | 359.2289 | |
| Benzyl sulfate | 2.16E-08 | 1.33E-07 | 0.5889 | 233.0119 | Benzene and substituted derivatives |
| L-beta-aspartyl-L-serine | 4.97E-06 | 1.33E-05 | 0.5776 | 219.0614 | Peptidomimetics |
| DG(2:0/0:0/LTE4) | 1.39E-08 | 9.31E-08 | 0.4315 | 554.2815 | |
| Leukotriene F4 | 4.66E-09 | 4.38E-08 | 0.5816 | 591.2733 | Fatty Acyls |
| PG(i-21:0/PGF2alpha) | 3.51E-05 | 7.55E-05 | 0.629 | 457.2948 | |
| Gamma-Glutamylcysteine | 1.15E-06 | 4.00E-06 | 0.6702 | 215.0485 | Carboxylic acids and derivatives |
| Libenzapril | 1.20E-14 | 2.73E-12 | 0.6128 | 405.2129 | Carboxylic acids and derivatives |
| N-Lauroylsarcosine | 2.49E-07 | 1.10E-06 | -0.5743 | 272.2219 | Carboxylic acids and derivatives |
| Ophiopogonin C' | 2.77E-08 | 1.82E-07 | 0.5461 | 373.2135 | Steroids and steroid derivatives |
| Polygonal | 7.29E-11 | 1.43E-09 | -0.4733 | 267.1596 | Organooxygen compounds |
| TYLVALOSIN | 8.17E-07 | 2.98E-06 | 0.5786 | 521.8056 | Phenanthrenes and derivatives |
| Thymine dimer | 1.16E-05 | 2.80E-05 | -0.6536 | 273.0625 | Diazines |
| 2,6-Dihydroxy-3-cyanopyridine | 1.09E-07 | 5.61E-07 | -0.5404 | 273.0617 | Pyridines and derivatives |
| Gln Phe Leu | 5.21E-08 | 3.04E-07 | 0.5931 | 407.2286 | |
| Murralongin | 1.48E-05 | 3.48E-05 | -0.5507 | 257.0814 | |
| Asn Leu Ile | 3.67E-06 | 1.06E-05 | 0.5861 | 359.2287 | |
| Bursin | 2.00E-08 | 1.41E-07 | -0.5782 | 322.2011 | Carboxylic acids and derivatives |
| Tryptophol | 4.81E-06 | 1.34E-05 | -0.5606 | 386.1853 | Indoles and derivatives |
| 5'-Methylthioadenosine | 1.43E-07 | 6.99E-07 | -0.5735 | 315.1225 | 5''-deoxyribonucleosides |
| Tyrosyl-Valine | 1.78E-10 | 3.31E-09 | -0.5643 | 313.1758 | Carboxylic acids and derivatives |
| 3-[4-(sulfooxy)phenyl]propanoic acid | 2.40E-10 | 3.67E-09 | 0.4672 | 245.0119 | Organic sulfuric acids and derivatives |
| Tetraazacyclododecanetetraacetic acid | 9.07E-09 | 6.56E-08 | 0.4707 | 403.1826 | Carboxylic acids and derivatives |
| Acetylcysteine | 2.73E-08 | 1.61E-07 | 0.5184 | 162.0219 | Carboxylic acids and derivatives |
| 7-Methylxanthine | 1.24E-08 | 8.40E-08 | 0.4822 | 497.1401 | Imidazopyrimidines |
| Physapruin A | 4.49E-08 | 2.69E-07 | 0.5605 | 500.2714 | Steroids and steroid derivatives |
| Eupatilin | 1.80E-07 | 8.40E-07 | -0.6111 | 309.0762 | Flavonoids |
| Pinometostat | 2.16E-09 | 2.11E-08 | 0.4816 | 583.3083 | 5''-deoxyribonucleosides |
| Gly Phe Ile | 7.57E-07 | 2.80E-06 | 0.639 | 336.1915 | |
| Byakangelicol | 7.51E-08 | 3.63E-07 | -0.546 | 361.0923 | Coumarins and derivatives |
| Salutaridinol | 1.04E-13 | 9.83E-12 | 0.4679 | 717.3411 | Phenanthrenes and derivatives |
| Aristospan | 8.52E-06 | 2.19E-05 | 0.755 | 267.1471 | Steroids and steroid derivatives |
| 14-HdoHE | 0.000163 | 0.000302 | 0.6372 | 401.2029 | Fatty Acyls |
| Epothilone A | 1.95E-08 | 1.39E-07 | 0.5532 | 535.2874 | Macrolides and analogues |
| Astragaloside III | 1.34E-15 | 4.02E-13 | 0.3559 | 765.4412 | Steroids and steroid derivatives |
| Esmolol | 6.00E-12 | 2.09E-10 | 0.4892 | 316.1506 | Phenol ethers |
| (3b,9R)-5-Megastigmene-3,9-diol 9-[apiosyl-(1->6)-glucoside] | 1.98E-08 | 1.40E-07 | 0.5605 | 529.2614 | Fatty Acyls |
| Suberylglycine | 1.14E-08 | 8.98E-08 | -0.5435 | 264.144 | Carboxylic acids and derivatives |
| Ethyl hydrogen sulfate | 1.44E-10 | 2.45E-09 | -0.4986 | 170.9958 | Organic sulfuric acids and derivatives |
| Normetanephrine | 8.09E-05 | 0.000159 | 0.6439 | 548.2563 | Phenols |

| | | | | | |
|---|---|---|---|---|---|
| 2-Amino-4-[carbamimidoyl(methyl)amino]butanoic acid | 1.93E-08 | 1.21E-07 | 0.4453 | 173.1034 | Carboxylic acids and derivatives |
| Plazomicin | 5.51E-11 | 1.31E-09 | 0.5245 | 557.3292 | Carboxylic acids and derivatives |
| (+/-)-3',4'-Methylenedioxy-5,7-dimethylepicatechin | 4.92E-09 | 4.57E-08 | -0.4614 | 331.1174 | Flavonoids |
| Pro Trp Phe | 3.19E-10 | 5.19E-09 | 0.5818 | 449.218 | |
| Metabolite A | 2.13E-10 | 3.32E-09 | 0.4509 | 815.3901 | Prenol lipids |
| Threonylarginine | 9.00E-10 | 1.15E-08 | 0.5565 | 589.2826 | Carboxylic acids and derivatives |
| Enalapril | 1.15E-08 | 9.06E-08 | 0.5974 | 394.2333 | Carboxylic acids and derivatives |
| Porphobilinogen | 1.82E-06 | 5.54E-06 | 0.6074 | 451.1823 | Organonitrogen compounds |
| CDP-DG(PGD1/a-17:0) | 2.23E-05 | 5.05E-05 | 0.7277 | 533.7741 | |
| Citbismine F | 6.77E-10 | 8.42E-09 | 0.4233 | 635.2024 | Quinolines and derivatives |
| Amastatin | 1.49E-08 | 9.87E-08 | 0.5216 | 473.2604 | Peptidomimetics |
| L-Arginine, L-asparaginylglycyl- | 8.55E-09 | 6.23E-08 | 0.5376 | 380.1455 | Carboxylic acids and derivatives |
| Cichorioside D | 2.00E-10 | 3.18E-09 | 0.4697 | 551.2127 | Organooxygen compounds |
| Thr Leu Ile | 3.12E-10 | 5.15E-09 | 0.6039 | 346.2334 | |
| Imazamox | 4.09E-07 | 1.54E-06 | 0.5577 | 350.135 | Carboxylic acids and derivatives |
| Glu Leu Ile | 6.24E-10 | 8.71E-09 | 0.45 | 396.2102 | |
| Butyl (S)-3-hydroxybutyrate glucoside | 3.52E-06 | 1.02E-05 | -0.634 | 355.197 | Fatty Acyls |
| PGP(18:1(11Z)/18:0) | 8.79E-08 | 4.66E-07 | 0.5644 | 451.2527 | Glycerophospholipids |
| Panax ginseng Tetrapeptide | 2.46E-06 | 7.51E-06 | 0.5514 | 459.2435 | Carboxylic acids and derivatives |
| Coformycin | 1.05E-07 | 4.83E-07 | 0.4985 | 283.1042 | Organooxygen compounds |
| 6-n-Octylaminouracil | 5.89E-11 | 1.21E-09 | 0.4592 | 284.1611 | Diazines |
| Tryptophyl-Threonine | 5.79E-10 | 7.42E-09 | 0.4985 | 304.1298 | Carboxylic acids and derivatives |
| Isoamericanol A | 1.58E-06 | 4.90E-06 | -0.4903 | 375.1078 | Benzodioxanes |
| Chenodeoxycholyltaurine | 1.53E-05 | 3.64E-05 | -0.5191 | 522.2861 | Steroids and steroid derivatives |
| Galactoarabinan | 5.69E-09 | 5.18E-08 | 0.4972 | 483.2084 | Organooxygen compounds |
| Glycyl-histidyl-arginyl-proline | 3.36E-06 | 9.81E-06 | 0.5544 | 430.23 | Carboxylic acids and derivatives |
| Fuegin | 3.80E-08 | 2.35E-07 | -0.5543 | 267.159 | |
| N-acetylmuramyl-l-alanine | 9.09E-09 | 7.52E-08 | -0.5197 | 347.145 | Carboxylic acids and derivatives |
| Phellopterin | 2.14E-06 | 6.37E-06 | -0.5148 | 345.0975 | Coumarins and derivatives |
| Ala Thr Val Glu | 2.40E-07 | 9.75E-07 | 0.4989 | 417.1983 | |
| 1-Oleoyl-2-{6-[(7-nitro-2-1,3-benzoxadiazol-4-yl)amino]hexanoyl}-sn-glycero-3-phospho-L-serine | 7.62E-08 | 3.67E-07 | 0.4468 | 844.3678 | |
| Deoxydihydro-artemisinin | 2.74E-08 | 1.80E-07 | -0.5677 | 286.2011 | Dioxolopyrans |
| Saxitoxin | 2.24E-07 | 1.01E-06 | -0.5414 | 341.1707 | Saxitoxins, gonyautoxins, and derivatives |
| Glu Asp Lys | 6.16E-06 | 1.66E-05 | 0.5444 | 391.1821 | |
| Geranylgeranyl-PP | 7.60E-06 | 1.99E-05 | 0.5732 | 492.2299 | Prenol lipids |
| Ile Glu Leu | 1.07E-07 | 5.50E-07 | 0.5524 | 374.2285 | |
| PG(TXB2/16:1(9Z)) | 1.01E-08 | 7.19E-08 | 0.4609 | 871.4336 | |
| Leu Tyr Ile | 1.34E-07 | 6.65E-07 | 0.5658 | 408.2489 | |
| Sorgolactone | 8.53E-07 | 2.89E-06 | -0.5683 | 315.124 | Prenol lipids |
| PIP(PGF1alpha/16:0) | 0.002078 | 0.00313 | 0.6497 | 494.2432 | |
| Furofoline | 3.88E-06 | 1.07E-05 | -0.4862 | 575.1481 | Quinolines and derivatives |
| Arginylglutamine | 5.94E-09 | 4.70E-08 | -0.5353 | 283.1548 | Carboxylic acids and derivatives |

| | | | | | |
|---|---|---|---|---|---|
| (S)-N-((S)-1-Amino-3,3-dimethyl-1-oxobutan-2-yl)-2-((S)-2-mercapto-4-(3,4,4-trimethyl-2,5-dioxoimidazolidin-1-yl)butanamido)-N,4-dimethylpentanamide | 2.15E-07 | 9.75E-07 | 0.5336 | 499.2872 | Peptidomimetics |
| Metabolite M6 | 5.15E-06 | 1.38E-05 | 0.4483 | 557.2926 | Indanes |
| Gonyautoxin I | 4.08E-07 | 1.54E-06 | -0.4719 | 448.0268 | Saxitoxins, gonyautoxins, and derivatives |
| Dyspropterin | 4.24E-07 | 1.59E-06 | 0.6302 | 218.0675 | Pteridines and derivatives |
| 1-Octen-3-yl primeveroside | 8.16E-07 | 2.77E-06 | 0.5273 | 467.2134 | Fatty Acyls |
| Val Thr Ile | 1.23E-07 | 6.16E-07 | 0.4488 | 332.2178 | |
| 5-(2-Hydroxyethyl)-4-methylthiazole | 4.18E-09 | 4.03E-08 | -0.4387 | 144.0479 | Azoles |
| Asp Ile Leu | 6.82E-08 | 3.79E-07 | 0.5709 | 360.2127 | |
| 2-Amino-3-methylbenzoate | 1.91E-06 | 6.08E-06 | -0.5733 | 303.1337 | Benzene and substituted derivatives |
| Valyl-prolyl-glycyl-valyl-glycine | 7.30E-08 | 3.55E-07 | 0.4464 | 472.2402 | Carboxylic acids and derivatives |
| N-Salicyloylaspartic acid | 2.31E-05 | 5.17E-05 | -0.5475 | 288.0297 | Carboxylic acids and derivatives |
| Asn Val Leu | 1.23E-09 | 1.47E-08 | 0.4095 | 345.2131 | |
| 2-Methyl-1-hydroxybutyl-ThPP | 8.72E-05 | 0.000171 | 0.6256 | 556.1183 | Diazines |
| Risbitin | 3.19E-07 | 1.25E-06 | -0.4604 | 443.3156 | Organooxygen compounds |
| 3-Hydroxy-cis-5-octenoylcarnitine | 2.48E-07 | 1.00E-06 | -0.4933 | 300.1811 | Fatty Acyls |
| Leucyl-Lysine | 1.37E-09 | 1.60E-08 | 0.6067 | 260.1969 | Carboxylic acids and derivatives |
| N2-Isobutyryl-2'-Deoxyguanosine | 3.37E-06 | 9.48E-06 | 0.6168 | 374.0864 | |
| Phthalide | 7.64E-09 | 6.57E-08 | -0.5191 | 307.0382 | Isocoumarans |
| 3-Pentadecylphenol | 3.20E-07 | 1.35E-06 | -0.4909 | 327.2681 | Phenols |
| Cnidilide | 2.48E-07 | 1.10E-06 | -0.5649 | 159.1169 | Isobenzofurans |
| Sinapic Acid | 2.61E-05 | 5.75E-05 | 0.569 | 223.0604 | Cinnamic acids and derivatives |
| PS(22:1(13Z)/22:6(5Z,8E,10Z,13Z,15E,19Z)-2OH(7S, 17S)) | 7.07E-08 | 3.46E-07 | 0.3946 | 956.5406 | |
| Isoscoparin 2''-(6-(E)-p-coumaroylglucoside) | 5.50E-10 | 7.85E-09 | 0.464 | 771.213 | Flavonoids |
| Isopropyl beta-D-glucoside | 8.83E-08 | 4.67E-07 | -0.5164 | 264.1439 | Organooxygen compounds |
| Echinacoside | 9.21E-10 | 1.08E-08 | 0.4437 | 767.2442 | Organooxygen compounds |
| Histidyltryptophan | 4.25E-08 | 2.31E-07 | 0.4919 | 340.1408 | Carboxylic acids and derivatives |
| 2-(acetylamino)-1,5-anhydro-2-deoxy-3-O-b-D-galactopyranosyl-D-arabino-Hex-1-enitol | 1.28E-08 | 9.85E-08 | -0.4876 | 398.1654 | Organooxygen compounds |
| 3-Cyclobutene-1,2-dione, 3-((3-(3-(4-(5-methoxy-4-pyrimidinyl)-1-piperazinyl)propyl)-1H-indol-5-yl)amino)-4-methyl- | 4.53E-07 | 1.82E-06 | -0.483 | 461.2277 | Indoles and derivatives |
| Prephenate | 1.03E-08 | 7.27E-08 | -0.4673 | 271.0468 | Keto acids and derivatives |
| Indolelactic Acid | 6.72E-10 | 9.20E-09 | 0.6172 | 206.0813 | Indoles and derivatives |
| L-Menthyl (R,S)-3-hydroxybutyrate | 1.35E-07 | 5.99E-07 | -0.4501 | 287.1859 | Prenol lipids |
| N-Acetylmuramate | 5.02E-05 | 0.000104 | -0.4465 | 276.1077 | Organooxygen compounds |
| 2-(3-Carboxy-3-aminopropyl)-L-histidine | 1.29E-10 | 2.59E-09 | 0.5355 | 257.1243 | Carboxylic acids and derivatives |
| (4Z,7Z,10Z,13Z,16E,18E)-20-Hydroxydocosa-4,7,10,13,16,18-hexaenoylcarnitine | 0.000771 | 0.001247 | 0.6745 | 470.3269 | Fatty Acyls |
| Asp Ile Lys | 5.30E-08 | 3.07E-07 | 0.399 | 375.2236 | |
| 1-Thiocarbamoyl-2-imidazolidinone | 1.97E-07 | 9.08E-07 | -0.6262 | 128.028 | Azolidines |
| Thr Ile Val | 1.28E-08 | 9.85E-08 | 0.5505 | 332.2178 | |
| Gly His | 8.21E-10 | 1.08E-08 | 0.5271 | 213.0983 | |
| 21-Deoxycortisol | 8.94E-07 | 3.22E-06 | -0.4594 | 385.1756 | Steroids and steroid derivatives |
| Isoleucylhydroxyproline | 2.57E-09 | 2.41E-08 | 0.4375 | 265.1189 | Carboxylic acids and derivatives |
| 4-Deacetylneosolaniol | 6.32E-07 | 2.41E-06 | -0.5237 | 363.1395 | Prenol lipids |

| | | | | | |
|---|---|---|---|---|---|
| Daprodustat | 3.64E-07 | 1.39E-06 | 0.5037 | 392.182 | Carboxylic acids and derivatives |
| Coumarin | 0.000295 | 0.000518 | 0.6022 | 191.0341 | Coumarins and derivatives |
| Acetyl-N-formyl-5-methoxykynurenamine | 4.96E-11 | 1.05E-09 | 0.4163 | 245.0926 | Organooxygen compounds |
| 4-Amino-1-piperidinecarboxylic acid | 1.47E-11 | 4.84E-10 | 0.6472 | 186.1239 | Piperidines |
| Citreoviridin | 3.12E-13 | 3.11E-11 | 0.5402 | 416.2126 | Pyrans |
| Cinncassiol C2 | 3.76E-09 | 3.70E-08 | 0.487 | 347.185 | Prenol lipids |
| PGP(20:2(11Z,14Z)/20:3(6,8,11)-OH(5)) | 1.22E-08 | 9.53E-08 | 0.5615 | 483.2504 | |
| 3,3'-Dihydroxy-4',5,7-trimethoxyflavan | 2.71E-09 | 2.51E-08 | -0.3627 | 331.1181 | Flavonoids |
| Asp Ile Val | 1.42E-05 | 3.41E-05 | 0.5862 | 346.1971 | |
| Oxyglutinosone | 9.39E-08 | 4.92E-07 | -0.498 | 269.1746 | Organooxygen compounds |
| (R)-Canadine | 0.01104 | 0.01447 | 0.8008 | 304.1331 | Protoberberine alkaloids and derivatives |
| Arg Met Thr | 3.94E-08 | 2.41E-07 | 0.4839 | 407.2032 | |
| Tolmetin | 2.07E-06 | 6.50E-06 | -0.4876 | 275.1389 | Organooxygen compounds |
| Cholylglutamine | 4.72E-12 | 1.78E-10 | 0.4416 | 571.3198 | Steroids and steroid derivatives |
| 9-Tetradecenoic acid | 1.76E-09 | 1.95E-08 | -0.3603 | 271.1651 | Fatty Acyls |
| Luvangetin | 1.47E-07 | 6.41E-07 | -0.508 | 257.0814 | |
| 1-nonadecanoyl-glycero-3-phosphate | 0.000112 | 0.000214 | 0.6551 | 238.1435 | Glycerophospholipids |
| Alanylproline | 3.70E-06 | 1.03E-05 | 0.5485 | 417.1983 | Carboxylic acids and derivatives |
| Dihydrozeatin-O-glucoside | 2.27E-07 | 1.02E-06 | 0.4981 | 384.1874 | Fatty Acyls |
| Docebenone | 9.43E-08 | 4.94E-07 | -0.5206 | 349.1756 | Fatty Acyls |
| PI(20:3(5Z,8Z,11Z)/20:3(8Z,11Z,14Z)-2OH(5,6)) | 2.96E-07 | 1.16E-06 | 0.4246 | 979.4986 | |
| Magnoflorine | 1.36E-06 | 4.30E-06 | 0.4558 | 729.3411 | Aporphines |
| Ser Leu Lys | 1.31E-06 | 4.44E-06 | 0.4385 | 347.2287 | |
| L-Canaline | 7.90E-10 | 9.58E-09 | 0.4387 | 401.2032 | Carboxylic acids and derivatives |
| Benazepril | 0.00011 | 0.00021 | 0.6002 | 459.1724 | Carboxylic acids and derivatives |
| 2,3-Dihydro-3-hydroxy-6-methoxy-2,2-dimethyl-4H-1-benzopyran-4-one | 4.66E-12 | 2.11E-10 | -0.3946 | 240.1231 | Benzopyrans |
| (2R)-2-Acetamido-6-hydroxy-2-(sulfanylmethyl)hex-3-enoic acid | 1.96E-06 | 5.92E-06 | -0.5113 | 254.0487 | Carboxylic acids and derivatives |
| LysoPC(0:0/20:4(5Z,8Z,11Z,14Z)) | 2.58E-06 | 7.48E-06 | 0.4534 | 588.3293 | Glycerophospholipids |
| Fludrocortisone acetate | 7.56E-06 | 1.98E-05 | -0.5233 | 486.2232 | Steroids and steroid derivatives |
| Aziridyl benzoquinone | 2.80E-05 | 6.17E-05 | -0.612 | 321.1438 | Organooxygen compounds |
| Cyclo(D-Trp-D-Asp-Pro-D-Ile-Leu) | 7.19E-06 | 1.90E-05 | 0.5099 | 335.1511 | Peptidomimetics |
| Gibberellin A20 | 9.90E-08 | 5.15E-07 | 0.5179 | 396.1763 | Prenol lipids |
| 1-Tert-Butyl 4-ethyl 3-oxopiperidine-1,4-dicarboxylate | 1.19E-06 | 4.09E-06 | -0.4972 | 335.16 | Piperidines |
| Deoxycholylalanine | 5.16E-09 | 4.24E-08 | -0.4177 | 484.3088 | |
| Acetylshikonin | 1.13E-06 | 3.94E-06 | -0.3942 | 348.1439 | Naphthalenes |
| Gamma-Glutamyl-2-aminobutyric acid | 0.004101 | 0.005877 | -0.7286 | 253.0798 | Carboxylic acids and derivatives |
| 2-Benzyl-aminoethanol | 2.43E-11 | 7.12E-10 | -0.5029 | 325.1864 | Benzene and substituted derivatives |
| Leu Gln Leu | 1.69E-10 | 3.16E-09 | 0.5605 | 373.2445 | |
| Butyl (S)-3-hydroxybutyrate [arabinosyl-(1->6)-glucoside] | 1.15E-10 | 2.38E-09 | 0.5003 | 472.24 | Fatty Acyls |
| Eugenol | 5.75E-05 | 0.000117 | 0.499 | 209.0811 | Phenols |
| PC(LTE4/18:3(9Z,12Z,15Z)) | 2.54E-10 | 4.37E-09 | 0.5529 | 470.2838 | |
| Methylisocitric acid | 7.77E-12 | 3.02E-10 | 0.6295 | 224.0778 | Carboxylic acids and derivatives |
| Etodolac | 4.08E-10 | 6.23E-09 | -0.46 | 320.1855 | Indoles and derivatives |

| | | | | | |
|---|---|---|---|---|---|
| Galactosyl 4-hydroxyproline | 1.61E-09 | 1.82E-08 | -0.411 | 276.108 | Carboxylic acids and derivatives |
| Cercosporamide | 8.41E-06 | 2.11E-05 | 0.5633 | 376.0654 | Benzene and substituted derivatives |
| Estriol | 1.72E-10 | 3.21E-09 | -0.3901 | 311.1601 | Steroids and steroid derivatives |
| Lysylhydroxyproline | 2.77E-09 | 2.56E-08 | 0.4049 | 304.1508 | Carboxylic acids and derivatives |
| Metoprolol | 1.49E-09 | 1.57E-08 | 0.4303 | 288.1561 | Phenols |
| (Z)-3-Oxo-2-(2-pentenyl)-1-cyclopenteneacetic acid | 1.78E-09 | 1.81E-08 | 0.4916 | 253.1076 | Organooxygen compounds |
| 2-(3-(2-Aminoethyl)-1h-indol-5-yloxy)acetyl-l-tyrosyl-glycinamide | 1.78E-05 | 4.10E-05 | -0.4808 | 474.1718 | Carboxylic acids and derivatives |
| Ferreirin | 2.90E-09 | 2.98E-08 | -0.4436 | 320.1126 | Isoflavonoids |
| TUBAIC ACID | 0.003512 | 0.0051 | 0.7533 | 219.0655 | |
| 2,3-Bis(3-hydroxybenzyl)butane-1,4-diol | 6.25E-07 | 2.20E-06 | -0.4687 | 301.144 | Dibenzylbutane lignans |
| Dibutylphthalic acid | 4.20E-08 | 2.56E-07 | -0.528 | 311.1827 | Benzene and substituted derivatives |
| Bursopoietin | 8.41E-10 | 1.09E-08 | -0.443 | 322.2011 | Carboxylic acids and derivatives |
| DG(20:4(5Z,8Z,11Z,13E)+=O(15)/0:0/i-19:0) | 1.64E-06 | 5.33E-06 | -0.4767 | 695.5216 | |
| Apigenin | 2.46E-06 | 7.18E-06 | 0.3418 | 269.0451 | Flavonoids |
| Delapril | 1.36E-06 | 4.60E-06 | -0.4528 | 470.2646 | Peptidomimetics |
| 2-Hydroxyiminostilbene | 5.84E-06 | 1.53E-05 | 0.4962 | 417.1621 | Benzazepines |
| 4-amino-4-deoxychorismate | 3.14E-10 | 5.15E-09 | -0.4919 | 190.05 | Carboxylic acids and derivatives |
| Zingerone | 0.004713 | 0.006673 | 0.8196 | 175.0754 | Phenols |
| Cis-Vaccenic acid | 1.57E-06 | 5.15E-06 | -0.4896 | 247.242 | Fatty Acyls |
| 2-Nonene-1,4-diol | 7.39E-09 | 5.60E-08 | -0.4332 | 203.128 | Fatty Acyls |
| 18-Nitrooctadec-9-enoic acid | 6.16E-10 | 7.79E-09 | 0.4772 | 364.1871 | Fatty Acyls |
| Nitecapone | 1.34E-05 | 3.19E-05 | -0.5289 | 300.03 | Cinnamic acids and derivatives |
| Felbamate | 5.25E-06 | 1.45E-05 | 0.4784 | 280.129 | Benzene and substituted derivatives |
| Gibberellin A51-catabolite | 7.62E-12 | 2.98E-10 | 0.4365 | 702.3302 | Prenol lipids |
| DG(18:4(6Z,9Z,12Z,15Z)/16:1(9Z)/0:0) | 8.28E-06 | 2.13E-05 | -0.5168 | 609.4485 | Fatty Acyls |
| 5-(Hydroxymethyl)-1H-pyrrole-2-carbaldehyde | 1.99E-07 | 8.33E-07 | 0.4552 | 309.1086 | Organooxygen compounds |
| Hydroxy Tyrosol -Acetate | 2.14E-06 | 6.68E-06 | -0.5243 | 226.1074 | Benzene and substituted derivatives |
| Petasitenine | 4.59E-07 | 1.84E-06 | -0.4288 | 346.1649 | Azaspirodecane derivatives |
| THYMOPENTIN | 7.35E-09 | 5.57E-08 | 0.4155 | 716.3091 | Carboxylic acids and derivatives |
| Okadaic acid | 1.87E-10 | 3.45E-09 | 0.4479 | 414.2346 | Organooxygen compounds |
| Indole-3-acetyl-myo-inositol | 3.37E-05 | 7.23E-05 | 0.5325 | 382.1136 | Indoles and derivatives |
| Emodin | 3.44E-07 | 1.33E-06 | 0.5169 | 269.045 | Anthracenes |
| 2',3,4',5-Tetrahydroxy-4-prenylstilbene | 2.39E-05 | 5.32E-05 | 0.467 | 623.2662 | Stilbenes |
| Tryptophyl-Glutamine | 2.12E-08 | 1.48E-07 | 0.4973 | 333.1558 | Carboxylic acids and derivatives |
| Asparaginyl-Tyrosine | 7.38E-08 | 3.58E-07 | 0.4593 | 294.1091 | Carboxylic acids and derivatives |
| N1-(alpha-D-ribosyl)-5,6-dimethyl-benzimidazole | 2.06E-14 | 2.78E-12 | 0.4227 | 323.1243 | Benzimidazole ribonucleosides and ribonucleotides |
| Lys Met Thr | 4.09E-09 | 3.96E-08 | 0.4993 | 379.1978 | |
| Phe Pro Thr | 7.97E-08 | 4.29E-07 | 0.4591 | 364.1876 | |
| Isoachifolidiene | 1.01E-07 | 4.66E-07 | 0.4451 | 389.1612 | Fatty Acyls |
| Anthra(1,9-cd)pyrazol-6(2H)-one, 2-(2-((2-hydroxyethyl)amino)ethyl)-5-((2-((2-hydroxyethyl)amino)ethyl)amino)- | 9.22E-09 | 7.62E-08 | 0.4497 | 473.2242 | Anthracenes |
| Doramectin | 1.59E-05 | 3.77E-05 | 0.4748 | 450.2633 | Macrolides and analogues |
| Asn Leu Lys | 3.01E-10 | 5.02E-09 | 0.4195 | 374.2395 | |

| | | | | | |
|---|---|---|---|---|---|
| (3R,5S,6R,7R,8S,9S,10S,13R,14S,17R)-6-Ethyl-17-((R)-4-hydroxybutan-2-yl)-10,13-dimethylhexadecahydro-1H-cyclopenta[a]phenanthrene-3,7-diol | 5.13E-07 | 1.86E-06 | -0.3865 | 391.3208 | Steroids and steroid derivatives |
| Chrysophanol | 0.00328 | 0.004702 | 0.7081 | 255.0651 | Anthracenes |
| Ilepatril | 2.67E-07 | 1.16E-06 | -0.4637 | 446.1818 | Carboxylic acids and derivatives |
| (6R,7R)-6-Methoxy-3,7-dimethyl-8-oxo-5-thia-1-azabicyclo[4.2.0]oct-2-ene-2-carboxylic acid | 0.003859 | 0.005458 | 0.8577 | 208.0428 | Carboxylic acids and derivatives |
| Delpazolid | 1.65E-08 | 1.07E-07 | -0.4399 | 329.1056 | Benzene and substituted derivatives |
| Tyr Pro Leu | 9.60E-06 | 2.43E-05 | 0.4458 | 392.2176 | |
| 4-Oxo-2-azetidinecarboxylic acid | 2.17E-06 | 6.75E-06 | 0.4603 | 133.0609 | Lactams |
| Soyasapogenol E | 1.72E-12 | 1.05E-10 | 0.4411 | 421.3461 | Prenol lipids |
| Galnac-1-4-glcnac | 8.93E-10 | 1.14E-08 | 0.4993 | 424.1713 | Organooxygen compounds |
| 5-Methylcytidine | 5.91E-09 | 5.33E-08 | 0.511 | 258.1084 | Pyrimidine nucleosides |
| N-Heptanoylglycine | 3.21E-11 | 8.61E-10 | -0.5341 | 205.1547 | Carboxylic acids and derivatives |
| Descarbonyl-lacosamide | 6.49E-12 | 2.65E-10 | -0.5214 | 250.1549 | Carboxylic acids and derivatives |
| 7-Ethyl-10-[4-(1-piperidino)-1-piperidino]carbonyloxycamptothecin | 6.17E-05 | 0.000125 | 0.4813 | 621.2509 | Camptothecins |
| 8-O-Acetyl shanzhiside methyl ester | 2.44E-06 | 7.14E-06 | 0.4098 | 483.1244 | Prenol lipids |
| 1,11-Undecanedicarboxylic acid | 4.89E-09 | 4.55E-08 | -0.4626 | 308.1857 | Fatty Acyls |
| (2R,4S)-1-Tert-Butyl 2-methyl 4-aminopyrrolidine-1,2-dicarboxylate | 8.52E-09 | 6.21E-08 | -0.4477 | 265.1189 | Carboxylic acids and derivatives |
| A-L-Arabinofuranosyl-(1->3)-b-D-xylopyranosyl-(1->4)-D-xylose | 3.05E-09 | 2.76E-08 | 0.339 | 449.106 | Organooxygen compounds |
| Gamma-Linolenic acid | 2.35E-06 | 7.21E-06 | -0.4538 | 296.2583 | Fatty Acyls |
| PE(22:6(4Z,7Z,10Z,13Z,16Z,19Z)/18:1(9Z)) | 1.24E-09 | 1.49E-08 | 0.4863 | 417.7575 | Glycerophospholipids |
| (2S)-1-[(2S)-3-(4H-Imidazol-4-yl)-2-[[(2S)-5-oxopyrrolidine-2-carbonyl]amino]propanoyl]pyrrolidine-2-carboxylic acid | 1.29E-05 | 3.08E-05 | 0.4722 | 400.106 | Carboxylic acids and derivatives |
| Panose | 1.07E-05 | 2.61E-05 | 0.4493 | 525.1445 | Organooxygen compounds |
| 1-(1-ethylindol-5-yl)sulfonyl-N-(3-pyrrolidin-1-ylbutyl)piperidine-4-carboxamide | 8.36E-08 | 4.46E-07 | 0.4322 | 461.2604 | |
| GPCho(18:1/16:1) | 0.007098 | 0.009609 | 0.5681 | 780.551 | Glycerophospholipids |
| 12S-HHT | 2.13E-10 | 3.81E-09 | -0.3255 | 325.1757 | Fatty Acyls |
| Homovanillic acid | 0.000184 | 0.000336 | 0.4051 | 181.0496 | Phenols |
| Humilixanthin | 1.69E-08 | 1.23E-07 | 0.4436 | 291.0974 | Carboxylic acids and derivatives |
| 2-Oxoarginine | 2.41E-09 | 2.29E-08 | 0.3385 | 154.0611 | Keto acids and derivatives |
| Cyclosomatostatin | 2.32E-05 | 5.24E-05 | 0.5689 | 390.7235 | Carboxylic acids and derivatives |
| T2 Triol | 5.60E-06 | 1.53E-05 | -0.421 | 446.2179 | Prenol lipids |
| Filipin II | 1.94E-11 | 5.08E-10 | 0.3738 | 637.394 | Macrolides and analogues |
| Pectolinarin | 9.38E-05 | 0.000182 | -0.4653 | 603.1763 | Tannins |
| Ursolic Acid | 8.77E-08 | 4.66E-07 | 0.5002 | 457.3671 | Prenol lipids |
| PL | 2.12E-10 | 3.32E-09 | 0.384 | 587.3034 | Macrolides and analogues |
| All-trans-18-Hydroxyretinoic acid | 2.18E-08 | 1.34E-07 | 0.373 | 361.2014 | Prenol lipids |
| Asp-Glu | 1.57E-06 | 5.15E-06 | 0.4423 | 263.0873 | Carboxylic acids and derivatives |
| Vulgarin | 3.97E-07 | 1.50E-06 | -0.395 | 285.1127 | Prenol lipids |
| Aflatoxin B1 dialcohol | 7.27E-08 | 3.99E-07 | -0.3201 | 348.1439 | Benzene and substituted derivatives |
| Furylacryloylalanyllysine | 3.88E-07 | 1.60E-06 | 0.4783 | 379.1973 | Carboxylic acids and derivatives |
| Bergenin | 1.84E-07 | 8.56E-07 | 0.4176 | 370.113 | Benzene and substituted derivatives |
| Etimicin | 4.42E-05 | 9.26E-05 | 0.5527 | 261.6497 | Organooxygen compounds |
| Sonolisib | 2.00E-09 | 1.98E-08 | 0.4074 | 560.2094 | Steroids and steroid derivatives |

| | | | | | |
|---|---|---|---|---|---|
| Asparaginyl-Threonine | 0.000364 | 0.000629 | 0.5129 | 465.1978 | Carboxylic acids and derivatives |
| Diphenylhydantoic acid | 1.29E-08 | 8.75E-08 | 0.3212 | 305.0694 | Benzene and substituted derivatives |
| Hovenidulcioside A2 | 8.97E-09 | 7.43E-08 | 0.4609 | 365.1981 | Prenol lipids |
| Kukoamine D | 1.20E-08 | 9.43E-08 | -0.3927 | 513.3068 | Phenols |
| Pindolol | 5.25E-06 | 1.45E-05 | 0.5175 | 271.1398 | Indoles and derivatives |
| 4-Hydroxyproline galactoside | 1.16E-07 | 5.89E-07 | -0.3099 | 276.1077 | Fatty Acyls |
| Pyriproxyfen | 0.03308 | 0.04055 | 1.1968 | 339.1703 | Benzene and substituted derivatives |
| 4-O-(Indole-3-acetyl)-D-glucopyranose | 5.63E-07 | 2.02E-06 | -0.4093 | 336.1083 | Indoles and derivatives |
| Russelioside B | 2.92E-06 | 8.72E-06 | 0.4453 | 410.2229 | Steroids and steroid derivatives |
| Estetrol | 5.32E-06 | 1.46E-05 | 0.4789 | 672.3557 | Steroids and steroid derivatives |
| Dopamine quinone | 0.000583 | 0.000965 | 0.5973 | 134.0602 | Organooxygen compounds |
| Septacidin | 2.08E-08 | 1.45E-07 | 0.4228 | 322.6898 | Carboxylic acids and derivatives |
| Asparaginyl-Phenylalanine | 1.43E-07 | 7.00E-07 | 0.4215 | 622.2553 | Carboxylic acids and derivatives |
| His Val | 9.61E-10 | 1.20E-08 | 0.4286 | 255.1451 | |
| Asparaginyl-Valine | 8.58E-12 | 3.19E-10 | -0.4158 | 214.1187 | Carboxylic acids and derivatives |
| 4-Hydroxy-6-octenoylcarnitine | 7.75E-08 | 4.20E-07 | -0.3709 | 302.1961 | Fatty Acyls |
| 3b-Hydroxy-17-(1h-1,2,3-triazol-1-yl)androsta-5,16-diene | 2.93E-14 | 5.39E-12 | 0.4723 | 362.2177 | Steroids and steroid derivatives |
| Androstane-3,17-diol 17-sulfate | 6.36E-06 | 1.65E-05 | 0.4172 | 417.1983 | Steroids and steroid derivatives |
| Gln Leu Arg | 8.20E-08 | 4.40E-07 | 0.4189 | 416.2613 | |
| 7,8-Dihydropteroic acid | 4.78E-07 | 1.75E-06 | 0.4819 | 335.088 | Pteridines and derivatives |
| M-Coumaric acid | 5.83E-07 | 2.08E-06 | -0.3203 | 327.0868 | Cinnamic acids and derivatives |
| Adipic dihydrazide | 2.28E-07 | 1.03E-06 | 0.3802 | 213.0749 | Carboxylic acids and derivatives |
| 4-[(2,4-Dihydroxy-3,3-dimethylbutanoyl)amino]butanoic acid | 1.95E-07 | 9.01E-07 | -0.3867 | 234.1337 | Carboxylic acids and derivatives |
| Asn Val Ile | 1.34E-09 | 1.57E-08 | 0.386 | 345.2131 | |
| Indoleacetaldehyde | 3.76E-06 | 1.08E-05 | -0.4495 | 382.1541 | Indoles and derivatives |
| Kanamycin | 7.01E-09 | 6.14E-08 | 0.4026 | 449.2241 | Organooxygen compounds |
| Gracillin | 1.11E-05 | 2.75E-05 | 0.4695 | 465.2285 | Steroids and steroid derivatives |
| Hesperidin | 5.89E-07 | 2.28E-06 | 0.3829 | 652.2294 | Flavonoids |
| Arginyltryptophan | 3.54E-05 | 7.61E-05 | 0.5199 | 374.2047 | Carboxylic acids and derivatives |
| 4,4-Dimethyl-2-[3-carboxylatopropyl]-2-tridecyloxazolidine 3-oxide | 6.64E-06 | 1.71E-05 | -0.3804 | 366.3003 | Fatty Acyls |
| Nystatin A3 | 1.11E-05 | 2.76E-05 | 0.4571 | 528.7878 | Organooxygen compounds |
| Physagulin E | 1.60E-06 | 5.23E-06 | 0.4362 | 739.3502 | Steroids and steroid derivatives |
| 1-O-all-trans-retinoyl-beta-glucuronic Acid | 1.61E-07 | 7.68E-07 | 0.4578 | 509.2759 | Prenol lipids |
| Ubiquinone-1 | 7.16E-09 | 6.25E-08 | -0.4771 | 251.1254 | Prenol lipids |
| N-Acetylcaprolactam | 4.52E-07 | 1.82E-06 | 0.5602 | 173.1286 | |
| Spermic acid 2 | 2.91E-07 | 1.14E-06 | 0.4083 | 213.1237 | Carboxylic acids and derivatives |
| Prostaglandin D1 Alcohol | 1.12E-06 | 3.65E-06 | -0.4102 | 339.2531 | |
| 3-Phenylpropyl 2-methylpropanoate | 7.00E-11 | 1.58E-09 | -0.3836 | 248.1645 | Benzene and substituted derivatives |
| Asp Trp | 2.94E-06 | 8.75E-06 | 0.4906 | 320.124 | |
| Glycyl-Lysine | 3.69E-06 | 1.03E-05 | 0.4277 | 202.1188 | Carboxylic acids and derivatives |
| PGP(20:2(11Z,14Z)/PGF1alpha) | 8.71E-07 | 3.15E-06 | 0.4906 | 478.269 | |
| Phe Glu Val | 2.25E-06 | 6.97E-06 | 0.4477 | 394.1971 | |
| 1-Amino-2-methylanthraquinone | 1.51E-07 | 7.28E-07 | -0.462 | 260.0698 | Anthracenes |
| N-[(5-Hydroxy-2-pyridinyl)methyl]adenosine | 5.75E-07 | 2.05E-06 | 0.3998 | 419.1303 | Purine nucleosides |

| | | | | | |
|---|---|---|---|---|---|
| Sativan | 1.41E-08 | 9.41E-08 | -0.3306 | 331.118 | Isoflavonoids |
| Histamine | 1.05E-05 | 2.62E-05 | 0.6286 | 112.0873 | Organonitrogen compounds |
| CDP-DG(20:4(7E,9E,11Z,13E)-3OH(5S,6R,15S)/i-12:0) | 1.78E-06 | 5.73E-06 | 0.4278 | 497.7258 | |
| Daunomycinone | 1.09E-08 | 7.58E-08 | -0.4022 | 443.0993 | Naphthacenes |
| LysoPC(20:3(8Z,11Z,14Z)/0:0) | 0.000216 | 0.000389 | 0.4154 | 590.3448 | Glycerophospholipids |
| L-Leucine | 1.10E-06 | 3.84E-06 | 0.5121 | 173.1286 | Carboxylic acids and derivatives |
| 3,7,8,15-Scirpenetetrol | 7.05E-08 | 3.89E-07 | -0.416 | 362.1596 | Prenol lipids |
| Hernandulcin | 5.49E-09 | 5.01E-08 | -0.4055 | 278.2113 | Prenol lipids |
| Nitroethane | 9.50E-06 | 2.35E-05 | 0.5552 | 149.0565 | Allyl-type 1,3-dipolar organic compounds |
| Norerythromycin | 1.62E-10 | 2.67E-09 | 0.3801 | 740.3939 | Steroids and steroid derivatives |
| Graveoline | 0.006441 | 0.008932 | 0.6998 | 324.087 | Quinolines and derivatives |
| Decarbamoylneosaxitoxin | 1.52E-10 | 2.55E-09 | -0.3533 | 317.1236 | Saxitoxins, gonyautoxins, and derivatives |
| Asn Glu Phe | 2.25E-07 | 1.01E-06 | 0.4361 | 409.1716 | |
| 7-Hydroxyetodolac | 3.01E-09 | 3.08E-08 | -0.3999 | 286.1439 | Indoles and derivatives |
| N-[(3s)-2-Oxotetrahydrofuran-3-Yl]butanamide | 9.92E-08 | 4.59E-07 | -0.3119 | 216.087 | Carboxylic acids and derivatives |
| 7,4'-Dihydroxy-8-methylflavan | 4.95E-06 | 1.33E-05 | -0.4614 | 301.1076 | Flavonoids |
| 15-Hydroxynorandrostene-3,17-dione glucuronide | 3.40E-06 | 9.56E-06 | 0.4651 | 463.1941 | Steroids and steroid derivatives |
| Notoginsenoside R10 | 5.13E-05 | 0.000106 | 0.4066 | 577.3343 | Steroids and steroid derivatives |
| Pro Asn Leu | 0.002025 | 0.003007 | 0.5593 | 343.1974 | |
| 7,4'-Dihydroxyflavone | 4.48E-10 | 6.01E-09 | 0.297 | 253.0502 | Flavonoids |
| (2E)-Oct-2-enedioylcarnitine | 7.59E-06 | 1.99E-05 | -0.4767 | 315.1659 | Fatty Acyls |
| Parylene C | 2.04E-08 | 1.26E-07 | 0.3112 | 321.0432 | |
| 3-(2-Hydroxy-4-methylphenyl)-2-butanone | 0.002579 | 0.003759 | 0.7106 | 220.1333 | Prenol lipids |
| Corticosterone | 1.87E-05 | 4.32E-05 | 0.4602 | 731.3936 | Steroids and steroid derivatives |
| 4-Methoxyestrone | 2.74E-07 | 1.19E-06 | 0.4383 | 364.1864 | Carboxylic acids and derivatives |
| (2E,4E)-Hexa-2,4-dienedioylcarnitine | 0.000102 | 0.000198 | 0.4739 | 303.1552 | Fatty Acyls |
| Aloeresin B | 2.32E-05 | 5.19E-05 | -0.4391 | 393.1156 | Organooxygen compounds |
| PC(2:0/PGE1) | 7.34E-06 | 1.93E-05 | 0.4081 | 649.3535 | |
| N-(N-(3-Carboxyoxirane-2-carbonyl)leucyl)isoamylamine | 3.85E-08 | 2.37E-07 | -0.4195 | 356.2178 | Carboxylic acids and derivatives |
| Arenobufagin | 8.75E-09 | 7.29E-08 | 0.4039 | 439.2083 | Steroids and steroid derivatives |
| Val Ala Ile Glu | 5.39E-09 | 4.94E-08 | 0.3908 | 431.25 | |
| Glu Val Leu | 2.57E-07 | 1.13E-06 | 0.4676 | 360.2127 | |
| CDP-DG(a-13:0/20:4(6E,8Z,11Z,13E)-2OH(5S,15S)) | 5.51E-11 | 1.31E-09 | 0.4363 | 496.7376 | |
| Asn Ile Phe | 6.81E-08 | 3.79E-07 | 0.421 | 393.2127 | |
| Ala-Hyp-Gly | 8.06E-10 | 9.72E-09 | 0.3839 | 304.1144 | Carboxylic acids and derivatives |
| Thr Trp Glu | 3.43E-08 | 2.15E-07 | 0.4342 | 435.1871 | |
| Val Ile Tyr | 0.000102 | 0.000197 | 0.4822 | 394.2337 | |
| Tryptophyl-Lysine | 5.43E-06 | 1.44E-05 | 0.4357 | 377.1823 | Carboxylic acids and derivatives |
| PE(14:1(9Z)/20:4(6E,8Z,11Z,14Z)-OH(5S)) | 0.001505 | 0.002294 | 0.5786 | 374.7335 | |
| Trp Trp | 2.35E-07 | 1.05E-06 | 0.3899 | 391.1761 | |
| Cannabinol | 3.52E-05 | 7.57E-05 | -0.454 | 333.1808 | Benzopyrans |
| Chenodeoxycholyllysine | 8.58E-09 | 7.18E-08 | -0.4179 | 521.3948 | Steroids and steroid derivatives |

| | | | | | |
|---|---|---|---|---|---|
| Glu Ile Val | 1.12E-08 | 8.84E-08 | 0.4 | 360.2128 | |
| 25-Hydroxyvitamin D3-26,23-lactol | 6.36E-06 | 1.70E-05 | 0.4455 | 475.2762 | Steroids and steroid derivatives |
| (R)-2-(4-(Tert-butoxycarbonyl)morpholin-3-yl)acetic acid | 7.57E-10 | 1.01E-08 | -0.4179 | 210.1125 | Oxazinanes |
| Tigloidine | 1.26E-13 | 1.56E-11 | -0.4612 | 265.1909 | Tropane alkaloids |
| 7-Methylrosmanol | 8.84E-07 | 2.98E-06 | -0.388 | 381.1708 | Prenol lipids |
| 1-Heptadecanoylglycerophosphoethanolamine | 0.000709 | 0.001161 | 0.4454 | 512.2977 | Glycerophospholipids |
| Ala His Phe Asp | 6.31E-05 | 0.000127 | 0.4793 | 487.1935 | |
| Thr Val Leu | 2.35E-06 | 7.22E-06 | 0.4324 | 332.2178 | |
| Met Ile Ile | 4.65E-07 | 1.86E-06 | 0.4062 | 376.2241 | |
| 5-Hydroxylysinonorleucine | 9.89E-10 | 1.14E-08 | 0.3742 | 272.1617 | Carboxylic acids and derivatives |
| Glycyl-glycine | 1.11E-05 | 2.76E-05 | 0.5406 | 177.0248 | Carboxylic acids and derivatives |
| Gln Leu Lys | 8.62E-07 | 3.12E-06 | 0.3934 | 194.6315 | |
| Ala-Val-OH | 1.26E-05 | 3.07E-05 | 0.4877 | 297.108 | |
| (a-D-mannosyl)2-b-D-mannosyl-N-acetylglucosamine | 9.99E-07 | 3.31E-06 | 0.3639 | 742.2162 | Organooxygen compounds |
| PS(20:4(5E,8Z,12Z,14Z)-OH(11R)/15:0) | 2.43E-06 | 7.44E-06 | 0.4439 | 415.7344 | |
| Betulinic acid | 3.91E-07 | 1.60E-06 | 0.3709 | 457.3672 | Prenol lipids |
| Gly Leu Val | 2.43E-08 | 1.66E-07 | 0.3447 | 288.1916 | |
| Gabazine | 3.91E-09 | 3.39E-08 | 0.3868 | 332.1246 | Diazines |
| 6-Hydroxyshogaol | 0.00928 | 0.01257 | 0.5676 | 327.1344 | Phenols |
| Ruscogenin | 9.03E-05 | 0.000177 | 0.4878 | 475.2757 | Prenol lipids |
| 3-hydroxyicosanoic Acid | 5.73E-06 | 1.51E-05 | -0.4187 | 327.2897 | Fatty Acyls |
| (2R,3R,4R,5R)-2-Amino-4,5,6-trihydroxy-3-[(2R)-1-oxopropan-2-yl]oxyhexanal | 3.42E-05 | 7.39E-05 | -0.3317 | 236.1128 | Organooxygen compounds |
| Glutethimide | 1.19E-07 | 6.01E-07 | 0.4492 | 250.1438 | Piperidines |
| Biliverdin | 0.000194 | 0.000353 | 0.3939 | 583.2549 | Tetrapyrroles and derivatives |
| 3-[2-[[(1S,2R,3S)-3-[4-(Pentylcarbamoyl)-1,3-oxazol-2-yl]-7-oxabicyclo[2.2.1]heptan-2-yl]methyl]phenyl]propanoic acid | 0.003965 | 0.005593 | 0.6291 | 423.2274 | Phenylpropanoic acids |
| Tyramine-O-sulfate | 0.001413 | 0.002188 | 0.5368 | 216.0328 | Organic sulfuric acids and derivatives |
| 1-(4-Hydroxy-3-methoxyphenyl)-3-decanone | 8.37E-07 | 3.05E-06 | 0.4449 | 317.1495 | Phenols |
| LysoPA(P-16:0/0:0) | 7.84E-08 | 3.75E-07 | -0.2978 | 439.2456 | Glycerophospholipids |
| Pentanoic acid, 5-(dipentylamino)-5-oxo-4-((3-quinolinylcarbonyl)amino)-, (R)- | 1.02E-09 | 1.26E-08 | 0.3125 | 442.2695 | Carboxylic acids and derivatives |
| Cyclo(glycylglycylleucylleucylleucylprolylprolylphenylalanyl) | 2.46E-06 | 7.51E-06 | 0.3577 | 398.2395 | Carboxylic acids and derivatives |
| Protoporphyrinogen IX | 5.40E-06 | 1.48E-05 | 0.4223 | 632.3248 | Tetrapyrroles and derivatives |
| 3,4-Methylenesebacic acid | 6.72E-12 | 2.72E-10 | -0.3561 | 209.1173 | Fatty Acyls |
| 4alpha-Methylzymosterol | 1.45E-10 | 2.83E-09 | 0.3904 | 421.3462 | Steroids and steroid derivatives |
| Gly-Pro-Gly-Arg-Ala-Phe | 1.63E-05 | 3.84E-05 | 0.4384 | 586.3082 | Carboxylic acids and derivatives |
| N-Palmitoyl Cysteine | 1.35E-07 | 5.99E-07 | 0.3143 | 567.3163 | Fatty Acyls |
| 1-O-Caffeoyl-beta-D-glucose | 9.79E-06 | 2.47E-05 | 0.4871 | 384.1288 | Steroids and steroid derivatives |
| CI-1044 | 4.04E-14 | 4.61E-12 | 0.332 | 378.1371 | Carboxylic acids and derivatives |
| 2-(Arabinosylamino)-3-(glucosylamino)propanenitrile | 1.63E-12 | 7.89E-11 | 0.3826 | 360.1406 | Organooxygen compounds |
| Salicylic Acid | 0.000294 | 0.000515 | 0.6291 | 121.0286 | Benzene and substituted derivatives |
| Cerebellin | 1.06E-06 | 3.73E-06 | 0.3763 | 816.9181 | Polypeptides |
| Beta-D-ribosylnicotinate | 8.08E-09 | 6.85E-08 | 0.4028 | 297.108 | Organooxygen compounds |
| S-Sulfo-L-Cysteine | 2.57E-09 | 2.41E-08 | 0.3024 | 199.9683 | Carboxylic acids and derivatives |

| | | | | | |
|---|---|---|---|---|---|
| Desglucocheirotoxol | 7.76E-09 | 6.64E-08 | 0.375 | 570.3245 | Steroids and steroid derivatives |
| (R)-6'-O-(4-Geranyloxy-2-hydroxycinnamoyl)-marmin | 4.91E-10 | 7.11E-09 | -0.3221 | 613.3222 | Prenol lipids |
| Thr Val Val | 2.12E-09 | 2.30E-08 | 0.3795 | 318.2022 | |
| Ser Lys | 1.04E-07 | 5.37E-07 | 0.4146 | 234.1449 | |
| HDMBOA-Glc | 1.32E-06 | 4.17E-06 | 0.2995 | 368.098 | Organooxygen compounds |
| Kynurenic Acid | 2.47E-08 | 1.68E-07 | -0.3878 | 190.0499 | Quinolines and derivatives |
| 7-Hydroxy-3-(3-hydroxy-4-methoxybenzyl)-5-methoxy-4-chromanone | 2.00E-08 | 1.25E-07 | -0.2781 | 329.1024 | Homoisoflavonoids |
| N-Jasmonoyltyrosine | 3.11E-08 | 1.78E-07 | 0.3664 | 394.1611 | Carboxylic acids and derivatives |
| Deoxypyridinoline | 1.25E-07 | 6.23E-07 | 0.3649 | 445.229 | Carboxylic acids and derivatives |
| 1-Octen-3-yl glucoside | 0.002264 | 0.003336 | 0.5231 | 603.3346 | Fatty Acyls |
| Milbemycin alpha9 | 1.06E-06 | 3.47E-06 | 0.3665 | 674.2773 | Macrolides and analogues |
| 9-(3-Imidazol-1-yl-2,6,6-trimethylcyclohexen-1-yl)-3,7-dimethylnona-2,4,6,8-tetraenoic acid | 1.23E-05 | 2.96E-05 | 0.3943 | 401.2033 | Prenol lipids |
| Ibuprofen | 4.54E-06 | 1.28E-05 | 0.4975 | 207.1379 | Phenylpropanoic acids |
| 2-Amino-3-(4-(4-hydroxyphenoxy)-3-iodophenyl)propanoic acid | 5.56E-05 | 0.000114 | 0.4148 | 433.965 | Carboxylic acids and derivatives |
| Asp Asp Ile | 8.26E-08 | 4.41E-07 | 0.3918 | 362.1555 | |
| 4-Methyl-2-oxo-2H-chromen-7-yl sulfamate | 1.24E-06 | 3.96E-06 | -0.4104 | 275.9932 | Coumarins and derivatives |
| 7-Hydroxyflumequine | 0.000125 | 0.000236 | -0.3623 | 260.074 | Quinolines and derivatives |
| N-(2-Hydroxypropyl)valine | 9.30E-09 | 7.66E-08 | -0.3122 | 176.1282 | Carboxylic acids and derivatives |
| 4-methoxy-3-(sulfooxy)benzoic acid | 5.55E-06 | 1.47E-05 | 0.4565 | 246.9913 | Benzene and substituted derivatives |
| Leu Leu Lys | 2.48E-05 | 5.55E-05 | 0.4421 | 373.2807 | |
| NeuNGc | 6.84E-11 | 1.35E-09 | 0.28 | 324.0929 | Organooxygen compounds |
| TRIETHYLENE GLYCOL | 4.44E-09 | 3.73E-08 | 0.3705 | 345.177 | Organooxygen compounds |
| 4-Chlorophenylacetic acid | 0.001065 | 0.001688 | -0.4872 | 169.0067 | Benzene and substituted derivatives |
| (2S)-2-(Diaminomethylideneamino)-3-phenylpropanoic acid | 5.60E-08 | 2.87E-07 | -0.4354 | 188.0818 | Carboxylic acids and derivatives |
| Ritonavir | 5.54E-07 | 2.16E-06 | 0.3811 | 720.3195 | Carboxylic acids and derivatives |
| Genistein | 2.14E-05 | 4.87E-05 | 0.3267 | 271.06 | Isoflavonoids |
| 4a-Carbinolamine tetrahydrobiopterin | 4.06E-05 | 8.58E-05 | 0.5039 | 222.0986 | Pteridines and derivatives |
| Edivoxetine | 8.38E-09 | 6.14E-08 | 0.3353 | 374.1563 | Phenol ethers |
| 3-((((-Oxoheptyl)amino)acetyl)amino)methyl-7-oxobicyclo(2.2.1)hept-2-yl-5-heptenoic acid | 6.09E-07 | 2.34E-06 | 0.4456 | 445.2654 | Fatty Acyls |
| PE(22:6(4Z,7Z,10Z,13E,15E,19Z)-OH(17)/22:6(4Z,7Z,10Z,13Z,16Z,19Z)) | 4.04E-05 | 8.55E-05 | 0.461 | 448.7455 | |
| PE(20:5(5Z,8Z,11Z,14Z,16E)-OH(18R)/DiMe(11,3)) | 2.35E-05 | 5.29E-05 | 0.414 | 432.7447 | |
| Tezosentan | 2.77E-07 | 1.10E-06 | -0.3264 | 642.1286 | Diazines |
| Olprinone | 6.48E-10 | 8.12E-09 | -0.357 | 295.0851 | Pyridines and derivatives |
| 3-Ethyl-1,2-cyclopentanedione | 3.20E-09 | 2.88E-08 | -0.3893 | 107.049 | Organooxygen compounds |
| Hexazinone | 0.000113 | 0.000215 | -0.2803 | 563.3337 | Organonitrogen compounds |
| Coumachlor | 0.000386 | 0.000663 | 0.5029 | 379.0123 | Coumarins and derivatives |
| Nanaomycin | 1.32E-08 | 8.87E-08 | -0.2893 | 301.0712 | |
| 4-Amino-5-hydroxymethyl-2-methylpyrimidine | 5.55E-07 | 2.17E-06 | 0.4088 | 320.1816 | Diazines |
| Zizybeoside I | 3.19E-05 | 6.93E-05 | -0.4485 | 455.1519 | Organooxygen compounds |
| Estrone sulfate | 5.96E-06 | 1.56E-05 | -0.327 | 331.1035 | Steroids and steroid derivatives |
| 4-Pentenoic acid, 2-fluoro-2-propyl- | 8.34E-10 | 9.99E-09 | 0.3588 | 365.1811 | Fatty Acyls |
| Daidzein | 9.27E-09 | 7.64E-08 | 0.2868 | 255.0652 | Isoflavonoids |

| | | | | | |
|---|---|---|---|---|---|
| Arginylisoleucine | 4.63E-06 | 1.30E-05 | 0.3978 | 288.2029 | Carboxylic acids and derivatives |
| L-N-(1H-Indol-3-ylacetyl)aspartic acid | 2.86E-07 | 1.13E-06 | 0.4387 | 335.0879 | Carboxylic acids and derivatives |
| Oxyphencyclimine | 1.54E-10 | 2.94E-09 | 0.3714 | 389.1817 | Diazines |
| LysoPS(16:0/0:0) | 1.06E-07 | 4.86E-07 | 0.3493 | 534.2221 | Glycerophospholipids |
| Therafectin | 6.33E-10 | 8.82E-09 | -0.3726 | 288.1807 | Organooxygen compounds |
| 17-Ethynyl-16-fluoroestradiol | 2.67E-05 | 5.93E-05 | 0.4717 | 328.1815 | Steroids and steroid derivatives |
| Azimexon | 6.71E-11 | 1.33E-09 | 0.3586 | 387.2241 | Carboxylic acids and derivatives |
| P-Hydroxyphenethyl trans-ferulate | 6.83E-07 | 2.38E-06 | -0.3058 | 313.1077 | Cinnamic acids and derivatives |
| Sparstolonin B | 1.68E-07 | 7.22E-07 | 0.3462 | 303.0072 | Benzopyrans |
| Fructosyl-lysine | 5.59E-06 | 1.48E-05 | 0.3963 | 307.1505 | Organooxygen compounds |
| 3,4-Dihydroxyphenylpropanoate | 5.78E-07 | 2.06E-06 | 0.3398 | 181.0496 | |
| Farnesylcysteine | 1.35E-06 | 4.26E-06 | 0.3348 | 360.1769 | Prenol lipids |
| (5Z,8Z,13E,15S)-11,12,15-Trihydroxyicosa-5,8,13-trienoylcarnitine | 1.14E-06 | 3.96E-06 | 0.3066 | 249.6736 | Fatty Acyls |
| Coniferin | 6.44E-11 | 1.30E-09 | 0.3482 | 377.0983 | Organooxygen compounds |
| 3-hydroxyquinidine | 8.10E-11 | 1.54E-09 | 0.3565 | 361.1512 | Cinchona alkaloids |
| Ile Arg | 8.24E-07 | 3.01E-06 | 0.3343 | 288.2029 | |
| Glycinoeclepin C | 0.000114 | 0.000217 | 0.4974 | 549.2299 | Prenol lipids |
| DG(10:0/a-21:0/0:0) | 4.47E-06 | 1.22E-05 | -0.335 | 575.4662 | Glycerolipids |
| 2,3-Diaminopyridine | 7.39E-09 | 6.39E-08 | 0.3556 | 110.0716 | |
| 17-Hydroxypregnenolone sulfate | 4.75E-06 | 1.28E-05 | -0.3703 | 411.1869 | Steroids and steroid derivatives |
| Lavoltidine | 3.51E-07 | 1.47E-06 | 0.4324 | 359.2287 | Piperidines |
| Kinetensin 1-8 | 0.000497 | 0.000835 | 0.4917 | 541.287 | Carboxylic acids and derivatives |
| 4-Nitrophenyl beta-D-xyloside | 0.000169 | 0.000311 | -0.4397 | 236.0553 | Organooxygen compounds |
| Mollicellin C | 6.50E-06 | 1.68E-05 | -0.381 | 449.0614 | Depsides and depsidones |
| (S)-4-(4-((2-(3,5-Dimethylphenyl)pyrrolidin-1-yl)Methyl)phenoxy)-3-fluorobenzaMide | 3.31E-08 | 1.87E-07 | 0.3602 | 417.1983 | Benzene and substituted derivatives |
| 6-Hydroxysandoricin | 1.33E-05 | 3.23E-05 | -0.3513 | 637.2864 | Carboxylic acids and derivatives |
| Hymenoxon | 8.15E-10 | 1.07E-08 | 0.3716 | 265.1434 | Oxanes |
| H-D-Tyr-val-gly-OH | 3.32E-07 | 1.29E-06 | 0.3358 | 318.1461 | Carboxylic acids and derivatives |
| Dihydroxylysinonorleucine | 8.86E-05 | 0.000173 | 0.3902 | 472.2399 | Carboxylic acids and derivatives |
| PC(14:1(9Z)/PGE2) | 9.97E-08 | 5.18E-07 | 0.375 | 422.7422 | |
| 4a-Formyl-5a-cholesta-8,24-dien-3b-ol | 7.53E-06 | 1.91E-05 | -0.3525 | 457.3312 | Steroids and steroid derivatives |
| Agavasaponin D | 2.34E-06 | 6.90E-06 | 0.319 | 596.2748 | Steroids and steroid derivatives |
| LysoPS(18:0/0:0) | 0.000212 | 0.000382 | 0.4086 | 570.2776 | Glycerophospholipids |
| Pantetheine 4'-phosphate | 5.34E-08 | 2.76E-07 | 0.3416 | 393.0642 | Carboxylic acids and derivatives |
| 17-Aminogeldanamycin | 1.58E-05 | 3.75E-05 | 0.4272 | 546.2767 | Macrolactams |
| Genkwanin | 0.002503 | 0.003657 | 0.5987 | 285.0757 | Flavonoids |
| Tetrahydrogeranylgeranyl-PP | 0.001839 | 0.002791 | -0.4135 | 472.1756 | Prenol lipids |
| Lysyltryptophan | 1.64E-08 | 1.06E-07 | -0.3618 | 353.1575 | Carboxylic acids and derivatives |
| 2-Deoxystreptamine | 5.55E-07 | 2.16E-06 | 0.4124 | 363.1661 | Organooxygen compounds |
| N-Palmitoyl Asparagine | 0.009568 | 0.01292 | 0.6683 | 241.017 | Organic sulfuric acids and derivatives |
| Prolyl-Lysine | 1.53E-09 | 1.60E-08 | 0.2931 | 288.156 | Carboxylic acids and derivatives |
| Cucurbic acid | 1.94E-08 | 1.38E-07 | -0.3893 | 177.1275 | Fatty Acyls |
| (S)-Nerolidol 3-O-[a-L-rhamnopyranosyl-(1->2)-b-D-glucopyranoside] | 4.21E-09 | 4.04E-08 | -0.3111 | 266.1625 | Fatty Acyls |

| | | | | | |
|---|---|---|---|---|---|
| Myxin | 0.007927 | 0.01086 | 0.6415 | 303.0617 | Diazanaphthalenes |
| PS(15:0/18:4(6Z,9Z,12Z,15Z)) | 1.55E-05 | 3.69E-05 | 0.3939 | 393.7213 | Glycerophospholipids |
| S-(1,2-Dicarboxyethyl)glutathione | 0.000409 | 0.000698 | 0.4574 | 404.0798 | Carboxylic acids and derivatives |
| Hoduloside X | 7.65E-13 | 6.07E-11 | 0.2981 | 558.2868 | Prenol lipids |
| Lopinavir | 1.36E-06 | 4.59E-06 | 0.3693 | 326.1818 | Carboxylic acids and derivatives |
| Scopoletin | 0.000731 | 0.001193 | 0.4607 | 237.0396 | Coumarins and derivatives |
| Niazirinin | 9.25E-07 | 3.09E-06 | 0.315 | 366.1186 | Organooxygen compounds |
| 4-Chloro-2-nitrobenzylalcohol | 0.001736 | 0.002614 | 0.4538 | 587.2858 | Benzoxazoles |
| 2-Polyprenyl-6-methoxy-1,4-benzoquinone | 3.68E-07 | 1.41E-06 | 0.3522 | 295.1293 | Prenol lipids |
| 3-methyl-4-pentenoic acid | 1.61E-05 | 3.81E-05 | -0.5051 | 115.0756 | |
| Des-arg(9)-bradykinin | 0.000778 | 0.001257 | 0.5111 | 452.7344 | Carboxylic acids and derivatives |
| Isokobusone | 2.02E-06 | 6.39E-06 | -0.42 | 264.1957 | Organooxygen compounds |
| Lysylisoleucine | 1.88E-07 | 8.73E-07 | 0.3466 | 260.1968 | Carboxylic acids and derivatives |
| Delta-Hydroxylysylnorleucine | 6.98E-09 | 5.33E-08 | 0.295 | 272.1612 | Carboxylic acids and derivatives |
| Ribostamycin | 9.24E-05 | 0.000181 | 0.4004 | 419.2135 | Organooxygen compounds |
| Tetrahydrothiophene-3-ol 1,1-dioxide | 9.10E-13 | 5.18E-11 | -0.3155 | 181.0166 | Thiolanes |
| Fe(II)-nicotianamine | 4.40E-07 | 1.64E-06 | 0.3698 | 302.135 | Carboxylic acids and derivatives |
| 2-Hydroxypropyl methacrylamide | 8.58E-10 | 1.02E-08 | -0.3568 | 188.0919 | Carboxylic acids and derivatives |
| Neuromedin B (1-3) | 6.02E-09 | 4.74E-08 | 0.3151 | 301.1511 | Carboxylic acids and derivatives |
| 2-(beta-D-Mannopyranosyl)-L-tryptophan | 1.96E-13 | 2.28E-11 | 0.2965 | 367.15 | Pyridines and derivatives |
| Quinagolida | 1.70E-07 | 7.29E-07 | 0.3262 | 430.1931 | Quinolines and derivatives |
| LysoPC(20:4(8Z,11Z,14Z,17Z)/0:0) | 0.000461 | 0.000778 | 0.3743 | 566.3214 | Glycerophospholipids |
| 2,3,4,5-Tetrahydro-2-pyridinecarboxylic acid | 1.47E-07 | 7.15E-07 | 0.3268 | 272.1603 | Carboxylic acids and derivatives |
| Capsaicin | 2.52E-08 | 1.69E-07 | 0.293 | 328.1865 | Phenols |
| Ile Leu Phe | 1.66E-07 | 7.87E-07 | 0.3505 | 392.254 | |
| Thr Ile Ile | 1.41E-11 | 4.69E-10 | 0.3612 | 346.2335 | |
| Oleanolic Acid | 1.44E-06 | 4.80E-06 | 0.2943 | 439.3575 | Prenol lipids |
| (S)-Reticuline | 3.37E-06 | 9.48E-06 | 0.3647 | 350.1351 | Isoquinolines and derivatives |
| Validamycin A | 7.39E-06 | 1.88E-05 | 0.3627 | 478.193 | Organooxygen compounds |
| (9S,10S)-10-hydroxy-9-(phosphonooxy)octadecanoate | 7.48E-07 | 2.78E-06 | 0.3511 | 419.2134 | Fatty Acyls |
| 7-Epi-12-hydroxyjasmonic acid | 2.65E-12 | 1.43E-10 | -0.2648 | 227.1278 | Fatty Acyls |
| Arg Leu | 7.77E-06 | 2.02E-05 | 0.3621 | 288.2028 | |
| Histidylarginine | 7.61E-07 | 2.61E-06 | 0.3168 | 332.1456 | Carboxylic acids and derivatives |
| PS(14:0/20:3(8Z,11Z,14Z)) | 5.99E-09 | 5.38E-08 | 0.3573 | 401.7357 | Glycerophospholipids |
| L-Histidine | 1.73E-08 | 1.25E-07 | 0.2825 | 156.0769 | Carboxylic acids and derivatives |
| 6''-O-Malonyldaidzin | 3.51E-14 | 6.23E-12 | 0.359 | 535.1443 | Coumarins and derivatives |
| Cis-3-Hexenyl lactate | 2.69E-10 | 4.02E-09 | -0.3129 | 217.1074 | Carboxylic acids and derivatives |
| PI(20:4(8Z,11Z,14Z,17Z)-2OH(5S,6R)/22:2(13Z,16Z)) | 2.34E-06 | 7.19E-06 | 0.4038 | 508.2744 | |
| Glycylphenylalanylleucylglycine | 9.84E-08 | 4.56E-07 | 0.3316 | 391.1977 | Carboxylic acids and derivatives |
| 7a-Hydroxydehydroepiandrosterone | 3.03E-08 | 1.96E-07 | -0.3525 | 327.1913 | Steroids and steroid derivatives |
| Mirogabalin | 1.60E-07 | 7.65E-07 | -0.3101 | 419.2909 | Carboxylic acids and derivatives |
| Ser Leu Leu | 2.61E-08 | 1.74E-07 | 0.3298 | 332.2178 | |
| His Ile Asp | 2.32E-06 | 7.15E-06 | 0.3626 | 384.1875 | |
| Comfrey | 0.001098 | 0.001735 | -0.4062 | 378.1946 | |

| | | | | | |
|---|---|---|---|---|---|
| Phoenicoxanthin | 6.83E-07 | 2.38E-06 | 0.2945 | 579.3884 | Prenol lipids |
| Thymidine | 1.22E-10 | 2.48E-09 | -0.3408 | 265.0818 | Pyrimidine nucleosides |
| Linatine | 3.07E-08 | 1.99E-07 | 0.343 | 301.1504 | Carboxylic acids and derivatives |
| 9-Oxo-nonanoic acid | 2.49E-10 | 4.29E-09 | -0.3614 | 214.1438 | Fatty Acyls |
| PC(LTE4/DiMe(11,5)) | 2.10E-05 | 4.80E-05 | 0.3595 | 528.2894 | |
| N-Oleoyl Histidine | 3.69E-05 | 7.90E-05 | 0.3389 | 437.3485 | Carboxylic acids and derivatives |
| Ser Ile Gln | 5.43E-09 | 4.96E-08 | 0.3472 | 347.1923 | |
| PA(22:6(4Z,7Z,10Z,12E,16Z,19Z)-OH(14)/13:0) | 3.81E-07 | 1.57E-06 | 0.3752 | 359.2104 | |
| Luteolin | 3.72E-05 | 7.93E-05 | 0.3787 | 321.0177 | Flavonoids |
| Gln Leu Phe | 4.48E-05 | 9.37E-05 | 0.3955 | 407.2287 | |
| Nordihydrocapsaicin | 3.37E-08 | 1.90E-07 | 0.299 | 314.1716 | Phenols |
| 5-Hydroxy-2,4-dimethylthiophen-3-one | 0.009555 | 0.0129 | 0.5719 | 165.0004 | Dihydrothiophenes |
| Solanolone | 6.71E-09 | 5.91E-08 | 0.353 | 292.1294 | Phenanthrenes and derivatives |
| 4-Bis(2-hydroxyethyl)amino-L-phenylalanine | 2.35E-06 | 7.21E-06 | -0.3679 | 269.1496 | Carboxylic acids and derivatives |
| Canavanine | 1.63E-09 | 1.68E-08 | -0.3246 | 211.0604 | Carboxylic acids and derivatives |
| (3R-(3alpha,4alpha(2R*,3R*),5beta,6beta))-5-Methoxy-4-(2-methyl-3-(3-methyl-2-butenyl)oxiranyl)-1-oxaspiro(2.5)octan-6-ol carbamate | 2.40E-10 | 3.67E-09 | 0.2989 | 346.1612 | Carboxylic acids and derivatives |
| PS(22:2(13Z,16Z)/PGD2) | 1.60E-05 | 3.78E-05 | 0.3572 | 478.7614 | |
| Gly Leu Leu | 2.58E-07 | 1.13E-06 | 0.2719 | 302.2073 | |
| Suloctidil | 3.54E-09 | 3.13E-08 | 0.2869 | 372.2131 | Benzene and substituted derivatives |
| N-Lauroyl Phenylalanine | 1.21E-08 | 8.26E-08 | -0.3128 | 368.2182 | Carboxylic acids and derivatives |
| Aclacinomycin N | 1.31E-06 | 4.46E-06 | 0.3356 | 846.3835 | Anthracyclines |
| Leucyl-Arginine | 1.60E-06 | 5.23E-06 | 0.3519 | 288.2027 | Carboxylic acids and derivatives |
| N(epsilon)-(carboxyethyl)lysine | 6.94E-07 | 2.62E-06 | 0.4202 | 201.1235 | Carboxylic acids and derivatives |
| LysoPE(0:0/20:4(8Z,11Z,14Z,17Z)) | 0.000124 | 0.000235 | 0.3323 | 546.2796 | Glycerophospholipids |
| 7-Acetylintermedine | 2.74E-08 | 1.81E-07 | -0.3633 | 355.1972 | |
| 7-a,25-Dihydroxycholesterol | 0.000336 | 0.000584 | 0.3357 | 439.3166 | Prenol lipids |
| His Gly Leu | 6.58E-06 | 1.76E-05 | 0.3479 | 326.1821 | |
| Ser Leu Val | 4.37E-08 | 2.63E-07 | 0.3369 | 318.2022 | |
| Asn Ile Leu | 6.43E-09 | 5.71E-08 | 0.2908 | 359.2286 | |
| Gibberellin A36 | 2.07E-08 | 1.28E-07 | 0.3087 | 361.165 | Prenol lipids |
| Beta-Funaltrexamine | 3.03E-06 | 8.99E-06 | 0.3731 | 472.2401 | Phenanthrenes and derivatives |
| Hypoglycin B | 7.66E-10 | 9.35E-09 | -0.3295 | 269.1139 | Carboxylic acids and derivatives |
| L-erythro-4-Hydroxyarginine | 8.76E-06 | 2.24E-05 | 0.3712 | 232.1404 | Carboxylic acids and derivatives |
| 7-Methyladenine | 8.36E-06 | 2.15E-05 | -0.4367 | 150.0777 | Imidazopyrimidines |
| (2-Mercaptomethyl-3-phenyl-propionyl)-glycine | 4.64E-07 | 1.85E-06 | -0.2723 | 276.0689 | Carboxylic acids and derivatives |
| LysoPE(0:0/22:6(4Z,7Z,10Z,13Z,16Z,19Z)) | 5.10E-05 | 0.000105 | 0.3333 | 589.3018 | Glycerophospholipids |
| Propyl gallate | 7.88E-10 | 1.05E-08 | -0.3293 | 230.1023 | Benzene and substituted derivatives |
| PE(20:2/0:0) | 7.31E-06 | 1.86E-05 | 0.3296 | 504.3081 | |
| Fluocortin butyl | 0.009685 | 0.01306 | 0.4869 | 445.2406 | Steroids and steroid derivatives |
| Galactosylglycerol | 2.96E-05 | 6.48E-05 | -0.416 | 254.1021 | Glycerolipids |
| Arginyl-Gamma-glutamate | 2.45E-07 | 1.09E-06 | 0.3521 | 668.3609 | Carboxylic acids and derivatives |
| PS(16:1(9Z)/20:4(8Z,11Z,14Z,17Z)-2OH(5S,6R)) | 0.000226 | 0.000406 | 0.4057 | 429.732 | |
| Ile Leu Lys | 5.74E-09 | 5.20E-08 | 0.2887 | 187.1442 | |

| | | | | | |
|---|---|---|---|---|---|
| Isoleucyl-Arginine | 0.02685 | 0.03327 | 0.9585 | 288.2025 | Carboxylic acids and derivatives |
| 1-Benzylpiperidine-4-carboxylic acid | 0.01164 | 0.01552 | 0.585 | 264.1236 | Piperidines |
| Combretastatin A4 | 4.28E-10 | 5.81E-09 | -0.2457 | 315.1233 | Stilbenes |
| (-)-Epiafzelechin | 6.53E-06 | 1.75E-05 | 0.3478 | 257.0808 | Flavonoids |
| O-Oxalylhomoserine | 2.01E-06 | 6.36E-06 | -0.3914 | 156.0292 | Carboxylic acids and derivatives |
| C-2 Ceramide | 7.03E-09 | 5.36E-08 | -0.3207 | 340.2848 | |
| PA(19:2(10Z,13Z)/20:5(5Z,8Z,10E,14Z,17Z)-OH(12)) | 0.000222 | 0.000398 | 0.4002 | 397.2261 | |
| (+)-Abscisic Acid | 5.72E-09 | 5.19E-08 | -0.3221 | 265.1435 | Prenol lipids |
| Glycyl-L-Tyrosine | 8.49E-07 | 2.87E-06 | 0.2732 | 237.0873 | Carboxylic acids and derivatives |
| Selamectin | 0.00108 | 0.001692 | 0.4159 | 407.7069 | Macrolides and analogues |
| (-)-Pinoresinol | 1.87E-07 | 8.69E-07 | 0.3445 | 562.2253 | Lignan glycosides |
| Cholylthreonine | 1.65E-07 | 7.84E-07 | 0.3361 | 509.3335 | |
| N-(3-Methyl-2-pyridyl)-3-phenylsuccinimide | 9.98E-05 | 0.000193 | 0.3967 | 299.139 | Pyrrolidines |
| N-(3-Hydroxypropyl)valine | 4.74E-11 | 1.17E-09 | -0.2958 | 176.1282 | Carboxylic acids and derivatives |
| 4,4'-Dihydroxyantipyrine | 3.20E-07 | 1.25E-06 | -0.2657 | 257.0331 | Azoles |
| Histidinyl-Leucine | 2.78E-07 | 1.20E-06 | 0.3649 | 269.1607 | Carboxylic acids and derivatives |
| (6E,8R,10Z)-8-hydroxy-3-oxohexadecadienoic acid | 1.32E-09 | 1.55E-08 | 0.3316 | 296.1967 | Fatty Acyls |
| 1-(beta-D-Glucopyranosyloxy)-3-octanone | 3.68E-12 | 1.79E-10 | 0.3063 | 345.1306 | Fatty Acyls |
| Dodeca-4,6,8-trienedioylcarnitine | 6.29E-08 | 3.16E-07 | 0.3037 | 388.1718 | Fatty Acyls |
| Gabexate | 0.000173 | 0.000318 | 0.3804 | 320.161 | Benzene and substituted derivatives |
| Serylisoleucine | 1.14E-06 | 3.70E-06 | 0.2769 | 217.1186 | Carboxylic acids and derivatives |
| Maltotetraose | 1.36E-07 | 6.01E-07 | 0.2421 | 701.1896 | Organooxygen compounds |
| Tazarotenic acid | 1.09E-06 | 3.83E-06 | -0.3108 | 346.0854 | Thiochromanes |
| Enterolactone | 3.17E-05 | 6.85E-05 | -0.2918 | 333.0894 | Furanoid lignans |
| 6-methyl-2-(2-morpholin-4-yl-2-oxoethyl)pyridazin-3-one | 4.92E-10 | 7.11E-09 | -0.299 | 238.1186 | |
| Hydratopyrrhoxanthinol | 2.34E-08 | 1.61E-07 | 0.2832 | 295.1814 | Prenol lipids |
| 1,7-Dimethylguanosine | 2.48E-08 | 1.68E-07 | -0.2713 | 276.1077 | Purine nucleosides |
| N(6)-(Octanoyl)lysine | 4.15E-05 | 8.75E-05 | 0.3329 | 336.228 | Carboxylic acids and derivatives |
| Ser Val Leu | 5.78E-09 | 5.23E-08 | 0.3024 | 318.2023 | |
| Syndyphalin-33 | 0.000942 | 0.001496 | 0.4366 | 467.2134 | Carboxylic acids and derivatives |
| Wharangin | 6.57E-10 | 8.22E-09 | -0.3263 | 325.0377 | Flavonoids |
| Safflomin C | 3.27E-05 | 7.09E-05 | 0.3459 | 597.1563 | Diarylheptanoids |
| CDP-DG(18:2(9Z,11Z)/i-17:0) | 1.32E-05 | 3.21E-05 | 0.3424 | 496.7742 | Glycerophospholipids |
| 3-(5'-methylthio)pentylmalate | 6.97E-06 | 1.85E-05 | -0.3381 | 266.1069 | Hydroxy acids and derivatives |
| 4a-Hydroxytetrahydrobiopterin | 1.47E-05 | 3.52E-05 | 0.3794 | 321.1291 | Pteridines and derivatives |
| 1-hydroxy-2-methyl-2-(E)-butenyl 4-diphosphate | 7.50E-06 | 1.91E-05 | -0.3555 | 303.9759 | Prenol lipids |
| Ineketone | 7.06E-10 | 8.71E-09 | 0.2409 | 363.217 | Prenol lipids |
| Ala Gly Leu Val Ser | 1.52E-07 | 6.61E-07 | 0.3248 | 444.2452 | |
| PE(22:6(5Z,8E,10Z,13Z,15E,19Z)-2OH(7S, 17S)/22:6(4Z,7Z,10Z,13Z,16Z,19Z)) | 8.89E-06 | 2.27E-05 | 0.3237 | 456.7427 | |
| Muramic acid | 5.24E-10 | 7.52E-09 | -0.3352 | 251.1026 | Organooxygen compounds |
| Glu Leu Leu | 4.86E-06 | 1.35E-05 | 0.35 | 374.2286 | |
| LysoPC(22:6(4Z,7Z,10Z,13Z,16Z,19Z)/0:0) | 0.000354 | 0.00061 | 0.298 | 568.3395 | Glycerophospholipids |
| (E)-Casimiroedine | 2.29E-05 | 5.17E-05 | -0.2962 | 417.1864 | Organooxygen compounds |
| Tyramine glucuronide | 4.50E-06 | 1.22E-05 | 0.3279 | 294.0979 | Organooxygen compounds |

| | | | | | |
|---|---|---|---|---|---|
| Diethylamine | 3.37E-07 | 1.42E-06 | -0.5008 | 74.09703 | Organonitrogen compounds |
| 5,6-epoxy,18R-HEPE | 3.88E-08 | 2.39E-07 | -0.3198 | 355.1866 | Fatty Acyls |
| 2-Methoxy-5-methylaniline | 4.93E-06 | 1.37E-05 | 0.368 | 292.2018 | Phenol ethers |
| Thr Ile Lys | 1.52E-07 | 7.34E-07 | 0.305 | 181.126 | |
| Bioadykinin Fragment 1-5 | 1.41E-07 | 6.91E-07 | 0.3015 | 586.3195 | Carboxylic acids and derivatives |
| 5,8-Dihydroxy-1,4-naphthoquinone | 5.68E-12 | 2.40E-10 | -0.2965 | 208.0605 | Naphthalenes |
| Phenmedipham | 3.79E-05 | 8.08E-05 | 0.4111 | 301.1181 | Benzene and substituted derivatives |
| L-Valine, N-(2-hydroxy-3-butenyl)- | 6.78E-13 | 5.46E-11 | 0.2457 | 229.1547 | Carboxylic acids and derivatives |
| Tryptophyl-Asparagine | 2.03E-07 | 8.45E-07 | 0.3096 | 317.1248 | Carboxylic acids and derivatives |
| L-L-Homoglutathione | 0.01042 | 0.01374 | 0.5529 | 339.1337 | Peptidomimetics |
| (S)-(-)-Perillyl alcohol | 1.28E-10 | 2.58E-09 | -0.2854 | 135.117 | Prenol lipids |
| (10Z,14E,16E)-10,14,16-Octadecatrien-12-ynoic acid | 3.59E-11 | 9.37E-10 | -0.2679 | 297.1807 | Fatty Acyls |
| N-Acetyl-N-[(2S)-3-(1H-indol-3-yl)-2-[[2-(4-piperidin-1-ylpiperidin-1-yl)acetyl]amino]propyl]-2-methoxybenzamide | 2.42E-05 | 5.42E-05 | -0.3419 | 591.3706 | Indoles and derivatives |
| Tobramycin | 3.42E-06 | 9.60E-06 | 0.318 | 488.2351 | Organooxygen compounds |
| 9-Oxodecanoylcarnitine | 1.19E-06 | 3.82E-06 | 0.3107 | 364.1872 | Fatty Acyls |
| Gamma-Glutamylasparagine | 6.10E-09 | 4.80E-08 | 0.2671 | 260.0882 | Carboxylic acids and derivatives |
| Oroselone | 2.43E-10 | 4.21E-09 | -0.3646 | 244.0968 | Coumarins and derivatives |
| (S)-(+)-1-(p-Hydroxy-trans-cinnamoyl)-glycerol | 2.09E-05 | 4.71E-05 | -0.3468 | 237.0758 | Cinnamic acids and derivatives |
| Deoxythioguanosine | 8.07E-13 | 6.35E-11 | 0.2727 | 248.0588 | Purine nucleosides |
| Mevalonic acid | 6.96E-07 | 2.42E-06 | 0.3103 | 443.213 | Fatty Acyls |
| (+/-)7-epi Jasmonic Acid | 1.60E-10 | 3.02E-09 | -0.3047 | 211.1329 | |
| Danazol | 1.08E-09 | 1.22E-08 | 0.2659 | 374.1559 | Steroids and steroid derivatives |
| Valtrate | 1.60E-09 | 1.82E-08 | 0.364 | 177.0547 | Prenol lipids |
| Ritipenem | 4.99E-05 | 0.000103 | 0.3506 | 287.0335 | Carboxylic acids and derivatives |
| Nodularin | 7.08E-05 | 0.000142 | 0.3456 | 789.4285 | Carboxylic acids and derivatives |
| N1,N10-Dicoumaroylspermidine | 7.40E-07 | 2.54E-06 | 0.2474 | 436.2232 | Cinnamic acids and derivatives |
| Gly Leu Asp | 1.69E-05 | 3.96E-05 | 0.3243 | 304.1501 | |
| N-Acetyl-b-D-galactosamine | 2.73E-07 | 1.09E-06 | -0.2539 | 202.0712 | Organooxygen compounds |
| Phenylalanylproline | 1.41E-10 | 2.78E-09 | -0.2437 | 295.1651 | Carboxylic acids and derivatives |
| N-(8-Amino-1-carboxyoctyl)-alanyl-proline | 7.93E-06 | 2.05E-05 | 0.3543 | 399.26 | Purine nucleosides |
| Gln Val Val | 1.43E-09 | 1.66E-08 | 0.3001 | 345.2133 | |
| Lactucin | 1.09E-08 | 7.56E-08 | 0.2829 | 257.0814 | Lactones |
| LysoPC(20:5(5Z,8Z,11Z,14Z,17Z)/0:0) | 3.28E-05 | 7.12E-05 | 0.3406 | 542.3237 | Glycerophospholipids |
| 3-Methyl-2-oxovaleric acid | 1.48E-05 | 3.49E-05 | 0.3195 | 389.1822 | Keto acids and derivatives |
| N-(L-Arginino)succinate | 1.36E-06 | 4.59E-06 | 0.298 | 291.1297 | Carboxylic acids and derivatives |
| Epigoitrin | 3.04E-07 | 1.19E-06 | 0.3531 | 128.0164 | Azolidines |
| Tryptophyl-Serine | 3.24E-06 | 9.16E-06 | 0.3218 | 290.1142 | Carboxylic acids and derivatives |
| Leu Gly Phe | 1.25E-06 | 4.27E-06 | 0.3216 | 336.1915 | |
| Alpha-dihydroartemisinin | 4.91E-08 | 2.58E-07 | -0.2919 | 283.1546 | Prenol lipids |
| Gln Val Ile | 1.32E-06 | 4.47E-06 | 0.3086 | 359.2287 | |
| Lotaustralin | 1.28E-07 | 6.35E-07 | 0.3109 | 325.1392 | Organooxygen compounds |
| Val Tyr | 7.79E-06 | 2.03E-05 | -0.3317 | 281.1495 | |
| Orotic Acid | 6.15E-08 | 3.11E-07 | -0.2534 | 155.0087 | Diazines |

| | | | | | |
|---|---|---|---|---|---|
| Asparaginyl-Leucine | 5.33E-09 | 4.33E-08 | 0.2783 | 244.1297 | Carboxylic acids and derivatives |
| 13-L-Hydroperoxylinoleic acid | 7.10E-08 | 3.47E-07 | -0.2225 | 311.2221 | Fatty Acyls |
| Manzamine A | 2.87E-08 | 1.67E-07 | 0.2288 | 569.3316 | Harmala alkaloids |
| 14-oxo-DoHE(1-) | 1.67E-07 | 7.92E-07 | -0.3348 | 381.1843 | Fatty Acyls |
| 4-Oxo-1,4-dihydroquinoline-3-carboxylic acid | 7.49E-10 | 1.00E-08 | -0.2173 | 190.05 | Quinolines and derivatives |
| Alpha-Trisaccharide | 0.001508 | 0.002298 | -0.314 | 579.2395 | Organooxygen compounds |
| Glycyl-prolyl-glutamic acid | 1.12E-09 | 1.25E-08 | 0.2579 | 300.1194 | Carboxylic acids and derivatives |
| 19-Benzyl-7-hydroxy-7,9,16,17-tetramethyl-2,4,15-trioxa-20-azatetracyclo[11.8.0.01,18.014,16]henicosa-5,11-diene-3,8,21-trione | 4.53E-06 | 1.27E-05 | 0.3185 | 518.2188 | Cytochalasans |
| Practolol | 1.53E-09 | 1.75E-08 | -0.2545 | 555.3177 | Benzene and substituted derivatives |
| (2S)-2-Amino-3-[(2S,3R)-2-amino-3-[(2S)-2-amino-3-(4-hydroxyphenyl)propanoyl]oxybutanoyl]oxypropanoic acid | 3.24E-10 | 5.25E-09 | 0.3214 | 387.1873 | Carboxylic acids and derivatives |
| Methoxamine | 2.74E-07 | 1.19E-06 | 0.3208 | 234.1084 | Benzene and substituted derivatives |
| Glu Val Glu | 1.20E-07 | 6.04E-07 | 0.2964 | 376.1717 | |
| Aspartylglycosamine | 7.47E-06 | 1.96E-05 | 0.3499 | 318.1294 | Organooxygen compounds |
| Estrone | 1.19E-09 | 1.31E-08 | 0.2744 | 599.3398 | Steroids and steroid derivatives |
| (23S,24S)-17,23-Epoxy-24,29-dihydroxy-27-norlanost-8-ene-3,15-dione | 2.34E-07 | 1.05E-06 | -0.2615 | 455.315 | Prenol lipids |
| Nonate | 1.79E-07 | 8.36E-07 | -0.3463 | 230.1387 | Fatty Acyls |
| PE(18:3(9Z,12Z,15Z)/TXB2) | 0.000213 | 0.000385 | 0.3659 | 436.7345 | |
| Tramadol | 1.86E-10 | 3.44E-09 | 0.2639 | 286.1761 | Phenol ethers |
| 2-Azetidinecarboxylic acid, 3-(3-((aminoiminomethyl)amino)propyl)-1-((4-(((1,1-dimethylethyl)amino)carbonyl)-1-piperazinyl)carbonyl)-4-oxo-, (2S,3R)- | 8.17E-07 | 2.77E-06 | 0.293 | 460.2039 | Lactams |
| Val Phe Phe | 1.13E-06 | 3.93E-06 | 0.3374 | 412.2227 | |
| 5-(N-Hexadecanoyl)aminofluorescin | 0.000691 | 0.001134 | 0.367 | 630.3089 | Benzopyrans |
| Vindoline | 2.19E-06 | 6.81E-06 | 0.3186 | 474.2559 | Plumeran-type alkaloids |
| 5-Hydroxyindoleacetylglycine | 8.53E-07 | 3.09E-06 | 0.2898 | 281.1131 | Carboxylic acids and derivatives |
| Ser Val Gln Leu Leu | 6.29E-07 | 2.41E-06 | 0.3014 | 559.3447 | |
| Oxandrolone | 4.11E-10 | 6.26E-09 | -0.2557 | 370.2334 | Steroids and steroid derivatives |
| Gly Leu Gln | 2.36E-06 | 7.24E-06 | 0.2761 | 317.1818 | |
| 18-hydroxyoleate | 3.10E-06 | 9.16E-06 | -0.2546 | 617.4747 | Fatty Acyls |
| 6-Ethylchenodeoxycholic acid | 4.59E-07 | 1.70E-06 | -0.2754 | 441.2986 | Steroids and steroid derivatives |
| Ile Lys | 9.82E-05 | 0.000191 | 0.3586 | 260.1968 | |
| N-[(3a,5b,7a,12a)-3,7-dihydroxy-24-oxo-12-(sulfooxy)cholan-24-yl]-Glycine | 0.000353 | 0.000611 | 0.3462 | 544.2609 | Steroids and steroid derivatives |
| Alpha-Cyperol | 6.84E-07 | 2.58E-06 | 0.3249 | 203.1796 | Prenol lipids |
| Turanose | 1.93E-08 | 1.21E-07 | 0.2719 | 323.0977 | Fatty Acyls |
| (9E)-Valenciaxanthin | 2.11E-05 | 4.80E-05 | 0.3209 | 457.2656 | Prenol lipids |
| Tuliposide A | 9.64E-05 | 0.000188 | -0.3514 | 261.0964 | Organooxygen compounds |
| Deoxycholic acid glycine conjugate | 3.42E-05 | 7.33E-05 | -0.2688 | 448.3054 | Steroids and steroid derivatives |
| Isopropamide | 1.28E-05 | 3.11E-05 | -0.3223 | 336.2531 | Benzene and substituted derivatives |
| 3'-O-Methylinosine | 2.11E-07 | 8.74E-07 | -0.2643 | 281.0886 | |
| Assamsaponin E | 5.45E-05 | 0.000112 | 0.3164 | 620.296 | Prenol lipids |
| Leu Val Leu | 2.07E-06 | 6.51E-06 | 0.3308 | 344.2541 | |
| (2S)-2-Amino-6-[(3-formylpiperidin-1-yl)amino]hexanoic acid | 6.90E-09 | 5.29E-08 | 0.2629 | 302.1715 | Carboxylic acids and derivatives |
| Glutaminyltyrosine | 1.30E-11 | 3.73E-10 | 0.2937 | 308.1247 | Carboxylic acids and derivatives |

| | | | | | |
|---|---|---|---|---|---|
| Threoninyl-Valine | 7.31E-06 | 1.92E-05 | 0.3022 | 500.2713 | Carboxylic acids and derivatives |
| Ser Ile Met | 1.40E-05 | 3.38E-05 | 0.3427 | 350.1741 | |
| (S)-Tetrahydrocolumbamine | 0.01629 | 0.02084 | 0.62 | 306.1487 | Protoberberine alkaloids and derivatives |
| 10-Hydroxy-2-oxabicyclo[6.2.2]dodeca-1(10),8,11-trien-3-one | 3.08E-09 | 3.13E-08 | -0.2916 | 210.1125 | Phenols |
| Maysin 3'-methyl ether | 7.93E-06 | 2.00E-05 | 0.2748 | 611.1356 | Flavonoids |
| Ganoderic acid xi | 0.000353 | 0.000611 | 0.3187 | 559.2874 | Prenol lipids |
| Alliospiroside C | 6.17E-06 | 1.66E-05 | 0.2706 | 769.3724 | Steroids and steroid derivatives |
| Levobunolol | 2.42E-11 | 7.12E-10 | -0.2951 | 324.2167 | Tetralins |
| Glutamyltryptophan | 3.37E-11 | 7.76E-10 | 0.2284 | 332.1246 | Carboxylic acids and derivatives |
| Serylvalylglycylglutamic acid | 2.15E-09 | 2.32E-08 | 0.2632 | 432.2089 | Carboxylic acids and derivatives |
| Coniferyl acetate | 3.15E-06 | 8.93E-06 | -0.2907 | 267.0869 | Phenols |
| Spirolide B | 0.001134 | 0.001769 | 0.3339 | 358.731 | Azepines |
| His Trp | 8.23E-11 | 1.81E-09 | 0.2542 | 342.1563 | |
| TRIMAZOSIN | 5.12E-05 | 0.000106 | 0.3141 | 468.2488 | Diazinanes |
| N6-Methyl-2'-deoxyadenosine | 2.47E-08 | 1.67E-07 | -0.2823 | 248.1143 | Purine nucleosides |
| Xanthoangelol H | 1.48E-05 | 3.54E-05 | 0.2902 | 319.132 | Cinnamic acids and derivatives |
| Fexinidazole | 2.84E-08 | 1.85E-07 | -0.3529 | 312.1011 | Azoles |
| Cadabicine | 3.46E-06 | 1.01E-05 | -0.2934 | 468.2489 | Organooxygen compounds |
| 2,3-Dihydroxycarbamazepine | 2.87E-08 | 1.67E-07 | 0.2763 | 305.0331 | Benzazepines |
| Asp Ala Ile | 3.20E-06 | 9.43E-06 | 0.3009 | 318.1658 | |
| 4-Hydroxyestradiol | 4.74E-10 | 6.92E-09 | -0.2888 | 311.1599 | Steroids and steroid derivatives |
| Alpha-Elemolic acid | 7.21E-09 | 6.27E-08 | 0.2694 | 439.3564 | Prenol lipids |
| Acacetin | 0.004486 | 0.006268 | 0.4474 | 285.0756 | |
| Ala Phe Leu | 4.13E-06 | 1.17E-05 | 0.3223 | 350.2072 | |
| Sarmentosin | 0.001478 | 0.002279 | -0.2627 | 274.0927 | Fatty Acyls |
| Domoic acid | 1.64E-08 | 1.06E-07 | 0.2668 | 332.1093 | Carboxylic acids and derivatives |
| Lycoperdic acid | 1.51E-10 | 2.53E-09 | 0.2217 | 262.0563 | Carboxylic acids and derivatives |
| Indicaxanthin | 0.01579 | 0.02025 | 0.5032 | 309.108 | Carboxylic acids and derivatives |
| L-Asparagine | 2.59E-05 | 5.72E-05 | 0.2668 | 131.045 | Carboxylic acids and derivatives |
| Gly Trp Leu | 2.20E-07 | 9.95E-07 | 0.2941 | 375.2024 | |
| 4-Hydroxy-3-methyl-2-(2-propynyl)-2-cyclopentene-1-one | 5.66E-08 | 2.89E-07 | -0.3067 | 195.0654 | Organooxygen compounds |
| N-Succinyl-L,L-2,6-diaminopimelate | 2.43E-08 | 1.66E-07 | 0.2579 | 332.145 | Carboxylic acids and derivatives |
| Gamma-Glutamylphenylalanine | 2.15E-09 | 2.11E-08 | 0.2451 | 293.1138 | Carboxylic acids and derivatives |
| KOBUSONE | 7.74E-09 | 6.63E-08 | -0.2562 | 223.1693 | Organooxygen compounds |
| Dibenzo-P-dioxin | 4.88E-06 | 1.32E-05 | 0.3021 | 423.1513 | Stilbenes |
| 4-Hydroxy-5-phenyltetrahydro-1,3-oxazin-2-one | 1.21E-08 | 9.43E-08 | -0.2399 | 226.1075 | Benzene and substituted derivatives |
| Leu Lys | 5.77E-08 | 3.29E-07 | 0.2931 | 260.1968 | |
| Leu Ser Ile | 2.38E-08 | 1.63E-07 | 0.2417 | 332.2177 | |
| Methyldopate | 1.05E-07 | 5.45E-07 | 0.2654 | 262.1032 | Phenylpropanoic acids |
| N1-(5-Phospho-a-D-ribosyl)-5,6-dimethylbenzimidazole | 9.81E-05 | 0.00019 | 0.3351 | 393.0641 | Benzimidazole ribonucleosides and ribonucleotides |
| Moperone | 3.39E-11 | 7.80E-10 | 0.2731 | 392.1455 | Organooxygen compounds |
| PGP(i-19:0/6 keto-PGF1alpha) | 3.42E-07 | 1.43E-06 | 0.2373 | 491.2611 | |

| | | | | | |
|---|---|---|---|---|---|
| S-(3-Methylbutanoyl)-dihydrolipoamide-E | 1.86E-05 | 4.31E-05 | 0.2858 | 291.1338 | Fatty Acyls |
| 6-Hydroxynicotinic Acid | 7.56E-07 | 2.80E-06 | -0.3072 | 140.0344 | Pyridines and derivatives |
| 1-{[(Z)-Benzylidene(oxido)-lambda~5~-azanyl]methyl}cyclohexanol | 7.97E-09 | 6.79E-08 | -0.2923 | 234.1489 | |
| Asn Leu Leu | 2.04E-08 | 1.44E-07 | 0.2595 | 359.2286 | |
| Anonaine | 0.004225 | 0.006039 | 0.4405 | 310.108 | Aporphines |
| Deoxyloganic acid | 5.10E-06 | 1.37E-05 | 0.301 | 405.1406 | Prenol lipids |
| Ovalicin | 5.02E-06 | 1.39E-05 | -0.3081 | 296.1604 | Organooxygen compounds |
| Fasoracetam | 2.89E-08 | 1.67E-07 | 0.2377 | 241.1187 | Carboxylic acids and derivatives |
| Contignasterol | 9.96E-08 | 5.17E-07 | -0.2188 | 473.3256 | Prenol lipids |
| 9,10-DHOME | 1.06E-08 | 8.50E-08 | -0.2478 | 297.2423 | Fatty Acyls |
| 4-Hydroxy-3-(3-methyl-2-butenyl)acetophenone | 3.20E-12 | 1.61E-10 | -0.3079 | 237.1485 | Organooxygen compounds |
| Carglumic acid | 6.87E-07 | 2.39E-06 | 0.2683 | 189.0506 | Carboxylic acids and derivatives |
| 11-Hydroxyyohimbine | 0.002044 | 0.003033 | 0.438 | 335.1752 | Yohimbine alkaloids |
| Lithocholate 3-O-glucuronide | 1.39E-06 | 4.38E-06 | 0.2363 | 551.3213 | Steroids and steroid derivatives |
| Maltotriose | 7.53E-08 | 3.64E-07 | 0.2041 | 539.137 | Organooxygen compounds |
| 5-methoxy-6-(2-propenyl)-1,3-benzodioxole | 7.11E-08 | 3.48E-07 | -0.3015 | 237.0759 | Benzodioxoles |
| Leu Arg | 6.10E-09 | 5.45E-08 | 0.3192 | 288.2028 | |
| Asp Ile Asn | 1.92E-05 | 4.44E-05 | 0.2832 | 361.1715 | |
| (R)-2-Benzylsuccinate | 3.84E-09 | 3.77E-08 | -0.3047 | 226.1074 | Phenylpropanoic acids |
| N-Nitrosoproline | 5.84E-09 | 4.64E-08 | 0.2668 | 165.0295 | Carboxylic acids and derivatives |
| Gly Val Ile | 1.55E-08 | 1.15E-07 | 0.2412 | 288.1916 | |
| 8-Methoxykynurenate | 4.57E-08 | 2.74E-07 | -0.2793 | 261.0872 | Quinolines and derivatives |
| 4-Ipomeanol | 7.78E-11 | 1.72E-09 | -0.2368 | 210.1125 | Organooxygen compounds |
| DL-2-Aminooctanoic acid | 1.02E-08 | 8.26E-08 | -0.3399 | 160.1333 | Carboxylic acids and derivatives |
| Cinncassiol D2 glucoside | 6.82E-05 | 0.000137 | 0.3342 | 548.3107 | Prenol lipids |
| Phenylalanylthreonine | 1.21E-09 | 1.33E-08 | 0.265 | 531.2409 | Carboxylic acids and derivatives |
| 1,5-Anhydro-d-mannitol | 9.42E-07 | 3.37E-06 | -0.3069 | 206.1023 | Organooxygen compounds |
| Cytidylyl-(3',5')-guanosine | 0.000226 | 0.000405 | 0.2827 | 587.1244 | (3''->5'')-dinucleotides and analogues |
| Vanillic acid | 0.000781 | 0.001266 | 0.3122 | 167.0339 | Benzene and substituted derivatives |
| CDP-DG(18:2(9Z,11Z)/a-15:0) | 2.72E-06 | 8.18E-06 | 0.296 | 482.7583 | Glycerophospholipids |
| 2-Furanmethanol | 3.99E-07 | 1.63E-06 | -0.3119 | 238.1074 | Heteroaromatic compounds |
| Pro-Pro-Pro | 1.79E-08 | 1.29E-07 | 0.199 | 310.1759 | Carboxylic acids and derivatives |
| Aniracetam | 3.11E-08 | 2.00E-07 | -0.3133 | 261.1237 | Benzene and substituted derivatives |
| Taxine B | 0.001938 | 0.00289 | 0.3277 | 548.2955 | Prenol lipids |
| 1-Aminocyclopropanecarboxylic acid | 5.29E-08 | 2.74E-07 | 0.2756 | 302.1351 | Carboxylic acids and derivatives |
| 3-[4-Hydroxy-3-(3-methyl-2-butenyl)phenyl]-2-propenal | 9.78E-07 | 3.48E-06 | 0.2696 | 234.1489 | Cinnamaldehydes |
| Opiorphin | 0.000241 | 0.00043 | -0.3293 | 693.3814 | Carboxylic acids and derivatives |
| Gly Val Val | 4.37E-10 | 6.54E-09 | 0.2379 | 274.1761 | |
| Unknown 370 | 9.08E-07 | 3.04E-06 | -0.2275 | 457.2947 | Prenol lipids |
| 6''-O-Acetylglycitin | 3.50E-12 | 1.45E-10 | 0.2466 | 533.1285 | Isoflavonoids |
| Hydroxypelenolide | 5.86E-09 | 5.29E-08 | -0.222 | 235.1692 | Prenol lipids |
| APGPR Enterostatin | 1.41E-05 | 3.41E-05 | 0.2996 | 461.2603 | Benzene and substituted derivatives |
| Fenvalerate | 0.006013 | 0.008226 | 0.3335 | 384.1148 | Fatty Acyls |
| Pseudouridine | 8.90E-09 | 7.39E-08 | -0.2316 | 245.0768 | Nucleoside and nucleotide analogues |

| | | | | | |
|---|---|---|---|---|---|
| N-(1,3-Dihydroxyoctadec-4-en-2-yl)acetamide | 1.68E-05 | 3.95E-05 | -0.2622 | 405.3111 | Sphingolipids |
| Herierin III | 1.63E-12 | 1.02E-10 | -0.2645 | 212.0918 | Pyrans |
| Asparaginylaspartic acid | 2.20E-10 | 3.42E-09 | 0.2793 | 246.0725 | Carboxylic acids and derivatives |
| 5-Heptyltetrahydro-2-oxo-3-furancarboxylic acid | 9.43E-10 | 1.10E-08 | -0.2179 | 227.1282 | Lactones |
| Vanillylmandelic acid | 7.53E-07 | 2.79E-06 | 0.326 | 181.0497 | Phenol ethers |
| Alanylthreonine | 1.24E-07 | 6.21E-07 | -0.3312 | 155.0816 | Carboxylic acids and derivatives |
| Riddelliine | 1.20E-06 | 3.85E-06 | -0.2503 | 386.1025 | |
| O-Demethylencainide | 1.61E-08 | 1.05E-07 | 0.2336 | 373.1721 | Benzene and substituted derivatives |
| Coclaurine | 0.000416 | 0.00071 | -0.2962 | 284.1287 | Isoquinolines and derivatives |
| Malonic semialdehyde | 9.75E-07 | 3.24E-06 | -0.2031 | 235.0452 | Organooxygen compounds |
| Serylvaline | 2.37E-05 | 5.29E-05 | 0.2788 | 203.1028 | Carboxylic acids and derivatives |
| Uridine, 2'-deoxy-2'-fluoro-2'-methyl-, (2'R)- | 3.34E-12 | 1.39E-10 | 0.241 | 305.0774 | Pyrimidine nucleosides |
| 4-Hydroxycyclohexylcarboxylic acid | 4.23E-12 | 1.68E-10 | -0.2624 | 189.0759 | Organooxygen compounds |
| Ala Ala Phe | 4.96E-06 | 1.38E-05 | 0.324 | 308.1597 | |
| 27-Deoxy-5b-cyprinol | 1.52E-06 | 4.73E-06 | -0.2653 | 457.3312 | Steroids and steroid derivatives |
| Ser Pro Leu | 4.03E-06 | 1.15E-05 | 0.2668 | 316.1863 | |
| N-[(1R)-2,3-Dihydro-1H-inden-1-yl]-adenosine | 3.56E-06 | 1.03E-05 | -0.257 | 366.1594 | Purine nucleosides |
| N2-gamma-L-Glutamyl-L-arginine | 6.16E-06 | 1.60E-05 | 0.2865 | 302.1463 | Carboxylic acids and derivatives |
| Adrenic acid | 9.76E-06 | 2.41E-05 | -0.2491 | 377.2688 | Fatty Acyls |
| Helenalin | 1.51E-08 | 1.13E-07 | -0.2868 | 263.1277 | Prenol lipids |
| Adenine | 5.22E-06 | 1.44E-05 | -0.2777 | 136.0619 | Imidazopyrimidines |
| Arg Ala Leu | 7.11E-05 | 0.000142 | 0.2878 | 359.2399 | |
| Wedelolactone | 1.61E-05 | 3.75E-05 | -0.269 | 359.043 | Isoflavonoids |
| (5Z,7S,8E,10Z,13Z,15E,17S,19Z)-7,17-Dihydroxydocosa-5,8,10,13,15,19-hexaenoylcarnitine | 5.72E-07 | 2.22E-06 | 0.2992 | 542.2892 | Fatty Acyls |
| Milbemycin A3 | 1.62E-08 | 1.19E-07 | -0.2447 | 511.3029 | Macrolides and analogues |
| Poncirin | 1.24E-06 | 3.97E-06 | 0.2351 | 615.176 | Flavonoids |
| 3-Amino-5-hydroxybenzoic acid | 6.39E-12 | 2.62E-10 | -0.2685 | 154.05 | Benzene and substituted derivatives |
| Histidylleucine | 1.47E-07 | 7.15E-07 | 0.22 | 269.1607 | Carboxylic acids and derivatives |
| Ala Leu Gln | 0.000164 | 0.000303 | 0.3071 | 331.1973 | |
| 2-Methoxy-5-(2,4-dioxo-5-thiazolidinyl)-N-((4-(trifluoromethyl)phenyl)methyl)benzamide | 4.14E-08 | 2.26E-07 | -0.2411 | 403.09 | Benzene and substituted derivatives |
| Glutaminylvaline | 1.40E-05 | 3.33E-05 | 0.2836 | 244.1296 | Carboxylic acids and derivatives |
| Aspartyl-Proline | 5.24E-08 | 3.05E-07 | -0.2228 | 213.087 | Carboxylic acids and derivatives |
| N-Formyl-met-leu-phe-lys | 6.75E-06 | 1.74E-05 | 0.2782 | 546.2764 | Carboxylic acids and derivatives |
| Calystegin A3 | 2.48E-08 | 1.68E-07 | 0.2559 | 360.2127 | Tropane alkaloids |
| 9-Oxononanoylcarnitine | 7.47E-07 | 2.57E-06 | 0.2777 | 352.1507 | Fatty Acyls |
| Scymnol | 1.44E-07 | 7.03E-07 | -0.2421 | 433.3309 | Steroids and steroid derivatives |
| Hydantoin-5-propionic acid | 1.65E-09 | 1.85E-08 | -0.2844 | 155.0451 | Azolidines |
| N-Docosahexaenoyl Asparagine | 0.003154 | 0.004532 | 0.4204 | 484.3127 | |
| PE(18:1(9Z)/0:0) | 3.63E-05 | 7.75E-05 | 0.2034 | 478.2925 | |
| Tumonoic Acid E | 0.000124 | 0.000235 | 0.3002 | 326.2322 | |
| 5-Hydroxypentanoic acid | 1.26E-10 | 2.55E-09 | -0.2548 | 278.1597 | Fatty Acyls |
| Licoricidin | 0.006538 | 0.008899 | 0.4087 | 469.1966 | Isoflavonoids |

| | | | | | |
|---|---|---|---|---|---|
| 3-O-beta-D-glucosyl-brassicasterol | 1.85E-05 | 4.24E-05 | -0.2666 | 559.3987 | Steroids and steroid derivatives |
| Aflatoxin B2 | 1.73E-08 | 1.11E-07 | -0.2589 | 359.0799 | Coumarins and derivatives |
| Baptifoline | 6.31E-08 | 3.16E-07 | -0.2662 | 295.1214 | Lupin alkaloids |
| Fenoprofen | 9.10E-09 | 6.56E-08 | -0.2524 | 263.0667 | Benzene and substituted derivatives |
| Benzyl alcohol beta-D-rutinoside | 2.95E-08 | 1.92E-07 | 0.2445 | 209.0921 | Organooxygen compounds |
| Apigenin 7,4'-dimethyl ether | 5.63E-06 | 1.54E-05 | -0.2716 | 299.0913 | Flavonoids |
| Methylphenidate | 0.000747 | 0.001211 | -0.3666 | 216.1383 | Organonitrogen compounds |
| Tryptophyl-Arginine | 2.81E-07 | 1.21E-06 | -0.2362 | 325.1758 | Carboxylic acids and derivatives |
| 4-Hydroxy-2H-pyran-3-carboxaldehyde | 0.000123 | 0.000233 | -0.3065 | 311.0769 | Pyrans |
| Valylglutamic acid | 8.09E-06 | 2.09E-05 | -0.3056 | 310.1394 | Carboxylic acids and derivatives |
| Miserotoxin | 4.99E-08 | 2.61E-07 | 0.2683 | 312.093 | Organooxygen compounds |
| Formiminoglutamic acid | 0.000106 | 0.000204 | 0.3324 | 207.0976 | Carboxylic acids and derivatives |
| PE(20:3(5Z,8Z,11Z)/18:1(12Z)-2OH(9,10)) | 4.25E-06 | 1.20E-05 | 0.2607 | 422.757 | |
| Ile Gly Lys | 2.75E-07 | 1.19E-06 | 0.2385 | 317.2181 | |
| 2H-1,4-Benzodiazepin-2-one, 1,3-dihydro-7-chloro-5-(2-fluorophenyl)-1-(2-hydroxyethyl)- | 1.18E-06 | 3.80E-06 | 0.2388 | 377.0693 | Benzodiazepines |
| Quassinol | 1.42E-07 | 6.94E-07 | 0.2701 | 424.1711 | Prenol lipids |
| 12-Oxo-2,3-dinor-10,15-phytodienoic acid | 0.000252 | 0.000449 | -0.3146 | 247.1693 | Fatty Acyls |
| Biotin | 5.12E-11 | 1.08E-09 | -0.2498 | 243.0804 | Biotin and derivatives |
| Hydroxyprolyl-Lysine | 5.64E-06 | 1.49E-05 | 0.2813 | 258.1454 | Carboxylic acids and derivatives |
| 10-Hydroxymelleolide | 2.37E-09 | 2.26E-08 | -0.2331 | 397.1675 | Prenol lipids |
| 4-{[1-(2,6-dimethylphenoxy)propan-2-yl]amino}-4-Oxobutanoic Acid | 3.35E-09 | 3.37E-08 | -0.2706 | 279.1453 | |
| Actinodaphnine | 4.54E-08 | 2.72E-07 | 0.2536 | 329.1494 | Aporphines |
| 3-Carboxy-4-methyl-5-ethyl-2-furanpropionic acid | 1.05E-06 | 3.45E-06 | 0.273 | 225.076 | Fatty Acyls |
| Metipranolol | 3.10E-07 | 1.21E-06 | 0.236 | 330.1664 | Phenol esters |
| Phenylalanylaspartic acid | 2.74E-10 | 4.67E-09 | 0.2412 | 281.1131 | Carboxylic acids and derivatives |
| 7-Hexadecynoic acid | 1.04E-08 | 8.38E-08 | -0.2615 | 297.1808 | Fatty Acyls |
| 2-(5-Hydroxy-1H-indol-2-yl)acetic acid | 6.04E-07 | 2.32E-06 | -0.2985 | 224.0912 | Indoles and derivatives |
| 2-Aminobenzylstatine | 1.38E-06 | 4.64E-06 | -0.2807 | 298.2125 | Carboxylic acids and derivatives |
| Asp Leu Lys | 1.55E-05 | 3.69E-05 | 0.2593 | 375.2238 | |
| Cinaciguat | 2.71E-06 | 8.17E-06 | -0.2524 | 607.3119 | Stilbenes |
| 5-Hydroxytryptophol sulfate | 0.002079 | 0.003131 | 0.3464 | 292.979 | Organic sulfuric acids and derivatives |
| Lomefloxacin | 3.10E-05 | 6.72E-05 | 0.257 | 350.135 | Lactams |
| Azacitidine | 1.03E-07 | 5.36E-07 | -0.2374 | 506.192 | Organooxygen compounds |
| Gentamicin B | 9.16E-05 | 0.000179 | 0.3396 | 447.2443 | Organooxygen compounds |
| Thr Asp Leu | 1.12E-06 | 3.92E-06 | 0.2476 | 348.1763 | |
| Doisynoestrol | 1.18E-08 | 8.07E-08 | -0.2401 | 319.1294 | Steroids and steroid derivatives |
| Cl-amidine | 2.06E-06 | 6.48E-06 | 0.2744 | 621.251 | Benzene and substituted derivatives |
| Asp Leu | 7.29E-11 | 1.63E-09 | 0.2338 | 247.1289 | |
| Modithromycin | 3.28E-06 | 9.64E-06 | 0.2562 | 432.229 | Organooxygen compounds |
| N-Oleoyl Asparagine | 6.63E-05 | 0.000133 | 0.3077 | 395.2905 | Carboxylic acids and derivatives |
| (3S,8R,9S,10R,13S,14S)-10,13-Dimethyl-17-pyrimidin-5-yl-2,3,4,7,8,9,11,12,14,15-decahydro-1H-cyclopenta[a]phenanthren-3-ol | 5.83E-08 | 2.97E-07 | 0.2249 | 387.1878 | Steroids and steroid derivatives |

| | | | | | |
|---|---|---|---|---|---|
| Penicillin G | 0.00557 | 0.007662 | -0.3022 | 299.0847 | Carboxylic acids and derivatives |
| LysoPE(18:1(11Z)/0:0) | 3.81E-05 | 8.13E-05 | 0.2252 | 480.3084 | Glycerophospholipids |
| 4-Amino-2-methylenebutanoic acid | 7.68E-08 | 3.69E-07 | 0.2234 | 344.182 | Carboxylic acids and derivatives |
| 3,5,7,8-Tetrahydro-2-[4-(trifluoromethyl)phenyl]-4H-thiopyrano[4,3-d]pyrimidin-4-one | 0.000928 | 0.001484 | 0.3209 | 349.0016 | Pyranopyridines |
| 7-Methylthioheptyl Glucosinolate | 6.03E-06 | 1.57E-05 | -0.2147 | 462.0911 | Organooxygen compounds |
| Falcarindiol | 4.21E-06 | 1.16E-05 | 0.2897 | 305.1752 | Fatty Acyls |
| Solacauline | 0.000119 | 0.000227 | -0.274 | 423.7319 | Steroids and steroid derivatives |
| Tezampanel | 2.93E-06 | 8.37E-06 | 0.2493 | 557.3289 | Carboxylic acids and derivatives |
| Oxdralazine | 3.85E-05 | 8.19E-05 | 0.3186 | 246.156 | Organonitrogen compounds |
| 5-Fluorouridine | 0.000421 | 0.000716 | 0.253 | 263.0696 | Pyrimidine nucleosides |
| Thr Val Thr | 2.20E-06 | 6.83E-06 | 0.2588 | 320.1814 | |
| 3-Hydroxytetradecanedioic acid | 3.03E-08 | 1.96E-07 | -0.2751 | 316.2121 | Fatty Acyls |
| Succinyl-trialanine-4-nitroanilide | 0.000375 | 0.000646 | 0.2827 | 432.1512 | Carboxylic acids and derivatives |
| Threonyltyrosine | 0.000225 | 0.000403 | 0.2925 | 281.1137 | Carboxylic acids and derivatives |
| Leucyl-Glutamate | 4.82E-08 | 2.86E-07 | 0.2525 | 243.1339 | Carboxylic acids and derivatives |
| Carbamoyl cholesterol | 1.20E-05 | 2.95E-05 | -0.2659 | 394.3425 | Steroids and steroid derivatives |
| Gamma-Glutamylglutamine | 1.03E-09 | 1.18E-08 | 0.2149 | 274.104 | Carboxylic acids and derivatives |
| Cholylglutamic acid | 6.75E-06 | 1.74E-05 | 0.2656 | 558.3085 | |
| Indicine | 1.05E-05 | 2.57E-05 | 0.2662 | 336.1194 | |
| Idazoxan | 1.03E-07 | 4.73E-07 | -0.2633 | 249.0875 | Benzodioxanes |
| PA(18:1(11Z)/PGD1) | 0.000676 | 0.001105 | 0.3912 | 409.2318 | |
| Esculentic acid (Phytolacca) | 4.31E-07 | 1.61E-06 | -0.2039 | 523.3048 | Prenol lipids |
| 5-Butyltetrahydro-2-oxo-3-furancarboxylic acid | 1.07E-05 | 2.60E-05 | 0.2645 | 417.177 | Lactones |
| 3,4-Dihydroxymandelic Acid | 0.000516 | 0.000868 | 0.3472 | 165.0183 | Phenols |
| Gly Leu Phe | 1.87E-05 | 4.31E-05 | 0.2811 | 336.1915 | |
| L-a-Lysophosphatidylserine | 9.46E-05 | 0.000184 | 0.2106 | 524.2975 | |
| Terbutaline | 3.09E-06 | 9.15E-06 | -0.2676 | 514.2913 | Phenols |
| 6-Hydroxynon-6-enoylcarnitine | 1.87E-06 | 5.69E-06 | 0.257 | 350.1714 | Fatty Acyls |
| Isoleucyl-Asparagine | 7.36E-06 | 1.88E-05 | 0.2575 | 244.1296 | Carboxylic acids and derivatives |
| Furanogermenone | 0.004038 | 0.005686 | 0.3198 | 487.2793 | Prenol lipids |
| Biotinyl-5'-AMP | 0.01941 | 0.02516 | -0.3784 | 610.0942 | Purine nucleotides |
| Leu Gly Leu | 7.64E-07 | 2.82E-06 | 0.2407 | 302.2072 | |
| Isomugineic acid | 1.93E-11 | 6.05E-10 | 0.26 | 362.1557 | Carboxylic acids and derivatives |
| 4-Methylbenzyl alcohol | 6.56E-10 | 9.04E-09 | 0.2152 | 267.1339 | Benzene and substituted derivatives |
| Gln Val Glu | 6.16E-08 | 3.48E-07 | 0.2351 | 375.1873 | |
| Aspartic Acid | 5.57E-09 | 4.48E-08 | 0.1784 | 132.029 | Carboxylic acids and derivatives |
| (2R)-2-Acetamido-3-[[(2R)-2-acetamido-2-carboxyethyl]disulfanyl]propanoic acid | 0.002654 | 0.003929 | -0.3302 | 323.037 | Carboxylic acids and derivatives |
| 1-Deoxynojirimycin | 2.68E-08 | 1.77E-07 | -0.2753 | 164.0918 | Piperidines |
| Gly Ile Lys | 2.38E-07 | 1.06E-06 | 0.2256 | 317.2182 | |
| Edotecarin | 0.00042 | 0.000717 | -0.2664 | 589.1608 | Phenol ethers |
| Decarbamoylsaxitoxin | 2.76E-06 | 8.27E-06 | 0.2733 | 320.145 | Saxitoxins, gonyautoxins, and derivatives |
| Acetylisoniazid | 2.22E-06 | 6.58E-06 | -0.2483 | 536.2058 | Pyridines and derivatives |
| 3-Ethyl-5-hydroxy-4,5-dimethyl-pyrrolin-2-one | 7.72E-07 | 2.85E-06 | 0.2813 | 173.1286 | Pyrrolines |

| | | | | | |
|---|---|---|---|---|---|
| Juzirine | 0.00445 | 0.006326 | 0.3689 | 326.1027 | Isoquinolines and derivatives |
| (6R,8Z)-6-Hydroxy-3-oxotetradecenoic acid | 0.000437 | 0.00074 | -0.3207 | 221.1536 | Fatty Acyls |
| Cortisone | 8.45E-05 | 0.000166 | 0.2942 | 424.2074 | Steroids and steroid derivatives |
| Queuine | 1.11E-10 | 2.32E-09 | -0.2189 | 319.1513 | Pyrrolopyrimidines |
| 3-Hydroxybenzoic Acid | 5.12E-09 | 4.22E-08 | 0.2043 | 137.0232 | Benzene and substituted derivatives |
| Alpha-Terpineol acetate | 5.62E-08 | 2.87E-07 | -0.2586 | 241.1439 | Prenol lipids |
| Virginiamycin m1 | 0.000223 | 0.0004 | 0.3038 | 508.2401 | Macrolide lactams |
| Ser Val Val | 1.79E-08 | 1.29E-07 | 0.2385 | 304.1865 | |
| Phenylglyoxylic acid | 4.14E-09 | 4.00E-08 | -0.2897 | 192.0657 | Benzene and substituted derivatives |
| Demethoxyrapamycin | 2.45E-07 | 1.09E-06 | 0.2357 | 453.768 | Macrolide lactams |
| Taurodeoxycholic acid | 5.52E-05 | 0.000113 | -0.1746 | 498.2881 | Steroids and steroid derivatives |
| P-Aminobenzoic acid | 1.38E-08 | 1.05E-07 | -0.2394 | 138.0551 | Benzene and substituted derivatives |
| (1R)-1-Amino-2-sulfanylethanesulfinic acid | 1.19E-08 | 8.14E-08 | -0.1996 | 139.9833 | Sulfinic acids and derivatives |
| Xi-8-Hydroxyhexadecanedioic acid | 3.51E-09 | 3.49E-08 | -0.2358 | 325.199 | Fatty Acyls |
| Asn Ala Ile | 5.10E-06 | 1.41E-05 | 0.2534 | 317.1817 | |
| (Z)-7-[(1R,2R,3R,5S)-2-(1,2-Dihydroxy-3-oxooctyl)-3,5-dihydroxycyclopentyl]hept-5-enoic acid | 2.24E-10 | 3.94E-09 | -0.2186 | 386.2283 | Fatty Acyls |
| Yucalexin P15 | 9.57E-05 | 0.000186 | 0.3032 | 394.1971 | Prenol lipids |
| 1,2-Disehydronorcoclaurine | 0.002528 | 0.003694 | 0.3666 | 602.2291 | Isoquinolines and derivatives |
| Glycyl-Methionine | 0.000387 | 0.000664 | 0.2508 | 205.0644 | Carboxylic acids and derivatives |
| 1,5-Naphthalenediamine | 3.99E-05 | 8.46E-05 | -0.3192 | 159.0918 | Naphthalenes |
| 7-[(1R,2R,3R,5S)-3,5-Dihydroxy-2-[(3S)-3-hydroxy-5-phenylpent-1-enyl]cyclopentyl]-N-ethylhept-5-enamide | 4.19E-05 | 8.84E-05 | 0.2735 | 460.2397 | Fatty Acyls |
| 9-Hydroxymegastigma-4,6,7-trien-3-one | 4.13E-07 | 1.69E-06 | 0.2984 | 207.138 | Prenol lipids |
| Glycyl-Threonine | 9.26E-07 | 3.10E-06 | 0.2502 | 175.0713 | Carboxylic acids and derivatives |
| N-Acetylasparagine | 6.75E-05 | 0.000135 | -0.2425 | 173.0557 | Carboxylic acids and derivatives |
| Epanolol | 8.68E-06 | 2.17E-05 | 0.2455 | 737.3339 | Phenol ethers |
| Fenhexamid | 0.0024 | 0.003578 | 0.3643 | 300.0539 | Benzene and substituted derivatives |
| Hydroxyprolyl-Valine | 3.02E-10 | 5.02E-09 | 0.2318 | 253.1181 | Carboxylic acids and derivatives |
| Gamma-Glutamyltryptophan | 1.03E-09 | 1.27E-08 | 0.2003 | 334.1396 | Carboxylic acids and derivatives |
| Moxaprindine | 3.17E-07 | 1.24E-06 | 0.2493 | 387.224 | Indanes |
| Pentaerythritol mononitrate | 6.03E-07 | 2.32E-06 | -0.2916 | 146.0437 | Organic oxoanionic compounds |
| Glutamylphenylalanine | 2.82E-09 | 2.60E-08 | 0.1992 | 293.1139 | Carboxylic acids and derivatives |
| Ile Ile Leu | 2.13E-06 | 6.65E-06 | 0.2541 | 358.2697 | |
| 3-hydroxyundecanoic acid | 5.54E-08 | 2.85E-07 | -0.2596 | 247.1545 | Hydroxy acids and derivatives |
| 4-(Methylnitrosamino)-1-(3-pyridyl)-1-butanol glucuronide | 4.74E-08 | 2.51E-07 | 0.2264 | 366.1301 | Fatty Acyls |
| L-alpha-Aminobutyric acid | 9.03E-10 | 1.15E-08 | -0.2056 | 104.071 | Carboxylic acids and derivatives |
| Lys Ile Glu | 2.80E-07 | 1.21E-06 | 0.2384 | 389.2392 | |
| Dihydrojasmonic acid | 1.41E-08 | 1.07E-07 | -0.2272 | 447.2717 | Fatty Acyls |
| Embutramide | 3.66E-08 | 2.04E-07 | 0.22 | 314.1716 | Benzene and substituted derivatives |
| Norhygrine | 5.78E-08 | 2.94E-07 | -0.2562 | 172.0969 | Organooxygen compounds |
| P-Phenylenediamine | 1.13E-05 | 2.75E-05 | -0.295 | 261.1338 | Benzene and substituted derivatives |
| Pantothenol | 1.45E-06 | 4.84E-06 | 0.2837 | 247.1652 | Organooxygen compounds |
| Glu-Thr | 9.79E-09 | 6.96E-08 | 0.2167 | 247.0929 | Carboxylic acids and derivatives |

| | | | | | |
|---|---|---|---|---|---|
| 5-Hydroxymethyluracil | 6.63E-08 | 3.71E-07 | -0.2874 | 184.0718 | Diazines |
| 5,9-Epidioxy-3-hydroxyergost-7-en-6-one | 7.17E-09 | 6.25E-08 | -0.2384 | 245.1496 | Steroids and steroid derivatives |
| 4-Methylbenzoic Acid | 0.004458 | 0.006336 | 0.3846 | 135.044 | Benzene and substituted derivatives |
| MeOSuc-Ala-Ala-Pro-Val-PNA | 2.25E-06 | 6.66E-06 | -0.2071 | 635.2707 | Carboxylic acids and derivatives |
| Metolachlor Morpholinone | 2.26E-08 | 1.56E-07 | -0.2607 | 251.1753 | |
| L-Leucyl-L-Alanine | 9.24E-07 | 3.09E-06 | 0.2425 | 201.1236 | Carboxylic acids and derivatives |
| Decanedioic acid | 3.01E-12 | 1.27E-10 | -0.1951 | 201.1124 | Fatty Acyls |
| Cyclotricuspidogenin C | 0.000351 | 0.000607 | -0.2394 | 581.3692 | Organooxygen compounds |
| Bicarbonate oxygen | 1.23E-09 | 1.34E-08 | 0.214 | 334.9764 | Organic carbonic acids and derivatives |
| Thr Val Phe | 1.45E-08 | 1.09E-07 | 0.225 | 366.2021 | |
| D-Digitoxose | 6.56E-09 | 5.80E-08 | -0.2471 | 297.1518 | Organooxygen compounds |
| Diprotin A | 1.59E-07 | 6.85E-07 | 0.228 | 340.2233 | Pyrroles |
| (S,E)-Zearalenone | 1.48E-06 | 4.63E-06 | -0.2161 | 363.1445 | Macrolides and analogues |
| 11,12,13-Trinor-1,3,5-bisabolatrien-10-oic acid | 3.24E-08 | 2.06E-07 | -0.2747 | 193.1224 | Prenol lipids |
| Lumichrome | 2.81E-09 | 2.92E-08 | -0.1966 | 243.0876 | Pteridines and derivatives |
| 5-Chloro-2'-deoxyuridine | 3.78E-09 | 3.29E-08 | -0.1971 | 243.0173 | Pyrimidine nucleosides |
| S-(PGA1)-glutathione | 0.001073 | 0.001699 | 0.31 | 642.3089 | Carboxylic acids and derivatives |
| Cephapirin | 4.34E-05 | 9.10E-05 | -0.2014 | 404.0401 | Carboxylic acids and derivatives |
| Cyasterone | 4.66E-08 | 2.49E-07 | -0.2099 | 519.2956 | Prenol lipids |
| Caprylic acid | 7.67E-06 | 1.94E-05 | -0.2772 | 189.1123 | Fatty Acyls |
| 2-Hydroxyquinoline-3-carboxylic acid | 1.33E-10 | 2.29E-09 | -0.1678 | 188.0344 | Quinolines and derivatives |
| Thr Phe Ile | 7.25E-08 | 3.98E-07 | 0.2365 | 380.2177 | |
| (3beta,5alpha,6alpha,7alpha,22E,24R)-5,6-Epoxyergosta-8,14,22-triene-3,7-diol | 2.73E-08 | 1.61E-07 | -0.1507 | 471.3102 | Steroids and steroid derivatives |
| Tranexamic Acid | 9.08E-11 | 1.96E-09 | -0.2509 | 190.1439 | Carboxylic acids and derivatives |
| PE(18:2/0:0) | 1.64E-06 | 5.06E-06 | 0.2008 | 476.2769 | |
| L-Tryptophan | 2.61E-08 | 1.56E-07 | 0.1622 | 203.0817 | Indoles and derivatives |
| Vomifoliol | 2.92E-08 | 1.91E-07 | -0.2622 | 189.1275 | Prenol lipids |
| Tryptamine | 1.92E-09 | 1.92E-08 | -0.2521 | 195.0689 | Indoles and derivatives |
| 5-O-[(3S)-1-Benzylpyrrolidin-3-yl] 3-O-methyl (4R)-2,6-dimethyl-4-(3-nitrophenyl)-3,4-dihydropyridine-3,5-dicarboxylate | 2.01E-06 | 6.03E-06 | -0.2319 | 536.2058 | Pyridines and derivatives |
| Epinepetalactone | 2.01E-10 | 3.65E-09 | -0.2421 | 167.1068 | Prenol lipids |
| Valylasparagine | 0.000369 | 0.000636 | 0.2606 | 230.1139 | Carboxylic acids and derivatives |
| N,N-Diallyl-tyrosyl-alpha-aminoisobutyric acid-phenylalanyl-leucine | 4.17E-10 | 6.33E-09 | 0.2547 | 304.176 | Carboxylic acids and derivatives |
| 5beta-Cholanic acid | 7.88E-07 | 2.69E-06 | -0.2204 | 405.2999 | Steroids and steroid derivatives |
| Ser Val Ile | 3.46E-08 | 2.17E-07 | 0.2071 | 318.2022 | |
| Ethinyl estradiol sulfate | 4.22E-05 | 8.89E-05 | -0.2695 | 394.1654 | Steroids and steroid derivatives |
| D-Pinitol | 1.61E-05 | 3.81E-05 | -0.2221 | 236.1128 | Organooxygen compounds |
| Clethodim | 0.000294 | 0.000517 | 0.3063 | 340.1142 | Organooxygen compounds |
| Pentadecanoic acid | 2.07E-07 | 9.48E-07 | -0.2522 | 306.2426 | Fatty Acyls |
| N-Palmitoyl Histidine | 3.04E-08 | 1.75E-07 | 0.2276 | 414.2713 | Carboxylic acids and derivatives |
| Sotalol | 1.45E-06 | 4.55E-06 | 0.2336 | 317.1167 | Benzene and substituted derivatives |
| Edetic Acid | 3.61E-06 | 1.04E-05 | 0.2592 | 334.1238 | Carboxylic acids and derivatives |
| Betamethasone 17-benzoate | 0.000106 | 0.000204 | -0.2226 | 529.2602 | Steroids and steroid derivatives |

| | | | | | |
|---|---|---|---|---|---|
| Trp Phe | 1.17E-07 | 5.93E-07 | 0.2615 | 352.1653 | |
| Hydroxyprolyl-Gamma-glutamate | 1.59E-09 | 1.65E-08 | 0.2228 | 304.1144 | Carboxylic acids and derivatives |
| Vomifoliol 9-[xylosyl-(1->6)-glucoside] | 1.14E-05 | 2.81E-05 | 0.2206 | 501.2289 | Fatty Acyls |
| 5'-Guanidinonaltrindole | 5.77E-06 | 1.52E-05 | 0.24 | 470.2244 | Morphinans |
| Artemisin | 9.98E-10 | 1.15E-08 | -0.2527 | 261.1127 | Prenol lipids |
| 10-Hydroxycarbazepine | 3.49E-05 | 7.51E-05 | 0.2298 | 531.2029 | Benzazepines |
| Betaine aldehyde | 6.58E-06 | 1.76E-05 | -0.2314 | 147.0634 | Organonitrogen compounds |
| Asperagenin | 0.000283 | 0.000498 | -0.2289 | 431.3115 | Prenol lipids |
| 3-Methyldioxyindole | 1.89E-10 | 3.04E-09 | -0.1759 | 144.0443 | Indoles and derivatives |
| 2,6-Dimethoxy-1,4-benzoquinone | 0.003391 | 0.004934 | 0.2381 | 149.0234 | Organooxygen compounds |
| 7-Methylhypoxanthine | 1.52E-06 | 5.02E-06 | -0.2426 | 151.0616 | Imidazopyrimidines |
| Rohitukine | 0.000419 | 0.000712 | 0.258 | 323.16 | Piperidines |
| Pro Trp Val | 8.38E-06 | 2.16E-05 | 0.2668 | 401.2181 | |
| Ala Ile Phe | 1.44E-06 | 4.82E-06 | 0.2484 | 350.2071 | |
| Artabsin | 1.13E-09 | 1.36E-08 | -0.2483 | 266.175 | Lactones |
| Val Ala Ser | 4.21E-08 | 2.56E-07 | 0.2214 | 276.1553 | |
| Skimmin | 0.02841 | 0.0351 | 0.4879 | 324.0865 | Coumarins and derivatives |
| Glycerophosphoinositol | 5.77E-05 | 0.000118 | -0.2147 | 333.0585 | Glycerophospholipids |
| ALA-ILE | 1.46E-06 | 4.58E-06 | 0.217 | 201.1236 | Carboxylic acids and derivatives |
| Glu Ser Leu | 1.68E-12 | 1.03E-10 | 0.2103 | 348.1764 | |
| 8beta-Angeloyloxy-15-hydroxy-1alpha,10R-dimethoxy-3-oxo-11(13)-germacren-12,6alpha-olide | 2.85E-07 | 1.23E-06 | -0.2228 | 463.1707 | Prenol lipids |
| Perindopril | 3.89E-06 | 1.11E-05 | -0.2589 | 351.2309 | Carboxylic acids and derivatives |
| LysoPE(18:2(9Z,12Z)/0:0) | 5.59E-06 | 1.53E-05 | 0.2079 | 478.2927 | Glycerophospholipids |
| L-Alanyl-l-Valine | 1.94E-06 | 5.86E-06 | 0.2456 | 187.1079 | Carboxylic acids and derivatives |
| 4-Hydroxyphenyl-2-propionic acid | 0.000714 | 0.001169 | -0.28 | 331.1182 | Phenylpropanoic acids |
| Alpha-Tocotrienoxyl radical | 2.74E-06 | 8.24E-06 | -0.2004 | 407.3266 | Prenol lipids |
| Ile Ile Arg | 3.57E-06 | 1.03E-05 | 0.2202 | 201.1474 | |
| (3R,4R)-4-Amino-1-[[4-[(3-methoxyphenyl)amino]pyrrolo[2,1-f][1,2,4]triazin-5-yl]methyl]piperidin-3-ol | 1.31E-07 | 6.48E-07 | 0.2268 | 432.2085 | Pyrrolotriazines |
| N-(2,3,4-Trihydroxybutyl)-L-valine | 1.83E-07 | 7.77E-07 | 0.255 | 202.1076 | Carboxylic acids and derivatives |
| 4',6-Dihydroxyflavone | 0.000165 | 0.000306 | 0.2546 | 255.0651 | |
| Leu-Gly-Gly | 2.63E-08 | 1.57E-07 | 0.1985 | 244.1297 | |
| Acetophenone | 0.009986 | 0.01321 | 0.2825 | 121.065 | Organooxygen compounds |
| Val His Phe | 6.63E-05 | 0.000134 | 0.2891 | 402.2134 | |
| Gly Ile Phe | 1.18E-05 | 2.91E-05 | 0.2167 | 336.1915 | |
| His Val Thr | 3.05E-05 | 6.65E-05 | 0.29 | 356.1925 | |
| 4-Acetylbutyrate | 1.88E-09 | 1.89E-08 | -0.2452 | 175.0602 | Fatty Acyls |
| Beta-Alanyl-L-arginine | 0.009336 | 0.01241 | 0.4288 | 529.2615 | Peptidomimetics |
| 3,4-Dimethoxy-N-(4-(3-nitrophenyl)thiazol-2-yl)benzenesulfonamide | 1.25E-05 | 3.00E-05 | -0.1841 | 466.0414 | Benzene and substituted derivatives |
| Glu Ile Leu | 2.01E-06 | 6.36E-06 | 0.2155 | 374.2284 | |
| Oxoglutaric acid | 1.72E-07 | 7.35E-07 | 0.1688 | 351.0563 | Keto acids and derivatives |
| 2-Hydroxycarbamazepine | 0.01103 | 0.01447 | 0.4209 | 275.0774 | Morphinans |
| PC(20:3/0:0) | 0.001532 | 0.002332 | 0.2275 | 546.3554 | |

| | | | | | |
|---|---|---|---|---|---|
| 3,5,6-Trihydroxy-5-(hydroxymethyl)-2-methoxy-2-cyclohexen-1-one | 1.14E-10 | 2.02E-09 | -0.2054 | 249.061 | Organooxygen compounds |
| Quisultazine | 1.89E-07 | 7.95E-07 | -0.2084 | 450.1102 | Benzothiazines |
| Cynaratriol | 1.25E-09 | 1.36E-08 | 0.2221 | 281.1389 | Prenol lipids |
| (1r,3r)-1-Aminocyclopentane-1,3-dicarboxylic acid | 1.87E-08 | 1.34E-07 | -0.2173 | 138.0551 | Carboxylic acids and derivatives |
| Lactarorufin B | 6.98E-11 | 1.37E-09 | -0.1978 | 281.1389 | |
| 3-Hydroxypropyl methacrylate | 3.17E-08 | 1.80E-07 | -0.2161 | 287.1496 | Carboxylic acids and derivatives |
| P-Anisic Acid | 0.000268 | 0.000473 | 0.2058 | 151.0389 | Benzene and substituted derivatives |
| N-(2,4-difluorophenyl)-4-(2-oxo-2-piperidin-1-ylethyl)piperidine-1-carboxamide | 4.24E-06 | 1.20E-05 | 0.2379 | 366.2021 | |
| 13-Hydroxyabscisic acid | 4.68E-09 | 3.91E-08 | -0.2301 | 279.1233 | Prenol lipids |
| N-Stearoyl Glutamine | 1.14E-06 | 3.95E-06 | -0.2495 | 395.3265 | Carboxylic acids and derivatives |
| L-Glutamic Acid | 1.35E-08 | 1.03E-07 | -0.2445 | 130.0501 | Carboxylic acids and derivatives |
| (8R,9S,10S,13S,14S,17R)-17-Ethyl-3,17-dihydroxy-10,13-dimethyl-3,4,5,6,7,8,9,11,12,14,15,16-dodecahydro-1H-cyclopenta[a]phenanthren-2-one | 3.40E-05 | 7.33E-05 | -0.2558 | 352.2843 | Steroids and steroid derivatives |
| (S)-Bitalin A 12-glucoside | 6.69E-07 | 2.34E-06 | -0.2199 | 417.0961 | Organooxygen compounds |
| Ile Val Asn | 0.001213 | 0.001878 | 0.2824 | 345.2131 | |
| Gemeprost | 5.22E-07 | 2.05E-06 | -0.2263 | 359.2579 | Fatty Acyls |
| 5-(Galactosylhydroxy)-L-Lysine | 1.44E-07 | 7.02E-07 | -0.2147 | 325.1603 | Fatty Acyls |
| Asn Gly Leu | 2.70E-07 | 1.17E-06 | 0.2291 | 303.1663 | |
| Pirarubicinol | 3.22E-05 | 6.95E-05 | -0.2006 | 650.2271 | Anthracyclines |
| Androsterone glucuronide | 1.35E-05 | 3.28E-05 | 0.2349 | 499.2872 | Steroids and steroid derivatives |
| 8-Aminooctanoic acid | 3.52E-07 | 1.47E-06 | 0.245 | 357.2129 | Fatty Acyls |
| 4-Hydroxycinnamic acid | 9.92E-05 | 0.000192 | 0.2465 | 387.1075 | Cinnamic acids and derivatives |
| 2,3-dihydroxy-3-methylvalerate | 1.90E-05 | 4.34E-05 | -0.2634 | 147.0652 | Fatty Acyls |
| N-Allylglycine | 2.31E-06 | 6.82E-06 | 0.2332 | 344.182 | Carboxylic acids and derivatives |
| Dihydromelilotoside | 0.000101 | 0.000194 | 0.2925 | 327.1081 | Organooxygen compounds |
| Hovenine A | 6.65E-06 | 1.77E-05 | 0.2406 | 244.1656 | Carboxylic acids and derivatives |
| Neogitogenin | 6.93E-09 | 6.08E-08 | -0.2052 | 397.3059 | Prenol lipids |
| V-Pyrro/NO | 4.76E-09 | 3.97E-08 | 0.2172 | 373.1872 | Pyrrolidines |
| Laninamivir | 2.31E-06 | 7.11E-06 | 0.2536 | 347.1557 | Carboxylic acids and derivatives |
| Calcifediol lactone | 3.89E-08 | 2.14E-07 | -0.1843 | 473.2898 | Steroids and steroid derivatives |
| Macrolactin-A | 5.56E-05 | 0.000114 | 0.247 | 447.2085 | Macrolides and analogues |
| Stachyose | 6.11E-06 | 1.59E-05 | 0.2116 | 665.2129 | Organooxygen compounds |
| (2S,3'S)-alpha-Amino-2-carboxy-5-oxo-1-pyrrolidinebutanoic acid | 2.50E-06 | 7.29E-06 | -0.1782 | 211.0716 | Carboxylic acids and derivatives |
| Neobyakangelicol | 2.71E-05 | 5.95E-05 | -0.2341 | 297.0763 | Coumarins and derivatives |
| Valylleucine | 0.00099 | 0.001565 | 0.3223 | 231.1704 | Carboxylic acids and derivatives |
| Dehydrozingerone | 3.88E-05 | 8.21E-05 | -0.2524 | 237.0762 | Cinnamic acids and derivatives |
| 3-Hydroxyanthranilic Acid | 1.78E-10 | 2.90E-09 | -0.2136 | 152.0342 | Benzene and substituted derivatives |
| Caffeic Acid | 8.20E-08 | 3.89E-07 | 0.2462 | 179.034 | Cinnamic acids and derivatives |
| 5,6-DHET | 2.25E-09 | 2.41E-08 | -0.2362 | 361.2331 | Fatty Acyls |
| Asp Ile Ala | 3.91E-08 | 2.40E-07 | 0.2008 | 318.1659 | |
| 1-Piperideine-2-carboxylic acid | 1.14E-06 | 3.95E-06 | 0.2422 | 277.1182 | Pyridines and derivatives |
| Methionine sulfoxide | 0.002201 | 0.003247 | 0.2953 | 207.0798 | Carboxylic acids and derivatives |
| Citrazinic Acid | 1.49E-09 | 1.57E-08 | -0.2473 | 154.0135 | |
| Val Gln Leu | 2.68E-05 | 5.94E-05 | 0.2417 | 359.2287 | |

| | | | | | |
|---|---|---|---|---|---|
| N-(1-Carboxy-3-carboxanilidopropyl)alanylproline | 3.38E-07 | 1.31E-06 | 0.2182 | 390.1671 | Carboxylic acids and derivatives |
| Imazapyr | 3.87E-09 | 3.79E-08 | -0.2425 | 262.1185 | Carboxylic acids and derivatives |
| 3-Methoxyanthranilate | 6.00E-06 | 1.63E-05 | 0.2502 | 352.15 | Benzene and substituted derivatives |
| Vanilloyl glucose | 2.92E-07 | 1.15E-06 | -0.2141 | 311.0767 | Tannins |
| Isoleucyl-Aspartate | 1.12E-06 | 3.64E-06 | 0.1829 | 245.1137 | Carboxylic acids and derivatives |
| 4-Methyl-1,4-dihydropyridine-3,5-dicarbaldehyde | 5.11E-07 | 1.85E-06 | 0.2573 | 150.0549 | Pyridines and derivatives |
| Glycyl-Glutamine | 9.55E-12 | 3.48E-10 | -0.2285 | 186.0874 | Carboxylic acids and derivatives |
| PS(20:1(11Z)/18:3(9,11,15)-OH(13)) | 0.01079 | 0.01418 | 0.3952 | 436.758 | |
| Phloretin | 0.000104 | 0.000199 | 0.2505 | 273.0766 | Organooxygen compounds |
| 1-Methyladenosine | 1.47E-07 | 7.15E-07 | -0.2362 | 282.1197 | Purine nucleosides |
| 5-Hydroxyindoleacetate | 1.40E-07 | 6.88E-07 | 0.2306 | 192.0656 | Indoles and derivatives |
| D-Cathine | 9.44E-07 | 3.38E-06 | -0.2515 | 134.0966 | |
| PI(22:2(13Z,16Z)/20:5(6E,8Z,11Z,14Z,17Z)-OH(5)) | 1.31E-10 | 2.61E-09 | 0.2115 | 488.285 | |
| ALANYL-dl-LEUCINE | 1.13E-06 | 3.93E-06 | -0.2382 | 203.1391 | |
| Methylphenobarbital | 1.55E-12 | 9.92E-11 | -0.2091 | 247.1076 | Diazines |
| L-Agaridoxin | 0.01735 | 0.02211 | -0.3085 | 255.0975 | Carboxylic acids and derivatives |
| Leu Val | 0.000169 | 0.000311 | -0.2617 | 231.1703 | |
| Serylthreonine | 6.78E-05 | 0.000136 | 0.2421 | 205.082 | Carboxylic acids and derivatives |
| 1,2-Cyclohexanediol | 2.98E-10 | 4.98E-09 | -0.2336 | 274.2011 | Organooxygen compounds |
| Leukotriene B4 ethanolamide | 4.10E-05 | 8.67E-05 | -0.1885 | 421.306 | Organonitrogen compounds |
| Dioscoretine | 1.32E-10 | 2.61E-09 | -0.2201 | 283.2015 | Fatty Acyls |
| HBOA trihexose | 1.33E-07 | 5.91E-07 | 0.1759 | 202.1076 | Carboxylic acids and derivatives |
| Cerasinone | 5.03E-07 | 1.83E-06 | -0.1644 | 329.1026 | Flavonoids |
| Gitonin | 6.60E-06 | 1.76E-05 | 0.2091 | 526.2742 | Steroids and steroid derivatives |
| Threonyltryptophan | 1.86E-08 | 1.17E-07 | 0.19 | 350.1351 | Carboxylic acids and derivatives |
| Sirolimus | 0.003057 | 0.004403 | 0.3129 | 479.771 | Benzene and substituted derivatives |
| 1-Acetamidocyclopentanecarboxylic acid | 2.61E-07 | 1.14E-06 | -0.2612 | 172.0969 | |
| Abiraterone | 4.20E-06 | 1.15E-05 | -0.221 | 384.2132 | Steroids and steroid derivatives |
| Ginkgolide A | 0.00127 | 0.001985 | -0.2514 | 389.1234 | Prenol lipids |
| Gln Ser Val | 1.29E-07 | 6.42E-07 | 0.2356 | 333.1766 | |
| Austdiol | 3.68E-05 | 7.87E-05 | -0.2502 | 278.1023 | Azaphilones |
| Methionine sulfone | 5.22E-08 | 2.71E-07 | -0.1761 | 180.0326 | Carboxylic acids and derivatives |
| Glu Leu Lys | 1.68E-05 | 3.94E-05 | 0.2249 | 389.2391 | |
| 8-Hydroxy-4(6)-lactarene-5,14-diol | 2.99E-07 | 1.18E-06 | -0.2081 | 299.1861 | Organooxygen compounds |
| Oxindole | 8.19E-08 | 4.39E-07 | -0.2497 | 134.0602 | Indoles and derivatives |
| 2'-hydroxypseudobaptigenin | 0.000785 | 0.001271 | -0.2431 | 359.0427 | Isoflavonoids |
| L-Xylonic acid | 7.93E-12 | 2.57E-10 | 0.1745 | 165.0394 | Organooxygen compounds |
| Gly Val Leu | 9.46E-06 | 2.40E-05 | 0.1871 | 288.1917 | |
| (4S,6S)-3,4,5,6-Tetrahydro-4-hydroxy-6-methyl-2H-pyran-2-one | 3.56E-05 | 7.62E-05 | -0.2564 | 175.0602 | Lactones |
| Lys Ile | 2.06E-05 | 4.72E-05 | 0.1966 | 260.1968 | |
| 4-Hydroxyquinoline | 5.65E-06 | 1.54E-05 | -0.1772 | 146.0601 | Quinolines and derivatives |
| Ala Met Leu | 6.08E-06 | 1.64E-05 | 0.264 | 334.1789 | |
| Rolitetracycline | 0.007291 | 0.01003 | 0.3326 | 508.2038 | Tetracyclines |
| Isoleucyl-Lysine | 4.72E-10 | 6.92E-09 | 0.215 | 260.1969 | Carboxylic acids and derivatives |

| | | | | | |
|---|---|---|---|---|---|
| Lys Val | 6.03E-05 | 0.000122 | 0.227 | 246.1812 | |
| (+/-)-Citronellyl acetate | 0.000173 | 0.000318 | -0.2049 | 593.4769 | Fatty Acyls |
| Lanthionine ketimine | 9.08E-08 | 4.26E-07 | 0.2309 | 169.9907 | Carboxylic acids and derivatives |
| 2-(Difluoromethyl)arginine | 0.000131 | 0.000248 | 0.2462 | 490.2507 | Carboxylic acids and derivatives |
| 3,4-Dimethyl-5-pentyl-2-furanpropanoic acid | 8.06E-05 | 0.000159 | -0.2408 | 203.1431 | Fatty Acyls |
| L-Dopa | 1.79E-06 | 5.77E-06 | -0.1951 | 162.055 | Carboxylic acids and derivatives |
| Dihydroorotic Acid | 0.001518 | 0.002336 | -0.3038 | 157.0244 | Carboxylic acids and derivatives |
| 9Z-Heptadecenoic acid | 4.37E-07 | 1.77E-06 | -0.238 | 310.2739 | Fatty Acyls |
| Ser Ile Tyr | 7.78E-08 | 4.21E-07 | 0.217 | 382.197 | |
| Diphenoxylate | 3.37E-06 | 9.83E-06 | 0.2211 | 417.2344 | Benzene and substituted derivatives |
| Isoleucyl-Methionine | 0.001541 | 0.002344 | -0.2971 | 263.1425 | Carboxylic acids and derivatives |
| Cotinine glucuronide | 2.46E-08 | 1.67E-07 | 0.2124 | 394.1607 | Pyrimidine nucleotides |
| Difructose anhydride III | 1.02E-07 | 4.69E-07 | 0.1935 | 323.0979 | Organooxygen compounds |
| Tetrahydrocortisone | 6.26E-06 | 1.68E-05 | -0.2226 | 365.23 | Steroids and steroid derivatives |
| Salicin | 5.35E-12 | 1.92E-10 | 0.1955 | 321.0723 | Organooxygen compounds |
| PC(5-iso PGF2VI/DiMe(11,3)) | 0.01636 | 0.02093 | 0.3926 | 457.7635 | |
| L-Kynurenine | 7.22E-07 | 2.70E-06 | -0.2086 | 241.1183 | Organooxygen compounds |
| 16-oxo-palmitate | 3.03E-05 | 6.58E-05 | 0.2089 | 314.208 | Fatty Acyls |
| Isovalerylglutamic acid | 1.40E-05 | 3.32E-05 | -0.2454 | 230.1027 | Carboxylic acids and derivatives |
| [1S-[1alpha,2alpha(Z),3alpha,4alpha]]-7-[3-[[2-[(Phenylamino)carbonyl]hydrazino]methyl]-7-oxabicyclo[2.2.1]hept-2-yl]-5-heptenoicacid | 0.000253 | 0.000449 | -0.2455 | 408.1874 | Benzene and substituted derivatives |
| 1-a,24R,25-Trihydroxyvitamin D2 | 1.53E-08 | 1.14E-07 | -0.2056 | 427.3213 | Steroids and steroid derivatives |
| Pro Phe Val | 0.000461 | 0.000777 | 0.2638 | 362.2072 | |
| Pro Ala | 7.18E-08 | 3.94E-07 | 0.1924 | 187.1078 | |
| Sericoside | 3.30E-05 | 7.14E-05 | 0.2615 | 345.1951 | Prenol lipids |
| Ureidosuccinic Acid | 0.000289 | 0.000508 | -0.2512 | 175.035 | Carboxylic acids and derivatives |
| DG(i-16:0/0:0/18:1(12Z)-O(9S,10R)) | 6.26E-05 | 0.000127 | -0.2024 | 631.4905 | |
| 7Z,10Z-Hexadecadienoic acid | 2.77E-06 | 8.30E-06 | -0.2238 | 275.2005 | Fatty Acyls |
| Neolinustatin | 1.06E-10 | 2.23E-09 | -0.2086 | 388.1599 | Organooxygen compounds |
| Threonylphenylalanine | 8.46E-07 | 2.86E-06 | 0.1938 | 265.1189 | Carboxylic acids and derivatives |
| Ala Val Tyr | 0.000132 | 0.000249 | 0.262 | 352.1865 | |
| N-Acetylsphinganine | 6.83E-05 | 0.000137 | 0.2505 | 344.3158 | Sphingolipids |
| 5-Fluoro-8-hydroxy-2-(dipropylamino)tetralin | 2.90E-06 | 8.29E-06 | 0.208 | 302.1351 | Tetralins |
| Heliotridine | 2.55E-07 | 1.12E-06 | 0.1807 | 328.2229 | |
| 4-(1,1,3,3-Tetramethylbutyl)-phenol | 8.52E-09 | 7.14E-08 | -0.2368 | 270.1811 | Benzene and substituted derivatives |
| Medroxyprogesterone | 2.42E-06 | 7.40E-06 | 0.2049 | 389.2029 | Steroids and steroid derivatives |
| Val Glu Ser | 2.13E-06 | 6.64E-06 | 0.2016 | 334.1607 | |
| Hexadeca-7,10,13-trienoic acid | 0.000295 | 0.000516 | -0.2522 | 215.1795 | Fatty Acyls |
| Dazmegrel | 4.76E-11 | 1.02E-09 | -0.1994 | 320.0803 | Indoles and derivatives |
| Coniferyl alcohol | 5.96E-10 | 7.61E-09 | -0.2002 | 225.0761 | Phenols |
| (2E)-Hept-2-enedioylcarnitine | 0.000269 | 0.000476 | -0.2683 | 301.1545 | Fatty Acyls |
| Asp Phe | 3.26E-06 | 9.58E-06 | 0.232 | 281.1131 | |
| Gly Leu Arg | 0.000134 | 0.000252 | 0.2327 | 345.2243 | |

| | | | | | |
|---|---|---|---|---|---|
| Butyl 3-hydroxy-2-methylidenebutanoate | 1.05E-08 | 7.37E-08 | -0.2176 | 217.1074 | Hydroxy acids and derivatives |
| N-lactoyl-Tyrosine | 2.73E-05 | 5.99E-05 | -0.2333 | 252.0872 | Carboxylic acids and derivatives |
| N-(N-(3-Amino-3-carboxypropyl)-3-amino-3-carboxypropyl)azetidine-2-carboxylic acid | 1.46E-07 | 6.39E-07 | 0.2061 | 302.1351 | Carboxylic acids and derivatives |
| Hydroxybutyl | 7.33E-10 | 8.99E-09 | -0.2263 | 203.1281 | Organooxygen compounds |
| Asp-Phe | 3.59E-06 | 1.00E-05 | 0.1979 | 279.0981 | Carboxylic acids and derivatives |
| Glycyl-Tryptophan | 4.26E-05 | 8.94E-05 | 0.203 | 260.1036 | Carboxylic acids and derivatives |
| 11-Meo-fes | 0.000806 | 0.001298 | 0.2901 | 334.1916 | Steroids and steroid derivatives |
| Phe Val Ile | 2.33E-07 | 1.05E-06 | 0.2154 | 378.2384 | |

Table S2 Summary of the up-regulated and down-regulated metabolites in BS group vs MD group

| Metabolite | Adjusted P value | FDR | $Log_2FC(BS/MD)$ | m/z | Class |
|---|---|---|---|---|---|
| Coumaryl acetate | 1.03E-05 | 0.000297 | -3.3234 | 237.0761 | Benzene and substituted derivatives |
| Deferitrin | 8.89E-08 | 3.21E-05 | -1.9062 | 254.0481 | Carboxylic acids and derivatives |
| Epiandrosterone | 4.33E-06 | 0.000263 | -1.1588 | 329.1858 | Steroids and steroid derivatives |
| N,n-Diethyl-2,4-Dimethoxybenzamide | 0.00102 | 0.008094 | -2.532 | 270.17 | |
| Cassythicine | 0.001241 | 0.009258 | -2.8858 | 348.1228 | Aporphines |
| Simulansine | 2.68E-06 | 0.000124 | -1.2633 | 699.3585 | Quinolines and derivatives |
| PA(14:1(9Z)/20:5(5Z,8Z,11Z,14Z,16E)-OH(18R)) | 1.87E-08 | 6.09E-06 | -1.0359 | 701.3736 | |
| Tanakine | 0.000266 | 0.002388 | -1.5183 | 264.1236 | Indoles and derivatives |
| Camphorsulfonic acid | 9.15E-05 | 0.001717 | -0.8607 | 232.0791 | Prenol lipids |
| (S)-N-Methylcoclaurine | 6.17E-05 | 0.001322 | -1.0093 | 363.17 | Isoquinolines and derivatives |
| 5-Hydroxyindoleacetaldehyde | 5.08E-06 | 0.000184 | -1.0535 | 174.055 | Indoles and derivatives |
| 2-Methoxyestrone | 0.000425 | 0.004672 | -1.2828 | 345.1443 | Steroids and steroid derivatives |
| Lacto-N-tetraose | 0.000236 | 0.002198 | -1.9828 | 688.2295 | Organooxygen compounds |
| Linolenelaidic acid | 5.39E-07 | 7.40E-05 | 0.8755 | 323.1964 | Fatty Acyls |
| Monocrotaline | 2.61E-06 | 0.00019 | 1.0728 | 325.1545 | Pyrrolizines |
| Tremetone | 0.00014 | 0.002237 | -0.9751 | 220.1331 | Benzene and substituted derivatives |
| 4-Hydroxyphenylacetic acid sulfate | 6.73E-07 | 5.90E-05 | -0.9054 | 230.9962 | Phenols |
| Aesculetin | 7.28E-05 | 0.001484 | -0.6588 | 357.0571 | Coumarins and derivatives |
| Pyriproxyfen | 0.01278 | 0.04626 | -1.5332 | 339.1703 | Benzene and substituted derivatives |
| Coumarin | 4.81E-05 | 0.000767 | -0.8316 | 191.0341 | Coumarins and derivatives |
| Equol | 1.31E-08 | 4.97E-06 | 0.8171 | 287.0921 | Isoflavonoids |
| Prunetin | 2.61E-06 | 0.000123 | -0.7859 | 283.0607 | Isoflavonoids |
| 5-(4'-Hydroxyphenyl)-gamma-valerolactone 4'-sulfate | 0.000122 | 0.001412 | -0.9683 | 317.033 | Organic sulfuric acids and derivatives |
| Gentianine | 4.15E-05 | 0.000696 | -0.7575 | 174.055 | Pyranopyridines |
| Mpomeovt | 0.000188 | 0.002682 | -0.5979 | 538.2527 | Carboxylic acids and derivatives |
| Remikiren | 9.70E-08 | 3.26E-05 | 0.5518 | 648.38 | Carboxylic acids and derivatives |
| Dihydrozeatin-7-N-glucoside | 3.18E-07 | 5.97E-05 | 0.5706 | 425.2126 | Organooxygen compounds |
| Glucosyl passiflorate | 0.000177 | 0.001799 | -0.6085 | 731.3844 | Prenol lipids |
| Soyasapogenol B 3-O-b-D-glucuronide | 0.007838 | 0.0329 | -0.6808 | 657.3968 | Prenol lipids |
| (+/-)-Tryptophan | 2.28E-05 | 0.000695 | -0.6574 | 409.1866 | Indoles and derivatives |
| Trichlormethine | 2.38E-05 | 0.000483 | -0.6834 | 248.0017 | Organonitrogen compounds |
| 6S,9R-Dihydroxy-4,7E-megastigmadien-3-one 9-[apiosyl-(1->6)-glucoside] | 3.35E-05 | 0.0006 | -0.5999 | 517.2277 | Fatty Acyls |
| Notoginsenoside R10 | 1.15E-05 | 0.000454 | -0.4667 | 577.3343 | Steroids and steroid derivatives |
| Momordicoside E | 3.51E-07 | 3.97E-05 | -0.5226 | 731.3845 | Steroids and steroid derivatives |
| Descarbonyl-lacosamide | 2.95E-10 | 1.04E-06 | 0.5522 | 250.1549 | Carboxylic acids and derivatives |
| (6R,7R)-6-Methoxy-3,7-dimethyl-8-oxo-5-thia-1-azabicyclo[4.2.0]oct-2-ene-2-carboxylic acid | 0.002805 | 0.01614 | -0.9059 | 208.0428 | Carboxylic acids and derivatives |
| DG(10:0/0:0/20:4(8Z,11Z,14Z,17Z)-2OH(5S,6R)) | 0.006646 | 0.02929 | -0.7563 | 294.1977 | |
| (R)-Canadine | 0.01172 | 0.04343 | -0.7889 | 304.1331 | Protoberberine alkaloids and derivatives |
| 4-Deacetylneosolaniol | 7.59E-07 | 8.82E-05 | 0.5103 | 363.1395 | Prenol lipids |
| 4a-Carbinolamine tetrahydrobiopterin | 3.13E-06 | 0.000214 | -0.5968 | 222.0986 | Pteridines and derivatives |
| 1-Benzylpiperidine-4-carboxylic acid | 0.00255 | 0.01179 | -0.8404 | 264.1236 | Piperidines |
| TUBAIC ACID | 0.002134 | 0.01042 | -0.8201 | 219.0655 | |

| | | | | | |
|---|---|---|---|---|---|
| Butyl (S)-3-hydroxybutyrate glucoside | 1.45E-05 | 0.000519 | 0.5563 | 355.197 | Fatty Acyls |
| 3,5,7-Trimethylepicatechin | 4.92E-06 | 0.000281 | 0.5043 | 333.1331 | Flavonoids |
| 8-Deoxylactucin | 1.47E-06 | 8.99E-05 | 0.6181 | 281.0812 | Lactones |
| Zingerone | 0.003808 | 0.01528 | -0.8562 | 175.0754 | Phenols |
| Histamine | 6.70E-06 | 0.00034 | -0.638 | 112.0873 | Organonitrogen compounds |
| N-Jasmonoylisoleucine | 4.18E-08 | 2.27E-05 | 0.5008 | 324.2168 | Carboxylic acids and derivatives |
| 2-Hydroxy-p-mentha-1,8-dien-6-one | 2.70E-07 | 5.65E-05 | 0.579 | 208.1333 | Prenol lipids |
| Graveoline | 0.0046 | 0.01732 | -0.7423 | 324.087 | Quinolines and derivatives |
| PIP(PGF1alpha/16:0) | 0.003507 | 0.01436 | -0.6208 | 494.2432 | |
| Fuegin | 1.45E-06 | 0.000133 | 0.4588 | 267.159 | |
| Benzyl gentiobioside | 0.001076 | 0.006384 | 0.8042 | 477.1601 | Organooxygen compounds |
| Dyspropterin | 5.02E-07 | 4.99E-05 | -0.6226 | 218.0675 | Pteridines and derivatives |
| 3-(2-Hydroxy-4-methylphenyl)-2-butanone | 0.003125 | 0.01737 | -0.685 | 220.1333 | Prenol lipids |
| Oxymorphone | 1.75E-07 | 4.62E-05 | 0.5178 | 319.1651 | Phenanthrenes and derivatives |
| Atractylenolide III | 2.73E-07 | 3.56E-05 | 0.4488 | 541.2824 | Prenol lipids |
| Adelmidrol | 7.30E-07 | 6.27E-05 | 0.5516 | 295.1657 | Organonitrogen compounds |
| Pyrazofurin | 0.00681 | 0.0298 | -0.7202 | 277.1117 | Nucleoside and nucleotide analogues |
| Estriol 3-sulfate | 1.81E-05 | 0.000416 | 0.4807 | 413.129 | Steroids and steroid derivatives |
| Niazirinin | 2.63E-08 | 7.71E-06 | -0.4116 | 366.1186 | Organooxygen compounds |
| 7,4'-Dihydroxy-8-methylflavan | 4.27E-06 | 0.000166 | 0.512 | 301.1076 | Flavonoids |
| Hydroxy Tyrosol -Acetate | 1.03E-05 | 0.000437 | 0.4712 | 226.1074 | Benzene and substituted derivatives |
| 7-O-Succinyl macrolactin A | 0.005085 | 0.01856 | 0.5271 | 537.226 | Macrolides and analogues |
| Saxitoxin | 6.72E-06 | 0.00034 | 0.4332 | 341.1707 | Saxitoxins, gonyautoxins, and derivatives |
| Bursin | 1.73E-06 | 0.000147 | 0.4414 | 322.2011 | Carboxylic acids and derivatives |
| PE(LTE4/20:2(11Z,14Z)) | 0.002504 | 0.01484 | -0.442 | 464.2846 | |
| PG(a-13:0/i-18:0) | 3.56E-07 | 6.23E-05 | 0.3914 | 377.2328 | Glycerophospholipids |
| Tyramine-O-sulfate | 0.000439 | 0.003389 | -0.5425 | 216.0328 | Organic sulfuric acids and derivatives |
| Cyclosquamosin E | 1.49E-05 | 0.000527 | -0.3935 | 334.1607 | Carboxylic acids and derivatives |
| Scopoletin | 0.000825 | 0.005261 | -0.5475 | 237.0396 | Coumarins and derivatives |
| (3b,20R,22R)-3,20,27-Trihydroxy-1-oxowitha-5,24-dienolide 3-glucoside | 0.002755 | 0.0159 | -0.4264 | 657.3237 | Prenol lipids |
| 2-(2-Hydroxyethyl)phenyl hydrogen sulfate | 1.99E-06 | 0.000104 | -0.3448 | 217.0169 | Organic sulfuric acids and derivatives |
| Streptolydigin | 0.00166 | 0.00866 | -0.4872 | 645.3087 | Carboxylic acids and derivatives |
| Chenodeoxycholyltaurine | 0.000288 | 0.003575 | 0.3985 | 522.2861 | Steroids and steroid derivatives |
| 3-(4-Methylphenyl)oxiranecarboxylic acid | 0.005418 | 0.0194 | -0.6758 | 223.0604 | Benzene and substituted derivatives |
| Homovanillic acid | 0.000314 | 0.002665 | -0.373 | 181.0496 | Phenols |
| Eremopetasinorol | 4.53E-07 | 7.17E-05 | 0.4363 | 250.1802 | Organooxygen compounds |
| 8-Ocimenyl acetate | 9.97E-07 | 0.000102 | 0.4735 | 236.1645 | Fatty Acyls |
| Glycerol tributanoate | 0.007181 | 0.03081 | -0.5162 | 347.1423 | Glycerolipids |
| Bursopoietin | 1.25E-07 | 3.85E-05 | 0.3529 | 322.2011 | Carboxylic acids and derivatives |
| 2-Benzyl-aminoethanol | 1.28E-07 | 3.85E-05 | 0.3928 | 325.1864 | Benzene and substituted derivatives |
| Propranolol | 0.0051 | 0.02442 | -0.5218 | 323.1751 | Naphthalenes |
| 1-O-Caffeoyl-beta-D-glucose | 0.000122 | 0.002022 | -0.442 | 384.1288 | Steroids and steroid derivatives |
| Soyasaponin I | 0.001017 | 0.008093 | -0.3135 | 943.526 | Prenol lipids |
| 16-Hydroxy-10-oxohexadecanoic acid | 5.30E-09 | 3.11E-06 | 0.3939 | 323.1605 | Fatty Acyls |

| | | | | | |
|---|---|---|---|---|---|
| [(2R,5R)-5-(2-Amino-6-oxo-1H-purin-9-yl)-3,4-dihydroxyoxolan-2-yl]methyl [(2R,5R)-5-(4-amino-2-oxopyrimidin-1-yl)-3,4-dihydroxyoxolan-2-yl]methyl hydrogen phosphate | 0.001354 | 0.007469 | -0.4348 | 569.1135 | (5'->5')-dinucleotides |
| Docebenone | 3.77E-06 | 0.00024 | 0.4006 | 349.1756 | Fatty Acyls |
| N-Palmitoyl Asparagine | 0.009515 | 0.02935 | -0.6689 | 241.017 | Organic sulfuric acids and derivatives |
| Genkwanin | 0.004206 | 0.02133 | -0.5492 | 285.0757 | Flavonoids |
| 5',8-Dihydroxy-3',4',7-trimethoxyflavan | 1.42E-05 | 0.000514 | 0.3773 | 333.1331 | Flavonoids |
| Myxin | 0.007881 | 0.0257 | -0.6427 | 303.0617 | Diazanaphthalenes |
| Phenylalanylvaline | 5.18E-07 | 7.40E-05 | 0.3534 | 297.1807 | Carboxylic acids and derivatives |
| Arvensoside D | 0.001192 | 0.006854 | -0.3216 | 941.5095 | Prenol lipids |
| Skimmin | 0.01142 | 0.04265 | -0.5979 | 324.0865 | Coumarins and derivatives |
| Cilengitide | 0.000481 | 0.005048 | -0.3573 | 553.2939 | Carboxylic acids and derivatives |
| Acetophenone | 0.003478 | 0.0187 | -0.3461 | 121.065 | Organooxygen compounds |
| Sulfamethoxazole | 0.000779 | 0.005044 | -0.4286 | 565.1184 | Benzene and substituted derivatives |
| 3'-DEOXY-3'-FLUOROTHYMIDINE | 0.001015 | 0.006132 | -0.4542 | 243.0769 | Pyrimidine nucleosides |
| 5-Hydroxy-2,4-dimethylthiophen-3-one | 0.009499 | 0.02931 | -0.5724 | 165.0004 | Dihydrothiophenes |
| PG(20:4(6E,8Z,11Z,13E)-2OH(5S,15S)/16:1(9Z)) | 0.005263 | 0.02496 | 0.4081 | 412.2371 | |
| Yangonin | 2.16E-05 | 0.000663 | 0.3924 | 259.0965 | Kavalactones |
| (+/-)-Dulciol E | 2.47E-05 | 0.000727 | 0.3735 | 313.1069 | Benzopyrans |
| L-L-Homoglutathione | 0.0118 | 0.04357 | -0.5374 | 339.1337 | Peptidomimetics |
| 5'-Methylthioadenosine | 4.41E-05 | 0.001052 | 0.396 | 315.1225 | 5'-deoxyribonucleosides |
| Eugenol | 0.000379 | 0.003032 | -0.3885 | 209.0811 | Phenols |
| 3alpha-Hydroxyoreadone | 0.01394 | 0.04895 | -0.5103 | 270.1698 | Naphthofurans |
| Fluocortin butyl | 0.009627 | 0.02965 | -0.4878 | 445.2406 | Steroids and steroid derivatives |
| Penicillin X | 0.01192 | 0.04391 | -0.5324 | 351.1007 | Lactams |
| Ibuprofen | 0.000148 | 0.002318 | -0.4143 | 207.1379 | Phenylpropanoic acids |
| Deoxydihydro-artemisinin | 1.11E-05 | 0.000447 | 0.3864 | 286.2011 | Dioxolopyrans |
| 2-Quinolinecarboxylic acid | 1.75E-05 | 0.000588 | 0.409 | 174.0551 | Quinolines and derivatives |
| Bergenin | 0.000252 | 0.003273 | -0.3172 | 370.113 | Benzene and substituted derivatives |
| Luteolin 4'-sulfate | 0.000183 | 0.001838 | -0.4346 | 364.9963 | Flavonoids |
| Tyrosyl-Valine | 2.84E-06 | 0.000199 | 0.3595 | 313.1758 | Carboxylic acids and derivatives |
| 1-Thiocarbamoyl-2-imidazolidinone | 1.28E-05 | 0.000492 | 0.45 | 128.028 | Azolidines |
| Pentanoic acid, 5-(dipentylamino)-5-oxo-4-((3-quinolinylcarbonyl)amino)-, (R)- | 1.40E-06 | 0.00013 | -0.2471 | 442.2695 | Carboxylic acids and derivatives |
| N-Lauroyl Asparagine | 0.002084 | 0.0132 | -0.3722 | 353.1855 | Carboxylic acids and derivatives |
| Turanose | 9.14E-06 | 0.000274 | -0.3339 | 323.0977 | Fatty Acyls |
| Delapril | 8.27E-05 | 0.001618 | 0.3324 | 470.2646 | Peptidomimetics |
| P-Anisic Acid | 2.06E-05 | 0.00045 | -0.2826 | 151.0389 | Benzene and substituted derivatives |
| PE(20:2/0:0) | 5.89E-06 | 0.000202 | -0.3408 | 504.3081 | |
| Indole-3-acetyl-myo-inositol | 0.001311 | 0.007312 | -0.4596 | 382.1136 | Indoles and derivatives |
| Filipin II | 1.86E-05 | 0.000424 | -0.3056 | 637.394 | Macrolides and analogues |
| S-(1,2-Dicarboxyethyl)glutathione | 0.000939 | 0.005778 | -0.4181 | 404.0798 | Carboxylic acids and derivatives |
| 4-Hydroxy-6-octenoylcarnitine | 4.71E-06 | 0.000274 | 0.2725 | 302.1961 | Fatty Acyls |
| Soyasaponin II | 0.007013 | 0.02352 | -0.3594 | 957.5041 | Prenol lipids |
| Estriol | 5.54E-07 | 7.51E-05 | 0.2684 | 311.1601 | Steroids and steroid derivatives |
| Shoyuflavone B | 0.00029 | 0.002518 | 0.3763 | 437.0251 | Isoflavonoids |

| | | | | | |
|---|---|---|---|---|---|
| Benzyl sulfate | 2.73E-05 | 0.000527 | -0.3937 | 233.0119 | Benzene and substituted derivatives |
| 2,3-Dihydro-3-hydroxy-6-methoxy-2,2-dimethyl-4H-1-benzopyran-4-one | 2.03E-07 | 4.83E-05 | 0.2664 | 240.1231 | Benzopyrans |
| Dopamine quinone | 0.003146 | 0.01743 | -0.4181 | 134.0602 | Organooxygen compounds |
| 4-Methylbenzoic Acid | 0.001432 | 0.007779 | -0.4725 | 135.044 | Benzene and substituted derivatives |
| 9-Tetradecenoic acid | 1.81E-06 | 0.000148 | 0.2409 | 271.1651 | Fatty Acyls |
| 4-Chloro-2-nitrobenzylalcohol | 0.006519 | 0.02895 | -0.3908 | 587.2858 | Benzoxazoles |
| Cyclotricuspidogenin C | 3.04E-05 | 0.000569 | 0.3099 | 581.3692 | Organooxygen compounds |
| 2,6-Dimethoxy-1,4-benzoquinone | 0.000883 | 0.005529 | -0.2939 | 149.0234 | Organooxygen compounds |
| Tolmetin | 0.0002 | 0.002759 | 0.339 | 275.1389 | Organooxygen compounds |
| Fludrocortisone acetate | 0.000334 | 0.003946 | 0.3686 | 486.2232 | Steroids and steroid derivatives |
| 5-Hydroxytryptophol sulfate | 9.86E-06 | 0.000289 | -0.403 | 292.979 | Organic sulfuric acids and derivatives |
| 3-Pentadecylphenol | 3.56E-05 | 0.000915 | 0.3331 | 327.2681 | Phenols |
| Tiglic acid | 0.001263 | 0.007142 | 0.5741 | 299.1495 | Fatty Acyls |
| Aminosalicylic Acid | 0.000311 | 0.00378 | -0.3384 | 307.0923 | Benzene and substituted derivatives |
| 3-Hydroxy-cis-5-octenoylcarnitine | 1.14E-05 | 0.000319 | 0.3758 | 300.1811 | Fatty Acyls |
| Alpha-Trisaccharide | 0.002974 | 0.01683 | 0.2891 | 579.2395 | Organooxygen compounds |
| Sorgolactone | 0.000136 | 0.00152 | 0.4125 | 315.124 | Prenol lipids |
| 1,11-Undecanedicarboxylic acid | 2.56E-06 | 0.000188 | 0.3129 | 308.1857 | Fatty Acyls |
| N-Lauroylsarcosine | 7.60E-05 | 0.001526 | 0.3516 | 272.2219 | Carboxylic acids and derivatives |
| L-N-(1H-Indol-3-ylacetyl)aspartic acid | 6.15E-07 | 5.70E-05 | 0.3046 | 335.0879 | Carboxylic acids and derivatives |
| S-(2-Methylbutanoyl)-dihydrolipoamide | 0.000738 | 0.006554 | -0.3015 | 274.1298 | Fatty Acyls |
| 2-Methoxy-N-[3-[4-[3-methyl-4-[(6-methyl-3-pyridinyl)oxy]anilino]-6-quinazolinyl]prop-2-enyl]acetamide | 3.93E-05 | 0.000668 | -0.3487 | 504.1763 | Diazanaphthalenes |
| Isopropyl beta-D-glucoside | 3.10E-05 | 0.000848 | 0.3323 | 264.1439 | Organooxygen compounds |
| LysoPC(0:0/20:4(5Z,8Z,11Z,14Z)) | 0.001253 | 0.007103 | -0.3295 | 588.3293 | Glycerophospholipids |
| 1-Tert-Butyl 4-ethyl 3-oxopiperidine-1,4-dicarboxylate | 0.000184 | 0.002662 | 0.3327 | 335.16 | Piperidines |
| (2R,4S)-1-Tert-Butyl 2-methyl 4-aminopyrrolidine-1,2-dicarboxylate | 9.61E-07 | 7.14E-05 | 0.3427 | 265.1189 | Carboxylic acids and derivatives |
| Soyasapogenol E | 0.000335 | 0.003955 | -0.3035 | 421.3461 | Prenol lipids |
| Gamma-Glutamylcysteine | 8.39E-05 | 0.001632 | -0.3248 | 215.0485 | Carboxylic acids and derivatives |
| N-Palmitoyl Cysteine | 1.34E-05 | 0.000348 | -0.261 | 567.3163 | Fatty Acyls |
| 4,4'-DIMETHOXYDALBERGIONE | 7.38E-05 | 0.001488 | 0.3338 | 267.1014 | |
| Thalidomide | 3.24E-06 | 0.00014 | -0.2285 | 303.0617 | Isoindoles and derivatives |
| Etodolac | 4.95E-06 | 0.000281 | 0.3057 | 320.1855 | Indoles and derivatives |
| Petasitenine | 5.38E-05 | 0.001207 | 0.2881 | 346.1649 | Azaspirodecane derivatives |
| PS(14:0/LTE4) | 4.31E-05 | 0.001033 | 0.2801 | 468.2271 | |
| Salicylic Acid | 0.001338 | 0.009765 | -0.4268 | 121.0286 | Benzene and substituted derivatives |
| LysoPE(0:0/20:4(8Z,11Z,14Z,17Z)) | 7.03E-05 | 0.000975 | -0.2868 | 546.2796 | Glycerophospholipids |
| 3,4-Dihydroxymandelic Acid | 0.000285 | 0.002501 | -0.3806 | 165.0183 | Phenols |
| Lycoperoside D | 1.69E-06 | 0.000147 | 0.2609 | 740.4633 | Steroids and steroid derivatives |
| Tryptophol | 0.000768 | 0.006729 | 0.3537 | 386.1853 | Indoles and derivatives |
| 1-[(3R,4R,5R)-3-Fluoro-3,4-dihydroxy-5-(1-hydroxyethyl)oxolan-2-yl]pyrimidine-2,4-dione | 6.70E-06 | 0.000223 | -0.321 | 321.0724 | Organooxygen compounds |
| (E)-4,4'-(Hex-3-ene-3,4-diyl)bis(4,1-phenylene) bis(dihydrogen phosphate) | 7.51E-06 | 0.000242 | 0.2663 | 409.059 | Stilbenes |
| Echinacoside | 0.000359 | 0.002911 | -0.3081 | 767.2442 | Organooxygen compounds |
| Eupatilin | 9.81E-05 | 0.001768 | 0.3771 | 309.0762 | Flavonoids |

| | | | | | |
|---|---|---|---|---|---|
| PG(TXB2/16:1(9Z)) | 0.002362 | 0.01116 | -0.3239 | 871.4336 | |
| 2-Amino-3-methylbenzoate | 0.00042 | 0.004647 | 0.3673 | 303.1337 | Benzene and substituted derivatives |
| Araliasaponin II | 0.000127 | 0.001452 | -0.3156 | 471.2407 | Prenol lipids |
| 12S-HHT | 5.09E-07 | 7.40E-05 | 0.2098 | 325.1757 | Fatty Acyls |
| 11-Hydroxyyohimbine | 0.003043 | 0.01706 | -0.3728 | 335.1752 | Yohimbine alkaloids |
| PI(6 keto-PGF1alpha/20:4(5Z,8Z,11Z,14Z)) | 0.002031 | 0.01304 | 0.327 | 498.2602 | |
| N-Acetyl-N-[(2S)-3-(1H-indol-3-yl)-2-[[2-(4-piperidin-1-ylpiperidin-1-yl)acetyl]amino]propyl]-2-methoxybenzamide | 0.000886 | 0.007449 | 0.3232 | 591.3706 | Indoles and derivatives |
| 21-Deoxycortisol | 0.000226 | 0.003002 | 0.289 | 385.1756 | Steroids and steroid derivatives |
| Pratenol A | 0.0121 | 0.04438 | -0.4418 | 324.0864 | Benzopyrans |
| Licoricidin | 0.009289 | 0.03716 | -0.3831 | 469.1966 | Isoflavonoids |
| 17-Ethynyl-16-fluoroestradiol | 0.002485 | 0.01481 | -0.3564 | 328.1815 | Steroids and steroid derivatives |
| Arginylglutamine | 1.61E-06 | 9.35E-05 | 0.3613 | 283.1548 | Carboxylic acids and derivatives |
| Indolelactic Acid | 2.63E-07 | 5.65E-05 | -0.2978 | 206.0813 | Indoles and derivatives |
| Phellopterin | 0.000125 | 0.001442 | 0.3695 | 345.0975 | Coumarins and derivatives |
| Gonyautoxin I | 0.000119 | 0.001387 | 0.3159 | 448.0268 | Saxitoxins, gonyautoxins, and derivatives |
| Ferreirin | 2.06E-05 | 0.00065 | 0.2757 | 320.1126 | Isoflavonoids |
| Heptenophos | 1.10E-09 | 1.17E-06 | -0.274 | 230.9962 | Organic phosphoric acids and derivatives |
| Cinncassiol C2 | 8.37E-05 | 0.001632 | -0.2638 | 347.185 | Prenol lipids |
| Arg Met Thr | 0.000275 | 0.003464 | -0.2739 | 407.2032 | |
| Monoglyceride citrate | 0.01217 | 0.04459 | -0.4707 | 284.095 | Carboxylic acids and derivatives |
| Glycerophosphocholine | 7.30E-05 | 0.001484 | -0.2279 | 258.1099 | Glycerophospholipids |
| Hernandulcin | 1.08E-05 | 0.00044 | 0.2727 | 278.2113 | Prenol lipids |
| 2-Hydroxyclomipramine | 0.000515 | 0.003799 | -0.3151 | 367.0959 | Benzazepines |
| Furanogermenone | 0.000835 | 0.007164 | -0.2626 | 487.2793 | Prenol lipids |
| 3-Ethyl-1,2-cyclopentanedione | 4.18E-07 | 4.50E-05 | 0.3275 | 107.049 | Organooxygen compounds |
| Cadabicine | 2.42E-05 | 0.000721 | 0.2553 | 468.2489 | Organooxygen compounds |
| Pindolol | 0.003247 | 0.01783 | -0.3401 | 271.1398 | Indoles and derivatives |
| 4-Pentenoic acid, 2-fluoro-2-propyl- | 2.18E-05 | 0.000462 | -0.298 | 365.1811 | Fatty Acyls |
| (1R,6R)-6-hydroxy-2-succinylcyclohexa-2,4-diene-1-carboxylate | 0.00529 | 0.01907 | -0.4191 | 239.0555 | Keto acids and derivatives |
| 2,3-Bis(3-hydroxybenzyl)butane-1,4-diol | 3.44E-05 | 0.000608 | 0.3334 | 301.144 | Dibenzylbutane lignans |
| Coclaurine | 0.000234 | 0.002186 | 0.312 | 284.1287 | Isoquinolines and derivatives |
| Limonoate a-ring-lactone | 7.18E-05 | 0.000984 | -0.2683 | 523.1743 | Prenol lipids |
| Juzirine | 0.004415 | 0.01684 | -0.3698 | 326.1027 | Isoquinolines and derivatives |
| N-(2-Hydroxypropyl)valine | 2.35E-06 | 0.000179 | 0.216 | 176.1282 | Carboxylic acids and derivatives |
| 4a-Hydroxytetrahydrobiopterin | 6.84E-06 | 0.000344 | -0.2777 | 321.1291 | Pteridines and derivatives |
| 4-amino-4-deoxychorismate | 4.75E-08 | 2.32E-05 | 0.2917 | 190.05 | Carboxylic acids and derivatives |
| 1-[3,4-Dihydroxy-5-(hydroxymethyl)oxolan-2-yl]-5-hydroxyimidazole-4-carboxamide | 8.00E-06 | 0.000375 | 0.2729 | 259.0825 | Imidazole ribonucleosides and ribonucleotides |
| Tebipenem | 0.002749 | 0.01219 | -0.3642 | 404.0746 | Lactams |
| LysoPS(18:0/0:0) | 1.13E-05 | 0.000449 | -0.2391 | 570.2776 | Glycerophospholipids |
| Anonaine | 0.006149 | 0.02128 | -0.4087 | 310.108 | Aporphines |
| (E)-2-Methyl-2-buten-1-ol O-beta-D-Glucopyranoside | 3.00E-05 | 0.000568 | -0.296 | 293.1232 | Fatty Acyls |
| 2-(acetylamino)-1,5-anhydro-2-deoxy-3-O-b-D-galactopyranosyl-D-arabino-Hex-1-enitol | 0.002209 | 0.01368 | 0.3153 | 398.1654 | Organooxygen compounds |
| Tripdiolide | 1.25E-05 | 0.000333 | -0.2852 | 375.1442 | Oxepanes |

| | | | | | |
|---|---|---|---|---|---|
| 1-Amino-2-methylanthraquinone | 2.31E-05 | 0.000701 | 0.3009 | 260.0698 | Anthracenes |
| Trh-gly-lys | 0.009311 | 0.0372 | -0.3128 | 590.3026 | Carboxylic acids and derivatives |
| QUISQUALIC ACID | 0.004574 | 0.0226 | 0.3261 | 231.0723 | Carboxylic acids and derivatives |
| 5-Hydroxy-6-methoxyindole glucuronide | 0.003507 | 0.01436 | -0.3481 | 384.093 | Organooxygen compounds |
| N5-(4-Methoxybenzyl)glutamine | 0.01421 | 0.03948 | -0.4358 | 265.119 | Carboxylic acids and derivatives |
| (8R,9S,10S,13S,14S,17R)-17-Ethyl-3,17-dihydroxy-10,13-dimethyl-3,4,5,6,7,8,9,11,12,14,15,16-dodecahydro-1H-cyclopenta[a]phenanthren-2-one | 5.62E-05 | 0.001238 | 0.256 | 352.2843 | Steroids and steroid derivatives |
| Vanillic acid | 0.001022 | 0.006149 | -0.2904 | 167.0339 | Benzene and substituted derivatives |
| 2-Hydroxycarbamazepine | 0.01298 | 0.04672 | -0.4062 | 275.0774 | Morphinans |
| 4alpha-Methylzymosterol | 0.000725 | 0.006465 | -0.2716 | 421.3462 | Steroids and steroid derivatives |
| Kukoamine D | 1.91E-05 | 0.00062 | 0.2388 | 513.3068 | Phenols |
| Kynurenic Acid | 8.00E-06 | 0.000375 | 0.2589 | 190.0499 | Quinolines and derivatives |
| Estrone sulfate | 0.000101 | 0.001251 | 0.2484 | 331.1035 | Steroids and steroid derivatives |
| Oxyglutinosone | 5.20E-05 | 0.001183 | 0.2845 | 269.1746 | Organooxygen compounds |
| 6-Hydroxysandoricin | 0.000517 | 0.005279 | 0.2511 | 637.2864 | Carboxylic acids and derivatives |
| 4'-Hydroxyflavanone | 0.004016 | 0.02068 | 0.4058 | 241.0858 | Flavonoids |
| Therafectin | 4.37E-07 | 7.10E-05 | 0.2499 | 288.1807 | Organooxygen compounds |
| Chrysosplenetin | 0.000658 | 0.004491 | 0.4021 | 395.0738 | Flavonoids |
| Tetradec-5-Ynoic Acid | 0.000156 | 0.001664 | 0.3254 | 223.1695 | |
| 4-[(2,4-Dihydroxy-3,3-dimethylbutanoyl)amino]butanoic acid | 0.000102 | 0.001807 | 0.2427 | 234.1337 | Carboxylic acids and derivatives |
| Acetamidopropanal | 1.08E-07 | 2.01E-05 | 0.2476 | 229.1187 | Organooxygen compounds |
| Isopropamide | 0.000164 | 0.002495 | 0.2635 | 336.2531 | Benzene and substituted derivatives |
| Furofoline | 0.002195 | 0.01061 | 0.3322 | 575.1481 | Quinolines and derivatives |
| De-O-methylsimmondsin | 0.000125 | 0.001438 | 0.2493 | 406.1347 | Organooxygen compounds |
| Geosmin | 0.000163 | 0.001709 | 0.3512 | 181.1587 | Organooxygen compounds |
| LysoPA(22:5(7Z,10Z,13Z,16Z,19Z)/0:0) | 0.01165 | 0.03421 | -0.3782 | 529.2614 | Glycerophospholipids |
| 2,6-Dihydroxy-3-cyanopyridine | 0.000179 | 0.002618 | 0.2882 | 273.0617 | Pyridines and derivatives |
| 7-Acetylintermedine | 2.81E-05 | 0.000796 | 0.2664 | 355.1972 | |
| Fenofibric acid | 0.00069 | 0.004637 | -0.2723 | 353.033 | Benzene and substituted derivatives |
| Tazarotenic acid | 3.77E-05 | 0.000948 | 0.2356 | 346.0854 | Thiochromanes |
| Nufenoxole | 0.01102 | 0.04153 | -0.29 | 797.4513 | Benzene and substituted derivatives |
| 2-Furanmethanol | 1.58E-06 | 0.000141 | 0.2546 | 238.1074 | Heteroaromatic compounds |
| Mycorradicin | 0.006032 | 0.02746 | -0.2782 | 266.1387 | Fatty Acyls |
| Gibberellin A24 | 0.000146 | 0.001594 | -0.2739 | 345.17 | Prenol lipids |
| DIMBOA trihexose | 4.63E-07 | 7.17E-05 | 0.2308 | 231.1342 | Carboxylic acids and derivatives |
| 3,3'-Dihydroxy-4',5,7-trimethoxyflavan | 8.89E-06 | 0.000268 | 0.2226 | 331.1181 | Flavonoids |
| Tigloidine | 1.28E-06 | 0.000124 | 0.2845 | 265.1909 | Tropane alkaloids |
| Phthalide | 2.09E-07 | 4.86E-05 | 0.2605 | 307.0382 | Isocoumarans |
| Epigoitrin | 1.32E-06 | 8.41E-05 | -0.2786 | 128.0164 | Azolidines |
| Galactosyl 4-hydroxyproline | 1.97E-05 | 0.000633 | 0.2224 | 276.108 | Carboxylic acids and derivatives |
| 1-Aminocyclohexanecarboxylic acid | 0.001233 | 0.009215 | 0.348 | 176.1282 | Prenol lipids |
| 4-Hydroxy-5-phenyltetrahydro-1,3-oxazin-2-one | 6.56E-07 | 8.21E-05 | 0.1868 | 226.1075 | Benzene and substituted derivatives |
| Luvangetin | 8.76E-05 | 0.001136 | 0.3341 | 257.0814 | |
| Gibberellin A51-catabolite | 0.000604 | 0.005775 | -0.2299 | 702.3302 | Prenol lipids |

| | | | | | |
|---|---|---|---|---|---|
| 15-Hydroxynorandrostene-3,17-dione glucuronide | 0.004908 | 0.01811 | -0.3319 | 463.1941 | Steroids and steroid derivatives |
| Cer(d18:2(4E,14Z)/18:1(9Z)-O(12,13)) | 0.000329 | 0.003911 | 0.2427 | 639.5068 | |
| Soyasaponin III | 0.0081 | 0.03366 | -0.1994 | 797.4679 | Prenol lipids |
| Difructose anhydride III | 1.80E-06 | 0.000101 | -0.2238 | 323.0979 | Organooxygen compounds |
| Isoamericanol A | 0.000803 | 0.005167 | 0.3029 | 375.1078 | Benzodioxanes |
| Pectolinarin | 0.006038 | 0.02101 | 0.3322 | 603.1763 | Tannins |
| Indoleacetaldehyde | 0.000612 | 0.00581 | 0.2842 | 382.1541 | Indoles and derivatives |
| Milbemycin A3 | 1.82E-06 | 0.000148 | 0.1975 | 511.3029 | Macrolides and analogues |
| Sinapic Acid | 0.000664 | 0.004514 | -0.3146 | 223.0604 | Cinnamic acids and derivatives |
| Lithocholate 3-O-glucuronide | 4.38E-06 | 0.000168 | -0.2049 | 551.3213 | Steroids and steroid derivatives |
| Phthalimidinoglutarimide | 0.008299 | 0.02672 | -0.3395 | 289.0826 | Isoindoles and derivatives |
| Acetylshikonin | 0.000731 | 0.006509 | 0.2228 | 348.1439 | Naphthalenes |
| Nelfinavir | 0.01565 | 0.04236 | -0.352 | 588.287 | Carboxylic acids and derivatives |
| Azacitidine | 9.33E-05 | 0.001724 | -0.2374 | 506.192 | Organooxygen compounds |
| Alliospiroside C | 0.000926 | 0.007661 | -0.2089 | 769.3724 | Steroids and steroid derivatives |
| Benazeprilat | 0.001274 | 0.009452 | -0.2372 | 397.1765 | Carboxylic acids and derivatives |
| Beraprost | 0.000779 | 0.006802 | 0.2955 | 437.1763 | Fatty Acyls |
| Alpha-Hydroxy-1-methyl-1H-indole-3-propanoic acid | 0.004024 | 0.02071 | -0.2831 | 252.123 | Indoles and derivatives |
| 2-Methoxyacetaminophen sulfate | 0.000746 | 0.004895 | 0.2757 | 242.0123 | Organic sulfuric acids and derivatives |
| Tropolone | 6.02E-06 | 0.000316 | 0.2104 | 164.0706 | Tropones |
| 4-Hydroxyphenylacetaldehyde | 0.01206 | 0.04428 | -0.3054 | 311.0694 | Benzene and substituted derivatives |
| Desfuroyl Ceftiofur | 0.000462 | 0.003522 | 0.3225 | 474.0242 | Lactams |
| (3R,5S,6R,7R,8S,9S,10S,13R,14S,17R)-6-Ethyl-17-((R)-4-hydroxybutan-2-yl)-10,13-dimethylhexadecahydro-1H-cyclopenta[a]phenanthrene-3,7-diol | 0.000124 | 0.001431 | 0.2316 | 391.3208 | Steroids and steroid derivatives |
| 4-Hydroxyestradiol | 7.13E-07 | 8.48E-05 | 0.2101 | 311.1599 | Steroids and steroid derivatives |
| Cucurbic acid | 1.08E-05 | 0.00044 | 0.2484 | 177.1275 | Fatty Acyls |
| Risbitin | 8.15E-05 | 0.001078 | 0.2699 | 443.3156 | Organooxygen compounds |
| PE(20:3/0:0) | 0.004271 | 0.01647 | -0.2676 | 502.2924 | |
| 3-Phenylpropyl 2-methylpropanoate | 5.12E-06 | 0.000285 | 0.2136 | 248.1645 | Benzene and substituted derivatives |
| HDMBOA-Glc | 0.000737 | 0.004857 | -0.2176 | 368.098 | Organooxygen compounds |
| Rhizopterin | 0.001054 | 0.00628 | 0.3106 | 339.0839 | |
| 3,4-Dihydroxyphenylacetaldehyde | 0.000117 | 0.001369 | -0.2716 | 363.108 | Benzene and substituted derivatives |
| Histidinal | 0.001391 | 0.009986 | 0.3543 | 140.0819 | Organonitrogen compounds |
| 2,3-dihydrobenzofuran | 0.007976 | 0.03331 | -0.2067 | 121.065 | Coumarans |
| Caffeic Acid | 6.68E-08 | 1.54E-05 | -0.2603 | 179.034 | Cinnamic acids and derivatives |
| Manzamine A | 1.24E-05 | 0.000333 | -0.181 | 569.3316 | Harmala alkaloids |
| 4a-Formyl-5a-cholesta-8,24-dien-3b-ol | 6.30E-05 | 0.000906 | 0.257 | 457.3312 | Steroids and steroid derivatives |
| Alpha-Elemolic acid | 0.007102 | 0.03063 | -0.2476 | 439.3564 | Prenol lipids |
| 6''-O-Malonyldaidzin | 0.000555 | 0.005487 | -0.2391 | 535.1443 | Coumarins and derivatives |
| Asp Ile Val | 6.75E-05 | 0.001413 | -0.2307 | 346.1971 | |
| THYMOPENTIN | 0.004047 | 0.01587 | -0.2693 | 716.3091 | Carboxylic acids and derivatives |
| Aziridyl benzoquinone | 0.005164 | 0.02458 | 0.3477 | 321.1438 | Organooxygen compounds |
| LysoPC(20:3(8Z,11Z,14Z)/0:0) | 0.001234 | 0.007034 | -0.2213 | 590.3448 | Glycerophospholipids |
| N-(3-Hydroxypropyl)valine | 4.82E-07 | 7.35E-05 | 0.1924 | 176.1282 | Carboxylic acids and derivatives |

| | | | | | |
|---|---|---|---|---|---|
| 4-Hydroxyproline galactoside | 0.000105 | 0.001828 | 0.1658 | 276.1077 | Fatty Acyls |
| L-a-Lysophosphatidylserine | 2.86E-06 | 0.000129 | -0.1689 | 524.2975 | |
| Cinobufotalin | 5.22E-05 | 0.001183 | 0.1904 | 497.1976 | Steroids and steroid derivatives |
| Hexobarbital | 0.01129 | 0.03337 | -0.3711 | 273.0625 | Diazines |
| 1-O-all-trans-retinoyl-beta-glucuronic Acid | 0.002356 | 0.01429 | -0.25 | 509.2759 | Prenol lipids |
| (R)-2-(4-(Tert-butoxycarbonyl)morpholin-3-yl)acetic acid | 6.12E-06 | 0.000319 | 0.2361 | 210.1125 | Oxazinanes |
| Tuliposide A | 0.000755 | 0.006658 | 0.255 | 261.0964 | Organooxygen compounds |
| Paricalcitol | 3.04E-07 | 3.76E-05 | 0.1908 | 437.3013 | Steroids and steroid derivatives |
| Deoxythioguanosine | 4.70E-06 | 0.000274 | -0.1727 | 248.0588 | Purine nucleosides |
| Ursolic Acid | 0.009826 | 0.03862 | -0.2904 | 457.3671 | Prenol lipids |
| Gardenin B | 0.001762 | 0.00908 | 0.3029 | 339.0873 | Flavonoids |
| Prephenate | 1.44E-05 | 0.000362 | 0.2657 | 271.0468 | Keto acids and derivatives |
| Midecamycin acetate | 0.002196 | 0.01363 | -0.2039 | 460.7345 | Organooxygen compounds |
| N-(N-(3-Carboxyoxirane-2-carbonyl)leucyl)isoamylamine | 0.000215 | 0.002891 | 0.2354 | 356.2178 | Carboxylic acids and derivatives |
| PE(22:1/0:0) | 0.000108 | 0.001307 | -0.2329 | 534.3548 | |
| PA(18:3(6Z,9Z,12Z)/PGJ2) | 0.004959 | 0.01824 | -0.2409 | 783.4004 | |
| Aflatoxin B1 dialcohol | 0.00019 | 0.002693 | 0.168 | 348.1439 | Benzene and substituted derivatives |
| (2S)-2-(Diaminomethylideneamino)-3-phenylpropanoic acid | 5.87E-06 | 0.000202 | 0.2879 | 188.0818 | Carboxylic acids and derivatives |
| Thymine dimer | 0.0018 | 0.009189 | 0.3874 | 273.0625 | Diazines |
| 1-Heptadecanoylglycerophosphoethanolamine | 0.005637 | 0.01995 | -0.2457 | 512.2977 | Glycerophospholipids |
| 4-Chlorophenylacetic acid | 0.006816 | 0.02297 | 0.385 | 169.0067 | Benzene and substituted derivatives |
| LysoPC(0:0/18:0) | 0.000144 | 0.002287 | -0.1749 | 524.3709 | Glycerophospholipids |
| N-((Hexahydro-1-azepinyl)carbonyl)-leucyl(1-methyl)-tryptophyl-tryptophan | 0.001861 | 0.00941 | -0.2495 | 600.3233 | Carboxylic acids and derivatives |
| Alpha-dihydroartemisinin | 1.86E-06 | 0.000101 | 0.2229 | 283.1546 | Prenol lipids |
| 2-(beta-D-Mannopyranosyl)-L-tryptophan | 1.08E-05 | 0.00044 | -0.1625 | 367.15 | Pyridines and derivatives |
| Frangulanine | 0.001027 | 0.008129 | -0.2152 | 545.3092 | Carboxylic acids and derivatives |
| Taxine B | 1.07E-05 | 0.00044 | -0.1751 | 548.2955 | Prenol lipids |
| Lucuminic acid | 0.000253 | 0.002301 | -0.2092 | 445.1341 | Organooxygen compounds |
| Aldosterone | 0.000751 | 0.004926 | -0.2329 | 395.1617 | Steroids and steroid derivatives |
| Phloretin | 0.000237 | 0.002201 | -0.2775 | 273.0766 | Organooxygen compounds |
| LysoPC(18:0/0:0) | 8.49E-05 | 0.00111 | -0.1915 | 568.3605 | Glycerophospholipids |
| Dihydroorotic Acid | 0.004927 | 0.01815 | 0.3248 | 157.0244 | Carboxylic acids and derivatives |
| Sativan | 2.21E-05 | 0.000464 | 0.1956 | 331.118 | Isoflavonoids |
| Anb-nos | 0.005469 | 0.01953 | -0.2514 | 304.0329 | Benzene and substituted derivatives |
| Isokobusone | 0.000715 | 0.006445 | 0.2611 | 264.1957 | Organooxygen compounds |
| Indole-3-carboxilic acid-O-sulphate | 0.01932 | 0.04936 | -0.3694 | 239.9966 | Indoles and derivatives |
| (Z)-Resveratrol 4'-glucoside | 0.000682 | 0.006229 | 0.1911 | 413.1204 | Stilbenes |
| 1-(beta-D-Glucopyranosyloxy)-3-octanone | 7.51E-06 | 0.000367 | -0.1748 | 345.1306 | Fatty Acyls |
| Glu-Ile-Leu-Asp-Val | 0.005995 | 0.0209 | -0.276 | 586.3079 | Peptidomimetics |
| (2R)-2-Acetamido-6-hydroxy-2-(sulfanylmethyl)hex-3-enoic acid | 0.00043 | 0.003333 | 0.3049 | 254.0487 | Carboxylic acids and derivatives |
| 3b-Hydroxy-17-(1h-1,2,3-triazol-1-yl)androsta-5,16-diene | 0.004134 | 0.0211 | -0.243 | 362.2177 | Steroids and steroid derivatives |
| Cilastatin | 3.36E-05 | 0.0006 | -0.2042 | 403.1532 | Carboxylic acids and derivatives |
| Dihydrojasmonic acid | 8.07E-07 | 9.05E-05 | 0.168 | 447.2717 | Fatty Acyls |
| Milbemycin alpha9 | 0.004832 | 0.01791 | -0.2529 | 674.2773 | Macrolides and analogues |

| | | | | | |
|---|---|---|---|---|---|
| D-Digitoxose | 2.04E-06 | 0.00016 | 0.186 | 297.1518 | Organooxygen compounds |
| 6-Thioxanthylic acid | 0.01419 | 0.03947 | -0.3328 | 379.012 | Purine nucleosides |
| (S)-(-)-Perillyl alcohol | 6.23E-07 | 8.00E-05 | 0.1747 | 135.117 | Prenol lipids |
| Verbascose | 0.000273 | 0.002437 | -0.188 | 809.2553 | Organooxygen compounds |
| Polygonal | 4.41E-06 | 0.000168 | 0.2192 | 267.1596 | Organooxygen compounds |
| Hordatine B | 0.008188 | 0.03391 | 0.2098 | 545.2926 | 2-arylbenzofuran flavonoids |
| 4-Methyl-2-oxo-2H-chromen-7-yl sulfamate | 0.000126 | 0.001445 | 0.2628 | 275.9932 | Coumarins and derivatives |
| Edotecarin | 0.003012 | 0.01287 | 0.2362 | 589.1608 | Phenol ethers |
| (R)-2-Hydroxysterculic acid | 2.12E-05 | 0.000659 | 0.1989 | 328.2844 | Fatty Acyls |
| Metabolite A | 0.00012 | 0.001401 | -0.1948 | 815.3901 | Prenol lipids |
| Practolol | 3.64E-05 | 0.000922 | 0.1692 | 555.3177 | Benzene and substituted derivatives |
| 2-(Arabinosylamino)-3-(glucosylamino)propanenitrile | 0.000125 | 0.001438 | -0.2252 | 360.1406 | Organooxygen compounds |
| Bradykinin | 0.01045 | 0.04017 | 0.2095 | 541.7773 | Carboxylic acids and derivatives |
| Contignasterol | 9.66E-06 | 0.000421 | 0.1435 | 473.3256 | Prenol lipids |
| 4-Nitrophenyl beta-D-xyloside | 0.006863 | 0.02995 | 0.2766 | 236.0553 | Organooxygen compounds |
| 2-Nonene-1,4-diol | 3.50E-05 | 0.000616 | 0.234 | 203.128 | Fatty Acyls |
| Gemeprost | 1.20E-05 | 0.000465 | 0.1767 | 359.2579 | Fatty Acyls |
| Sulindac sulfide | 1.09E-05 | 0.00031 | -0.2062 | 361.0705 | Indenes and isoindenes |
| Vulgarin | 0.000218 | 0.002078 | 0.2311 | 285.1127 | Prenol lipids |
| 7-Hydroxyetodolac | 0.000103 | 0.001807 | 0.1996 | 286.1439 | Indoles and derivatives |
| Picrocrocin | 6.43E-05 | 0.000919 | -0.2029 | 365.1353 | Organooxygen compounds |
| (23S,24S)-17,23-Epoxy-24,29-dihydroxy-27-norlanost-8-ene-3,15-dione | 6.30E-05 | 0.001344 | 0.1641 | 455.315 | Prenol lipids |
| LysoPC(17:0/0:0) | 0.001141 | 0.006651 | -0.2117 | 554.3446 | Glycerophospholipids |
| (+/-)-3',4'-Methylenedioxy-5,7-dimethylepicatechin | 0.001126 | 0.008653 | 0.2063 | 331.1174 | Flavonoids |
| Veliparib | 2.90E-05 | 0.000813 | 0.1946 | 262.1648 | Benzimidazoles |
| Norepinephrine | 0.007859 | 0.02565 | -0.2877 | 150.0549 | Phenols |
| 4-Bis(2-hydroxyethyl)amino-L-phenylalanine | 0.000323 | 0.003856 | 0.2209 | 269.1496 | Carboxylic acids and derivatives |
| 5,6-epoxy,18R-HEPE | 4.90E-05 | 0.001135 | 0.1888 | 355.1866 | Fatty Acyls |
| PE(22:6(4Z,7Z,10Z,13E,15E,19Z)-OH(17)/22:6(4Z,7Z,10Z,13Z,16Z,19Z)) | 0.006935 | 0.03017 | 0.206 | 448.7455 | |
| Isomugineic acid | 6.43E-06 | 0.00033 | -0.1746 | 362.1557 | Carboxylic acids and derivatives |
| Cyasterone | 1.11E-06 | 7.69E-05 | 0.1718 | 519.2956 | Prenol lipids |
| 3,7,8,15-Scirpenetetrol | 0.000709 | 0.006393 | 0.2104 | 362.1596 | Prenol lipids |
| DOPA sulfate | 0.01702 | 0.04493 | -0.4102 | 276.0177 | Carboxylic acids and derivatives |
| Algesterone acetophenide | 0.000573 | 0.005604 | 0.1843 | 471.2534 | Steroids and steroid derivatives |
| (2E)-Oct-2-enedioylcarnitine | 0.00292 | 0.01661 | 0.2516 | 315.1659 | Fatty Acyls |
| 14-Deoxy-11,12-Didehydroandrographolide | 1.01E-05 | 0.000295 | -0.174 | 377.1962 | Prenol lipids |
| [4-[4-(4-Hydroxyphenyl)hex-3-en-3-yl]phenyl] phosphono hydrogen phosphate | 0.008272 | 0.02665 | 0.2598 | 409.059 | Stilbenes |
| Cerasinone | 3.14E-06 | 0.000138 | 0.1448 | 329.1026 | Flavonoids |
| Lactucin | 4.65E-06 | 0.000174 | -0.1844 | 257.0814 | Lactones |
| Valtrate | 0.000447 | 0.004856 | -0.2131 | 177.0547 | Prenol lipids |
| Alongside | 0.01149 | 0.04275 | -0.2268 | 266.1387 | Macrolides and analogues |
| All-trans-18-Hydroxyretinoic acid | 0.000195 | 0.001918 | -0.172 | 361.2014 | Prenol lipids |
| (S)-Nerolidol 3-O-[a-L-rhamnopyranosyl-(1->2)-b-D-glucopyranoside] | 8.65E-05 | 0.001662 | 0.1698 | 266.1625 | Fatty Acyls |
| Combretastatin A4 | 6.55E-06 | 0.000219 | 0.1572 | 315.1233 | Stilbenes |

| | | | | | |
|---|---|---|---|---|---|
| LysoPC(20:5(5Z,8Z,11Z,14Z,17Z)/0:0) | 0.004667 | 0.02288 | -0.2022 | 542.3237 | Glycerophospholipids |
| Carbamoyl cholesterol | 0.000201 | 0.002771 | 0.1828 | 394.3425 | Steroids and steroid derivatives |
| Nanaomycin | 3.03E-05 | 0.000568 | 0.171 | 301.0712 | |
| Hymenoxon | 0.000211 | 0.00286 | -0.1794 | 265.1434 | Oxanes |
| (1R)-1-Amino-2-sulfanylethanesulfinic acid | 2.02E-07 | 2.89E-05 | 0.1542 | 139.9833 | Sulfinic acids and derivatives |
| 10-Hydroxy-2-oxabicyclo[6.2.2]dodeca-1(10),8,11-trien-3-one | 4.71E-06 | 0.000274 | 0.1763 | 210.1125 | Phenols |
| Nitroethane | 0.002872 | 0.01245 | -0.2584 | 149.0565 | Allyl-type 1,3-dipolar organic compounds |
| PS(20:5(5Z,8Z,11Z,14Z,16E)-OH(18R)/22:5(4Z,7Z,10Z,13Z,16Z)) | 0.004339 | 0.02177 | -0.1719 | 447.75 | |
| 3'-Deoxythymidine-5'-monophosphate | 0.000206 | 0.002 | 0.1897 | 341.0326 | Pyrimidine nucleotides |
| Astragaloside III | 0.008144 | 0.02636 | -0.1733 | 765.4412 | Steroids and steroid derivatives |
| PC(17:0/0:0) | 0.000157 | 0.002421 | -0.1357 | 532.3372 | Glycerophospholipids |
| Boviquinone 4 | 0.001033 | 0.006184 | -0.1979 | 393.2403 | Prenol lipids |
| Aclacinomycin N | 0.00424 | 0.02143 | -0.1894 | 846.3835 | Anthracyclines |
| 6-Hydroxy-4-methoxy-3-(3-methyl-2-butenyl)-2-(2-phenylethenyl)benzoic acid | 1.34E-07 | 3.85E-05 | -0.1568 | 361.1424 | Stilbenes |
| Cannabinol | 0.006319 | 0.02836 | 0.2477 | 333.1808 | Benzopyrans |
| KOBUSONE | 1.33E-05 | 0.000497 | 0.1547 | 223.1693 | Organooxygen compounds |
| (10Z,14E,16E)-10,14,16-Octadecatrien-12-ynoic acid | 2.83E-06 | 0.000199 | 0.1493 | 297.1807 | Fatty Acyls |
| (2R)-2-{[(9Z)-Hexadec-9-enoyl]oxy}-3-hydroxypropyl 2-(trimethylazaniumyl)ethyl phosphate | 0.000141 | 0.001565 | -0.1584 | 538.3135 | Glycerophospholipids |
| Aspartyl-Tryptophan | 0.000227 | 0.003002 | -0.1769 | 320.124 | Carboxylic acids and derivatives |
| Lysyltryptophan | 2.29E-05 | 0.000472 | 0.2138 | 353.1575 | Carboxylic acids and derivatives |
| Delpazolid | 3.32E-05 | 0.0006 | 0.2159 | 329.1056 | Benzene and substituted derivatives |
| Eptifibatide | 0.00444 | 0.01689 | 0.2588 | 812.2928 | Carboxylic acids and derivatives |
| Splitomicin | 0.01305 | 0.0469 | -0.2784 | 216.1021 | Naphthopyrans |
| Murabutida | 0.000277 | 0.002456 | 0.187 | 529.2461 | Carboxylic acids and derivatives |
| Imidazolepropionic acid | 1.05E-05 | 0.000301 | -0.1897 | 139.0501 | Azoles |
| 2-(3-(2-Aminoethyl)-1h-indol-5-yloxy)acetyl-l-tyrosyl-glycinamide | 0.002944 | 0.01264 | 0.2788 | 474.1718 | Carboxylic acids and derivatives |
| (S)-N-((S)-1-Amino-3,3-dimethyl-1-oxobutan-2-yl)-2-((S)-2-mercapto-4-(3,4,4-trimethyl-2,5-dioxoimidazolidin-1-yl)butanamido)-N,4-dimethylpentanamide | 0.01342 | 0.04778 | -0.2186 | 499.2872 | Peptidomimetics |
| Malonylcarnitine | 4.80E-06 | 0.000277 | -0.1673 | 289.1394 | Fatty Acyls |
| Fructosyl valine | 1.47E-06 | 0.000134 | -0.1291 | 280.1389 | Carboxylic acids and derivatives |
| PI(20:4(8Z,11Z,14Z,17Z)-2OH(5S,6R)/22:2(13Z,16Z)) | 0.00976 | 0.03843 | -0.2221 | 508.2744 | |
| (Carbamoylamino) (2R)-2,5-diaminopentanoate | 2.44E-05 | 0.000724 | 0.2559 | 190.1075 | Carboxylic acids and derivatives |
| Riddelliine | 0.000134 | 0.001503 | 0.1713 | 386.1025 | |
| Gibberellin A37 | 0.000213 | 0.002883 | -0.1691 | 379.211 | Prenol lipids |
| 2-[4-(sulfooxy)phenyl]acetic acid | 3.69E-06 | 0.000152 | 0.1365 | 230.9963 | Organic sulfuric acids and derivatives |
| Decarbamoylneosaxitoxin | 3.57E-05 | 0.000622 | 0.1785 | 317.1236 | Saxitoxins, gonyautoxins, and derivatives |
| N-[(3s)-2-Oxotetrahydrofuran-3-Yl]butanamide | 7.16E-05 | 0.000983 | 0.1649 | 216.087 | Carboxylic acids and derivatives |
| Indole-3-carboxylic acid | 0.002688 | 0.01564 | 0.258 | 144.0446 | Indoles and derivatives |
| N-Acetyl-DL-Phenylalanine | 3.70E-05 | 0.000636 | -0.1905 | 206.0814 | Carboxylic acids and derivatives |
| PI(20:3(5Z,8Z,11Z)/20:3(8Z,11Z,14Z)-2OH(5,6)) | 0.01671 | 0.04438 | -0.2152 | 979.4986 | |
| N-Acetylsphinganine | 0.006809 | 0.0298 | -0.2012 | 344.3158 | Sphingolipids |
| M-Coumaric acid | 0.000671 | 0.004546 | 0.1731 | 327.0868 | Cinnamic acids and derivatives |
| Ureidosuccinic Acid | 0.005307 | 0.0191 | 0.2407 | 175.035 | Carboxylic acids and derivatives |
| Perulactone B | 0.006278 | 0.02159 | -0.2351 | 533.2715 | Steroids and steroid derivatives |

| | | | | | |
|---|---|---|---|---|---|
| Dibutylphthalic acid | 0.000595 | 0.005733 | 0.2314 | 311.1827 | Benzene and substituted derivatives |
| Hydroxypelenolide | 1.37E-05 | 0.000506 | 0.1324 | 235.1692 | Prenol lipids |
| 9,10-DHOME | 3.41E-05 | 0.00089 | 0.1464 | 297.2423 | Fatty Acyls |
| Rohitukine | 0.002218 | 0.01369 | -0.1676 | 323.16 | Piperidines |
| 7-a,25-Dihydroxycholesterol | 0.000522 | 0.003835 | 0.155 | 439.3166 | Prenol lipids |
| (R)-Menthone 8-thioacetate | 0.000249 | 0.002283 | -0.1825 | 249.0948 | Prenol lipids |
| CI-1044 | 0.000915 | 0.005663 | -0.1825 | 378.1371 | Carboxylic acids and derivatives |
| Tetrahydrocortisone | 5.04E-05 | 0.00116 | 0.1569 | 365.23 | Steroids and steroid derivatives |
| Gamma-Linolenic acid | 0.004058 | 0.02084 | 0.2243 | 296.2583 | Fatty Acyls |
| Sesamolinol 4'-O-b-D-glucosyl (1->6)-O-b-D-glucoside | 0.004076 | 0.01594 | -0.1841 | 677.2128 | Organooxygen compounds |
| Vanillylmandelic acid | 0.000166 | 0.00251 | -0.181 | 181.0497 | Phenol ethers |
| 2-Aminobenzylstatine | 6.50E-05 | 0.001382 | 0.168 | 298.2125 | Carboxylic acids and derivatives |
| Calcifediol lactone | 1.95E-06 | 0.000103 | 0.1437 | 473.2898 | Steroids and steroid derivatives |
| (-)-Fumigaclavine B | 1.84E-06 | 0.000101 | -0.1612 | 293.1058 | Carboxylic acids and derivatives |
| PC(18:0/0:0) | 7.36E-05 | 0.001487 | -0.0968 | 524.3708 | Glycerophospholipids |
| 5-(2-Hydroxyethyl)-4-methylthiazole | 0.00201 | 0.01299 | 0.1807 | 144.0479 | Azoles |
| Unknown 370 | 6.95E-05 | 0.000966 | 0.1537 | 457.2947 | Prenol lipids |
| 12-Oxo-2,3-dinor-10,15-phytodienoic acid | 0.006931 | 0.03017 | 0.2096 | 247.1693 | Fatty Acyls |
| Asp Ile Leu | 5.41E-06 | 0.000296 | -0.1549 | 360.2127 | |
| Agmatine | 0.000356 | 0.004148 | -0.1998 | 131.1293 | Organonitrogen compounds |
| Dimethisterone | 6.81E-07 | 8.41E-05 | 0.1362 | 341.2474 | Steroids and steroid derivatives |
| 4-Oxo-1,4-dihydroquinoline-3-carboxylic acid | 8.29E-06 | 0.000378 | 0.1153 | 190.05 | Quinolines and derivatives |
| 14-oxo-DoHE(1-) | 0.00045 | 0.00486 | 0.1878 | 381.1843 | Fatty Acyls |
| GalNAc-beta1->4Gal-beta1->4Glc-beta1->1'Cer | 0.01013 | 0.03931 | -0.1749 | 446.2337 | Sphingolipids |
| Ala Gly Leu Val Ser | 0.002508 | 0.01167 | -0.2009 | 444.2452 | |
| Alpha-Cyperol | 0.004738 | 0.0231 | -0.1923 | 203.1796 | Prenol lipids |
| 2-Feruloyl-1-sinapoylgentiobiose | 0.00499 | 0.01831 | -0.1712 | 723.2184 | Cinnamic acids and derivatives |
| 7-Methylthioheptyl Glucosinolate | 0.000717 | 0.004762 | 0.1516 | 462.0911 | Organooxygen compounds |
| Scymnol | 4.15E-05 | 0.001011 | 0.141 | 433.3309 | Steroids and steroid derivatives |
| (1'R)-Nepetalic acid | 8.12E-06 | 0.000375 | 0.1413 | 202.1438 | Prenol lipids |
| 1-Methylcytosine | 0.000529 | 0.005366 | 0.1907 | 126.0664 | Diazines |
| Alpha-Bisabolol oxide A | 0.001387 | 0.007603 | -0.2066 | 283.1908 | Oxanes |
| Pentadecanoic acid | 0.000107 | 0.001853 | 0.1695 | 306.2426 | Fatty Acyls |
| 1-Oleoyl-2-{6-[(7-nitro-2-1,3-benzoxadiazol-4-yl)amino]hexanoyl}-sn-glycero-3-phospho-L-serine | 0.009971 | 0.03042 | -0.1795 | 844.3678 | |
| 4-hydroxysphinganine | 1.61E-05 | 0.000556 | 0.1178 | 381.311 | Organonitrogen compounds |
| 3,4,5,6-Tetrahydrohippuric acid | 0.004983 | 0.01829 | -0.1964 | 548.2565 | Carboxylic acids and derivatives |
| S-(3-Methylbutanoyl)-dihydrolipoamide-E | 0.000657 | 0.006094 | -0.1504 | 291.1338 | Fatty Acyls |
| Norchalciporyl propionate | 0.003766 | 0.0198 | -0.1926 | 536.3441 | Azepines |
| (Z)-3-Oxo-2-(2-pentenyl)-1-cyclopenteneacetic acid | 0.000854 | 0.005398 | -0.1988 | 253.1076 | Organooxygen compounds |
| 3-Hydroxybutyrylcarnitine | 0.002368 | 0.01433 | -0.1583 | 248.1492 | Fatty Acyls |
| Methionyl-Proline | 1.63E-05 | 0.00056 | 0.1543 | 247.1114 | Carboxylic acids and derivatives |
| Ilepatril | 0.001577 | 0.0109 | 0.1941 | 446.1818 | Carboxylic acids and derivatives |
| 13E-Tetranor-16-carboxy-LTE4 | 0.000596 | 0.004187 | -0.1743 | 394.132 | Carboxylic acids and derivatives |

| | | | | | |
|---|---|---|---|---|---|
| 2-Hydroxyethanesulfonate | 3.58E-08 | 9.13E-06 | -0.1482 | 124.9902 | Organic sulfonic acids and derivatives |
| 1,7-Dimethylguanosine | 3.20E-05 | 0.000863 | 0.1334 | 276.1077 | Purine nucleosides |
| 6''-O-Acetylglycitin | 0.000347 | 0.002853 | -0.1688 | 533.1285 | Isoflavonoids |
| Phenylalanylproline | 1.06E-05 | 0.00044 | 0.1231 | 295.1651 | Carboxylic acids and derivatives |
| 3-Demethylsimmondsin 2'-(Z)-ferulate | 0.004361 | 0.0167 | 0.217 | 572.1546 | Cinnamic acids and derivatives |
| Fumagillin | 8.39E-05 | 0.001102 | 0.137 | 495.1814 | Fatty Acyls |
| Zearalenone 4-sulfate | 0.004458 | 0.01693 | 0.2557 | 443.0996 | Macrolides and analogues |
| Malonic acid | 1.47E-05 | 0.000366 | -0.1843 | 207.0152 | Carboxylic acids and derivatives |
| 4-Amino-5-hydroxy-2,7-naphthalenedisulfonic acid | 0.004671 | 0.01752 | 0.186 | 317.9741 | Naphthalenes |
| Soyasaponin A1 | 0.01786 | 0.04653 | -0.1829 | 633.2935 | Prenol lipids |
| (6R)-Folinic acid | 0.002016 | 0.01301 | -0.1557 | 474.1729 | Pteridines and derivatives |
| Oroselone | 1.69E-07 | 4.57E-05 | 0.1773 | 244.0968 | Coumarins and derivatives |
| MeOSuc-Ala-Ala-Pro-Val-PNA | 0.000174 | 0.001779 | 0.1403 | 635.2707 | Carboxylic acids and derivatives |
| Strictosidine | 0.01456 | 0.04017 | -0.2241 | 575.2208 | Prenol lipids |
| Propafenone glucuronide | 0.000567 | 0.004047 | -0.1864 | 552.2013 | Linear 1,3-diarylpropanoids |
| L-Menthyl (R,S)-3-hydroxybutyrate | 0.000507 | 0.003752 | 0.205 | 287.1859 | Prenol lipids |
| (2R)-2-Acetamido-3-[[(2R)-2-acetamido-2-carboxyethyl]disulfanyl]propanoic acid | 0.01606 | 0.0431 | 0.2571 | 323.037 | Carboxylic acids and derivatives |
| Asparaginyl-Valine | 0.003276 | 0.01794 | 0.1903 | 214.1187 | Carboxylic acids and derivatives |
| 2-(Methylthio)ethyl glucosinolate | 0.01452 | 0.04009 | -0.2017 | 413.9993 | Organooxygen compounds |
| 2'',6''-O-Diacetylononin | 1.11E-06 | 7.69E-05 | -0.1321 | 415.1826 | Pteridines and derivatives |
| Ricinoleic acid | 0.000548 | 0.003952 | -0.1849 | 343.2481 | Fatty Acyls |
| Glutamylisoleucine | 0.001972 | 0.01283 | 0.1476 | 243.134 | Carboxylic acids and derivatives |
| N6-Methyl-2'-deoxyadenosine | 0.000163 | 0.002487 | 0.1537 | 248.1143 | Purine nucleosides |
| Betamethasone 17-benzoate | 0.004411 | 0.02202 | 0.1487 | 529.2602 | Steroids and steroid derivatives |
| 3-O-beta-D-glucosyl-brassicasterol | 0.001793 | 0.009176 | 0.1703 | 559.3987 | Steroids and steroid derivatives |
| Exo-2-Methyl-3-methylenebicyclo[2.2.1]heptan-2-ol | 1.31E-05 | 0.000497 | 0.1725 | 121.1014 | Prenol lipids |
| Bioadykinin Fragment 1-5 | 0.005272 | 0.02497 | -0.1589 | 586.3195 | Carboxylic acids and derivatives |
| Histidylproline | 0.000144 | 0.002281 | -0.1502 | 235.1188 | Carboxylic acids and derivatives |
| 3-Hydroxytetradecanedioic acid | 5.81E-05 | 0.001265 | 0.1573 | 316.2121 | Fatty Acyls |
| Vomifoliol | 2.09E-05 | 0.000654 | 0.1591 | 189.1275 | Prenol lipids |
| Oxandrolone | 2.02E-05 | 0.000643 | 0.1288 | 370.2334 | Steroids and steroid derivatives |
| Val-Gly-Val-Ala-Pro-Gly | 0.009009 | 0.02834 | 0.1933 | 543.2771 | Carboxylic acids and derivatives |
| Stachyose | 0.000276 | 0.002453 | -0.1584 | 665.2129 | Organooxygen compounds |
| Palmitoyl Ara-C | 0.000358 | 0.002911 | -0.148 | 526.3135 | Pyrimidine nucleosides |
| PE(21:0/0:0) | 0.004065 | 0.02084 | -0.1483 | 524.3707 | |
| 4-Hydroxyretinoic acid | 1.32E-05 | 0.000497 | 0.1439 | 317.2111 | Prenol lipids |
| N-Lauroyl Phenylalanine | 6.23E-05 | 0.000902 | 0.1666 | 368.2182 | Carboxylic acids and derivatives |
| 14-HDoHE | 7.24E-05 | 0.001482 | -0.1373 | 401.2029 | Fatty Acyls |
| P-Hydroxyphenethyl trans-ferulate | 0.000966 | 0.005904 | 0.1699 | 313.1077 | Cinnamic acids and derivatives |
| LysoPC(20:4(8Z,11Z,14Z,17Z)/0:0) | 0.009358 | 0.03733 | -0.1549 | 566.3214 | Glycerophospholipids |
| Cl-amidine | 0.007368 | 0.03152 | -0.1727 | 621.251 | Benzene and substituted derivatives |
| 4-Oxododecanedioic acid | 5.06E-06 | 0.000284 | 0.1192 | 267.1202 | |
| Zanthodioline | 0.002087 | 0.01322 | -0.1386 | 323.1602 | Quinolines and derivatives |
| N-Myristoyl Lysine | 9.13E-05 | 0.001717 | 0.1334 | 357.311 | Carboxylic acids and derivatives |

| | | | | | |
|---|---|---|---|---|---|
| LysoPC(20:1(11Z)/0:0) | 0.000318 | 0.00383 | -0.1192 | 550.3865 | Glycerophospholipids |
| Canavanine | 1.94E-06 | 0.000103 | 0.1703 | 211.0604 | Carboxylic acids and derivatives |
| Pteroic acid | 0.009855 | 0.0387 | 0.254 | 313.1042 | Pteridines and derivatives |
| 3-hydroxyicosanoic Acid | 0.007242 | 0.02407 | 0.2325 | 327.2897 | Fatty Acyls |
| (6R,8Z)-6-Hydroxy-3-oxotetradecenoic acid | 0.01259 | 0.0457 | 0.211 | 221.1536 | Fatty Acyls |
| Schaftoside | 0.000308 | 0.002625 | -0.1147 | 563.1391 | Flavonoids |
| Ligustilide | 1.05E-05 | 0.00044 | 0.1417 | 191.1067 | Isobenzofurans |
| N-[1'-(6-Cyano-1,2,3,4-tetrahydronaphthalen-2-yl)-4-hydroxyspiro[3,4-dihydrochromene-2,4'-piperidine]-6-yl]methanesulfonamide | 0.00027 | 0.002418 | -0.1454 | 488.1583 | Tetralins |
| LysoPC(22:6(4Z,7Z,10Z,13Z,16Z,19Z)/0:0) | 0.002341 | 0.01421 | -0.1277 | 568.3395 | Glycerophospholipids |
| Cis-4-Decenedioic acid | 7.05E-05 | 0.000976 | 0.15 | 181.086 | Fatty Acyls |
| Diphenylhydantoic acid | 0.000114 | 0.001353 | -0.125 | 305.0694 | Benzene and substituted derivatives |
| Cis-Vaccenic acid | 0.003376 | 0.01828 | 0.2084 | 247.242 | Fatty Acyls |
| 4-Isopropylbenzoic acid | 1.89E-05 | 0.000615 | 0.1463 | 165.0912 | Prenol lipids |
| Moroxydine | 0.000576 | 0.004085 | -0.1591 | 387.2241 | Organonitrogen compounds |
| Glu Val Glu | 8.96E-05 | 0.001697 | -0.1321 | 376.1717 | |
| Isovitexin 2''-O-arabinoside | 0.000278 | 0.003489 | -0.1072 | 565.1549 | Flavonoids |
| Norfloxacin | 0.001857 | 0.0094 | -0.2021 | 354.1049 | Carboxylic acids and derivatives |
| N-(2,3,4-Trihydroxybutyl)-L-valine | 5.86E-05 | 0.000867 | -0.1741 | 202.1076 | Carboxylic acids and derivatives |
| Folinic acid | 0.001355 | 0.007471 | -0.1488 | 472.1573 | Pteridines and derivatives |
| Androsterone sulfate | 0.000584 | 0.004127 | 0.1382 | 415.1786 | Steroids and steroid derivatives |
| Cis-Piceid | 7.74E-06 | 0.000244 | -0.1435 | 427.0805 | Stilbenes |
| Salbutamol | 0.000562 | 0.005535 | -0.125 | 496.3394 | Benzene and substituted derivatives |
| Caproic acid | 9.91E-06 | 0.000428 | -0.1578 | 158.1177 | Fatty Acyls |
| N-Acetyl-b-D-galactosamine | 0.000134 | 0.001509 | 0.1463 | 202.0712 | Organooxygen compounds |
| PL | 0.01283 | 0.03672 | -0.1861 | 587.3034 | Macrolides and analogues |
| PE(18:0/0:0) | 5.68E-05 | 0.000857 | -0.0963 | 480.3082 | |
| 4-Hydroxyphenyl-2-propionic acid | 0.01219 | 0.03544 | 0.1914 | 331.1182 | Phenylpropanoic acids |
| Adynerin | 1.44E-06 | 8.89E-05 | 0.1118 | 515.3 | |
| Terbutaline | 0.000672 | 0.006185 | 0.1598 | 514.2913 | Phenols |
| 2-Hydroxyquinoline-3-carboxylic acid | 2.98E-08 | 8.32E-06 | 0.1046 | 188.0344 | Quinolines and derivatives |
| Nonate | 0.000272 | 0.003443 | 0.1786 | 230.1387 | Fatty Acyls |
| Pectenotoxin 7 | 0.003536 | 0.0189 | -0.1517 | 456.2268 | Macrolides and analogues |
| Tetrahydropersin | 0.000751 | 0.006638 | -0.1373 | 448.3417 | Fatty Acyls |
| 9-Hydroxymegastigma-4,6,7-trien-3-one | 0.000852 | 0.007262 | -0.1655 | 207.138 | Prenol lipids |
| PS(6 keto-PGF1alpha/22:0) | 3.03E-05 | 0.000835 | -0.1269 | 489.7901 | |
| Byakangelicol | 0.007106 | 0.02374 | 0.2407 | 361.0923 | Coumarins and derivatives |
| (R)-2-Benzylsuccinate | 5.43E-05 | 0.001208 | 0.16 | 226.1074 | Phenylpropanoic acids |
| Biochanin A 7-(6-malonylglucoside) | 0.001505 | 0.01055 | -0.1178 | 565.155 | Isoflavonoids |
| (8R,9S,10S,13S,14S,17R)-16-Fluoro-17-hydroxy-10,13-dimethyl-1,2,4,5,6,7,8,9,11,12,14,15,16,17-tetradecahydrocyclopenta[a]phenanthren-3-one | 0.00081 | 0.007008 | 0.1271 | 341.2473 | Steroids and steroid derivatives |
| NeuNGc | 2.01E-05 | 0.000443 | -0.1111 | 324.0929 | Organooxygen compounds |
| 4,4-Dimethyl-2-[3-carboxylatopropyl]-2-tridecyloxazolidine 3-oxide | 0.003699 | 0.01494 | 0.1996 | 366.3003 | Fatty Acyls |
| Zedoarol | 3.68E-05 | 0.000636 | 0.1178 | 551.2645 | Prenol lipids |

| | | | | | |
|---|---|---|---|---|---|
| 4-Hydroxy-2H-pyran-3-carboxaldehyde | 0.003681 | 0.01489 | 0.2031 | 311.0769 | Pyrans |
| Lycoperdic acid | 7.88E-05 | 0.001053 | -0.123 | 262.0563 | Carboxylic acids and derivatives |
| (S,E)-Zearalenone | 0.000214 | 0.002045 | 0.1408 | 363.1445 | Macrolides and analogues |
| Coniferin | 0.007629 | 0.02504 | -0.1763 | 377.0983 | Organooxygen compounds |
| Alpha-Tocotrienoxyl radical | 0.002105 | 0.01327 | 0.1282 | 407.3266 | Prenol lipids |
| Cortisol | 0.0101 | 0.03925 | -0.1501 | 788.4296 | Steroids and steroid derivatives |
| Fructosyl-lysine | 8.86E-05 | 0.001144 | -0.1626 | 307.1505 | Organooxygen compounds |
| N-Stearoyl Glutamine | 0.00092 | 0.007641 | 0.1587 | 395.3265 | Carboxylic acids and derivatives |
| METHOPRENE (S) | 1.33E-05 | 0.000497 | 0.1033 | 333.2399 | |
| Adenine | 0.001181 | 0.008959 | 0.1459 | 136.0619 | Imidazopyrimidines |
| Acetyl-N-formyl-5-methoxykynurenamine | 0.00016 | 0.001688 | -0.1326 | 245.0926 | Organooxygen compounds |
| DG(6 keto-PGF1alpha/i-15:0/0:0) | 4.28E-05 | 0.001032 | 0.1243 | 357.2353 | |
| Isoetharine | 0.000143 | 0.002272 | -0.1179 | 496.3398 | Phenols |
| Dihydroactinidiolide | 2.94E-06 | 0.000203 | 0.1257 | 163.1118 | Benzofurans |
| Asp Phe | 0.000554 | 0.005487 | -0.1367 | 281.1131 | |
| 1-a,24R,25-Trihydroxyvitamin D2 | 1.40E-05 | 0.00051 | 0.1215 | 427.3213 | Steroids and steroid derivatives |
| Valerenolic acid | 0.000168 | 0.002511 | 0.1321 | 501.3205 | Prenol lipids |
| Glycocholenate sulfate | 5.36E-05 | 0.00083 | -0.1426 | 542.2415 | Steroids and steroid derivatives |
| 1-(4-Hydroxy-3-methoxyphenyl)-3-decanone | 0.00037 | 0.004254 | -0.1416 | 317.1495 | Phenols |
| N-[(1R)-2,3-Dihydro-1H-inden-1-yl]-adenosine | 0.000809 | 0.007007 | 0.1303 | 366.1594 | Purine nucleosides |
| N1-(5-Phospho-a-D-ribosyl)-5,6-dimethylbenzimidazole | 0.002872 | 0.01245 | 0.1521 | 393.0641 | Benzimidazole ribonucleosides and ribonucleotides |
| 1-Deoxynojirimycin | 1.57E-05 | 0.000548 | 0.1427 | 164.0918 | Piperidines |
| Apiin | 0.000648 | 0.004439 | -0.1111 | 563.1392 | Naphthalenes |
| 7Z,10Z-Hexadecadienoic acid | 0.000607 | 0.005779 | 0.1372 | 275.2005 | Fatty Acyls |
| Asp Ile Asn | 0.000168 | 0.002511 | -0.1201 | 361.1715 | |
| Polyethylene, oxidized | 9.82E-06 | 0.000289 | 0.1108 | 243.1232 | Keto acids and derivatives |
| Neomacrostemonoside D | 0.01428 | 0.03964 | -0.1591 | 552.2669 | Steroids and steroid derivatives |
| Citbismine F | 0.002595 | 0.01192 | -0.134 | 635.2024 | Quinolines and derivatives |
| Fenoprofen | 2.13E-05 | 0.00046 | 0.1361 | 263.0667 | Benzene and substituted derivatives |
| Ascorbic acid | 0.000392 | 0.003105 | 0.1605 | 197.0083 | Dihydrofurans |
| 1,5-Naphthalenediamine | 0.00308 | 0.01724 | 0.1888 | 159.0918 | Naphthalenes |
| Aplysiatoxin, 17-debromo- | 0.000141 | 0.002241 | 0.0872 | 593.3329 | |
| Tranexamic Acid | 8.95E-07 | 9.60E-05 | 0.1283 | 190.1439 | Carboxylic acids and derivatives |
| Muramic acid | 0.000526 | 0.005349 | 0.1551 | 251.1026 | Organooxygen compounds |
| Edivoxetine | 0.000185 | 0.001848 | -0.1381 | 374.1563 | Phenol ethers |
| Uridine, 2'-deoxy-2'-fluoro-2'-methyl-, (2'R)- | 8.19E-07 | 6.77E-05 | -0.125 | 305.0774 | Pyrimidine nucleosides |
| Asperagenin | 0.01081 | 0.04107 | 0.1437 | 431.3115 | Prenol lipids |
| Morphine 3-Sulfate | 0.000412 | 0.003223 | 0.126 | 364.0854 | Morphinans |
| Alpha-Terpineol acetate | 2.08E-05 | 0.000452 | 0.1563 | 241.1439 | Prenol lipids |
| Lotaustralin | 0.000746 | 0.006611 | -0.1285 | 325.1392 | Organooxygen compounds |
| 5,9-Epidioxy-3-hydroxyergost-7-en-6-one | 1.02E-05 | 0.000434 | 0.1213 | 245.1496 | Steroids and steroid derivatives |
| Hydroxyprolyl-Leucine | 5.64E-06 | 0.000197 | -0.1242 | 243.1344 | Carboxylic acids and derivatives |
| Thymidine | 0.000475 | 0.005022 | 0.1502 | 265.0818 | Pyrimidine nucleosides |
| PE(18:1(9Z)/0:0) | 6.42E-05 | 0.000918 | -0.0977 | 478.2925 | |

| | | | | | |
|---|---|---|---|---|---|
| Enterolactone | 0.003741 | 0.01506 | 0.1657 | 333.0894 | Furanoid lignans |
| 3-Methyldioxyindole | 6.27E-08 | 1.53E-05 | 0.1019 | 144.0443 | Indoles and derivatives |
| N-Methoxysuccinyl-Ala-Ala-Pro-Val | 0.001636 | 0.008573 | 0.1165 | 491.2086 | Carboxylic acids and derivatives |
| Allantoic acid | 0.00073 | 0.004814 | -0.1448 | 411.1261 | Carboxylic acids and derivatives |
| Violet-leaf aldehyde | 1.50E-05 | 0.000529 | 0.1267 | 121.1015 | Organooxygen compounds |
| Nalmefene | 0.005235 | 0.01894 | -0.1689 | 374.1561 | Phenanthrenes and derivatives |
| Demethoxyrapamycin | 0.01324 | 0.04732 | -0.1364 | 453.768 | Macrolide lactams |
| DG(i-16:0/0:0/18:1(12Z)-O(9S,10R)) | 0.002418 | 0.01454 | 0.1217 | 631.4905 | |
| Asp Asp Ile | 3.86E-07 | 6.60E-05 | -0.1084 | 362.1555 | |
| Histidyltryptophan | 0.007495 | 0.02469 | -0.1715 | 340.1408 | Carboxylic acids and derivatives |
| (3beta,5alpha,6alpha,7alpha,22E,24R)-5,6-Epoxyergosta-8,14,22-triene-3,7-diol | 1.68E-05 | 0.000391 | 0.0887 | 471.3102 | Steroids and steroid derivatives |
| Naphthalene-1,2-diol | 1.28E-05 | 0.00034 | -0.1342 | 319.096 | Naphthalenes |
| 2,3-Dihydroxycarbamazepine | 0.002031 | 0.01008 | -0.1586 | 305.0331 | Benzazepines |
| Mesobilirubinogen | 3.70E-05 | 0.000636 | 0.1031 | 591.3177 | Tetrapyrroles and derivatives |
| (S)-(+)-1-(p-Hydroxy-trans-cinnamoyl)-glycerol | 0.007733 | 0.02531 | 0.1827 | 237.0758 | Cinnamic acids and derivatives |
| N2-(gamma-Glutamyl)-4-carboxyphenylhydrazine | 0.000102 | 0.001807 | -0.126 | 323.1347 | Carboxylic acids and derivatives |
| Humilixanthin | 2.70E-05 | 0.000774 | -0.1103 | 291.0974 | Carboxylic acids and derivatives |
| N1-(alpha-D-ribosyl)-5,6-dimethyl-benzimidazole | 0.000423 | 0.003293 | -0.134 | 323.1243 | Benzimidazole ribonucleosides and ribonucleotides |
| Tumonoic Acid E | 5.39E-05 | 0.001207 | -0.1136 | 326.2322 | |
| N-Acetyl-L-glutamate 5-semialdehyde | 2.97E-05 | 0.000567 | -0.125 | 218.0662 | Carboxylic acids and derivatives |
| [1S-[1alpha,2alpha(Z),3alpha,4alpha]]-7-[3-[[2-[(Phenylamino)carbonyl]hydrazino]methyl]-7-oxabicyclo[2.2.1]hept-2-yl]-5-heptenoicacid | 0.001315 | 0.007327 | 0.1734 | 408.1874 | Benzene and substituted derivatives |
| Ala-Val-OH | 0.008309 | 0.03425 | -0.1599 | 297.108 | |
| L-Xylonic acid | 6.69E-07 | 5.90E-05 | -0.1028 | 165.0394 | Organooxygen compounds |
| 2,4,6(1H,3H,5H)-Pyrimidinetrione, 5-ethyl-5-(3-hydroxy-1-methylbutyl)- | 2.63E-06 | 0.000123 | -0.122 | 287.1244 | Diazines |
| Desglucocoroloside | 5.27E-06 | 0.000189 | 0.1011 | 549.3058 | Steroids and steroid derivatives |
| N-acetylmuramyl-l-alanine | 0.001207 | 0.009085 | 0.1578 | 347.145 | Carboxylic acids and derivatives |
| Asp-Glu | 0.000428 | 0.004697 | -0.1159 | 263.0873 | Carboxylic acids and derivatives |
| 1-(4-O-beta-D-glucopyranosyl-3-methoxyphenyl)-3,5-dihydroxydecane | 0.000154 | 0.001655 | -0.1176 | 475.2664 | Organooxygen compounds |
| 7-Methylxanthine | 0.00088 | 0.005519 | -0.1319 | 497.1401 | Imidazopyrimidines |
| N-[[3-(b-D-Glucopyranosyloxy)-2,3-dihydro-2-oxo-1H-indol-3-yl]acetyl]aspartic acid | 0.000706 | 0.004717 | -0.107 | 483.1244 | Carboxylic acids and derivatives |
| 5-Hydroxyindoleacetylglycine | 0.000552 | 0.005481 | -0.1119 | 281.1131 | Carboxylic acids and derivatives |
| 7-Methylrosmanol | 0.001996 | 0.009945 | 0.1596 | 381.1708 | Prenol lipids |
| LysoPC(15:0/0:0) | 0.000938 | 0.007691 | 0.1213 | 520.2801 | Glycerophospholipids |
| Dihydrobiopterin | 1.09E-05 | 0.000442 | -0.1106 | 303.1185 | Pteridines and derivatives |
| Butanedioic acid, octenyl- | 9.68E-06 | 0.000421 | 0.1018 | 211.1329 | Fatty Acyls |
| Ineketone | 0.000323 | 0.002711 | -0.1033 | 363.217 | Prenol lipids |
| Semilepidinoside A | 2.37E-06 | 0.000115 | -0.1168 | 335.1244 | Organooxygen compounds |
| Methylimidazoleacetic acid | 0.000466 | 0.004978 | -0.1058 | 141.0659 | Azoles |
| Idazoxan | 0.000913 | 0.005657 | 0.147 | 249.0875 | Benzodioxanes |
| (E)-N-(3-(3-(4-Fluorophenoxy)phenyl)-1-(R,S)-methylprop-2-enyl)-N-hydroxyurea | 8.70E-05 | 0.00113 | 0.1125 | 631.2399 | Benzene and substituted derivatives |
| (4S,6S)-3,4,5,6-Tetrahydro-4-hydroxy-6-methyl-2H-pyran-2-one | 0.000103 | 0.001263 | 0.157 | 175.0602 | Lactones |
| Pubesenolide | 0.002741 | 0.01219 | -0.1451 | 439.2838 | Prenol lipids |
| 6-Hydroxynicotinic Acid | 2.72E-07 | 5.65E-05 | 0.1226 | 140.0344 | Pyridines and derivatives |

| | | | | | |
|---|---|---|---|---|---|
| Cinaciguat | 0.00233 | 0.01416 | 0.1245 | 607.3119 | Stilbenes |
| Dialdehyde | 0.004983 | 0.02401 | -0.1378 | 413.1413 | Harmala alkaloids |
| Isofloxythepin | 0.00055 | 0.003959 | -0.1286 | 399.1875 | Benzothiepins |
| 3-Hydroxy-11-norcytisine | 0.000636 | 0.004385 | -0.1147 | 575.2589 | Pyridodiazepines |
| Galactoarabinan | 0.001958 | 0.01277 | -0.1142 | 483.2084 | Organooxygen compounds |
| Corchoroside B | 1.50E-05 | 0.00037 | 0.1092 | 499.2695 | Steroids and steroid derivatives |
| Suberylglycine | 0.004838 | 0.02347 | 0.173 | 264.144 | Carboxylic acids and derivatives |
| LysoPE(18:0/0:0) | 0.000262 | 0.003356 | -0.0841 | 482.3242 | Glycerophospholipids |
| 22-Hydroxydocosanoic acid | 9.17E-05 | 0.001717 | 0.1166 | 395.29 | Fatty Acyls |
| Asparaginylaspartic acid | 0.001047 | 0.00625 | -0.1432 | 246.0725 | Carboxylic acids and derivatives |
| (+/-)-Pelletierine | 4.27E-07 | 4.50E-05 | 0.1174 | 186.1126 | Piperidines |
| Alpha-Dimorphecolic acid | 0.000187 | 0.002674 | 0.115 | 297.2423 | Fatty Acyls |
| Dodecanedioic Acid | 1.13E-06 | 7.75E-05 | 0.0923 | 229.1438 | Fatty Acyls |
| Hexadeca-7,10,13-trienoic acid | 0.01002 | 0.03914 | 0.1577 | 215.1795 | Fatty Acyls |
| Methylisopelletierine | 9.79E-07 | 7.14E-05 | 0.113 | 200.1284 | Piperidines |
| Vanilloyl glucose | 0.00019 | 0.001887 | 0.1328 | 311.0767 | Tannins |
| Taurodeoxycholic acid | 0.005188 | 0.01883 | 0.0972 | 498.2881 | Steroids and steroid derivatives |
| Methyl (3b,11x)-3-Hydroxy-8-oxo-6-eremophilen-12-oate | 9.16E-07 | 7.14E-05 | -0.1112 | 301.1398 | Prenol lipids |
| Hippuric acid | 0.00219 | 0.01059 | -0.1565 | 224.0556 | Benzene and substituted derivatives |
| Glycitein | 0.000143 | 0.001582 | -0.1192 | 283.0607 | Isoflavonoids |
| N-Arachidonoyl Glutamic acid | 0.000152 | 0.002354 | 0.113 | 433.2846 | Carboxylic acids and derivatives |
| 5,8-Dihydroxy-1,4-naphthoquinone | 1.06E-05 | 0.00044 | 0.1141 | 208.0605 | Naphthalenes |
| 4-Hydroxy-3-(3-methyl-2-butenyl)acetophenone | 0.000193 | 0.002712 | 0.1406 | 237.1485 | Organooxygen compounds |
| Napelline | 3.56E-05 | 0.000915 | 0.116 | 377.2795 | |
| Tryptophyl-Glutamine | 0.002466 | 0.01474 | -0.1297 | 333.1558 | Carboxylic acids and derivatives |
| Neogitogenin | 8.80E-05 | 0.001676 | 0.105 | 397.3059 | Prenol lipids |
| 5-Fluorouridine | 0.001064 | 0.008331 | -0.0973 | 263.0696 | Pyrimidine nucleosides |
| Ser Val Gln Leu Leu | 0.0123 | 0.04487 | -0.1439 | 559.3447 | |
| Plumieride | 0.001437 | 0.01022 | 0.1231 | 470.1418 | Prenol lipids |
| Zaragozic acid A | 0.000305 | 0.002611 | 0.1156 | 671.2742 | Carboxylic acids and derivatives |
| Medicagenic acid | 7.33E-05 | 0.001485 | 0.0994 | 503.336 | Prenol lipids |
| Valylhydroxyproline | 1.30E-05 | 0.000494 | -0.1162 | 253.1158 | Carboxylic acids and derivatives |
| Arenobufagin | 0.001333 | 0.009744 | -0.1142 | 439.2083 | Steroids and steroid derivatives |
| PC(20:3/0:0) | 0.005755 | 0.02656 | -0.1042 | 546.3554 | |
| 2-Polyprenyl-6-methoxy-1,4-benzoquinone | 0.001557 | 0.008258 | -0.1462 | 295.1293 | Prenol lipids |
| DG(2:0/18:1(9Z)-O(12,13)/0:0) | 0.000823 | 0.005256 | -0.1098 | 447.2506 | |
| Idebenone | 0.004009 | 0.02065 | -0.134 | 181.1027 | |
| Asparaginyl-Proline | 0.002421 | 0.01454 | 0.1215 | 212.103 | Carboxylic acids and derivatives |
| 4-O-(Indole-3-acetyl)-D-glucopyranose | 0.004121 | 0.01607 | 0.1659 | 336.1083 | Indoles and derivatives |
| Ile Phe Gly | 0.001118 | 0.008618 | 0.123 | 336.1915 | |
| 4-Demethylsimmondsin 2'-(E)-ferulate | 0.01221 | 0.03548 | 0.1859 | 572.1545 | Cinnamic acids and derivatives |
| N-lactoyl-Tryptophan | 0.004174 | 0.0212 | 0.118 | 277.1182 | Carboxylic acids and derivatives |
| 16-feruloyloxypalmitate | 0.000655 | 0.006094 | -0.1147 | 489.3065 | Cinnamic acids and derivatives |
| Cassiaside B | 0.0172 | 0.0452 | 0.1851 | 565.1548 | Naphthopyrans |

| | | | | | |
|---|---|---|---|---|---|
| DG(18:4(6Z,9Z,12Z,15Z)/18:3(9Z,12Z,15Z)/0:0) | 0.000292 | 0.003597 | 0.1063 | 611.4639 | Fatty Acyls |
| N-Acetyl-L-Tyrosine | 2.23E-06 | 0.000112 | 0.1096 | 222.0765 | Carboxylic acids and derivatives |
| 1-Methyladenosine | 0.000467 | 0.004978 | 0.1238 | 282.1197 | Purine nucleosides |
| Neocnidilide | 0.007379 | 0.03155 | 0.1474 | 195.1381 | Isobenzofurans |
| Vulgaxanthin II | 0.004649 | 0.01747 | 0.1443 | 385.0921 | Carboxylic acids and derivatives |
| Jacarandic acid | 0.000719 | 0.004772 | 0.1047 | 469.3306 | Prenol lipids |
| 1b,3a,7b-Trihydroxy-5b-cholanoic acid | 8.00E-06 | 0.000248 | 0.0892 | 469.2793 | Glycerophospholipids |
| L-Cystine | 0.001619 | 0.008504 | -0.1299 | 239.0159 | Carboxylic acids and derivatives |
| Bufadienolide | 0.003463 | 0.01425 | -0.1361 | 399.2524 | Steroids and steroid derivatives |
| Ubiquinone-1 | 0.005618 | 0.02613 | 0.175 | 251.1254 | Prenol lipids |
| 3-hydroxyundecanoic acid | 6.52E-05 | 0.000927 | 0.1353 | 247.1545 | Hydroxy acids and derivatives |
| 11H-14,15-EETA | 0.001653 | 0.008627 | 0.1404 | 381.2275 | Fatty Acyls |
| 27-Deoxy-5b-cyprinol | 0.002079 | 0.01027 | 0.1305 | 457.3312 | Steroids and steroid derivatives |
| Dioscoretine | 6.84E-05 | 0.001428 | 0.1114 | 283.2015 | Fatty Acyls |
| Tetragastrin | 3.42E-06 | 0.000144 | 0.0891 | 297.1154 | Carboxylic acids and derivatives |
| Aminophenazone | 0.00074 | 0.006563 | -0.0908 | 504.3062 | Azoles |
| (2'E,4'Z,7'Z,8E)-Colnelenic acid | 3.05E-05 | 0.000837 | 0.0994 | 325.2367 | Fatty Acyls |
| Butralin | 0.000777 | 0.006796 | 0.0988 | 613.2993 | Benzene and substituted derivatives |
| 3,6-Ditigloyloxytropan-7-ol | 0.000614 | 0.004275 | -0.1346 | 358.1612 | Tropane alkaloids |
| L-Arginine, L-asparaginylglycyl- | 0.001219 | 0.006957 | -0.1312 | 380.1455 | Carboxylic acids and derivatives |
| 3-O-beta-D-Galactopyranosyl-L-arabinose | 8.39E-05 | 0.001102 | -0.0899 | 357.1031 | Organooxygen compounds |
| Olprinone | 3.24E-05 | 0.000593 | 0.1372 | 295.0851 | Pyridines and derivatives |
| Lys Met Thr | 0.005638 | 0.02618 | -0.1415 | 379.1978 | |
| Phoenicoxanthin | 0.001444 | 0.007832 | -0.1154 | 579.3884 | Prenol lipids |
| Wogonin | 0.001156 | 0.008819 | -0.0847 | 285.0756 | |
| Gibberellin A36 | 4.10E-05 | 0.00069 | -0.1098 | 361.165 | Prenol lipids |
| Mycophenolic acid | 0.006441 | 0.02199 | 0.1427 | 341.1019 | Isocoumarans |
| 10-Nitrolinoleic acid | 0.000772 | 0.006759 | 0.116 | 370.197 | Fatty Acyls |
| Prostaglandin D1 Alcohol | 0.009956 | 0.03041 | 0.1603 | 339.2531 | |
| Acetylcysteine | 0.004511 | 0.01709 | -0.1365 | 162.0219 | Carboxylic acids and derivatives |
| Quassinol | 0.001061 | 0.008318 | -0.1111 | 424.1711 | Prenol lipids |
| Aflatoxin B2 | 6.36E-07 | 5.79E-05 | 0.1146 | 359.0799 | Coumarins and derivatives |
| Moxaprindine | 0.01003 | 0.03054 | -0.1472 | 387.224 | Indanes |
| Hypoglycin B | 0.000249 | 0.002283 | 0.1393 | 269.1139 | Carboxylic acids and derivatives |
| Gingerglycolipid C | 0.002761 | 0.01592 | 0.118 | 703.3867 | Glycerolipids |
| Micronomicin | 0.000343 | 0.004022 | 0.1043 | 464.3123 | Organooxygen compounds |
| His His His | 0.004322 | 0.02172 | 0.1225 | 430.1917 | |
| 5''-(4-Hydroxy-(E)-cinnamoyl) alpha-L-arabinofuranosyl-(1->3)-beta-D-xylopyranosyl-(1->4)-D-xylopyranoside | 0.002559 | 0.01182 | -0.1081 | 581.1474 | Organooxygen compounds |
| Glutaminylproline | 0.003357 | 0.01822 | 0.1122 | 226.1185 | Carboxylic acids and derivatives |
| 8-O-Acetyl shanzhiside methyl ester | 6.29E-06 | 0.000213 | -0.0968 | 483.1244 | Prenol lipids |
| Leu Arg | 0.01412 | 0.04943 | -0.1445 | 288.2028 | |
| D-Sorbitol | 0.000272 | 0.002434 | -0.1106 | 217.0474 | Organooxygen compounds |
| Asp-Phe | 0.000877 | 0.005509 | -0.122 | 279.0981 | Carboxylic acids and derivatives |

| | | | | | |
|---|---|---|---|---|---|
| Aspartyl-Proline | 0.001221 | 0.009159 | 0.1002 | 213.087 | Carboxylic acids and derivatives |
| Cis-Dihydrocarvone | 5.47E-05 | 0.001213 | 0.1206 | 135.117 | Prenol lipids |
| Adrenic acid | 0.006692 | 0.02268 | 0.1231 | 377.2688 | Fatty Acyls |
| 7-Hydroxy-3-(3-hydroxy-4-methoxybenzyl)-5-methoxy-4-chromanone | 0.002455 | 0.01147 | 0.1108 | 329.1024 | Homoisoflavonoids |
| 1-Linoleoyl-sn-Glycero-3-Phosphocholine | 0.000339 | 0.002799 | -0.0868 | 564.3294 | Glycerophospholipids |
| 4-Ipomeanol | 4.66E-05 | 0.001095 | 0.0949 | 210.1125 | Organooxygen compounds |
| LysoPA(P-16:0/0:0) | 0.003625 | 0.01471 | 0.115 | 439.2456 | Glycerophospholipids |
| A-L-Arabinofuranosyl-(1->3)-b-D-xylopyranosyl-(1->4)-D-xylose | 0.000535 | 0.003891 | -0.0959 | 449.106 | Organooxygen compounds |
| Cis-3-Hexenyl lactate | 7.23E-06 | 0.000234 | 0.1223 | 217.1074 | Carboxylic acids and derivatives |
| 9-Oxo-nonanoic acid | 0.000723 | 0.006464 | 0.1312 | 214.1438 | Fatty Acyls |
| 4-Hydroxydodecanedioylcarnitine | 0.007769 | 0.03267 | -0.119 | 389.2393 | Fatty Acyls |
| Asp Ile | 0.001046 | 0.008236 | -0.0933 | 247.1289 | |
| 4-Trimethylammoniobutanoic Acid | 5.72E-07 | 7.62E-05 | 0.0779 | 146.1177 | Fatty Acyls |
| Pregnan-20-one, 17-(acetyloxy)-3-hydroxy-6-methyl-, (3b,5b,6a)- | 0.000306 | 0.003722 | 0.0817 | 391.284 | |
| Brexpiprazole | 0.000101 | 0.001806 | 0.0942 | 434.1887 | Diazinanes |
| Propyl gallate | 0.001255 | 0.009344 | 0.1231 | 230.1023 | Benzene and substituted derivatives |
| Perindopril | 0.001599 | 0.01102 | 0.1286 | 351.2309 | Carboxylic acids and derivatives |
| 2-Isopropyl-5-methylphenol acetate | 1.35E-05 | 0.000502 | 0.1059 | 193.1224 | Prenol lipids |
| Indole-3-acetic Acid | 8.41E-05 | 0.001632 | 0.1031 | 176.0707 | Indoles and derivatives |
| LysoPE(16:0/0:0) | 3.09E-05 | 0.000572 | -0.078 | 452.277 | Glycerophospholipids |
| Deflazacort | 0.001997 | 0.01295 | -0.1123 | 459.248 | Steroids and steroid derivatives |
| Hemiphloin | 0.000109 | 0.001316 | -0.1106 | 433.1129 | |
| 1,2,3,4-Tetrahydroisoquinoline-1-carboxylic acid | 0.00094 | 0.007697 | 0.1086 | 178.0864 | Tetrahydroisoquinolines |
| Allixin | 0.001105 | 0.008549 | 0.0982 | 290.1344 | Pyrans |
| Calcitroic acid | 0.000566 | 0.005552 | 0.0975 | 407.2792 | Steroids and steroid derivatives |
| Tezosentan | 0.01017 | 0.03082 | 0.1362 | 642.1286 | Diazines |
| Arginylproline | 0.001829 | 0.01213 | 0.1031 | 272.1716 | Carboxylic acids and derivatives |
| Kukoamine C | 0.000612 | 0.00581 | -0.0695 | 495.2963 | Phenols |
| Dimethylsphingosine | 0.000231 | 0.003052 | 0.0984 | 373.3059 | Organooxygen compounds |
| 7-Hexadecynoic acid | 0.000334 | 0.003946 | 0.1121 | 297.1808 | Fatty Acyls |
| D-Galactopyranosyl-(1->3)-D-galactopyranosyl-(1->3)-L-arabinose | 0.000134 | 0.00151 | -0.0919 | 519.1552 | Organooxygen compounds |
| 6-Lactoyltetrahydropterin | 0.001531 | 0.008162 | -0.1103 | 716.2915 | Pteridines and derivatives |
| 4-Ethyl-7,11-dimethyldodeca-trans-2-trans-6-1-o-trien-1-al | 0.0058 | 0.0267 | -0.1286 | 510.4262 | Prenol lipids |
| Leu Phe Ser | 0.005526 | 0.02581 | 0.117 | 366.2019 | |
| Ala His Phe Asp | 0.01263 | 0.03636 | 0.1491 | 487.1935 | |
| 1-{[(Z)-Benzylidene(oxido)-lambda~5~-azanyl]methyl}cyclohexanol | 0.000211 | 0.00286 | 0.1129 | 234.1489 | |
| 6-methyl-2-(2-morpholin-4-yl-2-oxoethyl)pyridazin-3-one | 0.000429 | 0.004701 | 0.108 | 238.1186 | |
| Gibberellin A51 | 0.000678 | 0.006219 | 0.111 | 365.1953 | Prenol lipids |
| Trilostane | 0.005616 | 0.02613 | -0.1203 | 393.2123 | Steroids and steroid derivatives |
| Threoninyl-Valine | 0.000666 | 0.006152 | -0.098 | 500.2713 | Carboxylic acids and derivatives |
| (5E)-7-[3,5-Dihydroxy-2-(3-hydroxyoctyl)cyclopentyl]hept-5-enoylcarnitine | 0.00104 | 0.006211 | 0.1161 | 534.316 | Fatty Acyls |
| CDP-ethanolamine | 0.01431 | 0.03969 | 0.1315 | 491.0618 | Pyrimidine nucleotides |
| Methyl dioxindole-3-acetate | 0.01285 | 0.03675 | 0.1585 | 202.0501 | Indoles and derivatives |
| 2-Deoxycastasterone | 0.003091 | 0.01312 | -0.1264 | 447.3467 | Steroids and steroid derivatives |

| | | | | | |
|---|---|---|---|---|---|
| Pro Arg | 0.002685 | 0.01564 | 0.1172 | 272.1715 | |
| Alanylthreonine | 0.000605 | 0.005775 | 0.1354 | 155.0816 | Carboxylic acids and derivatives |
| Leu Val | 0.01062 | 0.04064 | 0.1376 | 231.1703 | |
| Hydroxyphenylacetylglycine | 5.70E-06 | 0.000307 | 0.0896 | 242.1022 | Carboxylic acids and derivatives |
| Galactosylglycerol | 0.01091 | 0.04128 | 0.1734 | 254.1021 | Glycerolipids |
| 1,2-Cyclohexanediol | 0.000293 | 0.0036 | 0.1121 | 274.2011 | Organooxygen compounds |
| Lanthionine ketimine | 0.000651 | 0.004451 | -0.1228 | 169.9907 | Carboxylic acids and derivatives |
| 5,6-dehydro Arachidonic Acid | 9.97E-05 | 0.001239 | -0.0922 | 301.2166 | |
| Lumichrome | 0.000479 | 0.005043 | 0.0875 | 243.0876 | Pteridines and derivatives |
| Glutethimide | 0.000904 | 0.007565 | -0.1104 | 250.1438 | Piperidines |
| P-Phenylenediamine | 0.000991 | 0.006018 | 0.1565 | 261.1338 | Benzene and substituted derivatives |
| Rac-4-Hydroxy-4-O-(beta-D-glucuronide)-all-trans-retinyl acetate | 0.002622 | 0.01201 | 0.1025 | 501.2513 | Prenol lipids |
| Avocadyne 2-acetate | 0.000164 | 0.002489 | 0.0763 | 309.2422 | Fatty Acyls |
| Hydroxytetradecadienyl-l-carnitine | 0.001092 | 0.008499 | 0.0921 | 370.295 | Fatty Acyls |
| 6-(2-Hydroxyethoxy)-6-oxohexanoic acid | 0.000158 | 0.001682 | 0.1137 | 189.0759 | Fatty Acyls |
| Ser His Lys | 0.001295 | 0.009556 | 0.1088 | 371.2042 | |
| (E)-indol-3-ylacetaldoxime | 9.86E-05 | 0.001768 | 0.1134 | 175.0868 | Indoles and derivatives |
| S-4-Hydroxymephenytoin | 0.00055 | 0.005468 | 0.0849 | 235.1077 | Azolidines |
| Tryptophyl-Arginine | 0.001363 | 0.009882 | 0.0976 | 325.1758 | Carboxylic acids and derivatives |
| 7a-Hydroxydehydroepiandrosterone | 0.003003 | 0.01692 | 0.1326 | 327.1913 | Steroids and steroid derivatives |
| 2-(acetylamino)-1,5-anhydro-2-deoxy-4-O-b-D-galactopyranosyl-D-arabino-Hex-1-enitol | 0.004396 | 0.01678 | 0.1063 | 364.1244 | Organooxygen compounds |
| 18-hydroxyoleate | 0.00819 | 0.03391 | 0.1077 | 617.4747 | Fatty Acyls |
| 4-(2-Methylphenyl)butan-1-ol | 0.003656 | 0.01933 | 0.1324 | 147.1169 | Benzene and substituted derivatives |
| Coformycin | 0.000861 | 0.00543 | -0.1047 | 283.1042 | Organooxygen compounds |
| Tryptamine | 5.66E-05 | 0.000856 | 0.1214 | 195.0689 | Indoles and derivatives |
| PS(22:6(5Z,8E,10Z,13Z,15E,19Z)-2OH(7S, 17S)/20:4(8Z,11Z,14Z,17Z)) | 0.009064 | 0.03653 | -0.0968 | 444.7504 | |
| Falcarindiol | 0.000462 | 0.003522 | -0.1173 | 305.1752 | Fatty Acyls |
| Isoleucyl-Glutamate | 0.004731 | 0.01769 | 0.1057 | 241.1187 | Carboxylic acids and derivatives |
| Phenol A | 2.68E-05 | 0.000521 | -0.0957 | 241.1077 | Benzene and substituted derivatives |
| (2S)-1-[(2S)-3-(4H-Imidazol-4-yl)-2-[[(2S)-5-oxopyrrolidine-2-carbonyl]amino]propanoyl]pyrrolidine-2-carboxylic acid | 0.01712 | 0.04508 | -0.1597 | 400.106 | Carboxylic acids and derivatives |
| Pinolidoxin | 0.000167 | 0.001732 | 0.1063 | 383.1701 | Oxocins |
| Urobilinogen | 0.004906 | 0.02371 | 0.1124 | 591.317 | Tetrapyrroles and derivatives |
| (9Z,11E,13S,15Z)-13-Hydroxyoctadeca-9,11,15-trienoic acid | 9.25E-05 | 0.00172 | 0.0804 | 277.2161 | Fatty Acyls |
| DG(8:0/19:0/0:0) | 0.001466 | 0.01036 | 0.0959 | 537.3893 | Glycerolipids |
| Linoleyl-l-carnitine | 0.000423 | 0.00467 | 0.0793 | 454.3276 | Fatty Acyls |
| Cynaratriol | 0.001097 | 0.00646 | -0.1114 | 281.1389 | Prenol lipids |
| Hexylresorcinol | 0.000303 | 0.003701 | 0.0816 | 411.2503 | Phenols |
| (+)-Abscisic Acid | 6.96E-05 | 0.001444 | 0.103 | 265.1435 | Prenol lipids |
| (1r,3r)-1-Aminocyclopentane-1,3-dicarboxylic acid | 0.00014 | 0.002232 | 0.0953 | 138.0551 | Carboxylic acids and derivatives |
| Tetrahydrothiophene-3-ol 1,1-dioxide | 1.85E-05 | 0.000424 | 0.1104 | 181.0166 | Thiolanes |
| Abscisic alcohol | 0.001824 | 0.01212 | 0.0957 | 251.1641 | Prenol lipids |
| Darodipine | 4.92E-06 | 0.000281 | 0.0826 | 354.1446 | Benzoxadiazoles |
| (Z)-7-[(1R,2R,3R,5S)-2-(1,2-Dihydroxy-3-oxooctyl)-3,5-dihydroxycyclopentyl]hept-5-enoic acid | 0.000183 | 0.002657 | 0.0885 | 386.2283 | Fatty Acyls |

| | | | | | |
|---|---|---|---|---|---|
| N-Stearoyl Lysine | 0.000495 | 0.00513 | 0.0856 | 413.3735 | Carboxylic acids and derivatives |
| Colforsin daropate | 0.000662 | 0.004508 | 0.0866 | 530.2773 | Prenol lipids |
| D-Cathine | 0.003705 | 0.01955 | 0.1276 | 134.0966 | |
| Zalcitabine | 0.009333 | 0.03726 | 0.1093 | 253.1294 | Oxepanes |
| 4-Hydroxy-3-methyl-2-(2-propynyl)-2-cyclopentene-1-one | 0.00032 | 0.002697 | 0.1379 | 195.0654 | Organooxygen compounds |
| (5E,7E)-Undeca-2,5,7-trienedioylcarnitine | 0.000359 | 0.002911 | -0.1038 | 374.1565 | Fatty Acyls |
| Betaine aldehyde | 0.003955 | 0.02051 | 0.1074 | 147.0634 | Organonitrogen compounds |
| Ponasterone A | 0.01947 | 0.04959 | -0.1222 | 501.2667 | Steroids and steroid derivatives |
| Doxercalciferol | 0.000129 | 0.002087 | 0.0778 | 445.3674 | Steroids and steroid derivatives |
| Maltotetraose | 0.003528 | 0.01441 | -0.0951 | 701.1896 | Organooxygen compounds |
| Hydroxybutyl | 8.44E-06 | 0.000259 | 0.1145 | 203.1281 | Organooxygen compounds |
| Isoalantolactone | 0.002032 | 0.01304 | -0.1004 | 233.1537 | Prenol lipids |
| Ameltolide | 1.89E-05 | 0.000615 | 0.0984 | 273.1596 | Benzene and substituted derivatives |
| Ovalicin | 0.002394 | 0.01444 | 0.1188 | 296.1604 | Organooxygen compounds |
| Rhamnose | 1.20E-06 | 7.96E-05 | -0.0902 | 163.0601 | Organooxygen compounds |
| Avocadyne 4-acetate | 4.15E-05 | 0.001011 | 0.0707 | 349.2348 | Fatty Acyls |
| N-(N-(3-Amino-3-carboxypropyl)-3-amino-3-carboxypropyl)azetidine-2-carboxylic acid | 0.000308 | 0.002626 | -0.1078 | 302.1351 | Carboxylic acids and derivatives |
| Hydroxyphenyllactic acid | 0.001048 | 0.006251 | 0.0937 | 181.0497 | Phenylpropanoic acids |
| Trans-Piceid | 7.73E-05 | 0.001042 | -0.101 | 425.1016 | Stilbenes |
| Methyl bisnorbiotinyl ketone | 0.006532 | 0.02225 | 0.1351 | 259.0754 | Thienoimidazolidines |
| (2E,4E)-Hexa-2,4-dienedioylcarnitine | 0.001547 | 0.01074 | -0.0993 | 303.1552 | Fatty Acyls |
| 1-Pyrroline-2-carboxylic acid | 0.000253 | 0.003276 | 0.085 | 227.1026 | Pyrrolines |
| Dalfopristin | 0.006615 | 0.02917 | -0.1049 | 704.341 | Macrolide lactams |
| Corosin | 1.96E-06 | 0.000103 | 0.0881 | 539.2985 | Prenol lipids |
| LysoPG(18:2(9Z,12Z)/0:0) | 0.00087 | 0.005475 | -0.0799 | 507.2713 | Glycerophospholipids |
| Neolinustatin | 0.000176 | 0.002603 | 0.0918 | 388.1599 | Organooxygen compounds |
| 16-Nitroxystearate | 0.000458 | 0.004918 | 0.0933 | 403.3528 | Fatty Acyls |
| 2,6-Pyridinedicarboxylic Acid | 0.008127 | 0.03373 | 0.1054 | 168.0292 | Pyridines and derivatives |
| Hexyl benzoate | 0.00022 | 0.002092 | 0.1081 | 251.1283 | Benzene and substituted derivatives |
| Mycophenolic acid O-acyl-glucuronide | 0.000435 | 0.004757 | 0.0921 | 461.1438 | Organooxygen compounds |
| Avocadyne 1-acetate | 7.30E-05 | 0.001484 | 0.0777 | 344.2793 | Fatty Acyls |
| Lansiumarin C | 0.000165 | 0.002499 | 0.093 | 354.1448 | Coumarins and derivatives |
| N-Linoleoyl Tyrosine | 0.003545 | 0.01446 | 0.0972 | 442.2952 | Carboxylic acids and derivatives |
| Ethyl glucoside | 0.005095 | 0.01857 | -0.112 | 461.1875 | Organooxygen compounds |
| 1,2-Dihydroxy-3-keto-5-methylthiopentene | 0.003493 | 0.01876 | -0.1173 | 204.0691 | Organooxygen compounds |
| 8beta-Angeloyloxy-15-hydroxy-1alpha,10R-dimethoxy-3-oxo-11(13)-germacren-12,6alpha-olide | 0.009695 | 0.03828 | 0.107 | 463.1707 | Prenol lipids |
| Hygrine | 0.000144 | 0.001583 | 0.0976 | 186.1126 | |
| (4-Ethoxyphenyl)urea | 0.000723 | 0.004788 | -0.1027 | 225.0874 | Benzene and substituted derivatives |
| 2-Hydroxystearic acid | 0.01005 | 0.03918 | -0.0906 | 318.3001 | Fatty Acyls |
| Methylphenobarbital | 0.000332 | 0.00394 | 0.0923 | 247.1076 | Diazines |
| 3-Methylcrotonylglycine | 5.71E-05 | 0.000859 | 0.099 | 202.0712 | Carboxylic acids and derivatives |
| Isoleucyl-Valine | 0.01707 | 0.04498 | 0.1694 | 229.155 | Carboxylic acids and derivatives |
| 5-Amino-2-oxopentanoic acid | 0.001994 | 0.01295 | -0.0985 | 304.1503 | Keto acids and derivatives |
| Ethyl (S)-3-hydroxybutyrate glucoside | 0.008054 | 0.02614 | -0.1168 | 293.1236 | Fatty Acyls |

| | | | | | |
|---|---|---|---|---|---|
| Prostaglandin H2 | 0.000185 | 0.002664 | 0.0748 | 317.2086 | Fatty Acyls |
| 4-methoxy-3-(sulfooxy)benzoic acid | 9.22E-05 | 0.001179 | 0.1014 | 246.9913 | Benzene and substituted derivatives |
| L-Kynurenine | 0.004623 | 0.02275 | 0.1038 | 241.1183 | Organooxygen compounds |
| Bufogenin | 0.005661 | 0.02625 | -0.0922 | 402.2672 | Steroids and steroid derivatives |
| 7-Methylhypoxanthine | 0.003331 | 0.01812 | 0.11 | 151.0616 | Imidazopyrimidines |
| N,N,O-Tridesmethylvenlafaxine | 0.01431 | 0.04982 | -0.1175 | 218.154 | Organooxygen compounds |
| Wedelolactone | 0.006483 | 0.02212 | 0.1308 | 359.043 | Isoflavonoids |
| 1-Arachidonoylglycerol | 1.72E-05 | 0.000398 | 0.0645 | 423.2743 | Glycerolipids |
| Taurocholic acid 3-sulfate | 3.01E-05 | 0.000568 | 0.0704 | 296.6163 | Steroids and steroid derivatives |
| Doisynoestrol | 0.000502 | 0.003726 | 0.0999 | 319.1294 | Steroids and steroid derivatives |
| Sampatrilat | 0.00111 | 0.006521 | -0.0999 | 605.2234 | Peptidomimetics |
| LysoPC(16:1(9Z)/0:0) | 0.002156 | 0.01348 | -0.0708 | 494.3238 | Glycerophospholipids |
| (9Z,12R)-12-Hydroxyoctadec-9-enoic acid | 0.003243 | 0.01782 | 0.1128 | 281.2474 | Fatty Acyls |
| Acoric acid | 0.000158 | 0.002434 | 0.0853 | 251.1641 | Prenol lipids |
| Stizolamine | 0.000437 | 0.004769 | -0.0894 | 220.0816 | Diazines |
| Ala Met Leu | 0.004147 | 0.02112 | -0.1073 | 334.1789 | |
| Medicagenate | 1.68E-06 | 9.62E-05 | 0.0661 | 499.3054 | Prenol lipids |
| Puromycin | 0.002867 | 0.01641 | 0.0944 | 494.21 | Purine nucleosides |
| Decanedioic acid | 1.69E-06 | 9.62E-05 | 0.0805 | 201.1124 | Fatty Acyls |
| (R)-3,4-Dihydro-6,8-dihydroxy-3-methyl-1H-2-benzopyran-1-one | 0.01081 | 0.04107 | -0.0996 | 212.0918 | Benzene and substituted derivatives |
| Oxoglutaric acid | 0.000694 | 0.004662 | -0.0789 | 351.0563 | Keto acids and derivatives |
| 11b-Hydroxyandrost-4-ene-3,17-dione | 7.90E-06 | 0.000375 | 0.0872 | 325.1754 | Oxazinanes |
| PI(5-iso PGF2VI/22:5(4Z,7Z,10Z,13Z,16Z)) | 2.39E-05 | 0.000716 | 0.066 | 999.4775 | |
| Alanine lactate pyruvate | 0.000137 | 0.002202 | 0.0827 | 281.0993 | Carboxylic acids and derivatives |
| N-Lauroylglycine | 3.50E-05 | 0.000909 | 0.0734 | 258.2063 | Carboxylic acids and derivatives |
| Prostaglandin E3 | 3.92E-06 | 0.000245 | 0.0781 | 315.1953 | Fatty Acyls |
| ACEXAMIC ACID | 8.56E-05 | 0.001651 | 0.0809 | 174.1126 | Fatty Acyls |
| Butyl 3-hydroxy-2-methylidenebutanoate | 9.55E-05 | 0.001208 | 0.1072 | 217.1074 | Hydroxy acids and derivatives |
| Cypridina luciferin | 0.000126 | 0.002073 | -0.0793 | 406.2334 | Indoles and derivatives |
| Cholesterol glutamate | 0.001602 | 0.008442 | -0.09 | 550.3496 | Steroids and steroid derivatives |
| Homocapsaicin | 0.008775 | 0.03562 | -0.1052 | 342.2017 | Phenols |
| Hydrazinonicotinamide | 0.001166 | 0.008884 | -0.0885 | 194.1038 | Pyridines and derivatives |
| N2-Succinyl-L-ornithine | 0.000921 | 0.007641 | -0.0923 | 233.1133 | Carboxylic acids and derivatives |
| (S)-2-Methyl-1-butanol O-beta-D-Glucopyranoside | 0.000103 | 0.001263 | 0.0731 | 545.2775 | Fatty Acyls |
| 5-methoxy-6-(2-propenyl)-1,3-benzodioxole | 0.002214 | 0.01066 | 0.1286 | 237.0759 | Benzodioxoles |
| Phenylacetylglycine | 2.69E-06 | 0.000193 | 0.0869 | 226.1074 | Carboxylic acids and derivatives |
| 4-Hydroxyquinoline | 0.003817 | 0.02002 | 0.0793 | 146.0601 | Quinolines and derivatives |
| 3,5,6-Trihydroxy-5-(hydroxymethyl)-2-methoxy-2-cyclohexen-1-one | 2.01E-05 | 0.000443 | 0.0881 | 249.061 | Organooxygen compounds |
| S-(N,N-Diethylcarbamoyl)glutathione | 0.01291 | 0.0466 | -0.1038 | 439.186 | Carboxylic acids and derivatives |
| Tetranorprostanedioic acid | 0.000642 | 0.00602 | -0.093 | 326.2323 | Fatty Acyls |
| LysoPE(20:2(11Z,14Z)/0:0) | 0.002197 | 0.01061 | -0.087 | 486.2982 | Glycerophospholipids |
| PE(18:2/0:0) | 0.003846 | 0.01539 | -0.0836 | 476.2769 | |
| Maltotriose | 0.003367 | 0.01395 | -0.0821 | 539.137 | Organooxygen compounds |
| C75 trans | 0.000177 | 0.001797 | 0.0899 | 299.1495 | Lactones |

| | | | | | |
|---|---|---|---|---|---|
| 11-Hydroxyandrosterone | 0.000198 | 0.002749 | 0.0809 | 307.2266 | Steroids and steroid derivatives |
| Abt-751 | 0.000569 | 0.004051 | -0.088 | 406.0643 | Benzene and substituted derivatives |
| Macimorelin | 0.008272 | 0.03416 | -0.0994 | 538.2506 | Carboxylic acids and derivatives |
| 5-Hydroxymethyl-4-methyluracil | 0.004913 | 0.02373 | 0.0914 | 174.0874 | Diazines |
| Proline betaine | 4.41E-05 | 0.000722 | -0.0781 | 188.0919 | Carboxylic acids and derivatives |
| PGF2a ethanolamide | 0.000501 | 0.005174 | 0.0869 | 439.3164 | Fatty Acyls |
| PC(16:0/0:0) | 0.001229 | 0.009199 | -0.0645 | 518.3214 | Glycerophospholipids |
| 3,4-Methylenedioxyamphetamine | 0.00386 | 0.02016 | 0.0892 | 197.1286 | Benzodioxoles |
| 8-Methoxykynurenate | 0.002133 | 0.01338 | 0.101 | 261.0872 | Quinolines and derivatives |
| Valylglutamic acid | 0.009456 | 0.03756 | 0.1208 | 310.1394 | Carboxylic acids and derivatives |
| Triletide | 0.001885 | 0.009502 | 0.1033 | 486.2196 | Carboxylic acids and derivatives |
| Indicine | 0.001805 | 0.009208 | -0.103 | 336.1194 | |
| Methionine sulfone | 0.000127 | 0.001452 | 0.0797 | 180.0326 | Carboxylic acids and derivatives |
| Dehydrozingerone | 0.005634 | 0.01994 | 0.1332 | 237.0762 | Cinnamic acids and derivatives |
| 1-[3-[2-[5-(oxan-4-yl)-1,3,4-oxadiazol-2-yl]ethyl]piperidin-1-yl]ethanone | 0.000127 | 0.002085 | -0.0773 | 308.1966 | |
| Indole | 0.002536 | 0.01499 | 0.0865 | 118.0654 | Indoles and derivatives |
| 12-Hydroxydodecanoic acid | 5.22E-05 | 0.001183 | 0.0921 | 255.1338 | Hydroxy acids and derivatives |
| Dehydrocurdione | 0.005146 | 0.02453 | -0.0761 | 235.1692 | Prenol lipids |
| Apo-8'-capsorubinal | 0.000207 | 0.002837 | 0.0709 | 466.3277 | Prenol lipids |
| 2-Aminoheptanedioic acid | 1.59E-05 | 0.000554 | 0.0809 | 158.0813 | Carboxylic acids and derivatives |
| Kaempferide | 0.009522 | 0.02936 | -0.1052 | 299.0554 | Flavonoids |
| (R)-Pterosin B | 0.000952 | 0.007768 | -0.069 | 459.2478 | Indanes |
| Metolachlor Morpholinone | 0.000625 | 0.005918 | 0.096 | 251.1753 | |
| 2,3-Diaminopyridine | 0.005136 | 0.02451 | -0.0864 | 110.0716 | |
| Norhygrine | 6.93E-05 | 0.000966 | 0.1053 | 172.0969 | Organooxygen compounds |
| Ganoderic acid DM | 0.001185 | 0.008969 | 0.0853 | 469.3275 | Prenol lipids |
| Atenolol | 0.000972 | 0.007848 | -0.0763 | 267.1703 | Benzene and substituted derivatives |
| PE(17:1/0:0) | 0.01711 | 0.04506 | 0.1113 | 464.2766 | |
| Tsangane L 3-glucoside | 0.008561 | 0.03504 | 0.1014 | 397.2171 | Prenol lipids |
| Avocadyne | 0.000763 | 0.006699 | 0.0811 | 307.2265 | Fatty Acyls |
| Thr Asp Leu | 0.01372 | 0.04844 | -0.0903 | 348.1763 | |
| Isoquinoline | 9.44E-05 | 0.001738 | 0.0857 | 130.0653 | Isoquinolines and derivatives |
| Capsaicin | 0.002936 | 0.01668 | -0.0731 | 328.1865 | Phenols |
| Daidzein | 0.000408 | 0.004558 | -0.0593 | 255.0652 | Isoflavonoids |
| Glutaminylphenylalanine | 0.001032 | 0.00815 | -0.0828 | 326.1709 | Carboxylic acids and derivatives |
| Amodiaquine | 2.56E-05 | 0.000509 | 0.0712 | 400.1468 | Quinolines and derivatives |
| [6]-Gingerdione | 0.00595 | 0.02081 | 0.1046 | 629.3295 | Phenols |
| 24-Hydroxycholesterol | 0.00102 | 0.008094 | 0.0751 | 403.3531 | Steroids and steroid derivatives |
| 3-hydroxytridecanoic acid | 0.000289 | 0.002518 | 0.0955 | 275.1859 | Fatty Acyls |
| Citrazinic Acid | 3.52E-06 | 0.000146 | 0.1029 | 154.0135 | |
| Dihomo-gamma-linolenoylethanolamide | 0.000527 | 0.005352 | 0.069 | 367.3316 | Organonitrogen compounds |
| L-alpha-Aminobutyric acid | 0.000994 | 0.007956 | 0.0742 | 104.071 | Carboxylic acids and derivatives |
| Gibberellin A29-catabolite | 0.00321 | 0.0135 | -0.0887 | 383.0911 | Prenol lipids |
| 7,4'-Dihydroxyflavone | 0.000306 | 0.002614 | -0.0693 | 253.0502 | Flavonoids |

| | | | | | |
|---|---|---|---|---|---|
| 5'-Guanidinonaltrindole | 0.005254 | 0.019 | 0.0976 | 470.2244 | Morphinans |
| Irinotecan | 0.000577 | 0.004086 | 0.0904 | 567.2595 | Camptothecins |
| Pregnene | 0.001477 | 0.01041 | 0.0733 | 309.2575 | Steroids and steroid derivatives |
| N-Arachidonoyl Isoleucine | 8.04E-05 | 0.001588 | 0.0602 | 440.3141 | Carboxylic acids and derivatives |
| L-Histidine | 0.005136 | 0.02451 | -0.0711 | 156.0769 | Carboxylic acids and derivatives |
| Dazmegrel | 9.57E-05 | 0.001208 | 0.0888 | 320.0803 | Indoles and derivatives |
| Nitrendipine | 4.83E-06 | 0.000177 | -0.0799 | 359.1255 | Pyridines and derivatives |
| Dodeca-4,6,8-trienedioylcarnitine | 0.000512 | 0.003785 | -0.0906 | 388.1718 | Fatty Acyls |
| Glycyl-Glutamine | 2.72E-05 | 0.000777 | 0.0837 | 186.0874 | Carboxylic acids and derivatives |
| 3-Hydroxybenzaldehyde | 0.0109 | 0.04128 | 0.0757 | 123.0443 | |
| 4-Thiazolidinecarboxylic acid, 3-formyl-2,2,5,5-tetramethyl- | 0.004151 | 0.02112 | 0.0923 | 281.092 | |
| LysoPC(18:1(9Z)/0:0) | 0.01067 | 0.04076 | -0.0725 | 544.3398 | Glycerophospholipids |
| 3-Carboxy-4-Methyl-5-Propyl-2-Furanpropanoic Acid | 0.000206 | 0.001999 | -0.085 | 239.0918 | Fatty Acyls |
| 3b-Hydroxy-5-cholenoic acid | 0.003616 | 0.0192 | -0.0696 | 357.2788 | Steroids and steroid derivatives |
| Rhodiocyanoside A | 0.000233 | 0.002186 | -0.0922 | 258.0978 | |
| Prolyl-Arginine | 0.007933 | 0.0332 | 0.0858 | 272.1715 | Carboxylic acids and derivatives |
| Lysylproline | 0.000721 | 0.006464 | -0.0787 | 282.1195 | Carboxylic acids and derivatives |
| Oxyphencyclimine | 0.000657 | 0.006094 | -0.0783 | 389.1817 | Diazines |
| Cytosine deoxyribonucleoside | 0.000217 | 0.002923 | 0.0714 | 260.124 | Pyrimidine nucleosides |
| Aspartyl-Leucine | 0.001539 | 0.008192 | -0.0885 | 245.1137 | Carboxylic acids and derivatives |
| Quisultazine | 0.003345 | 0.0139 | 0.0913 | 450.1102 | Benzothiazines |
| 2-Methylhippuric acid | 8.72E-05 | 0.00167 | 0.0884 | 176.0707 | Benzene and substituted derivatives |
| Tirofiban | 0.01233 | 0.03571 | -0.109 | 475.2039 | Carboxylic acids and derivatives |
| 5-Alpha-Pregnan-3-BETA,17,21-Triol-11,20-Dione | 3.60E-05 | 0.000918 | 0.0671 | 347.2192 | |
| Pentaerythritol mononitrate | 0.001476 | 0.01041 | 0.1062 | 146.0437 | Organic oxoanionic compounds |
| Revefenacin | 0.005362 | 0.01926 | -0.0871 | 596.3217 | Piperidines |
| Asn Glu Phe | 0.000938 | 0.007691 | -0.0819 | 409.1716 | |
| 1-[6-(2-Carboxypyrrolidin-1-yl)-6-oxohexanoyl]pyrrolidine-2-carboxylic acid | 0.000621 | 0.004308 | 0.085 | 321.1449 | Carboxylic acids and derivatives |
| 2,4,5,7alpha-Tetrahydro-1,4,4,7a-tetramethyl-1H-inden-2-ol | 0.01053 | 0.03153 | -0.1277 | 237.1491 | Organooxygen compounds |
| Muscimol | 0.004748 | 0.02313 | 0.0808 | 292.1025 | Organonitrogen compounds |
| DG(18:3(9Z,12Z,15Z)/16:0/0:0) | 0.00217 | 0.01352 | 0.0729 | 613.4795 | Fatty Acyls |
| N-Palmitoyl Lysine | 8.59E-05 | 0.001654 | 0.0553 | 385.3423 | Carboxylic acids and derivatives |
| Neplanocin A | 0.01036 | 0.04004 | 0.096 | 296.1353 | Nucleoside and nucleotide analogues |
| Ser Lys | 0.004527 | 0.02248 | -0.089 | 234.1449 | |
| Epsilon-Tocopherol | 0.001116 | 0.006547 | -0.0807 | 455.3154 | Prenol lipids |
| 3-Hydroxy-5,5,8a-Trimethyl-3,4,4a,6,7,8-Hexahydronaphthalene-2-Carboxylic Acid | 0.000536 | 0.005373 | 0.0793 | 261.1444 | |
| Coumafuryl | 0.000302 | 0.002593 | 0.0847 | 343.0839 | Coumarins and derivatives |
| (3beta,17alpha,23S)-17,23-Epoxy-3,28,29-trihydroxy-27-norlanost-8-en-24-one | 0.006933 | 0.02332 | 0.1012 | 519.3309 | Prenol lipids |
| 3-Indoleacetic Acid | 0.000358 | 0.002911 | 0.0941 | 174.055 | Indoles and derivatives |
| Phenylalanylglutamic acid | 0.01228 | 0.03562 | 0.0949 | 275.1033 | Carboxylic acids and derivatives |
| Tyrosyl-Glutamate | 0.000519 | 0.003814 | 0.0763 | 291.0983 | Carboxylic acids and derivatives |
| Isovalerylcarnitine | 0.00078 | 0.005046 | 0.0756 | 535.3266 | Fatty Acyls |
| PC(15:0/0:0) | 0.005625 | 0.02615 | -0.0688 | 482.324 | Glycerophospholipids |
| Lucidenic acid M | 0.000381 | 0.004342 | 0.0665 | 462.2955 | Flavonoids |

| | | | | | |
|---|---|---|---|---|---|
| (8R,9S,10R,13S,14S,17S)-2,2,17-Trihydroxy-10,13-dimethyl-6,7,8,9,11,12,14,15,16,17-decahydro-1H-cyclopenta[a]phenanthren-3-one | 1.37E-05 | 0.000353 | 0.0692 | 365.1961 | Steroids and steroid derivatives |
| Arginine ornithine | 1.86E-05 | 0.000613 | 0.0777 | 271.1901 | Carboxylic acids and derivatives |
| S-Sulfo-L-Cysteine | 0.002188 | 0.01059 | -0.0781 | 199.9683 | Carboxylic acids and derivatives |
| DG(18:2(10E,12Z)+=O(9)/8:0/0:0) | 0.001971 | 0.01283 | 0.0853 | 494.3612 | |
| Cinncassiol D2 | 0.003969 | 0.01568 | -0.0997 | 405.1688 | Prenol lipids |
| Betaxolol | 0.003363 | 0.01824 | 0.0892 | 352.1865 | Phenols |
| 3'-O-Methylinosine | 0.00243 | 0.01138 | 0.0938 | 281.0886 | |
| Himbacine | 7.00E-05 | 0.001451 | 0.0546 | 363.3004 | Naphthofurans |
| Beta-Tyrosine | 9.54E-06 | 0.000421 | 0.079 | 164.0705 | Carboxylic acids and derivatives |
| 20-HETE ethanolamide | 7.99E-05 | 0.001582 | 0.0509 | 381.311 | Organonitrogen compounds |
| Herierin III | 0.000128 | 0.002085 | 0.0786 | 212.0918 | Pyrans |
| P-Octopamine | 0.002393 | 0.01444 | 0.0936 | 307.1649 | Phenols |
| Daunomycinone | 0.0145 | 0.04007 | 0.1297 | 443.0993 | Naphthacenes |
| 2-Amino-3-cyclohexylpropanoic acid | 0.000175 | 0.001788 | 0.0971 | 216.1234 | Carboxylic acids and derivatives |
| Glycyl-Lysine | 0.002056 | 0.01018 | -0.0853 | 202.1188 | Carboxylic acids and derivatives |
| Lichenin | 0.003812 | 0.02001 | 0.0831 | 180.088 | Organooxygen compounds |
| Tyr Pro Val | 8.81E-06 | 0.000396 | 0.0678 | 378.2021 | |
| N-Acetyl-L-Glutamic Acid | 9.15E-07 | 7.14E-05 | 0.0584 | 188.0555 | Carboxylic acids and derivatives |
| Androst-5-ene-3beta,17beta-diol | 0.002038 | 0.01305 | 0.0841 | 291.2316 | Steroids and steroid derivatives |
| INDOPHENOL | 7.99E-05 | 0.001582 | 0.0787 | 232.0968 | Organonitrogen compounds |
| Cepagenin | 0.001779 | 0.01191 | 0.0748 | 411.2851 | Prenol lipids |
| N-Heptanoylglycine | 0.004845 | 0.0235 | 0.1282 | 205.1547 | Carboxylic acids and derivatives |
| Bedoradrine | 0.005543 | 0.01969 | -0.0853 | 449.203 | Tetralins |
| P-Aminobenzoic acid | 0.000238 | 0.003126 | 0.0759 | 138.0551 | Benzene and substituted derivatives |
| Ibufenac | 0.001147 | 0.008774 | 0.0835 | 193.1225 | Benzene and substituted derivatives |
| 7a-Hydroxy-cholestene-3-one | 0.003834 | 0.02006 | 0.0793 | 423.3215 | Steroids and steroid derivatives |
| D-Galactaric acid | 4.85E-05 | 0.000771 | -0.0734 | 209.0294 | Organooxygen compounds |
| 6-Ethylchenodeoxycholic acid | 0.003919 | 0.01556 | 0.1046 | 441.2986 | Steroids and steroid derivatives |
| Cephapirin | 0.0164 | 0.04372 | 0.0911 | 404.0401 | Carboxylic acids and derivatives |
| Glu Lys Leu | 0.01095 | 0.04133 | -0.0939 | 389.2393 | |
| (E)-10-Hydroxy-8-decenoic acid | 6.27E-05 | 0.000904 | 0.0811 | 231.1231 | Hydroxy acids and derivatives |
| 6b-Angeloyl-3b,8b,9b-trihydroxy-7(11)-eremophilen-12,8-olide | 0.000319 | 0.00269 | 0.0617 | 379.1753 | Prenol lipids |
| 3-Hydroxypropyl methacrylate | 0.000823 | 0.005256 | 0.0842 | 287.1496 | Carboxylic acids and derivatives |
| 2-Keto-glutaramic acid | 9.50E-05 | 0.001205 | 0.0809 | 144.029 | Keto acids and derivatives |
| N-Eicosapentaenoyl Glycine | 0.001047 | 0.008236 | 0.0751 | 377.2795 | Carboxylic acids and derivatives |
| Biotin | 0.000183 | 0.001838 | 0.0842 | 243.0804 | Biotin and derivatives |
| Arginylleucine | 0.0173 | 0.04538 | 0.1097 | 268.1776 | Carboxylic acids and derivatives |
| Trigonelline | 0.000504 | 0.005194 | 0.0588 | 138.0551 | |
| N-Acetylleucine | 1.31E-05 | 0.000344 | 0.0594 | 172.0969 | Carboxylic acids and derivatives |
| 2-Oxoarginine | 0.004423 | 0.01685 | -0.072 | 154.0611 | Keto acids and derivatives |
| Cortisone | 0.001565 | 0.01084 | -0.0804 | 424.2074 | Steroids and steroid derivatives |
| Methylisocitric acid | 0.006066 | 0.02758 | 0.0971 | 224.0778 | Carboxylic acids and derivatives |
| 7-Sulfocholic acid | 5.57E-06 | 0.000196 | 0.0396 | 487.236 | Steroids and steroid derivatives |

| | | | | | |
|---|---|---|---|---|---|
| (1xi,2xi)-1-(4-Hydroxyphenyl)-1,2,3-propanetriol 3-O-beta-D-Glucopyranoside | 0.000512 | 0.005243 | 0.0771 | 388.1599 | Fatty Acyls |
| His Trp | 0.01136 | 0.04249 | -0.0737 | 342.1563 | |
| Pro Val | 0.000536 | 0.005373 | 0.0748 | 215.1391 | |
| Cetirizine | 0.000225 | 0.00213 | -0.0812 | 425.1016 | Benzene and substituted derivatives |
| Erythromycin A enol ether | 0.01097 | 0.03261 | -0.0913 | 752.3935 | Organooxygen compounds |
| Curdione | 0.000248 | 0.003225 | 0.0689 | 219.1744 | Prenol lipids |
| Ptaquiloside | 0.002233 | 0.01374 | 0.076 | 398.1919 | Organooxygen compounds |
| N-Decanoyl-DL-Homoserine Lactone | 0.000817 | 0.007053 | 0.0772 | 256.1907 | |
| LysoPC(20:4(5Z,8Z,11Z,14Z)/0:0) | 0.003571 | 0.01904 | -0.0628 | 544.3371 | Glycerophospholipids |
| LysoPE(18:1(9Z)/0:0) | 0.001988 | 0.009932 | -0.061 | 460.2823 | Glycerophospholipids |
| 3h-Adrenaline | 0.001191 | 0.008999 | 0.0716 | 203.1391 | Organooxygen compounds |
| Homoeriodictyol | 0.00457 | 0.01725 | -0.09 | 301.0712 | Flavonoids |
| Helenalin | 0.00099 | 0.007939 | 0.0845 | 263.1277 | Prenol lipids |
| Nandrolone | 5.21E-05 | 0.001183 | 0.0686 | 292.2269 | Steroids and steroid derivatives |
| 1-Nonadecanoyl-glycero-3-phosphoethanolamine | 0.004555 | 0.01721 | -0.072 | 476.3132 | Glycerophospholipids |
| (3beta,17alpha,23R)-17,23-Epoxy-3,29-dihydroxy-27-norlanost-8-ene-15,24-dione | 4.76E-06 | 0.000176 | 0.0544 | 517.3156 | Prenol lipids |
| Cinobufagin | 1.24E-05 | 0.000333 | 0.0566 | 487.2359 | Steroids and steroid derivatives |
| 5-Hydroxymethyluracil | 0.008704 | 0.0354 | 0.1074 | 184.0718 | Diazines |
| 5-Hydroxymethyl-2-furancarboxaldehyde | 0.00042 | 0.00465 | 0.0657 | 168.0656 | Organooxygen compounds |
| Tyrosyl-Serine | 0.01794 | 0.04663 | 0.0921 | 267.0983 | Carboxylic acids and derivatives |
| Pyridoxal | 6.09E-05 | 0.001314 | 0.0722 | 168.0656 | Pyridines and derivatives |
| 4-Acetylbutyrate | 3.42E-05 | 0.000606 | 0.086 | 175.0602 | Fatty Acyls |
| Acetyl-DL-Leucine | 0.000475 | 0.005022 | 0.0584 | 174.1126 | Carboxylic acids and derivatives |
| Phenylalanylaspartic acid | 0.000424 | 0.004672 | -0.0628 | 281.1131 | Carboxylic acids and derivatives |
| 3a,6b,7a,12a-Tetrahydroxy-5b-cholanoic acid | 0.004451 | 0.02217 | -0.0787 | 424.2804 | Organooxygen compounds |
| Dimethyl dimethoxy biphenyl | 0.001861 | 0.00941 | -0.0737 | 529.2615 | Benzene and substituted derivatives |
| 2-Hydroxypropyl methacrylamide | 1.36E-06 | 8.46E-05 | 0.09 | 188.0919 | Carboxylic acids and derivatives |
| Gamma-Glutamyltryptophan | 0.000114 | 0.001954 | -0.0524 | 334.1396 | Carboxylic acids and derivatives |
| 1-Nonadecanoyl-glycero-3-phosphoserine | 0.001484 | 0.007995 | -0.0695 | 574.2907 | Glycerophospholipids |
| Oleoyl Ethanolamide | 7.51E-06 | 0.000367 | 0.0488 | 326.3052 | Organonitrogen compounds |
| Vanillylamine | 0.000194 | 0.002716 | 0.0783 | 136.0758 | Phenols |
| Hydroxyzine | 0.002011 | 0.009999 | 0.073 | 373.172 | Benzene and substituted derivatives |
| 3-Amino-5-hydroxybenzoic acid | 0.00615 | 0.02786 | 0.0894 | 154.05 | Benzene and substituted derivatives |
| Deoxyribose | 4.52E-06 | 0.000171 | 0.0735 | 133.0494 | Organooxygen compounds |
| L-cis-Cyclo(aspartylphenylalanyl) | 0.006438 | 0.02199 | -0.0864 | 261.0876 | Carboxylic acids and derivatives |
| Glutamyltryptophan | 0.000146 | 0.001594 | -0.0616 | 332.1246 | Carboxylic acids and derivatives |
| Alpha-Zearalenol | 6.44E-05 | 0.000919 | 0.072 | 365.1598 | Macrolides and analogues |
| 7(14)-Bisabolene-2,3,10,11-tetrol | 0.001076 | 0.006385 | 0.0866 | 317.1962 | Prenol lipids |
| Tetradecanedioic acid | 4.28E-05 | 0.000708 | 0.0707 | 257.1752 | Fatty Acyls |
| 3-(3-Oxobutanoyloxy)butanoic acid | 0.000914 | 0.007619 | 0.0787 | 377.1453 | Keto acids and derivatives |
| Skimmianine | 0.000419 | 0.004645 | 0.0737 | 277.1182 | Quinolines and derivatives |
| N-Acetyl-D-mannosamine | 0.001815 | 0.01208 | 0.0506 | 204.0867 | Organooxygen compounds |
| HBOA trihexose | 0.001018 | 0.006146 | -0.0596 | 202.1076 | Carboxylic acids and derivatives |
| 3beta-Hydroxy-17-(1H-imidazol-1-yl)androsta-5,16-diene | 0.000342 | 0.002819 | -0.0608 | 375.1837 | Steroids and steroid derivatives |

| | | | | | |
|---|---|---|---|---|---|
| Reserpine | 0.008187 | 0.02645 | -0.0812 | 589.2578 | Yohimbine alkaloids |
| 5-(Galactosylhydroxy)-L-Lysine | 0.004536 | 0.02251 | 0.0771 | 325.1603 | Fatty Acyls |
| 3-Hydroxyvaleric acid | 0.000192 | 0.002707 | -0.0611 | 160.0969 | Fatty Acyls |
| Phenacetin | 0.001041 | 0.008205 | 0.0711 | 180.102 | Benzene and substituted derivatives |
| N-Arachidonoyl Phenylalanine | 0.0131 | 0.03727 | -0.0678 | 450.2999 | Carboxylic acids and derivatives |
| Tyramine glucuronide | 0.000541 | 0.003915 | -0.0796 | 294.0979 | Organooxygen compounds |
| Glutamyllysine | 0.004341 | 0.02177 | -0.0859 | 258.1448 | Carboxylic acids and derivatives |
| 2,3-Dimethoxy-5-methyl-6-(9'-carboxynonyl)-1,4-benzoquinone | 0.000922 | 0.007641 | 0.0675 | 375.1775 | Prenol lipids |
| Gamma-Glutamylproline | 0.000925 | 0.007651 | 0.0546 | 245.1132 | Carboxylic acids and derivatives |
| Imazamox | 0.01406 | 0.03923 | -0.0914 | 350.135 | Carboxylic acids and derivatives |
| LysoPE(0:0/18:0) | 0.004414 | 0.01684 | -0.0673 | 462.298 | Glycerophospholipids |
| 5,7-Dimethoxyflavone | 0.000205 | 0.001996 | 0.0675 | 327.0899 | Flavonoids |
| N-Palmitoyl Threonine | 0.000332 | 0.00394 | 0.0558 | 375.3216 | Carboxylic acids and derivatives |
| Clozapine | 0.007486 | 0.02468 | -0.0813 | 371.127 | Benzodiazepines |
| Phenylacetic Acid | 0.007546 | 0.02483 | 0.1065 | 135.044 | Benzene and substituted derivatives |
| 7-Ketodeoxycholic acid | 0.000289 | 0.003578 | 0.046 | 371.2579 | Steroids and steroid derivatives |
| Epiheterodendrin | 0.000719 | 0.006464 | 0.0745 | 226.1074 | Organooxygen compounds |
| 3-Pyridylacetic acid | 0.00138 | 0.007578 | 0.0712 | 319.0961 | Pyridines and derivatives |
| Malonic semialdehyde | 0.0142 | 0.03947 | 0.0781 | 235.0452 | Organooxygen compounds |
| MG(0:0/5-iso PGF2VI/0:0) | 0.008333 | 0.02679 | 0.0927 | 381.2275 | |
